# Genome-wide association meta-analysis identifies 48 risk variants and highlights the role of the stria vascularis in hearing loss

**DOI:** 10.1016/j.ajhg.2022.04.010

**Published:** 2022-05-16

**Authors:** Natalia Trpchevska, Maxim B. Freidin, Linda Broer, Berthe C. Oosterloo, Shuyang Yao, Yitian Zhou, Barbara Vona, Charles Bishop, Argyro Bizaki-Vallaskangas, Barbara Canlon, Fabio Castellana, Daniel I. Chasman, Stacey Cherny, Kaare Christensen, Maria Pina Concas, Adolfo Correa, Ran Elkon, Estonian Biobank Research Team, Andres Metspalu, Mari Nelis, Reedik Mägi, Tõnu Esko, Jonas Mengel-From, Yan Gao, Anne B.S. Giersch, Giorgia Girotto, Alexander Gudjonsson, Vilmundur Gudnason, Nancy L. Heard-Costa, Ronna Hertzano, Jacob v.B. Hjelmborg, Jens Hjerling-Leffler, Howard J. Hoffman, Jaakko Kaprio, Johannes Kettunen, Kristi Krebs, Anna K. Kähler, Francois Lallemend, Lenore J. Launer, I-Min Lee, Hampton Leonard, Chuan-Ming Li, Hubert Lowenheim, Patrik K.E. Magnusson, Joyce van Meurs, Lili Milani, Cynthia C. Morton, Antti Mäkitie, Mike A. Nalls, Giuseppe Giovanni Nardone, Marianne Nygaard, Teemu Palviainen, Sheila Pratt, Nicola Quaranta, Joel Rämö, Elmo Saarentaus, Rodolfo Sardone, Claudia L. Satizabal, John M. Schweinfurth, Sudha Seshadri, Eric Shiroma, Eldad Shulman, Eleanor Simonsick, Christopher Spankovich, Anke Tropitzsch, Volker M. Lauschke, Patrick F. Sullivan, Andre Goedegebure, Christopher R. Cederroth, Frances M.K. Williams, Andries Paul Nagtegaal

**Affiliations:** 1 Department of Physiology and Pharmacology, Karolinska Institutet, 17177 Stockholm, Sweden; 2 Department of Twin Research and Genetic Epidemiology, King’s College London, London, UK; 3 Department of Internal Medicine, Erasmus Medical Center, 3015 CE Rotterdam, the Netherlands; 4 Department of Otorhinolaryngology, Erasmus Medical Center, 3015 CE Rotterdam, the Netherlands; 5 Department of Medical Epidemiology and Biostatistics, Karolinska Institutet, 17177 Stockholm, Sweden; 6 Institute of Human Genetics, University Medical Center Göttingen, 37073 Göttingen, Germany; 7 Institute for Auditory Neuroscience and InnerEarLab, University Medical Center Göttingen, 37075 Göttingen, Germany; 8 Department of Otolaryngology–Head & Neck Surgery, University of Tübingen Medical Center, 72076 Tübingen, Germany; 9 Department of Otolaryngology and Communicative Sciences, The University of Mississippi Medical Center, Jackson, MS 39216, USA; 10 Department of Otolaryngology, University of Tampere, 33100 Tampere, Finland; 11 Pirkanmaan Sairaanhoitopiiri, 33520 Tampere, Finland; 12 Unit of Data Sciences and Technology Innovation for Population Health, National Institute of Gastroenterology “Saverio de Bellis”, Research Hospital, Castellana Grotte, 70124 Bari, Italy; 13 Division of Preventative Medicine, Brigham and Women’s Hospital, Harvard Medical School, Boston, MA 02115, USA; 14 Broad Institute of MIT and Harvard, Cambridge, MA 02142, USA; 15 Department of Anatomy and Anthropology and Department of Epidemiology and Preventive Medicine, Sackler Faculty of Medicine, Tel Aviv University, 69978 Tel Aviv, Israel; 16 The Danish Twin Registry, Department of Public Health, University of Southern Denmark, 5000 Odense C, Denmark; 17 Department of Clinical Genetics, Odense University Hospital, 5000 Odense C, Denmark; 18 Department of Clinical Biochemistry and Pharmacology, Odense University Hospital, 5000 Odense C, Denmark; 19 Institute for Maternal and Child Health — IRCCS, Burlo Garofolo, 34127 Trieste, Italy; 20 Jackson Heart Study, The University of Mississippi Medical Center, Jackson, MS 39216, USA; 21 Department of Human Molecular Genetics & Biochemistry, Sackler School of Medicine, Tel Aviv University, 69978 Tel Aviv, Israel; 22 Estonian Genome Centre, Institute of Genomics, University of Tartu, Tartu, Estonia; 23 Department of Population Health Science, University of Mississippi Medical Center, Jackson, MS 39216, USA; 24 Department of Pathology, Brigham and Women’s Hospital, Harvard Medical School, Boston, MA 02115, USA; 25 Department of Medicine, Surgery and Health Sciences, University of Trieste, 34139 Trieste, Italy; 26 Icelandic Heart Association, 201 Kopavogur, Iceland; 27 Faculty of Medicine, University of Iceland, 101 Reykjavik, Iceland; 28 Department of Neurology, Boston University School of Medicine, Boston, MA 02118, USA; 29 Framingham Heart Study, Framingham, MA 01702, USA; 30 Department of Otorhinolaryngology-Head and Neck Surgery, University of Maryland Baltimore, Baltimore, MD 21201, USA; 31 Department of Anatomy and Neurobiology, University of Maryland Baltimore, Baltimore, MD 21201, USA; 32 Institute for Genome Sciences, University of Maryland Baltimore, Baltimore, MD 21201, USA; 33 Department of Medical Biochemistry and Biophysics, Karolinska Institutet, 17177 Stockholm, Sweden; 34 Division of Scientific Programs, Epidemiology and Statistics Program, National Institute on Deafness and Other Communications Disorders (NIDCD), NIH, Bethesda, MD 20892, USA; 35 Institute for Molecular Medicine Finland (FIMM), University of Helsinki, 00014 Helsinki, Finland; 36 Computational Medicine, Center for Life Course Health Research, Faculty of Medicine, University of Oulu, 90220 Oulu, Finland; 37 Biocenter Oulu, University of Oulu, 90220 Oulu, Finland; 38 Finnish Institute for Health and Welfare, 00271 Helsinki, Finland; 39 Department of Neuroscience, Karolinska Institutet, 17177 Stockholm, Sweden; 40 Laboratory of Epidemiology and Population Sciences, Intramural Research Program National Institute on Aging, Bethesda, MD 20892, USA; 41 Laboratory of Neurogenetics, National Institute on Aging, National Institutes of Health, Bethesda, MD 20892, USA; 42 Center for Alzheimer’s and Related Dementias, National Institutes of Health, Bethesda, MD 20892, USA; 43 Data Tecnica International, Glen Echo, MD 20812, USA; 44 Department of Obstetrics and Gynecology and of Pathology, Brigham and Women’s Hospital, Harvard Medical School, Boston, MA 02115, USA; 45 Manchester Centre for Audiology and Deafness, University of Manchester, Manchester M13 9PL, UK; 46 Department of Otorhinolaryngology - Head and Neck Surgery, University of Helsinki and Helsinki University Hospital, 00029 Helsinki, Finland; 47 Department of Communication Science & Disorders, University of Pittsburgh, Pittsburgh, PA 15260, USA; 48 Otolaryngology Unit, Department of Basic Medical Science, Neuroscience and Sense Organs, University of Bari Aldo Moro, 70121 Bari, Italy; 49 Glenn Biggs Institute for Alzheimer’s & Neurodegenerative Diseases and Department of Population Health Sciences, University of Texas Health Sciences Center, San Antonio, TX 78229, USA; 50 Laboratory of Epidemiology and Population Sciences, National Institute on Aging, Baltimore, MD 21224, USA; 51 Longitudinal Studies Section, Translational Gerontology Branch, National Institute on Aging, Baltimore, MD 21224, USA; 52 Department of Genetics, University of North Carolina, Chapel Hill, NC 27516, USA; 53 National Institute for Health Research (NIHR) Nottingham Biomedical Research Centre, Nottingham University Hospitals NHS Trust, Ropewalk House, NG1 5DU Nottingham, UK; 54 Hearing Sciences, Division of Clinical Neuroscience, School of Medicine, University of Nottingham, NG7 2UH Nottingham, UK

**Keywords:** hearing loss, ARHL, cochlea, genetics, GWAS, stria vascularis, spindle cells, root cells, basal cells, hair cells

## Abstract

Hearing loss is one of the top contributors to years lived with disability and is a risk factor for dementia. Molecular evidence on the cellular origins of hearing loss in humans is growing. Here, we performed a genome-wide association meta-analysis of clinically diagnosed and self-reported hearing impairment on 723,266 individuals and identified 48 significant loci, 10 of which are novel. A large proportion of associations comprised missense variants, half of which lie within known familial hearing loss loci. We used single-cell RNA-sequencing data from mouse cochlea and brain and mapped common-variant genomic results to spindle, root, and basal cells from the stria vascularis, a structure in the cochlea necessary for normal hearing. Our findings indicate the importance of the stria vascularis in the mechanism of hearing impairment, providing future paths for developing targets for therapeutic intervention in hearing loss.

## Introduction

The number of people with mild-to-complete hearing impairment is projected to increase to an estimated 2.45 billion worldwide by 2050, principally driven by age-related hearing impairment (ARHI).^1^ Hearing impairment is ranked third for causes of global years lived with disability (YLDs) across all ages and the leading cause of YLDs in those older than 70, as compared with all other disease categories.^1^ The overall global cost of unaddressed hearing loss exceeds $981 billion annually.^2^ ARHI has been associated with social withdrawal, depression, anxiety, as well as cognitive decline and dementia.^3^ There is no preventive treatment for hearing decline and therapeutics are currently available only in the form of hearing aids or cochlear implants. Moreover, the impact of untreated hearing loss remains underestimated as governmental and industry incentives are still very low in comparison to other diseases of equal prevalence.^4^

Hearing thresholds tend to deteriorate gradually with age and ARHI is typically more pronounced in the higher frequencies. Knowledge of the pathophysiological mechanisms of hearing loss derives primarily from animal studies (particularly mouse models^5^), as well as clinical research on specific families with hearing loss.^6^ Hearing loss is moderately heritable, with recent studies attributing 36%–70% of the variation in the heritability of hearing impairment to additive genetic effects.^7^^,^^8^ Large genome-wide association studies (GWASs) have been recently conducted: UK Biobank (n = 87,056 individuals with self-reported hearing difficulty) revealed 44 independent loci associated with self-reported hearing difficulty and confirmed that hearing loss is a complex polygenic disorder.^9^ A combined Icelandic cohort and UK Biobank (n = 121,934 individuals identified through pure-tone audiograms and self-reported hearing difficulty) yielded another 21 novel associations of which 13 were rare variants.^10^ Kalra et al. performed a multi-trait analysis of GWASs (MTAG)^11^ using UKB data from up to 337,000 participants with different hearing phenotypes and identified 8 novel hits supported with transcription data.^12^ However, many loci were not replicated, which may be explained by differences in phenotyping (ICD diagnoses, self-report, hearing thresholds assessed by audiometry), imbalanced sample size with UK Biobank predominating, statistical power, or ancestral differences between samples. While early-onset genetic hearing loss is determined by monogenic factors, ARHI appearing in late adulthood develops from the interaction of environmental and polygenic factors.^13^

**Figure 1:**
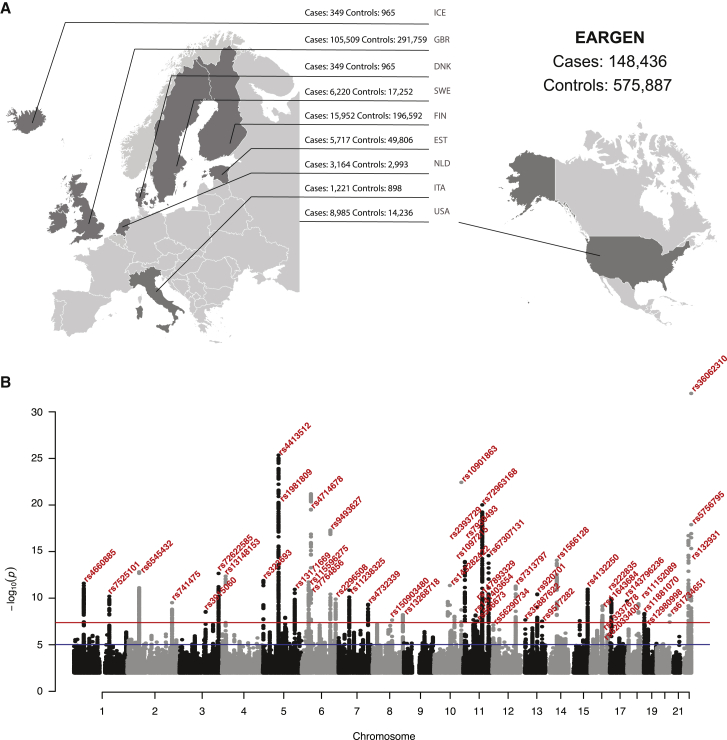
GWAS meta-analysis for ARHI (n = 723,266) (A) Origin of the datasets used for the meta-analysis of the ARHI GWAS: 8 European countries and the United States. (B) Manhattan plot displays all associations per variant ordered according to their genomic position on the x axis and showing the strength of the association with the −log_10_ transformed p values on the y axis. The threshold for genome-wide significance (p < 5 × 10^−8^) is indicated by the red line, while the blue line represents the suggestive threshold (p < 1 × 10^−5^).

In order to gain fundamental knowledge on the genetic basis of hearing loss, we conducted a meta-analysis of 17 hearing loss GWASs using both ICD diagnoses and self-reported hearing loss. The latter has been demonstrated to be a good proxy for formal hearing assessment.^14^ The study comprised 147,997 affected individuals and 575,269 control subjects including 60,941 affected individuals that were not in our previously published GWAS meta-analysis.^9^ We compiled a dataset comprising multiple different European and US population-based cohorts (Figure 1A, Table S1).

## Material and methods

### Study design and phenotyping

Adult male and female participants were included from the following 17 population-based cohort studies: Age, Genes/Environment Susceptibility - Reykjavik (AGES; n = 3,134), the Danish Twin Registry (DTR; n = 1,314), the Estonian Genome Center at the University of Tartu (EGCUT; n = 55,523), FinnGen (n = 212,544), Framingham Heart Study (FHS; n = 2,536), Health Aging and Body Composition (HABC; n = 1,288), Italian Network of Genetic Isolates - Friuli Venezia Giulia (INGI-FVG; n = 339), the Rotterdam Study (RS, cohorts 1–3; n = 6,157), the Salus in Apulia study (SA; n = 1,780; formerly known as Great Age study), Screening Across the Lifespan Twin (SALT; n = 9,565, and SALTY - young; n = 5,133), Screening Twin Adults: Genes and Environment (STAGE; n = 8,345), TwinsUK (n = 5,125), UK Biobank (UKBB; n = 392,143), and the Women’s Genome Health Study (WGHS; n = 18,340). All participants provided written informed consent; ethical approval was obtained locally. The declaration of Helsinki was adhered to. Phenotype was based on ICD9 & 10 diagnoses of hearing loss (EGCUT and FinnGen) or questions on hearing loss (all other cohorts). Mean age overall was 59.6. A detailed description of phenotype definition case control distribution and age range for each study can be found in Table S1. Study details and cohort descriptions are available in supplemental methods. An umbrella ethics license was granted by the local ethics committee, Regionala etikprövningsnämnden in Stockholm (2015/2129-31/1). Informed consent was obtained from all participants. Individual ethical license from all contributing cohorts are included in supplemental methods.

### Genome-wide association studies and meta-analysis

GWASs have been carried out for each cohort locally and summary statistics were collected for each study. Standardized quality control was performed using EasyQC software,^15^ followed by meta-analysis using METAL software.^16^ Briefly, the quality control steps with EasyQC were excluding monomorphic SNPs, SNP missingness <0.05, filtering out duplicate SNPs and SNPs with imputation score <0.5. After harmonizing allele coding and marker names, uniformed summary statistics were produced. LD score regression^17^ was applied to estimate the impact of population stratification and other confounders on test-statistic inflation for cohorts with sample size n > 5,000 for QC-ed and harmonized GWAS data. The genome build was hg19.

Meta-analysis of GWAS summary statistics was conducted using an inverse-variance-weighted fixed-effect model in METAL.^16^ To control for population stratification and other confounders, individual cohorts were adjusted for either genomic control (cohort with sample size >5,000) or intercept. A genome-wide significance threshold was defined as p < 5 × 10^−8^. Conditional and joint association analysis (COJO) was carried out to reveal independent lead SNPs for genome-wide significant loci using GCTA software.^18^ We randomly selected 50,000 individuals of European ancestry from UK Biobank as a reference sample for COJO. We examined whether the SNPs have been previously associated with hearing loss in a large-scale GWAS. A locus was designated “new” when LD with previous associated variants was <0.6. In the case of missense SNPs, the Combined Annotation Dependent Depletion (CADD) score (GRCh37-v1.6) was used to estimate the deleterious effect of the SNP.^19^

### Gene prioritization and pathway analysis

For gene prioritization in the genome-wide significant loci, we used MAGMA^20^ (significance threshold p < 2.66 × 10^−6^) implemented in FUMA^21^ and VEGAS2 software^22^ (Bonferroni corrected p < 0.05). We used the offline version of VEGAS2 and analyzed the most associated 10 SNPs flanking 10 kb upstream and downstream the genes. The list of genes was obtained from the VEGAS2 website (https://vegas2.qimrberghofer.edu.au/glist-hg19) and included 26,056 genes. Given that the number of genes in the output would depend on the analysis parameters, we chose to correct for multiple testing assuming 20,000 independent tests by Bonferroni approach.

Genes within 500 Mb from the top SNP were checked for any known association with hearing loss in either humans or mice. For that purpose, existing literature and the website of the International Mouse Phenotyping Consortium (www.mousephenotype.org) were consulted.^23^ The Shared Inner Ear Laboratory Database (SHIELD)^24^ was used to examine whether candidate genes were expressed in inner and outer hair cells of the cochlea in adult mice (P25–P30), designated positive when expression levels (measured by fluorescent intensity readings) exceeded 10.9, as described in the referenced paper.^25^

We used VEGAS2 for pathway analysis based on the results of gene prioritization as described above and the list of pathways provided as part of VEGAS2 distribution (https://vegas2.qimrberghofer.edu.au/biosystems20160324.vegas2pathSYM).^26^

### Variant analysis

All variants identified in genes of interest in the gnomAD v.2.1.1 were downloaded, and minor allele frequencies (MAFs) were filtered to select for variants that were common (MAF ≥1%) in the non-Finnish European (EUR) population. Transcripts were selected based on consensus with the Deafness Variation Database transcript catalog,^27^ where possible, or otherwise we used the longest transcript according to Ensembl. Variant effects were analyzed using an array of partially orthogonal computational prediction algorithms, PolyPhen-2,^28^ CADD,^19^ DANN,^29^ PROVEAN,^30^ REVEL,^31^ VEST3,^32^ and Eigen,^33^ that consider genetic, evolutionary, structural, and biochemical information to infer variant pathogenicity and deleteriousness. The individual algorithmic assessments were aggregated into a consensus ensemble score normalized to the range from zero (variant unanimously predicted to be deleterious) to one (unanimously predicted to be benign). The secondary structure of GJB2 was obtained from the Protein Data Bank (PDB: 2ZW3) and the structural consequences of *GJB2* variants were modeled using PyMOL v..1.1.

Fine-mapping was carried out using CAUSALdb-finemapping-pip pipeline (https://github.com/mulinlab/CAUSALdb-finemapping-pip). We analyzed 1 Mb regions surrounding lead SNPs in each genome-wide significant locus. The output includes credible sets and posterior probabilities for SNPs to be causal as per PAINTOR, CAVIARBF, and FINEMAP algorithms.

### Genetic correlations

In LD Hub^34^ we used LD score regression to estimate the genetic correlation between hearing loss and a range of other disorders and traits, to evaluate the extent of shared genetic architecture based on common gene variants and hypothesize about association with potential risk factors. After excluding all UK Biobank-based phenotypes, the list comprised 256 phenotypes. Significance was thus set at p < 2 × 10^−04^ after Bonferroni correction.

In the internal genome-wide association library (Omnibus data) collated by the Psychiatric Genomics Consortium, we used LDSC to get SNP-based genetic correlation between hearing loss and psychiatric and anthropometric traits.

### Expression data sets

We obtained expression specificity data in 37 GTEx v8^35^ human tissues processed by previous research.^36^ Briefly, the following tissue filters were applied: (1) tissues with fewer than 100 donors, (2) non-natural tissues (e.g., cancer tissue and cell lines), and (3) testis tissues (expression outlier). Since GTEx does not contain cochlear data, we sought expression data from 36,616 cells originating from 2 datasets from adult mouse cochlea (post-natal day 60, CBA/CaJ) published by Milon et al.^37^ (one from the stria vascularis and the other from spiral ganglion neuron [SGN]) summing to 15 different cell types (Table S2). Since these two datasets were genotyped using exactly the same technique in the same technical infrastructure, we merged them first by aggregating the count per gene per cell type and normalized to 1 TPM per cell type to account for the variation of cell counts per cell type in each dataset, while preserving the relative expression pattern per cell type (Table S2). Monocytes and neutrophils were found in both the stria vascularis and in the SGN data, and the correlations of normalized expression between the two datasets were high (0.90 for monocytes and 0.98 for neutrophils based on 15,798 genes), supporting the appropriateness of the merging step. Additionally, since the data from Milon et al. did not contain data from the organ of Corti (e.g., hair cells and and Deiters’ cells), we relied on single-cell data extracted by Ranum et al.^38^ (post-natal day 15, C3HeB/FeJ). We note that the murine cochlea is functionally mature at P14. Finally, we also used expression data from Zeisel et al.^39^ consisting in 160,796 cells of 39 broad cell types sampled from 19 regions in the entire mouse neural system (post-natal day P12–P30, as well as 6 and 8 weeks old, CD-1 and Swiss) that were processed to get cell type expression specificity^36^ (Table S2). Only genes with 1:1 orthology between human and mouse were preserved for calculating the expression specificity.

### Calculation of cell-type expression specificity

We processed and calculated the expression specificity as previously described.^36^ Briefly, in each tissue expression dataset (i.e., organ of Corti,^38^ stria vascularis/SGNs,^37^ and neural tissue^39^), we first aggregated the count per gene per cell type and excluded genes that are (1) not expressed in any cell type, (2) with duplicated identifier, or (3) not 1:1 orthologous between mouse and human. We then normalized the expression to 1 TPM (transcripts per million) per cell type. Next, gene expression specificity was calculated per gene per cell type as:

$$Specificity=\frac{Normalized\;expression\;in\;the\;cell\;type}{Sum\;of\;normalized\;expression\;in\;all\;cell\;types\;}$$

Specificity ranges from 0 to 1; a higher value indicates that the gene is more specific to the corresponding cell types compared to its expression profiles across all included cell types. We selected genes with the top 10% specificity values in each cell type as the gene list for the cell type that was used for the heritability enrichment analyses.

### SNP-heritability enrichment

We used MAGMA (v.1.08)^20^ and partitioned LDSC^40^ to evaluate whether the top 10% specifically expressed genes per tissue/cell type were enriched of the SNP-based h^2^ of hearing loss.

MAGMA evaluated whether the cell-type-specific genes were enriched in hearing loss gene-level associations in two steps. In the first step (SNP-wise gene analysis), we filtered out SNPs with minor allele frequency <1% and poor imputation quality (INFO < 0.6) from the ARHI summary statistics and calculated the p value for per gene association with hearing loss using SNP p values (35 kb upstream and 10 kb downstream window per gene).^36^ In the second step (gene-set analysis), the p values were converted to Z-scores, and one-sided tests were performed to compare whether the Z-scores in the gene set (i.e., cell-type-specific genes) were higher than those not in the gene set, which indicated enrichment of SNP-heritability in the gene set.^20^^,^^41^

We then applied partitioned LDSC adjusting for the baseline annotations.^42^ Partitioned LDSC evaluates whether the per-SNP heritability is higher in the SNPs in an SNP list (in our study the SNPs within ±100 kb of the cell-type-specific genes) compared to the other SNPs.^40^ We calculated p values from one-sided *Z* score coefficient for tissue/cell-type-specific genes.

## Results

We curated summary statistics from 17 independent cohorts, a total of 723,266 individuals of European descent comprising 147,997 hearing loss cases (20.5%) and 575,269 controls (79.5%). Affected individuals were defined by either clinical diagnosis of hearing loss (ICD9 and 10; FinnGen and EGCUT, 37% of participants) or self-reported hearing impairment (all other cohorts, 63% of participants). We completed a genome-wide meta-analysis of 8,244,938 imputed SNPs that passed quality control (QC) and identified 48 significant loci (p < 5 × 10^−8^). There was no evidence of residual population stratification in the results of meta-analysis (λ_GC_ = 1.2764, λ_GC_ scaled to 1,000 cases and 1,000 controls = 1.001173). LD score regression intercept is 1.0039 (0.0095), indicating that the inflation of the GWAS test statistics was due to polygenicity (Figure S1). SNP heritability (h^2^) on the observed scale was h^2^ = 0.0252 (SE = 0.0013) and on the liability scale was estimated to be between 0.033 (SE = 0.002) and 0.061 (SE = 0.003) given the case/control ratio in our sample (20%) and populational prevalence in the range of 5%–40%. Next, we employed conditional and joint analysis (COJO) to identify lead independent signals in the loci (Table S3). Of 48 lead significant SNPs, 10 were considered novel associations (Table 1), defined as LD < 0.6. LDSC genetic correlations attributable to genome-wide SNPs (rg) were estimated across all hearing loss cohorts (Table S4). Regional locus zoom plots and forest plots of significant loci are presented in Figures S2 and S3, respectively.

**Table 1:** Summary statistics of significantly associated loci identified in the genome-wide association meta-analysis of AHRI

**SNP**	**Chr**	**Pos (hg19)**	**EA**	**OA**	**EAF**	**Beta**	**SE**	**p value**	**Direction**^a^	**Locus annotation**
rs4660885	1	46243756	a	G	0.4344	−0.007	9.00E−04	3.74E−12	--+--?----?-+----	IPP-[x]-MAST2
rs7525101	1	165109131	t	C	0.4424	0.006	9.00E−04	8.64E−11	++++-?++++?--++++	PBX1---[x]-LMX1A
rs6545432	2	54817683	a	G	0.5091	0.007	9.00E−04	2.36E−13	++++++?-++?++++++	[SPTBN1] intronic
rs741475	2	208087139	t	C	0.5771	−0.006	9.00E−04	4.02E−10	-----?-+--?------	KLF7-[x]--CREB1
rs3915060	3	121712980	t	C	0.7272	−0.006	1.00E−03	3.96E−09	+-+--?+-+-?+++---	[ILDR1] intronic
rs72622585^b^	3	181992315	t	C	0.8252	0.009	1.30E−03	3.41E−13	++-++--+++???-+++	SOX2---[x]---ATP11B
rs13148153	4	17517558	t	c	0.1342	0.010	1.40E−03	2.64E−12	+++++?++++???-+++	[CLRN2] intronic
rs323693	5	2562593	t	c	0.882	−0.010	1.40E−03	1.91E−12	--++-?--++?------	IRX4---[x]--IRX2
rs1981809	5	72920029	t	c	0.4526	−0.009	9.00E−04	1.36E−20	-----+?-+-?+-+---	UTP15-[x]-ARHGEF28
rs4413512^b^	5	73077349	a	g	0.5289	−0.010	9.00E−04	1.28E−25	-----?--+-?-+----	[ARHGEF28] intronic
rs13171669	5	148601243	a	g	0.5682	−0.006	9.00E−04	1.61E−11	-----?+++-?------	[ABLIM3] intronic
rs115596275	6	32420218	c	g	0.0213	0.024	3.50E−03	2.73E−12	?+???++-?+???++++	HLA-DRA-[x]-HLA-DRB5
rs7764856^b^	6	32680640	a	t	0.3435	0.007	1.00E−03	1.10E−10	?+-??+++?-???++++	HLA-DQB1-[x]-HLA-DQA2
rs4714678	6	43342591	a	g	0.4031	−0.009	9.00E−04	7.20E−20	+-++--+---?+-----	ZNF318-[x]-ABCC10
**rs9493627**	**6**	**133789728**	**a**	**g**	**0.3191**	**0.009**	**1.00E−03**	**9.56E−18**	**++++-+?+-+?-+++++**	**[EYA4] G>S**
rs2296508	6	158497717	t	c	0.4795	−0.006	9.00E−04	4.34E−10	--+++??-+-?++----	[SYNJ2] V>V
rs11238325	7	50853151	t	c	0.7315	0.007	1.00E−03	1.97E−11	++++-?++++?+-++++	[GRB10] intronic
rs4732339	7	138491839	a	g	0.5864	0.006	9.00E−04	6.10E−10	++++-?++-+?+-++++	TMEM213-[x]-KIAA1549
rs150903480	8	91376248	a	g	0.0114	−0.025	4.40E−03	2.70E−08	?-?+-+--+-?--+---	[LINC00534]
rs13268718	8	141687200	t	g	0.5072	−0.005	9.00E−04	7.47E−09	+++-++-++-?--++--	[PTK2] intronic
rs2393729	10	63837016	t	c	0.4218	−0.006	9.00E−04	3.07E−10	-++--??-+-?+++---	[ARID5B] intronic
**rs143282422**	**10**	**73377112**	**a**	**g**	**0.0112**	**0.032**	**4.60E−03**	**6.27E−12**	**?+?++?++++???++++**	**[CDH23] A>T**
rs1097215	10	94787804	a	g	0.4752	−0.005	9.00E−04	1.11E−08	-+-+----+-?--+---	[EXOC6] intronic
rs10901863	10	126812270	t	c	0.2683	0.011	1.10E−03	9.30E−23	++-++?++-+?++++++	[CTBP2] 5′ UTR
rs7939493	11	8073610	a	t	0.1911	−0.009	1.20E−03	2.47E−14	-++--?-+---??----	[TUB] intronic
rs141403654	11	47715487	a	t	0.9837	−0.022	3.90E−03	2.52E−08	+-?--?-+-------+-	[AGBL2] intronic
rs147893329^b^	11	57735006	c	g	0.0107	0.028	4.80E−03	8.17E−09	+??++?++--+++++++	CTNND1--[x]-OR9Q1
rs566673	11	66401373	t	g	0.5339	−0.005	9.00E−04	3.41E−08	--+-++?+---------	RBM14-[x]-RBM4
rs72963168	11	88943035	t	c	0.7254	−0.009	1.00E−03	3.73E−19	-++--?------+----	[TYR] intronic
rs67307131	11	118480223	t	c	0.654	−0.008	1.00E−03	4.62E−15	-+--+?---?-??+-?-	[PHLDB1] intronic
rs7313797^b^	12	109896165	t	c	0.5604	−0.006	9.00E−04	7.38E−12	++++++?++-?------	[KCTD10] intronic
**rs35887622**^b^	**13**	**20763620**	**a**	**g**	**0.9854**	**−0.022**	**3.90E−03**	**2.59E−08**	**?-?-+++++-?------**	**[GJB2] M>T**
rs920701	13	76417101	t	c	0.6357	−0.006	1.00E−03	5.06E−11	-+++--?-+-?------	[LMO7] intronic
rs9517282^b^	13	99059183	a	c	0.548	−0.005	9.00E−04	3.54E−08	-+-+-?----?-+----	[FARP1] intronic
rs1566128	14	52514981	a	g	0.4126	0.007	9.00E−04	1.42E−14	+--+++?+++?-+++++	[NID2] intronic
rs4132250	15	89229000	c	g	0.778	0.007	1.10E−03	3.18E−11	+++++?++-+?++++++	ISG20-[x]--ACAN
rs62033400	16	53811788	a	g	0.6044	0.005	9.00E−04	4.52E−08	---+++-+-++-+-+++	[FTO] intronic
rs11643684	16	55490167	t	g	0.2031	−0.007	1.10E−03	2.26E−09	--+--?------+----	IRX6--[x]-MMP2
rs13337678^b^	16	56379937	t	c	0.5711	−0.005	9.00E−04	3.72E−08	++-++??----+-----	[GNAO1] 3′ UTR
rs222835	17	7134129	a	g	0.4247	0.006	9.00E−04	4.81E−10	-++++??+++?-+++++	[DVL2] intronic
**rs143796236**	**17**	**79495969**	**t**	**c**	**0.0076**	**0.035**	**5.60E**−**03**	**2.73E**−**10**	**?+?++?+??-???++++**	**[FSCN2] H>Y**
rs11152089	18	52625943	t	c	0.2134	0.007	1.10E−03	9.24E−10	+-++++++++?++-+++	[CCDC68] 5′ UTR
rs11881070	19	2389140	t	c	0.2882	−0.006	1.00E−03	5.72E−09	----+??---?+-+---	SPPL2B-[x]-TMRPS9
**rs12980998**^b^	**19**	**4217510**	**a**	**t**	**0.8135**	**−0.007**	**1.20E**−**03**	**1.02E**−**07**	**+---+?-?+-???----**	**[ANKRD24] T>S**
**rs61734651**^b^	**20**	**61451332**	**t**	**c**	**0.0721**	**0.011**	**1.90E**−**03**	**8.16E**−**09**	**?+-+-+++++?++++++**	**[COL9A3] R>W**
**rs5756795**	**22**	**38122122**	**t**	**c**	**0.5419**	**−0.008**	**9.00E**−**04**	**3.65E**−**17**	**+-+++-----?++----**	**[TRIOBP] F>I**
rs132931	22	38487526	a	g	0.5869	−0.007	0.0009	1.59E−14	++---+?---?------	[BAIAP2L2] intronic
**rs36062310**	**22**	**50988105**	**a**	**G**	**0.0427**	**0.027**	**0.0023**	**4.25E**−**32**	**++++++---+?+-++++**	**[KLHDC7B] V>M**

48 loci significantly (p < 5 × 10^−8^) associated with hearing loss. Abbreviations: Chr, chromosome; Pos, genomic position (bp); EA, effect allele; OA, other allele; EAF, effect allele frequency; Beta, effect size for EA; SE, standard error of effect size. Missense SNPs are listed in bold, with corresponding amino acid change.

a Summary of effect direction for each study: + is risk increasing, - is risk decreasing, ? indicated the SNP was not present in sample cohort sequence: AGES, SA, FVG, RS2, RS3, DTR, HABC, FHS, RS1, EGC, SALT, STAGE, SALTY, TWINSUK, WGHS, FinnGen, UKBB. Locus annotation: single dash (-), <100 kb; double dash (--), 100–500 kb; triple dash (---), >500 kb.

b No previous association with hearing loss in a GWAS.

### Gene prioritization and pathway analysis

We used MAGMA v.1.08 and VEGAS2 for gene-set analysis and prioritization of genes at associated loci (Figure S4). Genes were examined for their relationship with hearing loss in human or mice. Seventeen loci were in or near genes with known associations to hearing loss (Table S5). Pathway analysis using VEGAS2 revealed strong enrichment in sensory perception of mechanical stimulus, sensory perception of sound, actin binding, and negative regulation of actin filament polymerization (Table S6). Interestingly, sensory perception pathways included *KCNQ4, OTOF, POU4F3, PDH15,* and *GRIN2B*, genes known to play a role in many different aspects of hearing function. Additional fine-mapping analysis identified credible sets of SNPs for each locus with 95% probability of being causal (total of 5,605 SNPs, Table S7).

### Missense SNPs

Eight SNPs encoded missense mutations (Table 2). The proportion of missense SNPs overall was 17%, which is significantly higher than the average of 5.4% found in other GWAS results (GWAS Catalog accessed 19^th^ October 2021; 1,107 studies with at least 10 genome-wide significant loci were included; Fisher exact test p = 0.005). Four of the identified genes have an established connection to deafness: *EYA4* (Deafness, autosomal dominant 10, DFNA10 [MIM: 601316]), *CDH23* (DFNB12 [MIM: 601543]), *GJB2* (DFNA3A [MIM 601544] and DFNB1A [MIM: 220290]), and *TRIOBP* (DFNB28 [MIM: 609823]).^43^ Another three are related to hearing loss in mice: *FSCN2,**^44^* *ANKRD24*, and *KLHDC7B* (https://www.mousephenotype.org/about-impc/). Mutations in *COL9A3* cause autosomal-recessive Stickler syndrome,^45^ a disorder affecting connective tissue (such as the spiral ligament) and commonly leading to hearing loss. With one exception (*ANKRD24* [CADD score = 0.241]), CADD scores of the missense SNPs (18.45 to 31) were among the top ∼1%–0.1% of deleterious variants in the human genome. We conducted an additional *in silico* functional analysis on these 8 missense variants and the results provide strong evidence that variants in *FSCN2* and *COL9A3* are highly deleterious and likely impact gene function (Table S8). High-resolution protein structures were only available for *GJB2*; however, the utilized algorithms were not unanimous regarding the functional consequences of the identified variant *GJB2* p.Met34Thr (rs35887622) (Figure S5A). The GJB2 transporter, also named connexin-26 (Cx26), is a hexamer with the altered amino acid being located at the core of the channel (Figure S5A). The amino acid exchange results in a substitution of hydrophilic arginine for hydrophobic methionine, which alters the surface energy of the channel and likely affects substrate translocation (Figure S5B).

**Table 2:** Missense SNPs in genes associated with hearing loss

**SNP**	**rs9493627**	**rs143282422**	**rs35887622**	**rs143796236**	**rs12980998**	**rs61734651**	**rs5756795**	**rs36062310**
**Gene**	***EYA4***	***CDH23***	***GJB2***	***FSCN2***	***ANKRD24***	***COL9A3***	***TRIOBP***	***KLHDC7B***
**SNP characteristics**								

Chr	6	10	13	17	19	20	22	22
Pos (hg19)	133789728	73377112	20763620	79495969	4217510	61451332	38122122	50988105
Locus	*DFNA10*	*DFNB12*	*DFNA3A* *DFNB1A*	–	–	–	*DFNB28*	–
Alleles (major>minor)	G>A	G>A	A>G	C>T	A>T	C>T	T>C	G>A
MAF	0.319	0.011	0.015	0.008	0.187	0.072	0.458	0.043
AA change	p.Gly277Ser	p.Ala366Thr	p.Met34Thr	p.His138Tyr	p.Thr785Ser	p.Arg103Trp	p.Phe1187Leu	p.Val1145Met
Pathogenicity score	0.29	0.43	0.71	0	1	0	1	0.71
Phenotype	hearing loss, AD	hearing loss, AR/Usher syndrome	hearing loss, AD and AR	hearing loss in mice^44^	abnormal ABR in mice^23^	Stickler syndrome, AR	hearing loss, AR	abnormal ABR in mice^23^

**Gene characteristics**								

Transcript	NM_004100.5	NM_022124.6	NM_00400.6	NM_001077182.3	NM_133475.1	NM_001853.4	NM_001039141.3	–
Gene length (bp)	5,699	10,085	2,250	1,665	4,026	2,485	10,129	2,990
Translation length	639	3,359	226	492	1,146	684	2,365	594
Number of exons	20	70	2	5	22	32	24	1
Total variants gnomAD v2.1.1	1,081	6,088	345	836	1,793	2,251	3,281	609
All ≥1% (total)	14	84	4	1	48	54	38	7
MAF ≥1% in EUR	10	60	1	6	36	53	38	7
Total unique gnomAD SNPs in same exon as GWAS SNP	0	1	0	1	6	1	14	6

**Pathogenicity**								

DM	52	353	270	0	0	9	49	0
DM?	8	67	77	0	0	1	14	1
SUM	60	420	347	0	0	10	63	1
Exon containing SNP	11	11	2	1	18	5	7	1

Abbreviations: Chr, chromosome; Pos, genomic position; MAF, minor allele frequency in the current study; AA change, amino acid change; AD, autosomal dominant; AR, autosomal recessive; bp, base pair; EUR, European (non-Finnish); MAF, minor allele frequency; SNP, single-nucleotide polymorphism; DM, disease causing mutation. Pathogenicity score is estimated from an aggregated score detailed in Table S6. Aggregated pathogenicity score is normalized from 0 (variant predicted to be deleterious) to 1 (predicted to be benign). Phenotype: in humans, except where noted otherwise. DM?, likely disease causing mutation based on the Human Gene Mutation Database (HGMD) Professional version 2021.3.^46^

### Genetic correlations

A significant positive genetic correlation was found between hearing loss and insomnia, depressive symptoms, neuroticism, and obesity (LD Hub, Figure S6, Table S9). A significant negative genetic correlation was found with subjective well-being. No significant correlation was established between hearing loss and several neurological disorders or medical conditions. Additionally, we implemented the same approach to investigate SNP-based correlation between hearing loss and other traits using recent results from the Psychiatric Genomics Consortium and found significant genetic correlation with major depressive disorder, autism spectrum disorder, alcohol dependence, neuroticism, attention deficit hyperactivity disorder, and smoking initiation/smoking (Figure S7, Table S10).

### GTEx tissue enrichment analysis and cell-type specificity of hearing loss genetic associations

**Figure 2:**
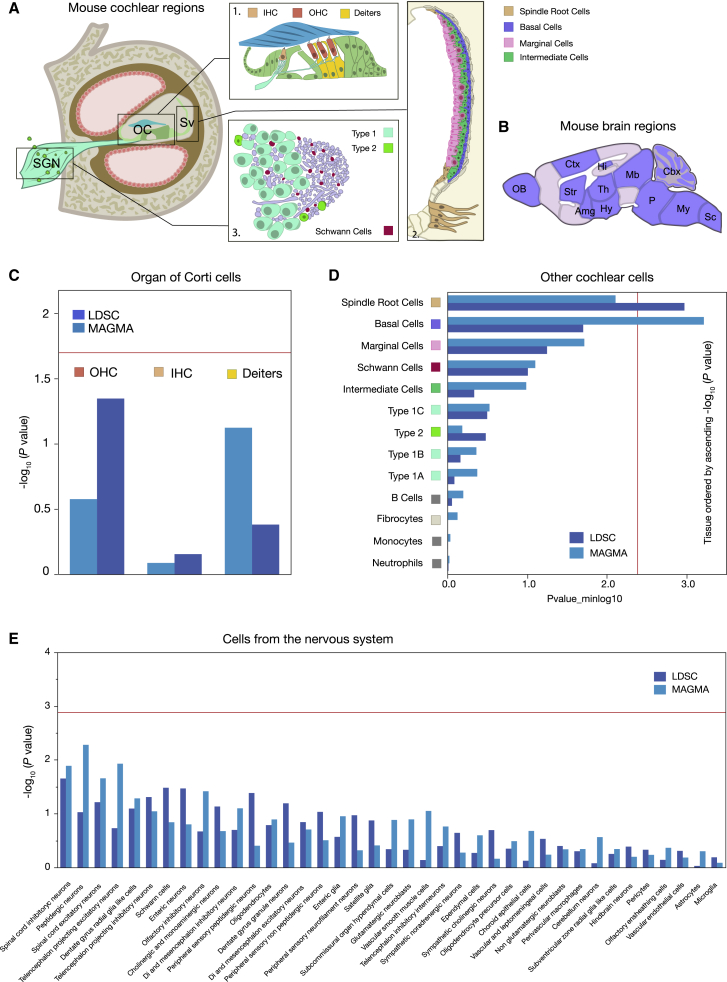
Evaluation of enrichment of common-variant hearing loss GWAS results in scRNA-seq mouse datasets Schematic of the mouse cochlea (A) and the mouse brain (B) regions used for the enrichment analysis. Abbreviations: Amg, amygdala; Cbx, cerebellum; Ctx, cerebral cortex; DC, Deiters’ cells; Hi, hippocampus; Hy, hypothalamus; IHC, inner hair cells; Mb, midbrain; My, medulla; OB, olfactory bulb; OC, organ of Corti; OHC, outer hair cells; P, pons; Sc, spinal cord; Str, striatum; Th, thalamus. OC is magnified in box 1 and illustrates the IHC, OHC, and DC, whose enrichment is shown in (C). A color box links a specific cell to the schematic. The red line is the Bonferroni significance threshold (−log_10_ p value 1.77). The enrichment analysis using cells from the stria vascularis (box 2) and the spiral ganglion neuron region (box 3) reveals a significant enrichment for spindle root cells and basal cells (D). All type 1 spiral ganglion neurons (type 1a, b, c) were all labeled the same color for sake of clarity. Given the broad and scarce distribution of immune cells (monocytes, neutrophils, and B cells), these are not shown on the schematic. The red line shows the Bonferroni significance threshold (−log_10_ p value 2.42) (E). Mouse nervous system cell type enrichment showing no significant enrichment. The red line shows the Bonferroni significance threshold (−log_10_ p value 2.89). Images from (A) and (B) were reproduced from previous work^47^, ^48^, ^49^ with permission from Nature Springer.

We next attempted to identify the specific somatic tissues implicated by our GWAS and define a spatial topography of hearing loss heritability-associated gene expression. Expression datasets for multiple human tissues GTEx v.8 do not contain inner ear tissue and, unsurprisingly, LDSC and MAGMA revealed no significant enrichment of SNP-h^2^ in GTEx tissues (Figure S8; Table S11). We relied on mouse cochlear^37^ and brain^39^ cell-specific expression profiles to determine cell types matching the common variants identified. This approach enabled us to prioritize cells that are fundamental to the etiology of hearing loss. Mouse cochlea scRNA-seq originated from Milon et al.,^37^ which included the spiral ganglion region and the stria vascularis (total of 36,616 cells; Figure 2A, Table S2), but since this dataset did not include scRNA-seq data from cells of the organ of Corti (that harbor hair cells and Deiters’ cells), we included an additional dataset from Ranum et al. containing Deiters’ cells and inner and outer hair cells from the mouse cochleae^38^ (total of 3,189 cells; Figure 2A; Table S2). Since these two cochlea studies used different methods, these were analyzed separately. Nervous system scRNA-seq included 39 broad cell types from 19 regions of the mouse central, peripheral, and enteric nervous system (total of 160,796 cells; Figure 2B).^39^

If hearing loss is associated with a particular cell type, we would expect more of the genome-wide association signal to be concentrated in genes with greater specificity for that cell type. To show evidence connecting hearing loss GWASs to cell type, we used two different methods accounting for gene size and linkage disequilibrium: LDSC,^40^ assessing the enrichment of the common SNP heritability of hearing loss in the most cell-type-specific genes and MAGMA,^20^ evaluating whether gene-level genetic association with hearing loss linearly increases with cell-type expression specificity. We found no enrichment in cells from the organ of Corti (Deiters’ cells, inner and outer hair cells; Figure 2C, Table S12). Arguably, the lack of results in this enrichment analysis could be due to the fact that specificity is a relative measure between these three cell types. This assumes that the “effective” genes have similar expression pattern in all three cell types and therefore would not be captured by the specificity measure. However, this reasoning is not supported by current findings since Deiters’ cells and hair cells differ considerably in gene expression signature.^38^ When assessing the enrichment in SGN and cells from the cochlear lateral wall (stria vascularis), LDSC analysis revealed the involvement of spindle cells of the stria vascularis and root cells of the outer sulcus, whereas MAGMA analysis highlighted the involvement of basal cells of the stria vascularis in hearing loss (Figure 2D, Table S13). Here, spindle and root cells could not be distinguished molecularly one from another, which is why they were labeled “spindle root cells.” In contrast, no enrichment was found in any cell type from the mouse nervous system (Figure 2E, Table S14). These findings strongly support a prominent role of the stria vascularis in hearing loss.

To further gain insights into the potential molecular mechanisms involved in strial dysfunction, we investigated the top 10% specifically expressed genes in basal (342 genes, Table S15) and spindle root (380 genes, Table S16) cells. In basal cells, 10 genes were associated with SNPs that were GWAS significant, but none of them were found in the significant pathways associated with hearing loss and listed in Table S6. Among these genes, the evidence for an involvement in hearing loss is sparse. For instance, *NID2*, which has been associated in humans with the Landau-Kleffner syndrome, a rare language disorder with suspicions of hearing loss.^50^ *PC* encodes a pyruvate carboxylase that requires biotin and ATP for catalyzing gluconeogenesis. Pyruvate carboxylase deficiency is a rare severe metabolic disease that in some cases can be manifested with hearing loss.^51^ *CCS* encodes a copper ion binding protein and its mutations are associated with disfunctions of copper metabolism resulting in Wilson disease, a rare inherited disorder that causes excess accumulation of copper in several organs.^52^ Individuals with Wilson disease display abnormal auditory brainstem responses.^53^ Similarly, *AHDC1* is most probably involved in DNA binding, and loss-of-function mutations result in Xia-Gibbs syndrome—a neurodevelopmental disorder with rare presentation of hearing loss.^54^ In spindle root cells, *EYA4* and *HOMER2* were identified in pathways that were found significant in VEGAS2—sensory perception of sound and sensory perception of mechanical stimulus, respectively. The contribution of *EYA4* variants to hearing loss has been well established.^55^ HOMER2 is involved in intracellular homeostasis of calcium and cytoskeletal organization and has been previously associated with hearing loss,^56^ but its function within the stria vascularis remains unknown. *TMPRSS9* encodes a membrane-bound serine polyprotease involved in the proliferation of inner ear progenitor cells in the mouse cochlea.^57^ *GAS2* encodes an actin filament binding protein that plays a role in cell shape and regulating microfilament rearrangements. In mice, *Gas2* is expressed in supporting cells but also in the stria vascularis from the post-natal cochlea and its disruption causes hearing loss in mice and human.^58^ Taken together, these findings suggest that dysfunctions in the stria vascularis involve a large range of molecular mechanisms, globally impacting strial function.

## Discussion

The present genome-wide meta-analysis is among the largest conducted in hearing genetics to date and provides an association catalog that helps to refine the fundamental basis of hearing loss. We find evidence of association for 48 common genetic loci, of which 10 were novel and highlight the role of genes expressed in cochlear lateral wall, consisting of the spiral ligament and stria vascularis as an important contributor to hearing loss. We employed a pragmatic, clinically informed approach by including cohorts that met empirical criteria for sufficient genetic and phenotypic similarity, based on both self-report and medical registries. We previously verified a high genetic correlation between objective measures of hearing loss and questionnaires.^14^ Second, our findings point to multiple genes that have been reported to cause Mendelian forms of hearing loss previously, including *EYA4, CDH23, TRIOBP*, and *GJB2*, the latter being the most commonly reported gene in autosomal-recessive non-syndromic hearing loss.^59^ This study is part of an expanding number of hearing loss GWASs with ever increasing sample size and the emerging functional and cellular bioinformatics tools enable us to unravel more of its pathophysiological pathways. Our study confirms as do other recent GWASs that hearing loss is driven by multiple common variants in known hearing genes.

Our results allow us to draw several broad conclusions. Of importance, a large proportion of potentially disruptive missense variants were found in contrast to other disease-related GWASs (Fischer’s exact test; p = 0.005; Figure S9), suggesting that a burden of common and rare yet impactful variants may drive the risk of hearing loss.

Second, our results do not point toward a large involvement of the brain within hearing loss. Although this is not entirely unexpected, proficient hearing acuity requires the functional integration of signals that are captured at the level of the cochlea, which are transduced to provide signal down the VIII^th^ cranial nerve and further propagate via the brainstem toward the thalamus and the auditory cortex. Abnormal CNS streaming of signal to noise has been implicated in ARHI^60^ and is supported by the association with cognitive decline, but our GTEx and scRNA-seq analyses revealed no enrichment of GWA signals in the brain, nor in its regions or its major cell types.

Third, we found significant positive genetic correlations with depressive symptoms, obesity, and smoking, but not with Alzheimer disease. The latter is interesting as hearing impairment is an established risk factor for cognitive decline and dementia,^61^ suggesting that hearing impairment rather than shared underlying genetic factors contribute to the development of dementia. A more complete analysis of the drivers of this relationship is needed, but the inference is that shared environmental factors contributing to hearing loss and dementia will predominate, rather than shared genetic factors.

Fourth, our findings in hearing loss pathway analyses implicate the processes involved in cytoskeleton organization and actin binding, two broad features of the mechano-transduction apparatus of the sensory hair cells.^62^ Indeed, these findings are consistent with a recent GWAS performed on the UKBB that localized a number of lead SNPs in cells from the post-natal mouse cochleae using scRNA-seq data^12^ or in the human cochlea (mainly in type I SGN, or hair cells) using immunohistochemistry on samples collected from individuals with life-threatening posterior cranial fossa meningioma compressing the brain stem.^63^ These included *EYA4, LMX1A, PTK2/FAK, UBE3B, MMP2, SYNJ2, GRM5, TRIOBP, LMO-7,* and *NOX4*. Consistent with their findings, we also identified *SPTBN1*, a mouse ortholog Spectrin expressed in the cuticular plate at the base of the stereocilia, deletion of which causes profound deafness.^64^ Interestingly, synaptic plasticity genes were also found such *CTBP2*, which is an important marker of the pre-synaptic machinery—namely the synaptic ribbon—gathering the glutamate-filled vesicles prior to their release. Alterations in ribbon abundance has been associated with cochlear synaptopathy in mouse models of noise-induced hearing loss.^65^, ^66^, ^67^, ^68^ In these models a decrease in ribbon abundance in the absence of hearing loss (the so-called hidden hearing loss) is thought to be associated with problems in speech in noise recognition and tinnitus.^69^^,^^70^

Fifth, although lead association SNPs are related to sensory hair cell function and their involvement in hearing loss is well established, our cell-specific enrichment analysis revealed hearing loss being also driven, at least in part, by basal cells and spindle cells in the stria vascularis and root cells in the outer sulcus. Indeed, the lead SNPs mainly related to hair cell and auditory neuron function represent the tip of the iceberg (e.g., *STBPN1, CLRN2, EYA4, SYNJ2, CDH23, CTBP2, LMO7, FSCN2, ANKRD24, TRIOBP,* and *BAIAP2L2*) influencing hearing loss with the greatest probability, while the whole iceberg is pictured by the contribution of all GWAS signals, pointing to genes expressed in cells from the lateral wall, namely basal cells and spindle cells of the stria vascularis and root cells in the outer sulcus (e.g., *EYA4, MMP2, GJB2*, and *GJB6*)*.* These three cell types are primarily involved in endolymph ion homeostasis.^47^^,^^71^, ^72^, ^73^ The basal cells are coupled to each other by tight junctions to prevent leakage of ions^74^ and to keep the stria vascularis separate from the spiral ligament. In addition, the spindle cells have recently been shown through gene regulatory networks to have a role in responses to inflammation.^47^ However, we were unable to differentiate the spindle cells from the root cells unlike Gu et al.^47^ who used single-nucleus RNA-seq and could identify a differential expression between these two cell types.

Our findings are in opposition to a recent study suggesting that outer and inner hair cell loss is the main contributor to ARHI and that strial tissue loss does not correlate with audiologic patterns of ARHI.^75^ The human otopathologic analysis of this study focused on cellular loss of the stria vascularis and may have captured loss of basal cells that cover the full extension of stria. However, cellular loss of the other two cell types is not covered by this analysis. The spindle cells reside at the edges of the stria and the root cells are outside of the stria in the outer sulcus region. Furthermore, genetically caused functional loss cannot be recognized at the histopathologic level and may precede cellular loss. The precise contribution of the sensory cell and stria vascularis mechanisms to the development of hearing loss needs to be further elucidated using molecular techniques and at a time before cell death is apparent.

The lack of cochlear tissue in the GTEx biobank is indeed a major limitation to all genetic studies of hearing and communication disorders that needs to be addressed. Having access to eQTL data from human cochlear tissue would also provide significant advances in understanding the biology of hearing loss. These limitations were partially addressed in our study by gathering a unique combination of datasets of mouse cochlear scRNA-seq. Given the complexity of the organ and its ossification, the number of compartments, and the variety of constituent cell types, such comprehensive knowledge was not available until recently. Using expression data from rodents to infer on human auditory physiology may be seen as a limitation, since mouse expression data are not fully representative of human cells. However, from a total of 48 loci we identified, 18 harbor genes that have been associated with hearing loss. From these 18 loci, 16 are related to genes disruption of which causes hearing loss in mice. Thus, these findings strongly argue in favor of the translational reliability of the present findings.

We also note that part of the scRNA-seq mouse data used here was generated from 10X Genomics, which has a sequencing resolution that may have yielded insufficient number of genes to reveal enrichments (e.g., when compared to new methodologies such as Smart-Seq2). For instance, using 1,100 proprioceptive neurons and Smart-seq2, Wu et al. detected 11,000 genes per cell and identified 8 cell types,^76^ while previous studies using 10X Genomics favoring a higher number of cells but with lower coverage could not differentiate proprioceptive neurons in any subtypes.^77^ In the study from Milon et al., only one type of fibrocyte was identified, whereas there are 5 known types of fibrocytes (type I–V) present in both man and experimental animals, but that have quite different function and molecular expression.^78^^,^^79^ Thus, new sequencing technologies may offer increased resolution and statistical power to reveal more accurate predictions of the involvement of more specific cells in hearing loss.

Hearing loss is a heterogeneous disorder with many contributing factors during life. A potential implication for future genetic studies is the elucidation of the bulk of common variants using a cost-effective shortcut involving on-line self-reports combined with auditory tests, such as automated speech and noise test.^80^ The use of online assessment would allow for a comprehensive recording of phenotypes and environmental exposures in millions of individuals. Conversely, current clinical audiologic phenotyping is mostly limited to pure tone thresholds and by far not exhaustive. Carefully phenotyped individuals with hearing loss in combination with next-generation sequencing may increase the resolution of the genetic coverage and reduce the sample size to thousands, something that has been shown highly effective in the context of schizophrenia.^81^

### Conclusions

This study of hearing loss identified 48 associated loci, including 10 novel associations and 8 missense SNPs. Our work highlights the role of the cochlear lateral wall including the stria vascularis and the outer sulcus as a contributor to hearing loss. The results provide a valuable resource for the selection of promising genes for further functional validation in pre-clinical models and define targets for screening purposes, drug development, gene therapy, or stratification approaches. We believe such experiments will serve as a solid foundation for ultimately improving therapies against hearing loss.

## Consortia

The Estonian Biobank Research Team is composed of Andres Metspalu, Mari Nelis, Reedik Mägi, and Tõnu Esko.

## Data Availability

The GWAS summary statistics are deposited and available in Zenodo (https://zenodo.org/record/5769707#.Ybm6v33MKhx) and codes are available on GitHub (https://github.com/translational-audiology-lab/GWAS_ARHL).

## Supplemental information

Data S1. Tables S1–S16

Document S2. Article plus supplemental information
