# Supplementary material for: Genome-wide association meta-analysis identifies 48 risk variants and highlights the role of the stria vascularis in hearing loss
Source: Am J Hum Genet. 2022 May 16;109(6):1077–91. doi: 10.1016/j.ajhg.2022.04.010 (PMC9247887; doi:10.1016/j.ajhg.2022.04.010)
Supplement: Document S1. Supplemental methods and Figures S1–S9 [file mmc1.pdf]

The American Journal of Human Genetics, Volume *109*

Supplemental information

Genome-wide association meta-analysis identifies 48 risk variants and highlights the role of the stria vascularis in hearing loss

Natalia Trpchevska, Maxim B. Freidin, Linda Broer, Berthe C. Oosterloo, Shuyang Yao, Yitian Zhou, Barbara Vona, Charles Bishop, Argyro Bizaki-Vallaskangas, Barbara Canlon, Fabio Castellana, Daniel I. Chasman, Stacey Cherny, Kaare Christensen, Maria Pina Concas, Adolfo Correa, Ran Elkon, Estonian Biobank Research Team, Jonas Mengel-From, Yan Gao, Anne B.S. Giersch, Giorgia Girotto, Alexander Gudjonsson, Vilmundur Gudnason, Nancy L. Heard-Costa, Ronna Hertzano, Jacob v.B. Hjelmborg, Jens Hjerling-Leffler, Howard J. Hoffman, Jaakko Kaprio, Johannes Kettunen, Kristi Krebs, Anna K. Kähler, Francois Lallemend, Lenore J. Launer, I-Min Lee, Hampton Leonard, Chuan-Ming Li, Hubert Lowenheim, Patrik K.E. Magnusson, Joyce van Meurs, Lili Milani, Cynthia C. Morton, Antti Mäkitie, Mike A. Nalls, Giuseppe Giovanni Nardone, Marianne Nygaard, Teemu Palviainen, Sheila Pratt, Nicola Quaranta, Joel Rämö, Elmo Saarentaus, Rodolfo Sardone, Claudia L. Satizabal, John M. Schweinfurth, Sudha Seshadri, Eric Shiroma, Eldad Shulman, Eleanor Simonsick, Christopher Spankovich, Anke Tropitzsch, Volker M. Lauschke, Patrick F. Sullivan, Andre Goedegebure, Christopher R. Cederroth, Frances M.K. Williams, and Andries Paul Nagtegaal

Supplementary information

Figure S1. Quantile-Quantile plot of association test results from meta-analysis of hearing impairment

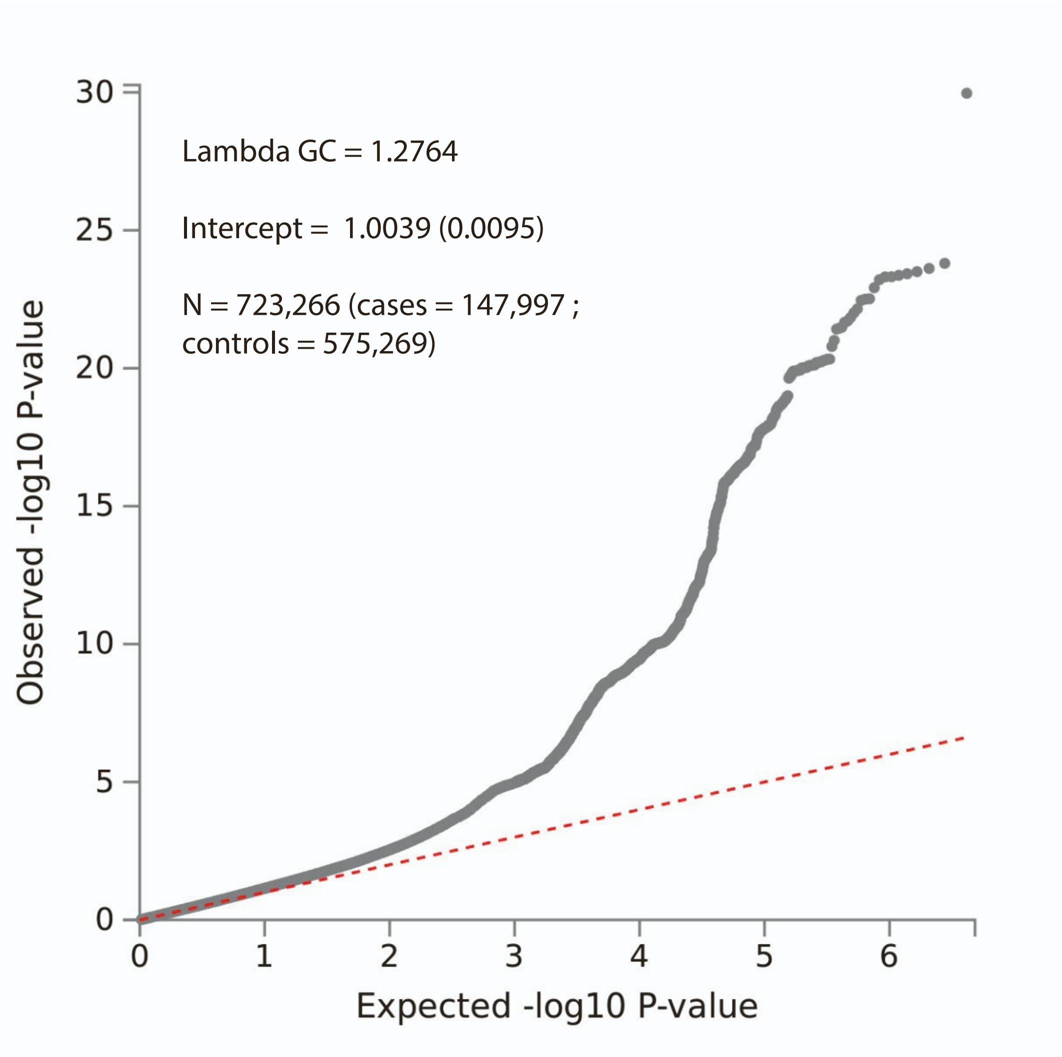

Figure S2 Locus Zoom plot of each statistically significant and independent SNP

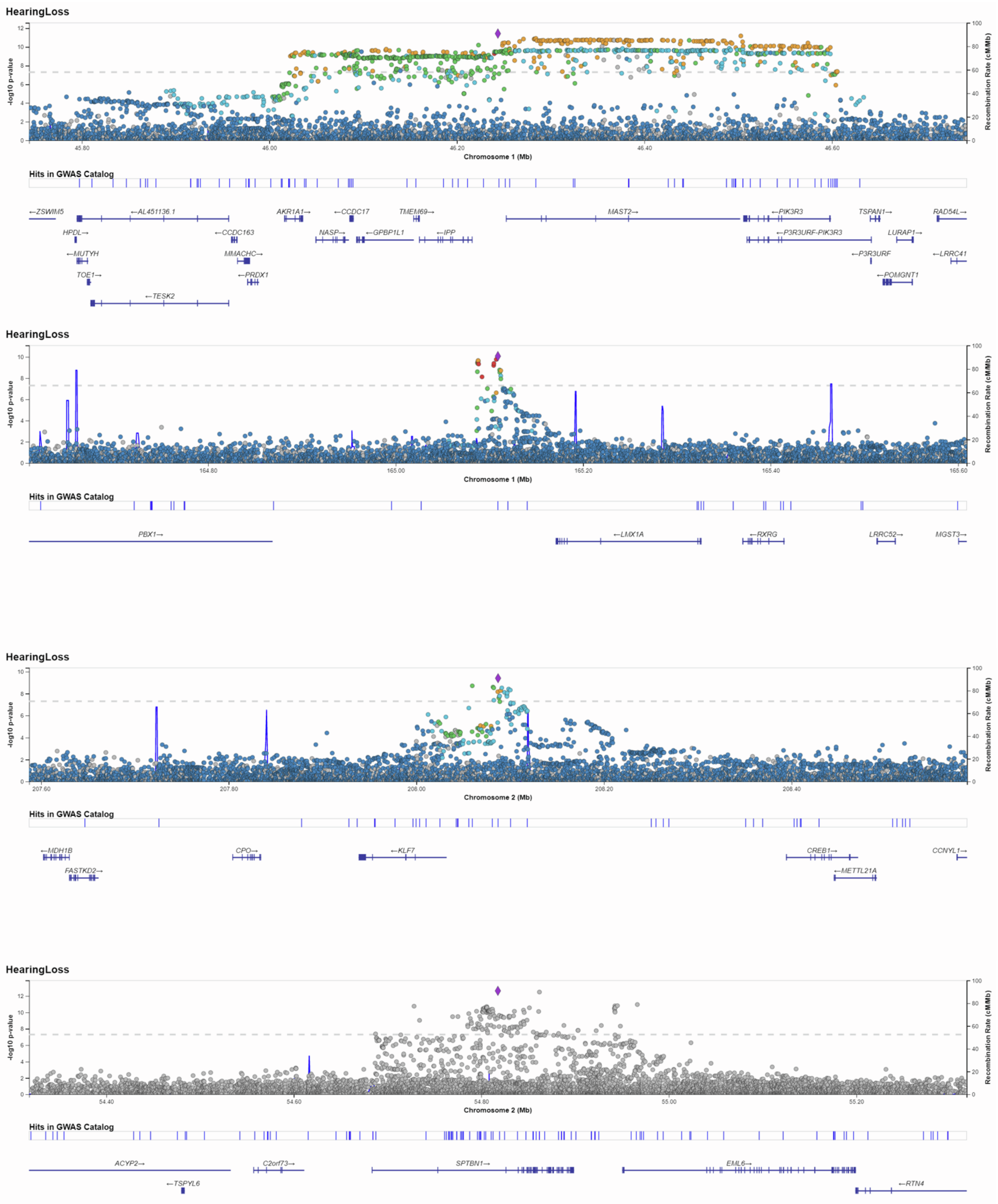

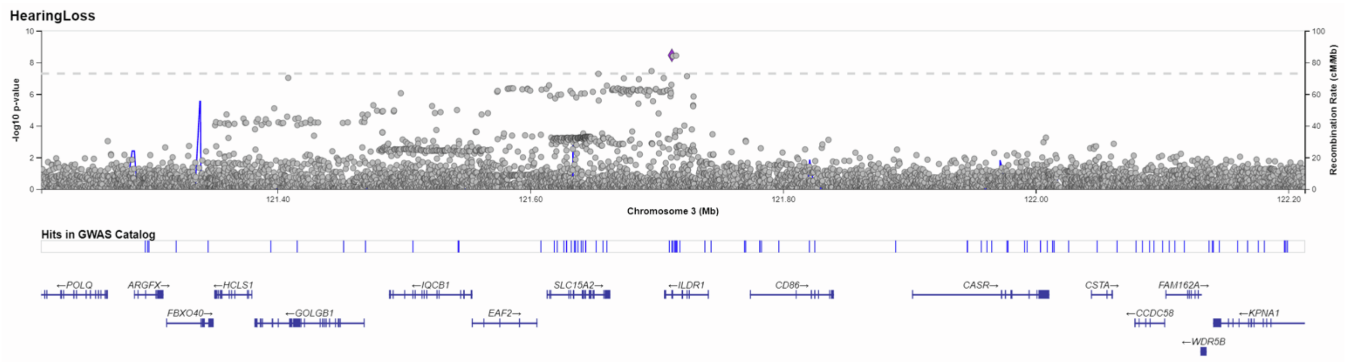
HearingLoss
-log10 p-value
Recombination Rate (cM/Mb)
Chromosome 3 (Mb)
Hits in GWAS Catalog
←POLQ
ARGFX→
←HCLS1
←IQCB1
SLC15A2→
←ILDR1
CD86→
CASR→
CSTA→
FAM162A→
FBXO40→
←GOLGB1
EAF2→
←CCDC58
←KPNA1
←WDR5B

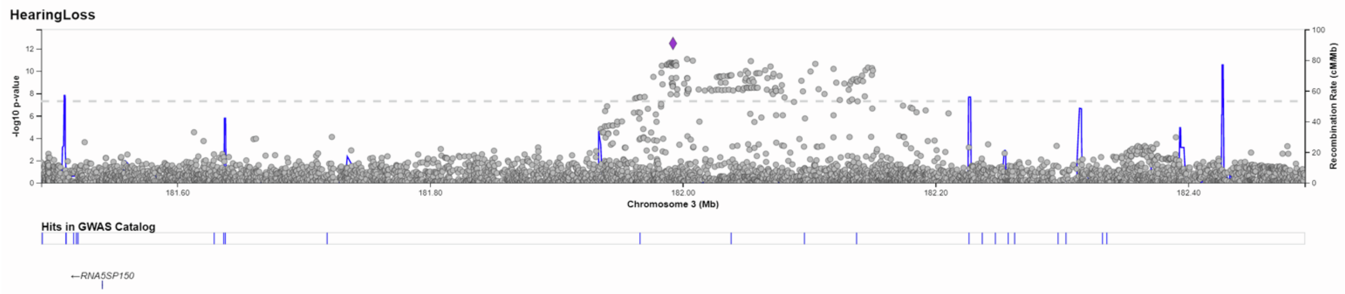
HearingLoss
-log10 p-value
Recombination Rate (cM/Mb)
Chromosome 3 (Mb)
Hits in GWAS Catalog
←RNA5SP150

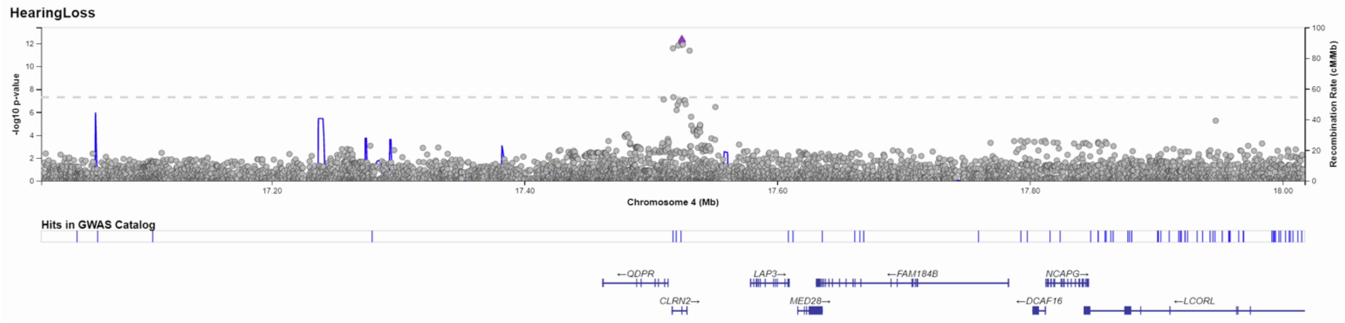
HearingLoss
-log10 p-value
Recombination Rate (cM/Mb)
Chromosome 4 (Mb)
Hits in GWAS Catalog
←QDPR
LAP3→
←FAM184B
NCAPG→
CLRN2→
MED28→
←DCAF16
←LCORL

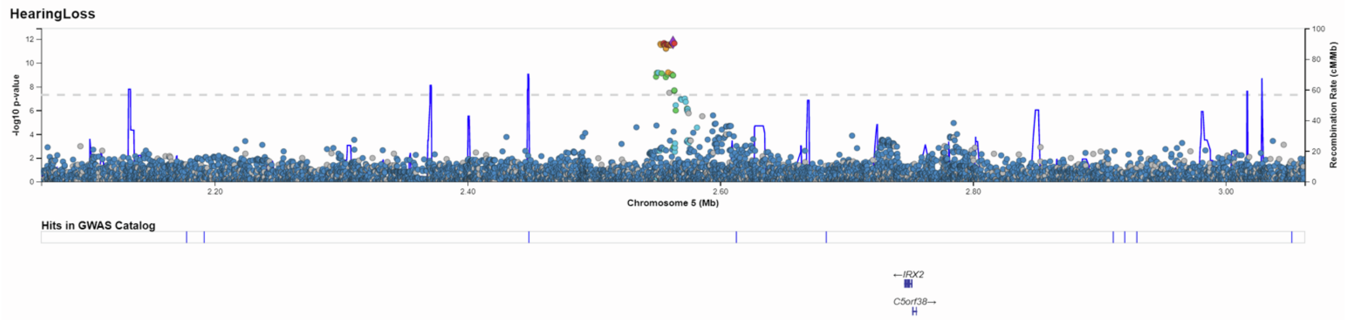
HearingLoss
-log10 p-value
Recombination Rate (cM/Mb)
Chromosome 5 (Mb)
Hits in GWAS Catalog
←IRX2
C5orf38→

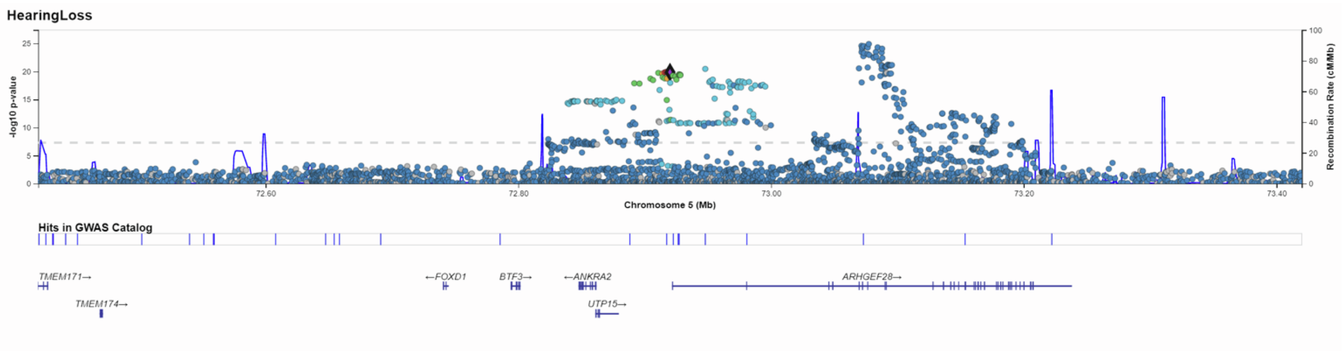
HearingLoss
-log10 p-value
Recombination Rate (cM/Mb)
Chromosome 5 (Mb)
Hits in GWAS Catalog
TMEM171→
TMEM174→
←FOXD1
BTF3→
←ANKRA2
UTP15→
ARHGEF28→

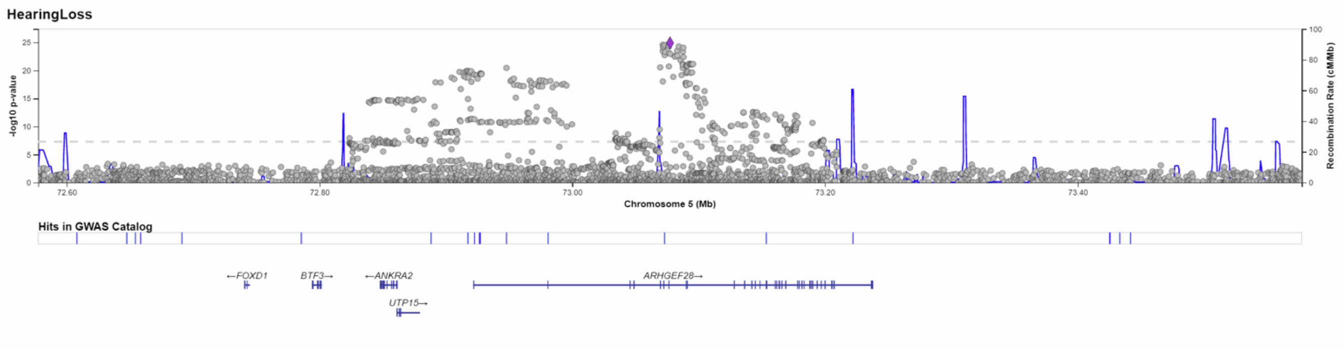
HearingLoss
-log10 p-value
Recombination Rate (cM/Mb)
Chromosome 5 (Mb)
Hits in GWAS Catalog
←FOXD1
BTF3→
←ANKRA2
UTP15→
ARHGEF28→

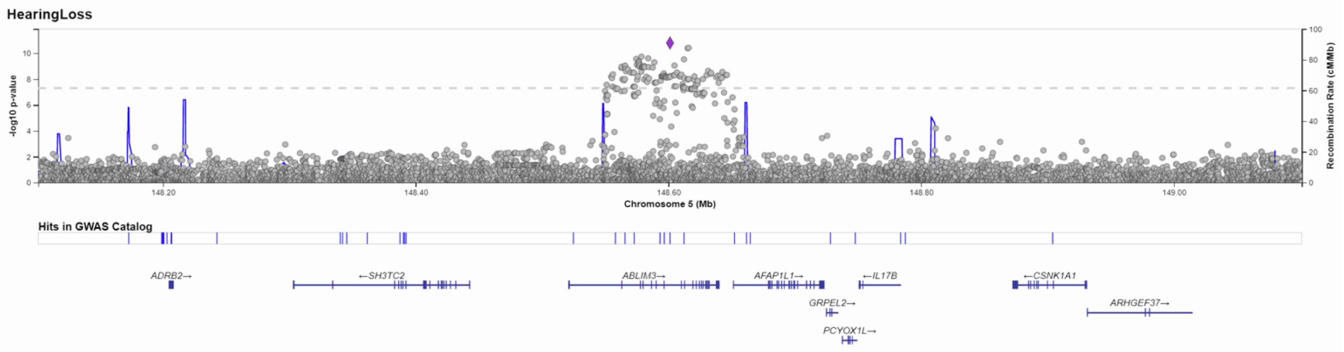
HearingLoss
-log10 p-value
Recombination Rate (cM/Mb)
Chromosome 5 (Mb)
Hits in GWAS Catalog
ADRB2→
←SH3TC2
ABLIM3←
AFAP1L1→
←IL17B
←CSNK1A1
GRPEL2→
PCYOX1L→
ARHGEF37→

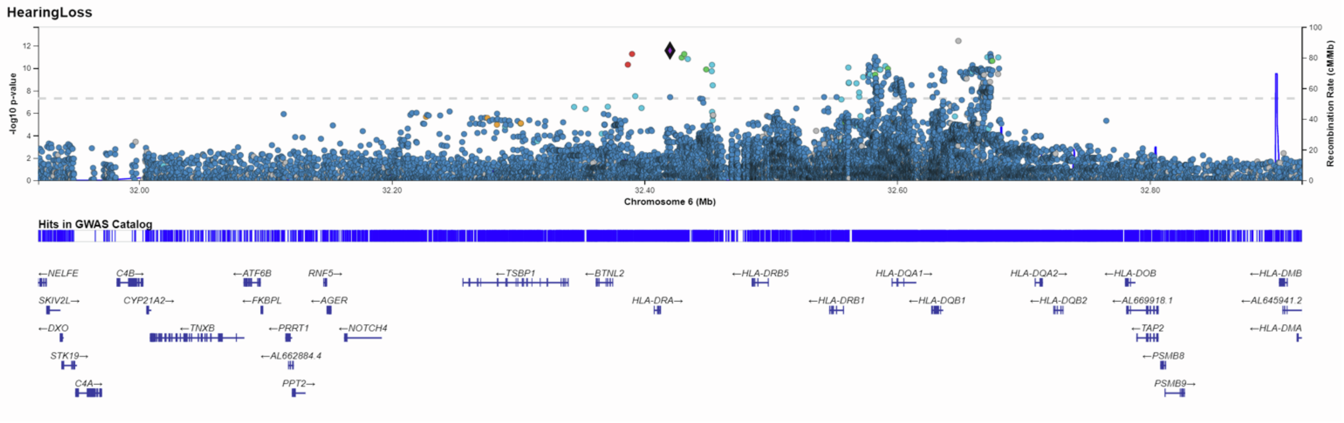
HearingLoss
-log10 p-value
Recombination Rate (cM/Mb)
Chromosome 6 (Mb)
Hits in GWAS Catalog
←NELFE
C4B→
←ATF6B
RNF5→
←TSBP1
←BTNL2
←HLA-DRB5
HLA-DQA1→
HLA-DQA2→
←HLA-DOB
←HLA-DMB
SKIV2L→
CYP21A2→
←FKBPL
←AGER
HLA-DRA→
←HLA-DRB1
←HLA-DQB1
←HLA-DQB2
←AL669918.1
←AL645941.2
←DXO
←TNXB
←PRRT1
←NOTCH4
←TAP2
←HLA-DMA
STK19→
←AL662884.4
←PSMB8
C4A→
PPT2→
PSMB9→

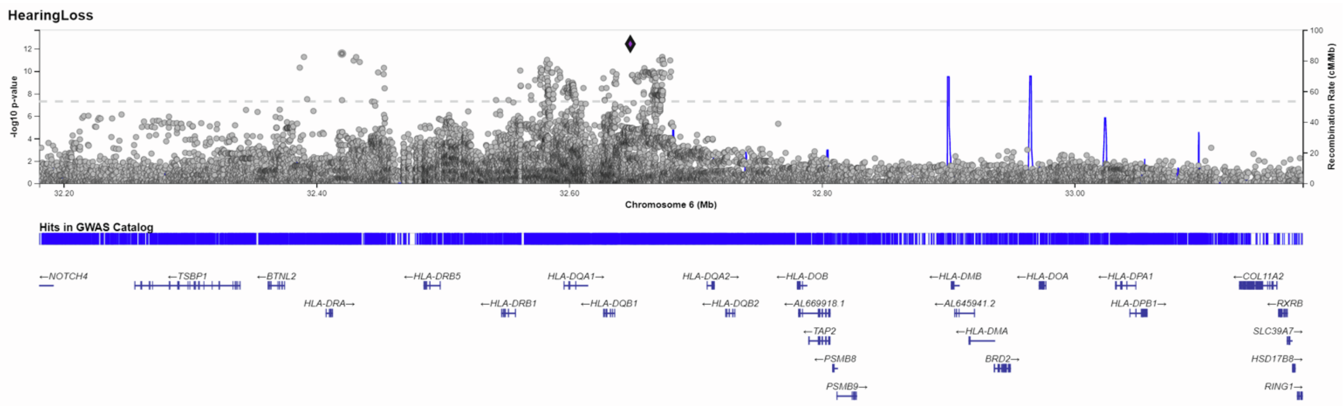
HearingLoss
-log10 p-value
Recombination Rate (cM/Mb)
Chromosome 6 (Mb)
Hits in GWAS Catalog
←NOTCH4
←TSBP1
←BTNL2
HLA-DRA→
←HLA-DRB5
←HLA-DRB1
HLA-DQA1→
←HLA-DQB1
HLA-DQA2→
←HLA-DQB2
←HLA-DOB
←AL669918.1
←TAP2
←PSMB8
PSMB9→
←HLA-DMB
←AL645941.2
←HLA-DMA
BRD2→
←HLA-DOA
←HLA-DPA1
HLA-DPB1→
←COL11A2
←RXRB
SLC39A7→
HSD17B8→
RING1→

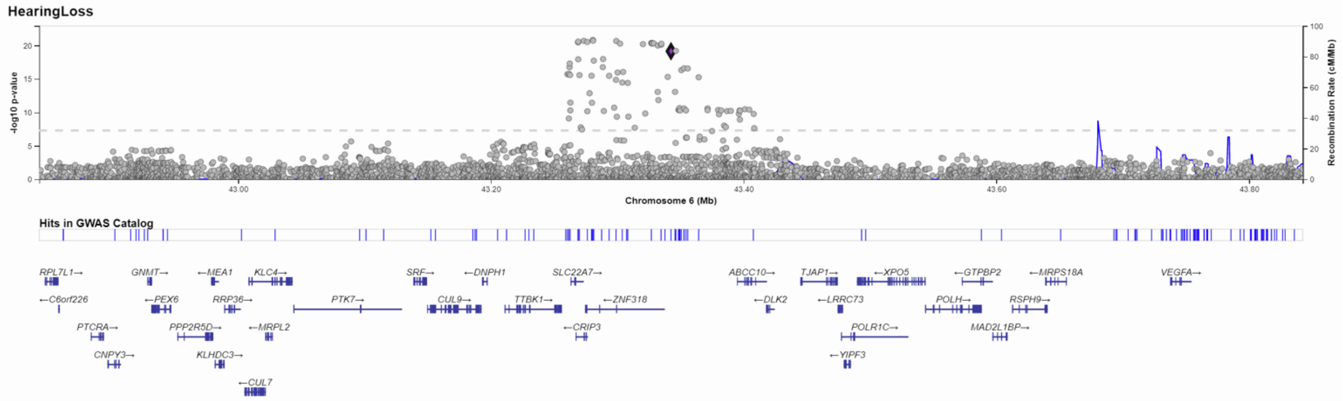
HearingLoss
-log10 p-value
Recombination Rate (cM/Mb)
Chromosome 6 (Mb)
Hits in GWAS Catalog
RPL7L1→
←C6orf226
PTCRA→
CNPY3→
GNMT→
←PEX6
PPP2R5D→
←MEA1
RRP36→
KLHDC3→
KLC4→
←MRPL2
←CUL7
PTK7→
SRF→
CUL9→
←DNPH1
TTBK1→
SLC22A7→
←CRIP3
←ZNF318
ABCC10→
←DLK2
TJAP1→
←LRRC73
POLR1C→
←YIPF3
←XPO5
POLH→
←GTPBP2
MAD2L1BP→
←MRPS18A
RSPH9→
VEGFA→

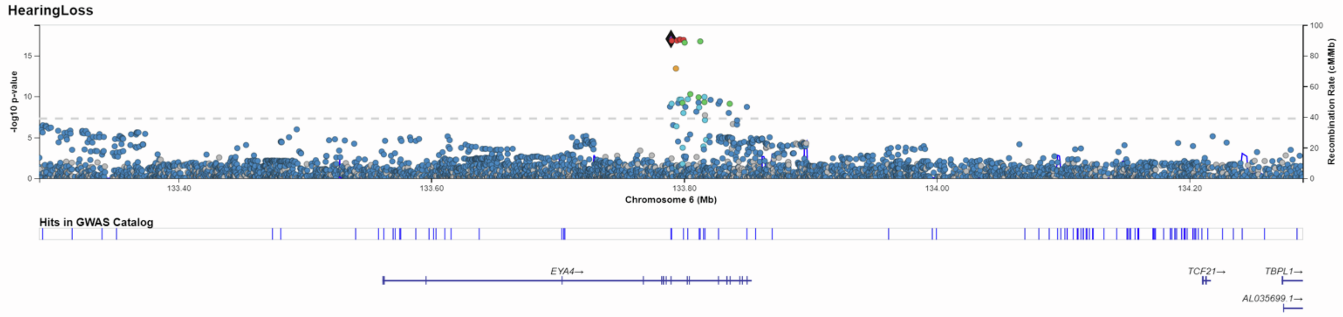
HearingLoss
-log10 p-value
Recombination Rate (cM/Mb)
Chromosome 6 (Mb)
Hits in GWAS Catalog
EYA4→
TCF21→
TBPL1→
AL035699.1→

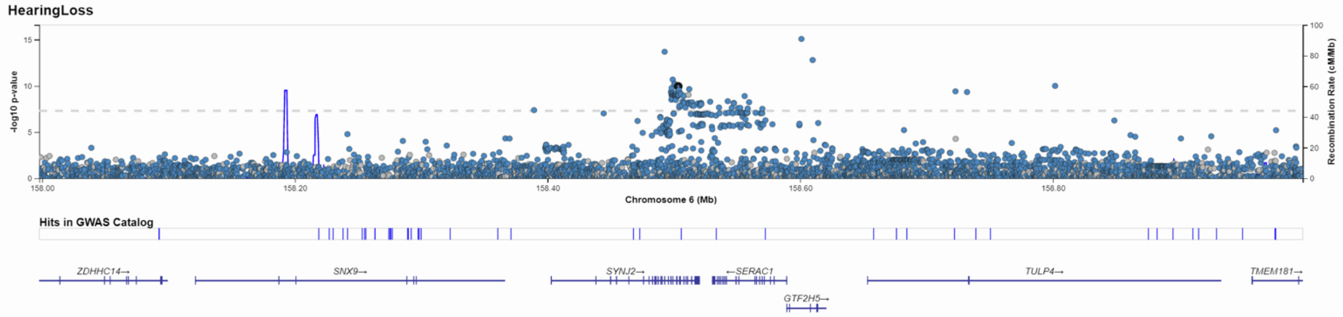
HearingLoss
-log10 p-value
Recombination Rate (cM/Mb)
Chromosome 6 (Mb)
Hits in GWAS Catalog
ZDHHC14→
SNX9→
SYNJ2→
←SERAC1
GTF2H5→
TULP4→
TMEM181→

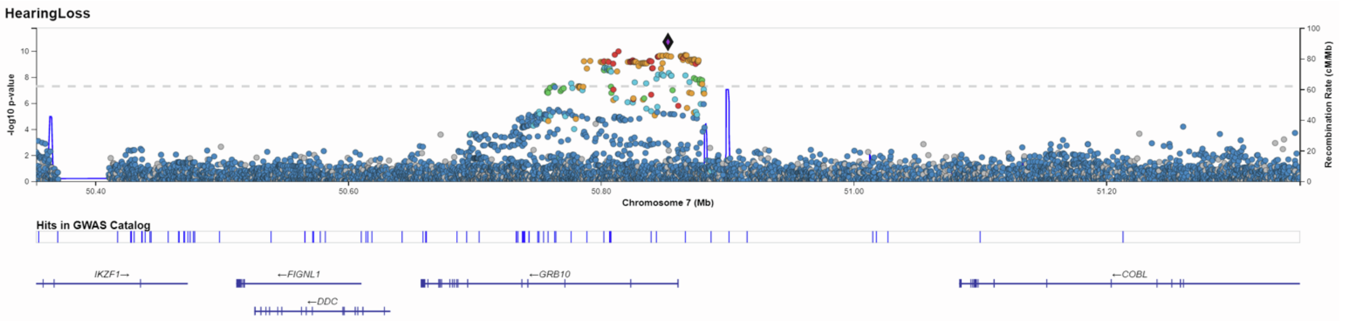
HearingLoss
-log10 p-value
Recombination Rate (cM/Mb)
Chromosome 7 (Mb)
50.40
50.60
50.80
51.00
51.20
Hits in GWAS Catalog
IKZF1→
←FIGNL1
←DDC
←GRB10
←COBL

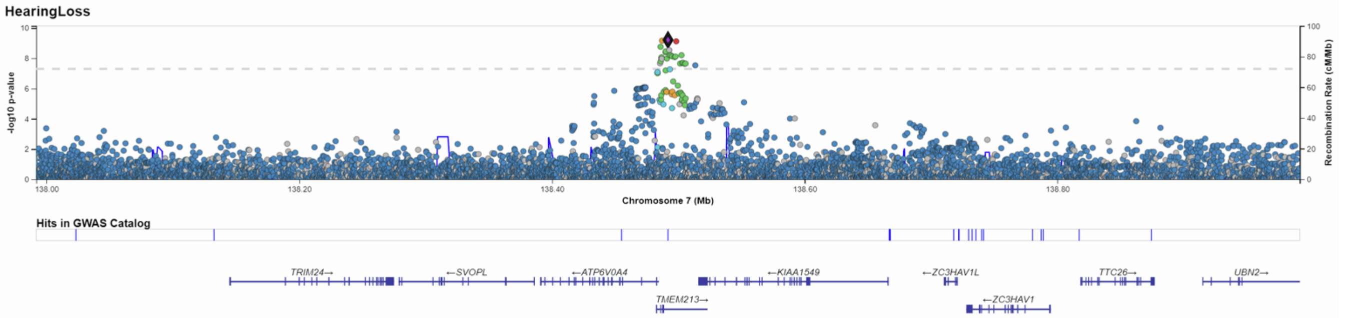
HearingLoss
-log10 p-value
Recombination Rate (cM/Mb)
Chromosome 7 (Mb)
138.00
138.20
138.40
138.60
138.80
Hits in GWAS Catalog
TRIM24→
←SVOPL
←ATP6V0A4
TMEM213→
←KIAA1549
←ZC3HAV1L
←ZC3HAV1
TTC26→
UBN2→

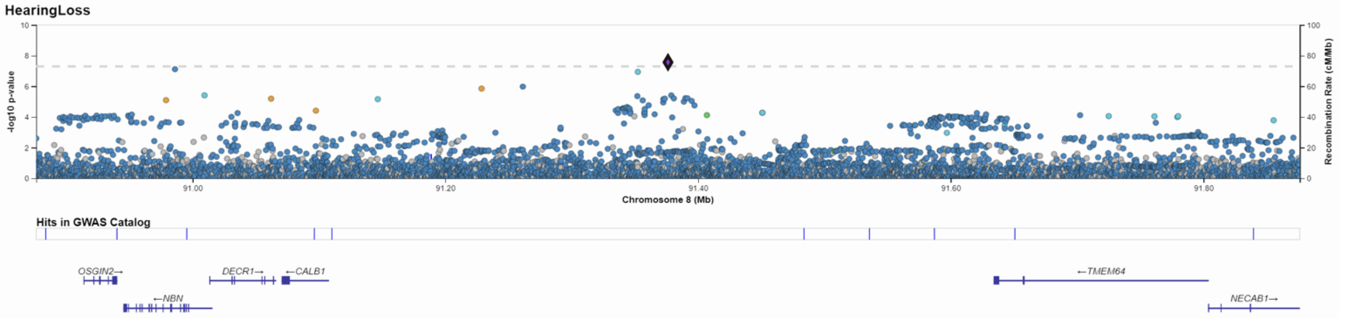
HearingLoss
-log10 p-value
Recombination Rate (cM/Mb)
Chromosome 8 (Mb)
91.00
91.20
91.40
91.60
91.80
Hits in GWAS Catalog
OSGIN2→
←NBN
DECR1→
←CALB1
←TMEM64
NECAB1→

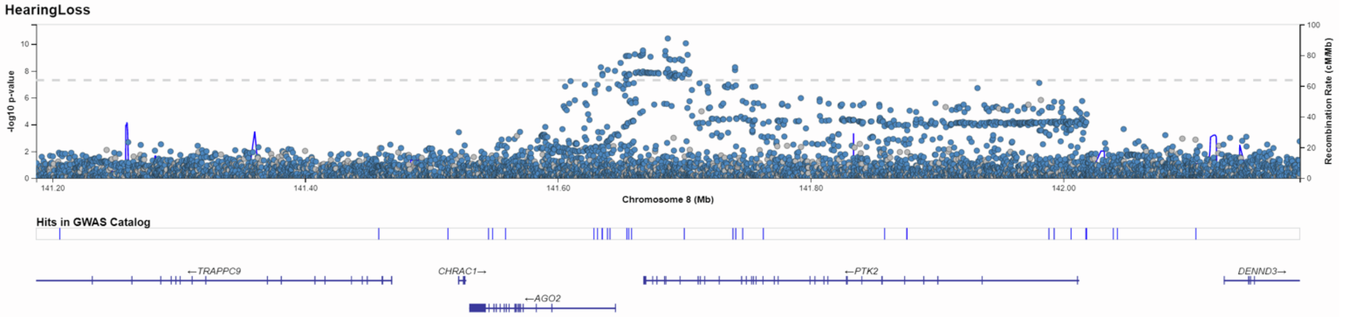
HearingLoss
-log10 p-value
Recombination Rate (cM/Mb)
Chromosome 8 (Mb)
141.20
141.40
141.60
141.80
142.00
Hits in GWAS Catalog
←TRAPPC9
CHRAC1→
←AGO2
←PTK2
DENND3→

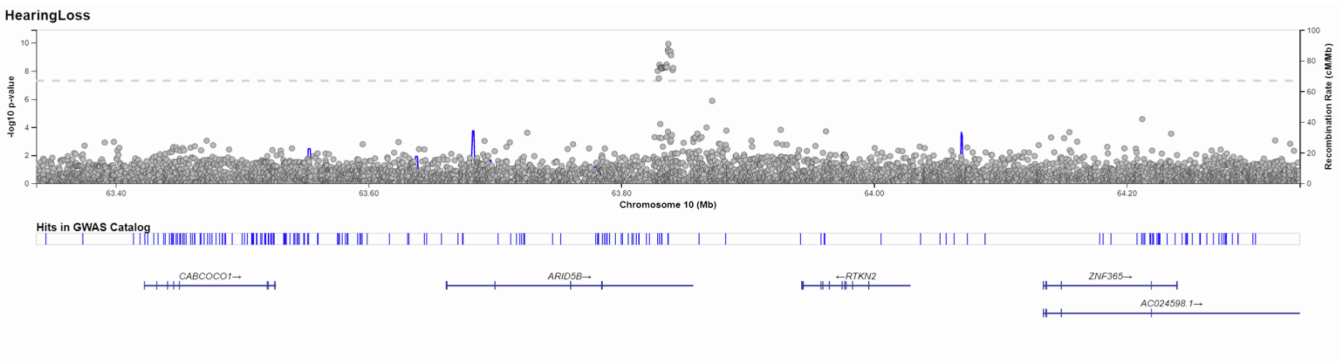
HearingLoss
-log10 p-value
Recombination Rate (cM/Mb)
Chromosome 10 (Mb)
Hits in GWAS Catalog
CABCOCO1→
ARID5B→
←RTKN2
ZNF365→
AC024598.1→

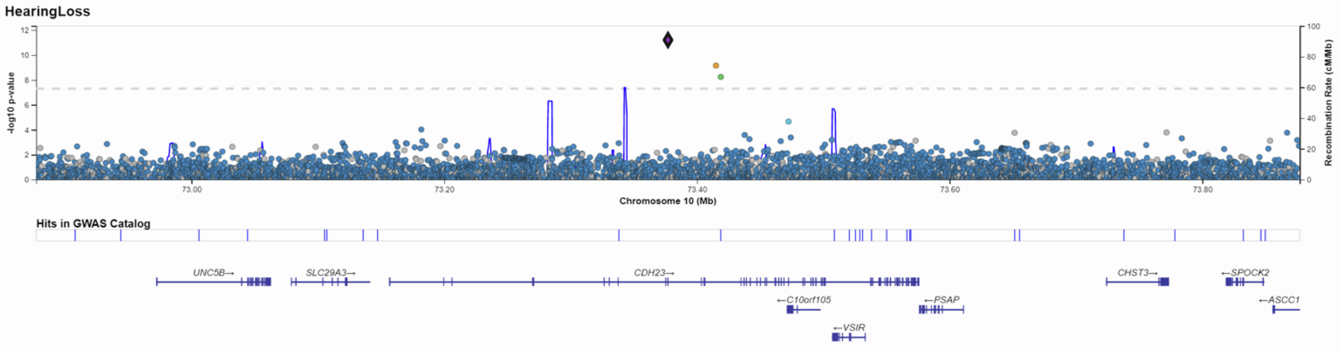
HearingLoss
-log10 p-value
Recombination Rate (cM/Mb)
Chromosome 10 (Mb)
Hits in GWAS Catalog
UNC5B→
SLC29A3→
CDH23→
CHST3→
←SPOCK2
←C10orf105
←PSAP
←ASCC1
←VSIR

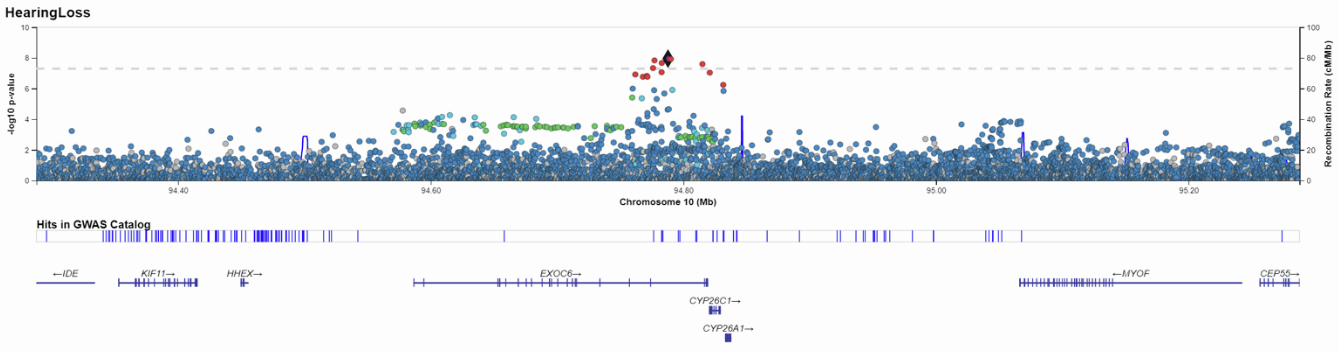
HearingLoss
-log10 p-value
Recombination Rate (cM/Mb)
Chromosome 10 (Mb)
Hits in GWAS Catalog
←IDE
KIF11→
HHEX→
EXOC6→
←MYOF
CEP55→
CYP26C1→
CYP26A1→

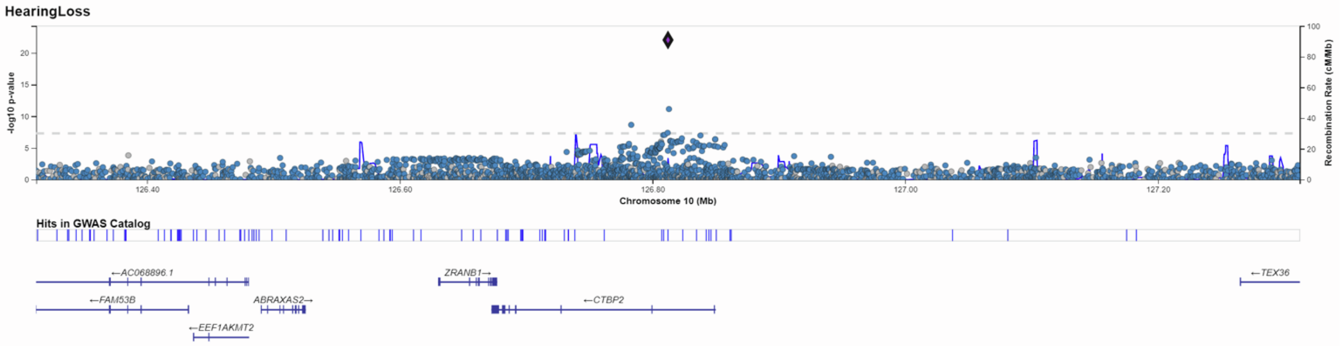
HearingLoss
-log10 p-value
Recombination Rate (cM/Mb)
Chromosome 10 (Mb)
Hits in GWAS Catalog
←AC068896.1
ZRANB1→
←TEX36
←FAM53B
ABRAXAS2→
←CTBP2
←EEF1AKMT2

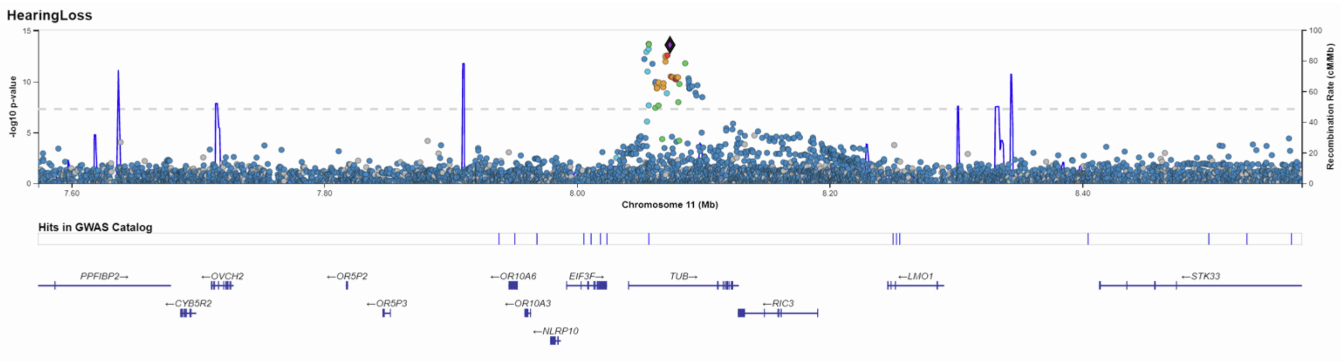

HearingLoss
-log10 p-value
Recombination Rate (cM/Mb)
Chromosome 11 (Mb)
Hits in GWAS Catalog
PPFIBP2→
←OVCH2
←CYB5R2
←OR5P2
←OR5P3
←OR10A6
←OR10A3
←NLRP10
EIF3F→
TUB→
←RIC3
←LMO1
←STK33

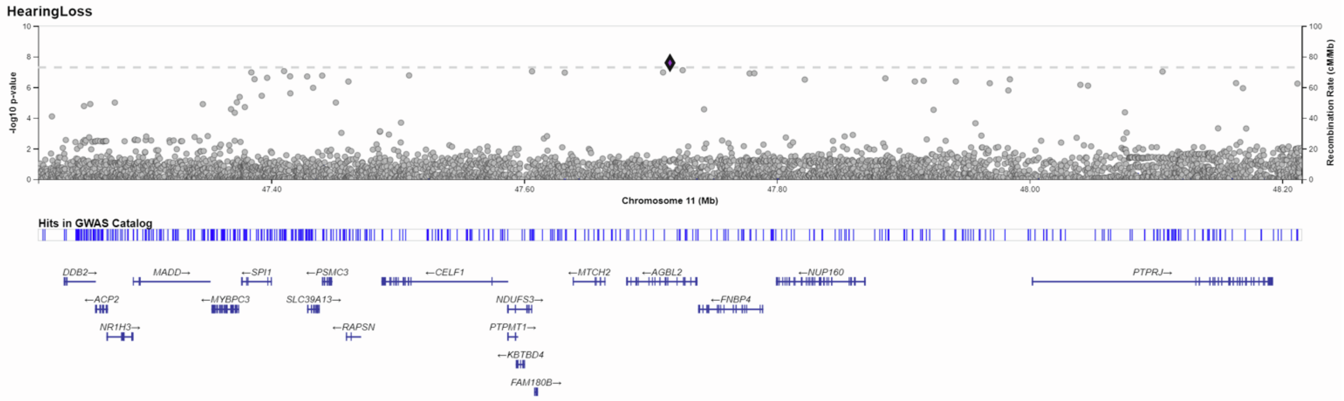

HearingLoss
-log10 p-value
Recombination Rate (cM/Mb)
Chromosome 11 (Mb)
Hits in GWAS Catalog
DDB2→
←ACP2
NR1H3→
MADD→
←MYBPC3
←SPI1
←PSMC3
SLC39A13→
←RAPSN
←CELF1
NDUFS3→
PTPMT1→
←KBTBD4
FAM180B→
←MTCH2
←AGBL2
←FNBP4
←NUP160
PTPRJ→

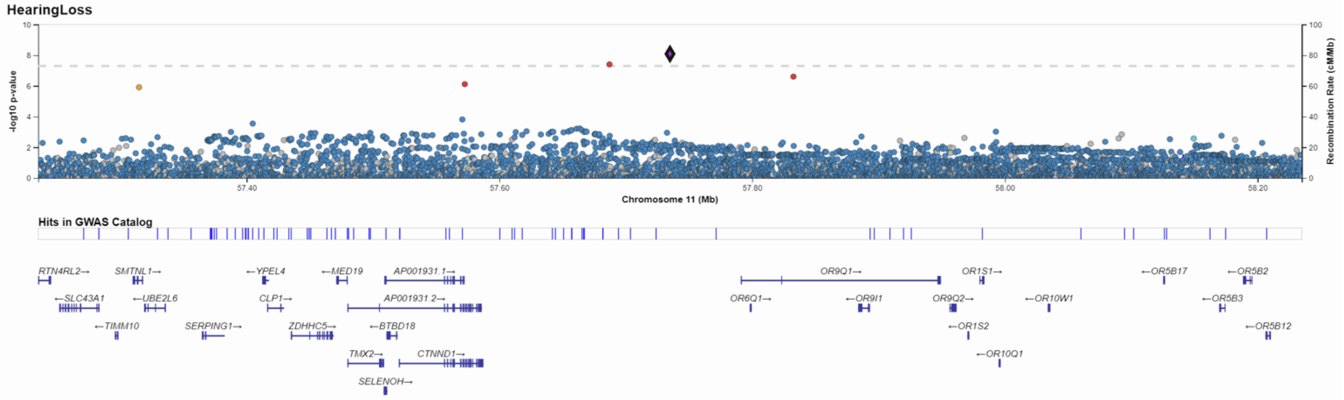

HearingLoss
-log10 p-value
Recombination Rate (cM/Mb)
Chromosome 11 (Mb)
Hits in GWAS Catalog
RTN4RL2→
←SLC43A1
←TIMM10
SMTNL1→
←UBE2L6
SERPING1→
←YPEL4
CLP1→
ZDHHC5→
←MED19
←BTBD18
TMX2→
SELENOH→
AP001931.1→
AP001931.2→
CTNND1→
OR9Q1→
OR6Q1→
←OR9I1
OR9Q2→
OR1S1→
←OR1S2
←OR10Q1
←OR10W1
←OR5B17
←OR5B2
←OR5B3
←OR5B12

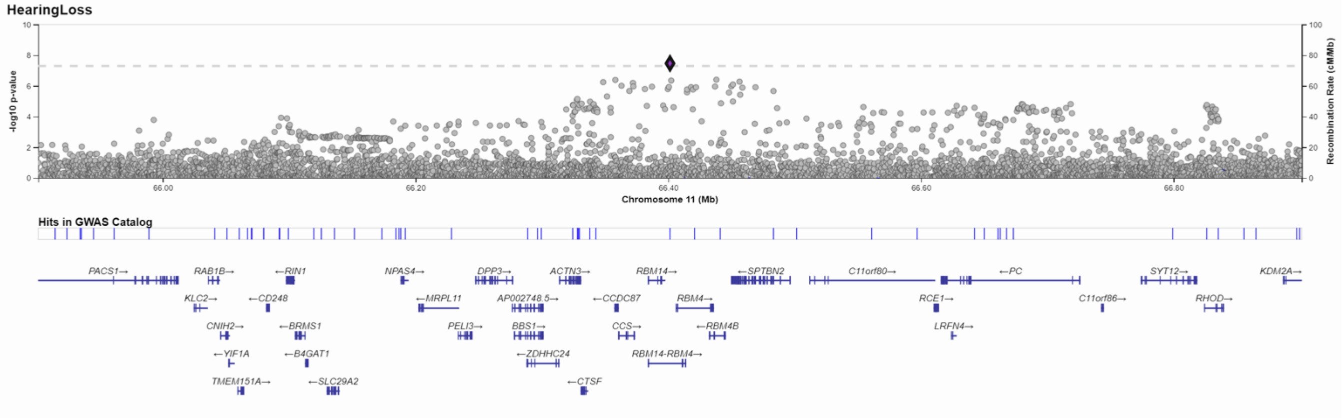

HearingLoss
-log10 p-value
Recombination Rate (cM/Mb)
Chromosome 11 (Mb)
Hits in GWAS Catalog
PACS1→
RAB1B→
KLC2→
CNIH2→
←YIF1A
TMEM151A→
←RIN1
←CD248
←BRMS1
←B4GAT1
←SLC29A2
NPAS4→
←MRPL11
PELI3→
DPP3→
AP002748.5→
BBS1→
←ZDHHC24
ACTN3→
←CCDC87
CCS→
←CTSF
RBM14→
RBM4→
←RBM4B
RBM14-RBM4→
←SPTBN2
C11orf80→
RCE1→
LRFN4→
←PC
C11orf86→
SYT12→
RHOD→
KDM2A→

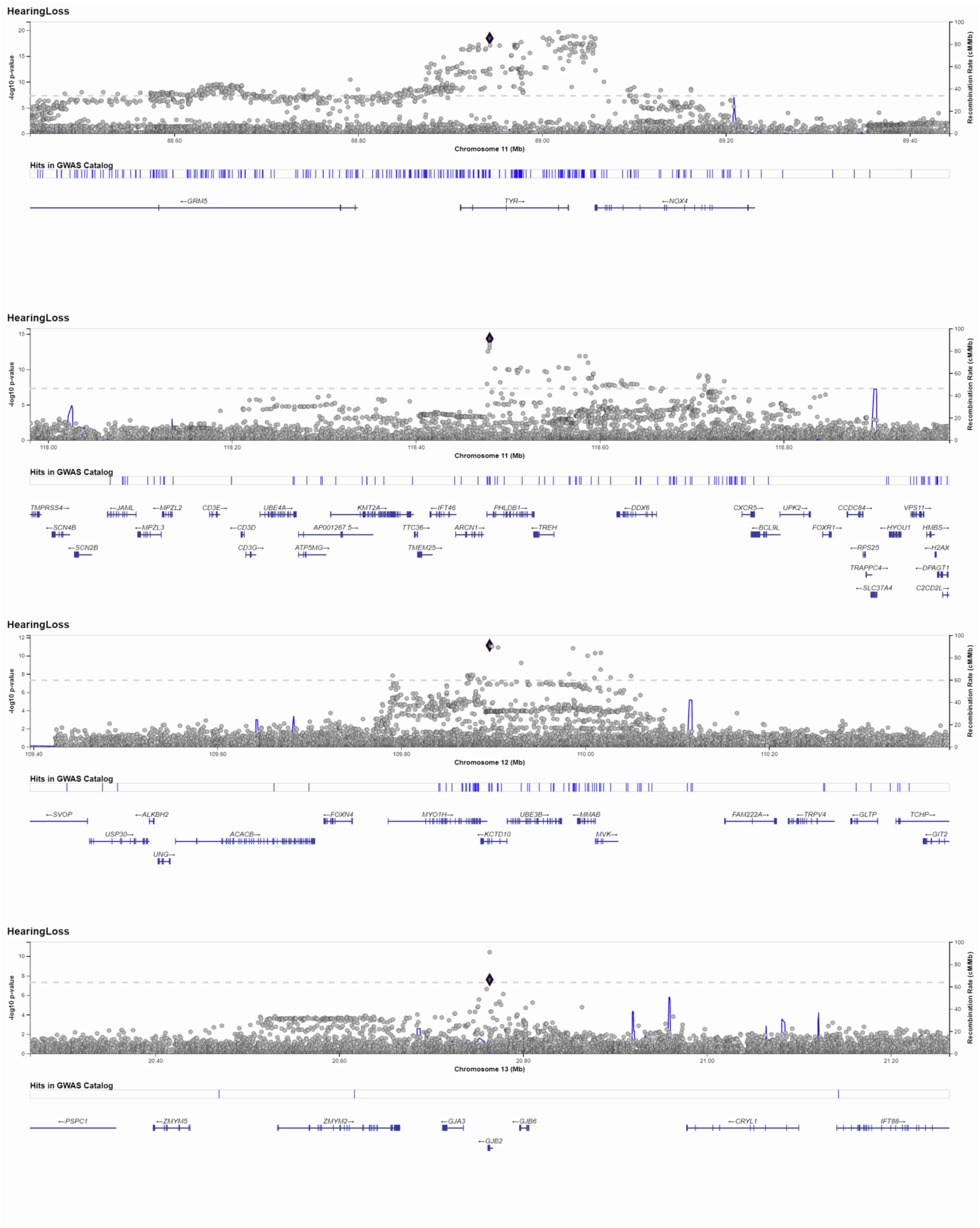
HearingLoss
-log10 p-value
Recombination Rate (cM/Mb)
Chromosome 11 (Mb)
Hits in GWAS Catalog
←GRM5
TYR→
←NOX4
HearingLoss
-log10 p-value
Recombination Rate (cM/Mb)
Chromosome 11 (Mb)
Hits in GWAS Catalog
TMPRSS4→
←JAML
←MPZL2
CD3E→
UBE4A→
KMT2A→
←IFT46
PHLDB1→
←DDX6
CXCR5→
UPK2→
CCDC84→
VPS11→
←SCN4B
←MPZL3
←CD3D
AP001267.5→
TTC36→
ARCN1→
←TREH
←BCL9L
FOXR1→
←HYOU1
HMBS→
←SCN2B
CD3G→
ATP5MG→
TMEM25→
←RPS25
←H2AX
TRAPPC4→
←DPAGT1
←SLC37A4
C2CD2L→
HearingLoss
-log10 p-value
Recombination Rate (cM/Mb)
Chromosome 12 (Mb)
Hits in GWAS Catalog
←SVOP
←ALKBH2
←FOXN4
MYO1H→
UBE3B→
←MMAB
FAM222A→
←TRPV4
←GLTP
TCHP→
USP30→
ACACB→
←KCTD10
MVK→
←GIT2
UNG→
HearingLoss
-log10 p-value
Recombination Rate (cM/Mb)
Chromosome 13 (Mb)
Hits in GWAS Catalog
←PSPC1
←ZMYM5
ZMYM2→
←GJA3
←GJB6
←CRYL1
IFT88→
←GJB2

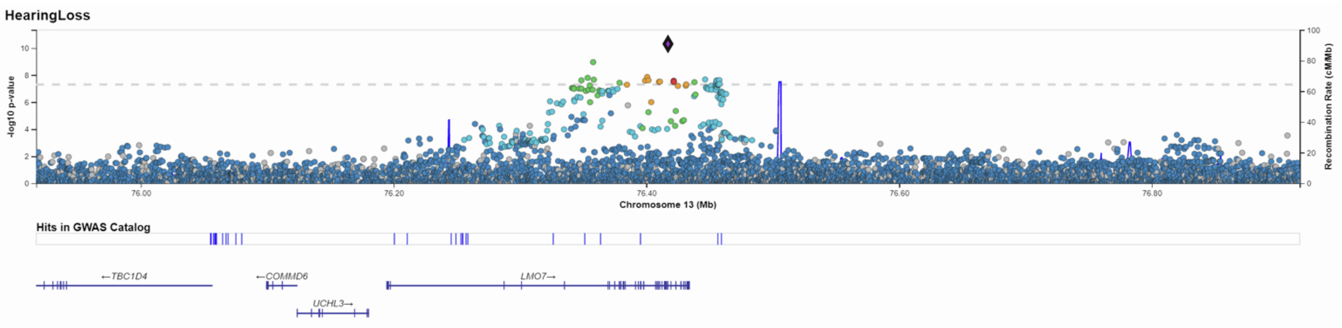
HearingLoss
-log10 p-value
Recombination Rate (cM/Mb)
Chromosome 13 (Mb)
Hits in GWAS Catalog
←TBC1D4
←COMMD6
UCHL3→
LMO7→

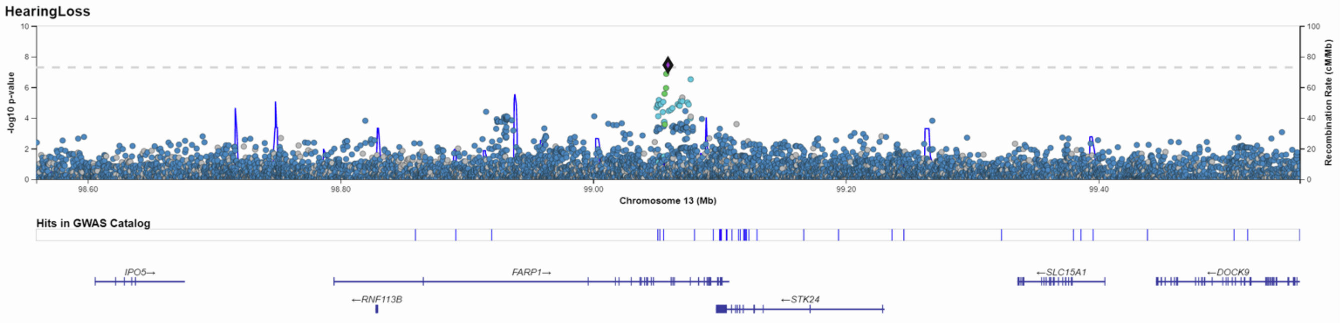
HearingLoss
-log10 p-value
Recombination Rate (cM/Mb)
Chromosome 13 (Mb)
Hits in GWAS Catalog
IPO5→
FARP1→
←RNF113B
←STK24
←SLC15A1
←DOCK9

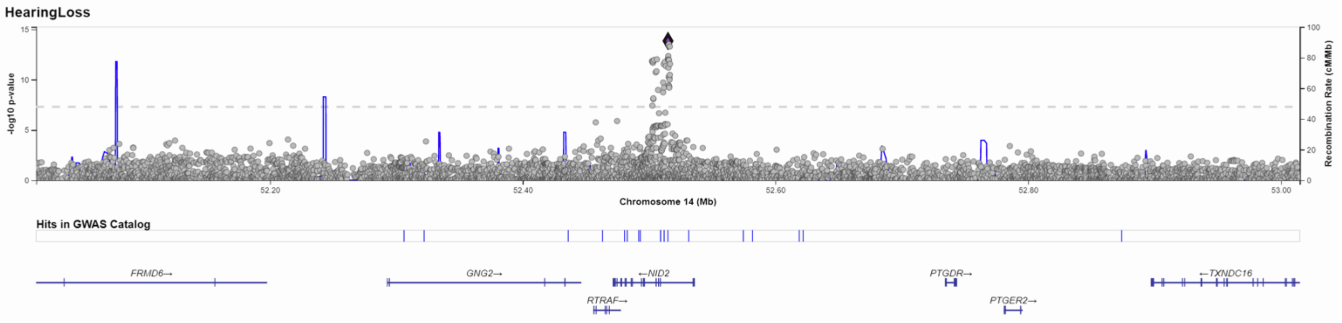
HearingLoss
-log10 p-value
Recombination Rate (cM/Mb)
Chromosome 14 (Mb)
Hits in GWAS Catalog
FRMD6→
GNG2→
←NID2
RTRAF→
PTGDR→
PTGER2→
←TXNDC16

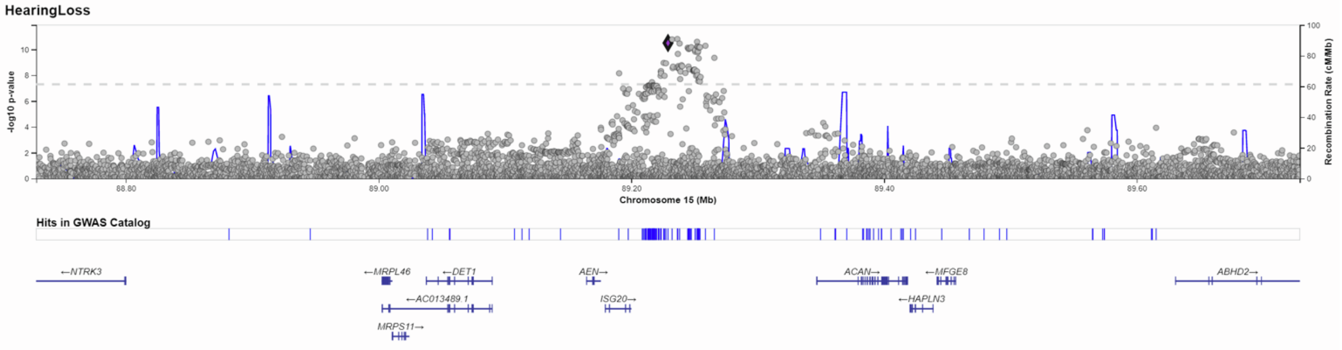
HearingLoss
-log10 p-value
Recombination Rate (cM/Mb)
Chromosome 15 (Mb)
Hits in GWAS Catalog
←NTRK3
←MRPL46
←DET1
←AC013489.1
MRPS11→
AEN→
ISG20→
ACAN→
←MFGE8
←HAPLN3
ABHD2→

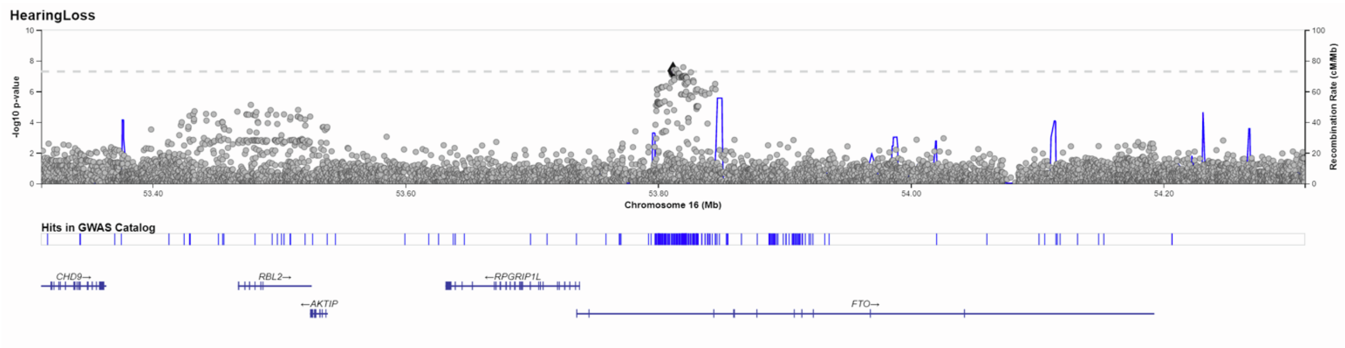
HearingLoss
-log10 p-value
Recombination Rate (cM/Mb)
Chromosome 16 (Mb)
53.40
53.60
53.80
54.00
54.20
Hits in GWAS Catalog
CHD9→
RBL2→
←AKTIP
←RPGRIP1L
FTO→

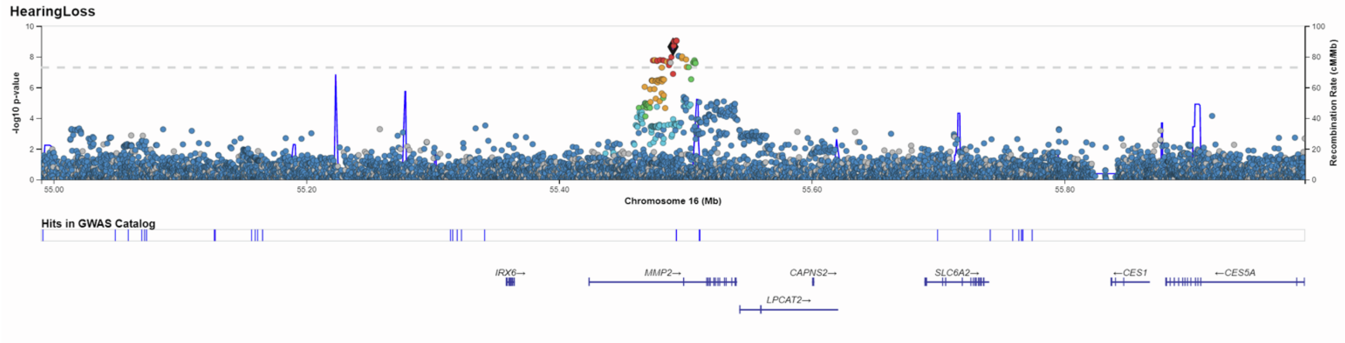
HearingLoss
-log10 p-value
Recombination Rate (cM/Mb)
Chromosome 16 (Mb)
55.00
55.20
55.40
55.60
55.80
Hits in GWAS Catalog
IRX6→
MMP2→
CAPNS2→
LPCAT2→
SLC6A2→
←CES1
←CES5A

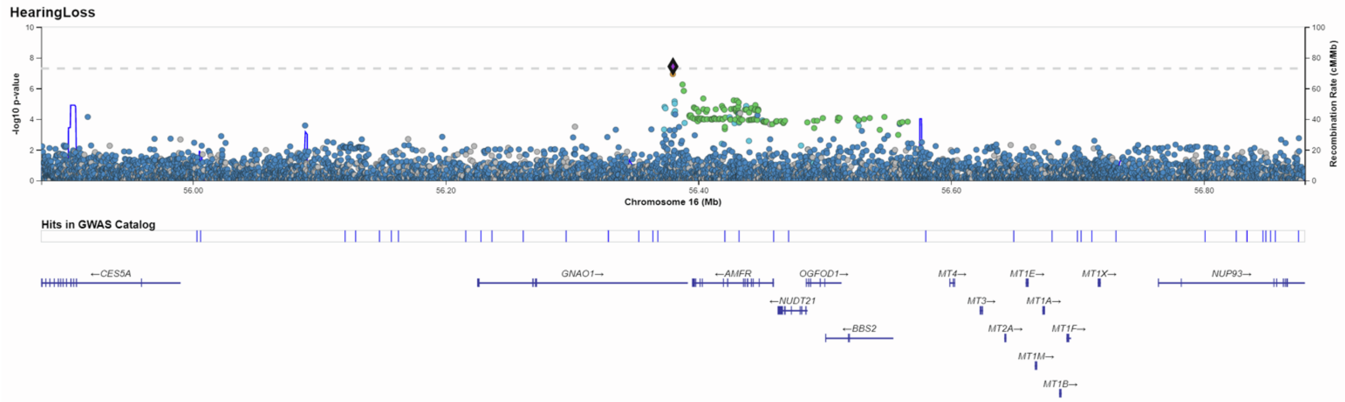
HearingLoss
-log10 p-value
Recombination Rate (cM/Mb)
Chromosome 16 (Mb)
56.00
56.20
56.40
56.60
56.80
Hits in GWAS Catalog
←CES5A
GNAO1→
←AMFR
OGFOD1→
←NUDT21
←BBS2
MT4→
MT3→
MT2A→
MT1M→
MT1E→
MT1A→
MT1B→
MT1X→
MT1F→
NUP93→

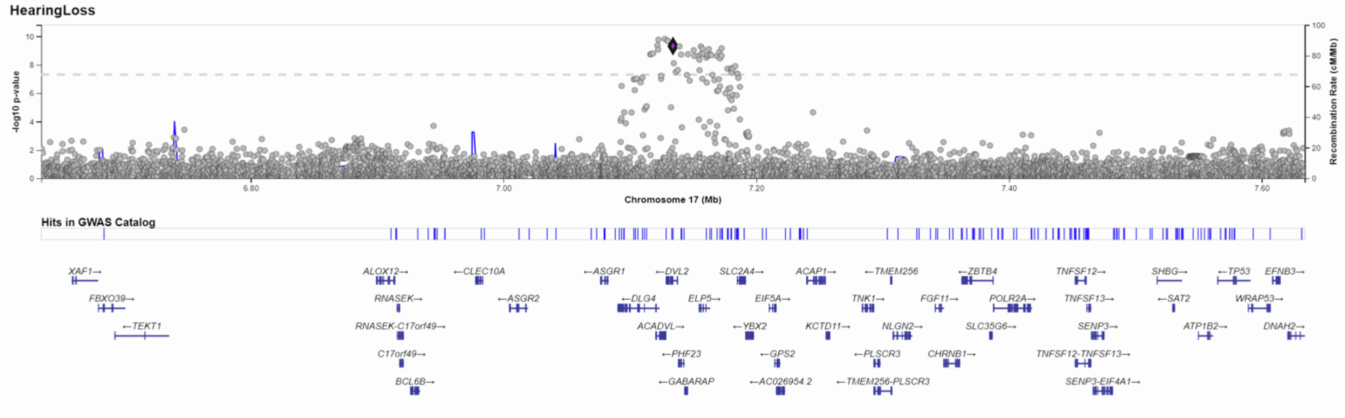
HearingLoss
-log10 p-value
Recombination Rate (cM/Mb)
Chromosome 17 (Mb)
6.80
7.00
7.20
7.40
7.60
Hits in GWAS Catalog
XAF1→
FBXO39→
←TEKT1
ALOX12→
RNASEK→
RNASEK-C17orf49→
C17orf49→
BCL6B→
←CLEC10A
←ASGR2
←ASGR1
←DLG4
←DVL2
ACADVL→
←PHF23
←GABARAP
ELP5→
SLC2A4→
←YBX2
←AC026954.2
EIF5A→
←GPS2
ACAP1→
KCTD11→
←TMEM256
TNK1→
←PLSCR3
←TMEM256-PLSCR3
NLGN2→
←ZBTB4
FGF11→
CHRNB1→
POLR2A→
SLC35G6→
TNFSF12→
TNFSF13→
SENP3→
TNFSF12-TNFSF13→
SENP3-EIF4A1→
SHBG→
←SAT2
←TP53
ATP1B2→
EFNB3→
WRAP53→
DNAH2→

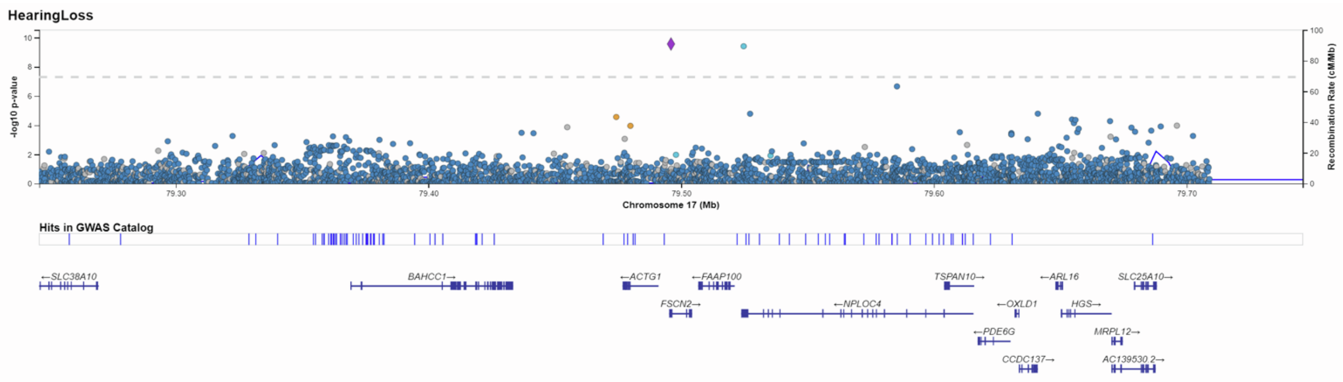
HearingLoss
-log10 p-value
Recombination Rate (cM/Mb)
Chromosome 17 (Mb)
Hits in GWAS Catalog
←SLC38A10
BAHCC1→
←ACTG1
←FAAP100
FSCN2→
←NPLOC4
TSPAN10→
←ARL16
SLC25A10→
←OXLD1
HGS→
←PDE6G
MRPL12→
CCDC137→
AC139530.2→

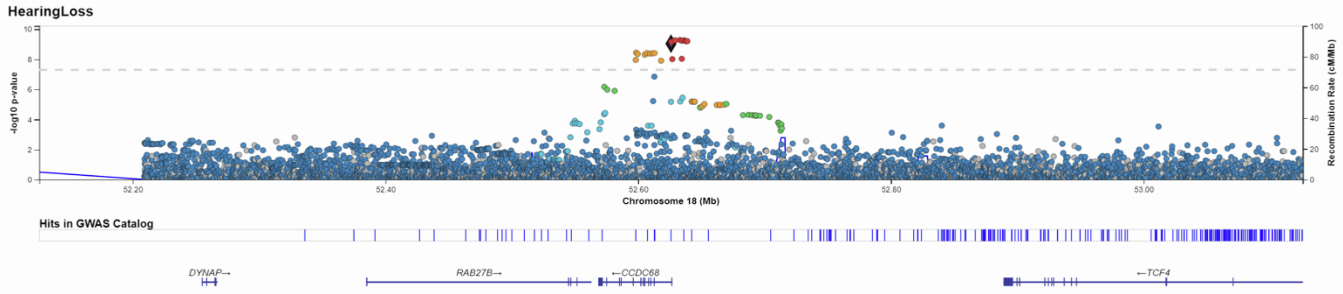
HearingLoss
-log10 p-value
Recombination Rate (cM/Mb)
Chromosome 18 (Mb)
Hits in GWAS Catalog
DYNAP→
RAB27B→
←CCDC68
←TCF4

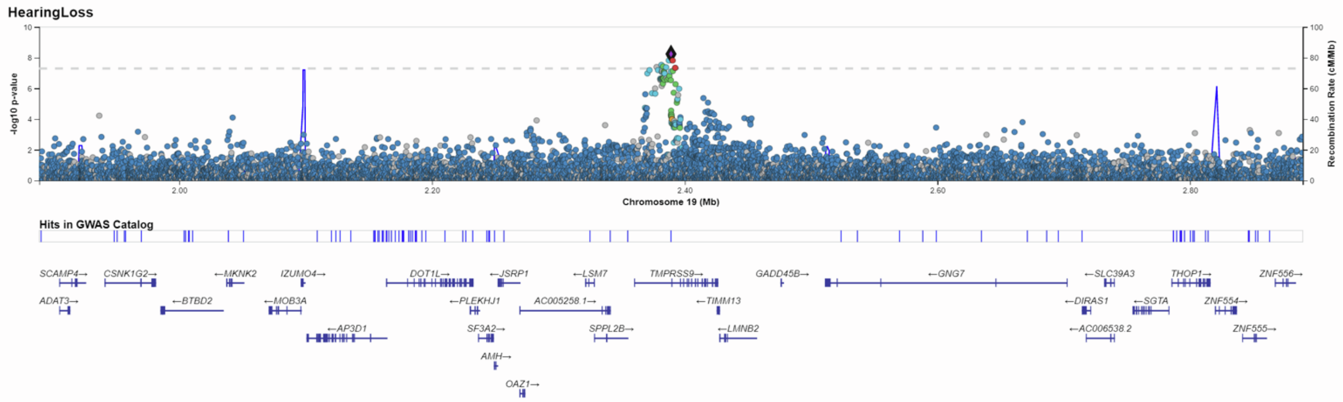
HearingLoss
-log10 p-value
Recombination Rate (cM/Mb)
Chromosome 19 (Mb)
Hits in GWAS Catalog
SCAMP4→
CSNK1G2→
←MKNK2
IZUMO4→
DOT1L→
←JSRP1
←LSM7
TMPRSS9→
GADD45B→
←GNG7
←SLC39A3
THOP1→
ZNF556→
ADAT3→
←BTBD2
←MOB3A
←PLEKHJ1
AC005258.1→
←TIMM13
←DIRAS1
←SGTA
ZNF554→
←AP3D1
SF3A2→
SPPL2B→
←LMNB2
←AC006538.2
ZNF555→
AMH→
OAZ1→

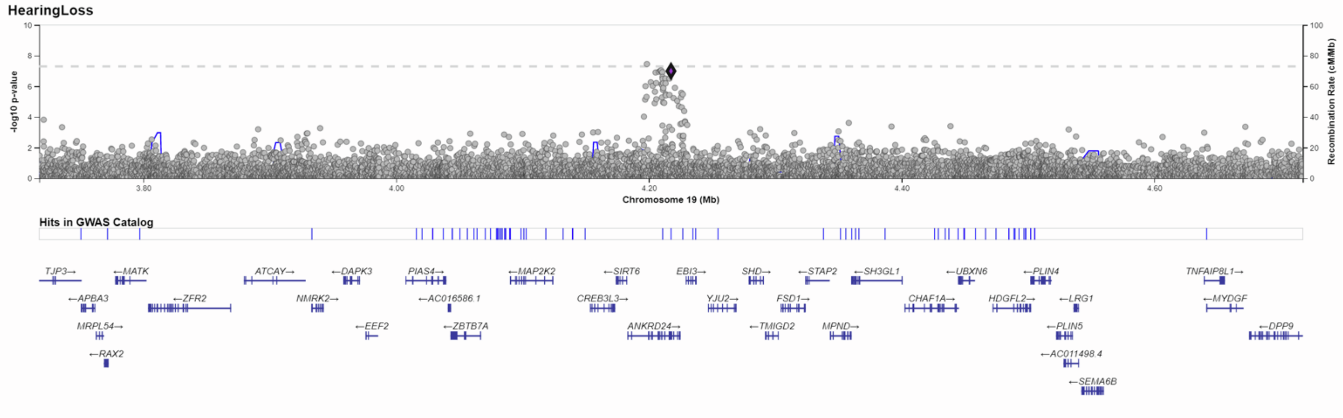
HearingLoss
-log10 p-value
Recombination Rate (cM/Mb)
Chromosome 19 (Mb)
Hits in GWAS Catalog
TJP3→
←MATK
ATCAY→
←DAPK3
PIAS4→
←MAP2K2
←SIRT6
EBI3→
SHD→
←STAP2
←SH3GL1
←UBXN6
←PLIN4
TNFAIP8L1→
←APBA3
←ZFR2
NMRK2→
←AC016586.1
CREB3L3→
YJU2→
FSD1→
CHAF1A→
HDGFL2→
←LRG1
←MYDGF
MRPL54→
←EEF2
←ZBTB7A
ANKRD24→
←TMIGD2
MPND→
←PLIN5
←DPP9
←RAX2
←AC011498.4
←SEMA6B

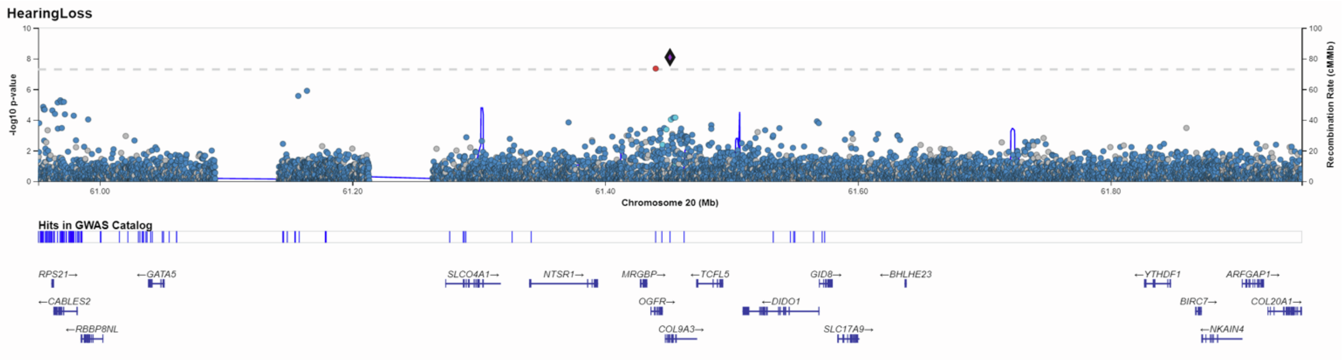
HearingLoss
-log10 p-value
Recombination Rate (cM/Mb)
Chromosome 20 (Mb)
Hits in GWAS Catalog
RPS21
GATA5
CABLES2
RBBP8NL
SLCO4A1
NTSR1
MRGBP
OGFR
COL9A3
TCFL5
DIDO1
GID8
SLC17A9
BHLHE23
YTHDF1
BIRC7
NKAIN4
ARFGAP1
COL20A1

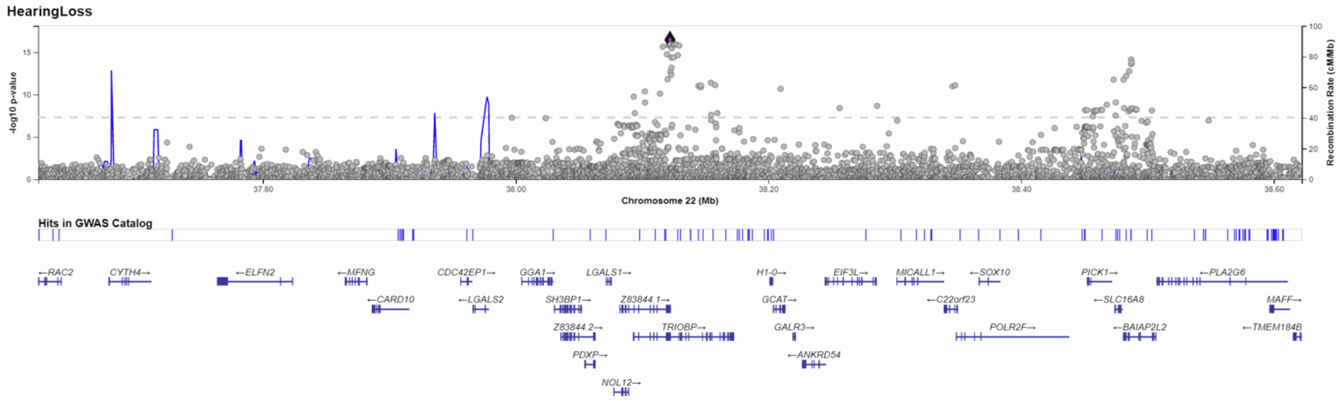
HearingLoss
-log10 p-value
Recombination Rate (cM/Mb)
Chromosome 22 (Mb)
Hits in GWAS Catalog
RAC2
CYTH4
ELFN2
MFNG
CARD10
CDC42EP1
LGALS2
GGA1
SH3BP1
Z83844.2
PDXP
NOL12
LGALS1
Z83844.1
TRIOBP
H1-0
GCAT
GALR3
ANKRD54
EIF3L
MICALL1
C22orf23
POLR2F
SOX10
PICK1
SLC16A8
BAIAP2L2
PLA2G6
MAFF
TMEM184B

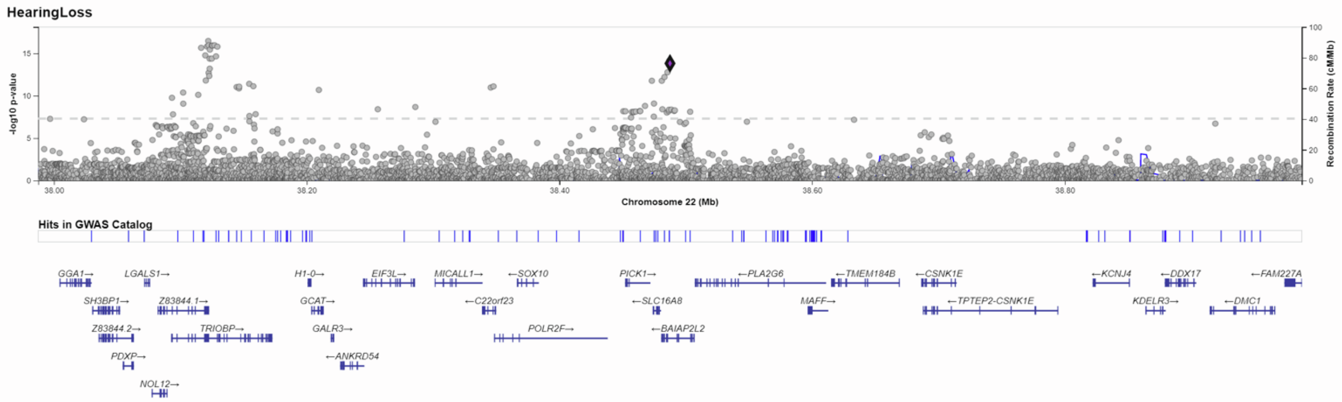
HearingLoss
-log10 p-value
Recombination Rate (cM/Mb)
Chromosome 22 (Mb)
Hits in GWAS Catalog
GGA1
SH3BP1
Z83844.2
PDXP
NOL12
LGALS1
Z83844.1
TRIOBP
H1-0
GCAT
GALR3
ANKRD54
EIF3L
MICALL1
C22orf23
POLR2F
SOX10
PICK1
SLC16A8
BAIAP2L2
PLA2G6
MAFF
TMEM184B
CSNK1E
TPTEP2-CSNK1E
KCNJ4
KDELR3
DDX17
DMC1
FAM227A

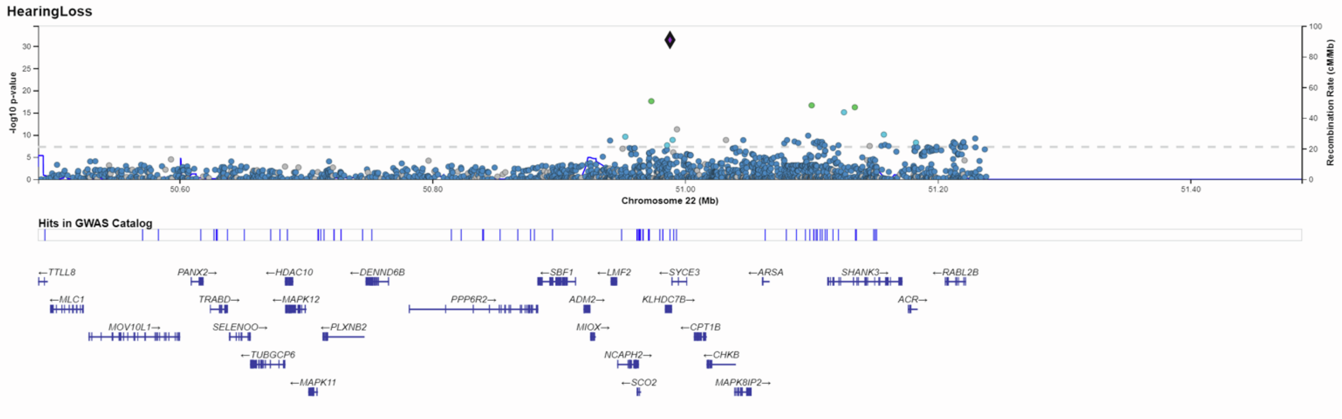
HearingLoss
-log10 p-value
Recombination Rate (cM/Mb)
Chromosome 22 (Mb)
Hits in GWAS Catalog
TTLL8
MLC1
MOV10L1
PANX2
TRABD
SELENOO
TUBGCP6
HDAC10
MAPK12
MAPK11
PLXNB2
DENND6B
PPP6R2
SBF1
ADM2
MIOX
NCAPH2
SCO2
LMF2
SYCE3
KLHDC7B
CPT1B
CHKB
MAPK8IP2
ARSA
SHANK3
ACR
RABL2B

Figure S3. Forest plots for each statistically significant and independent SNP

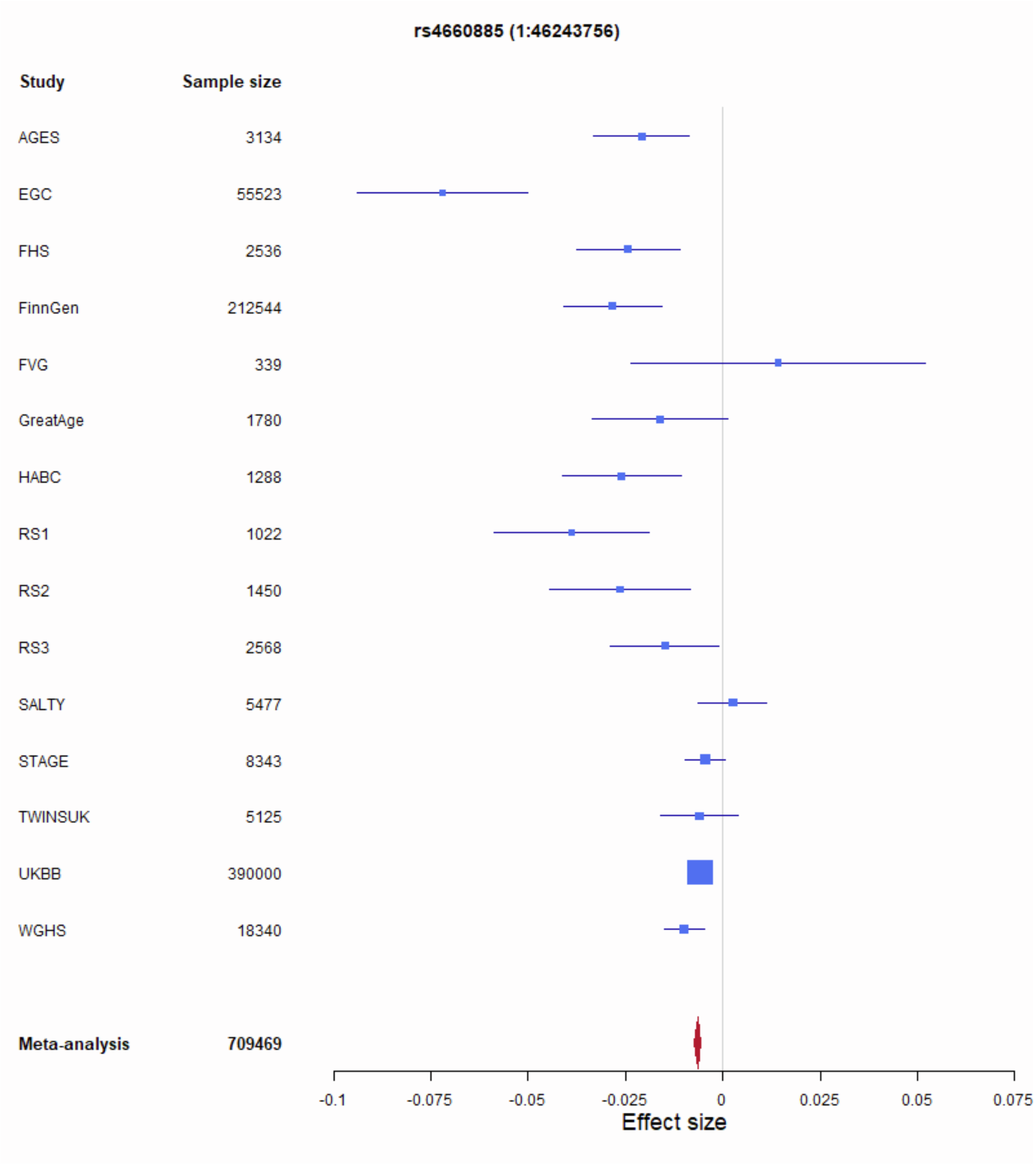

Locus annotation: IPP-[x]-MAST2

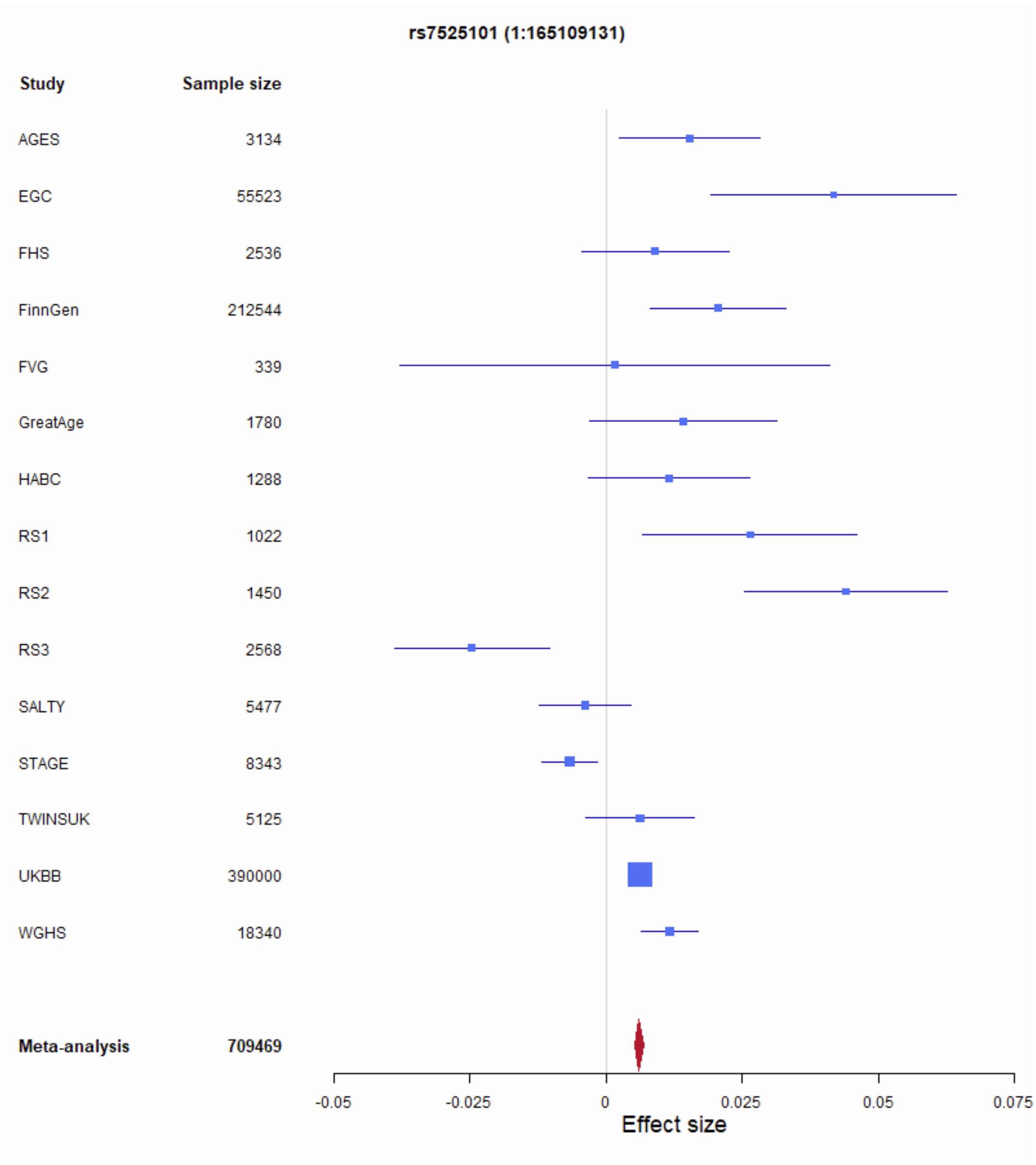

Locus annotation: PBX1---[x]-LMX1A

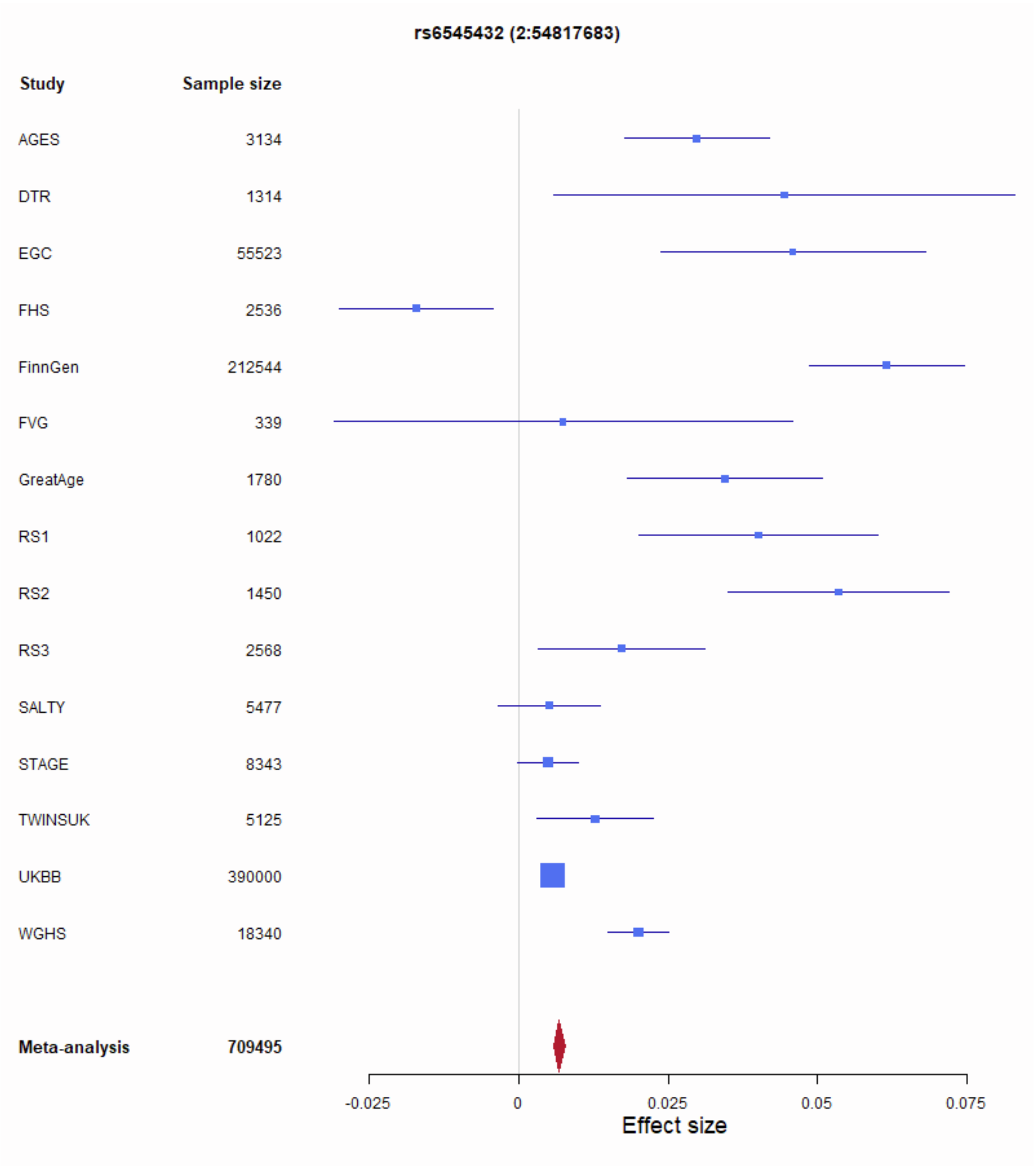

Locus annotation: [SPTBN1] intronic

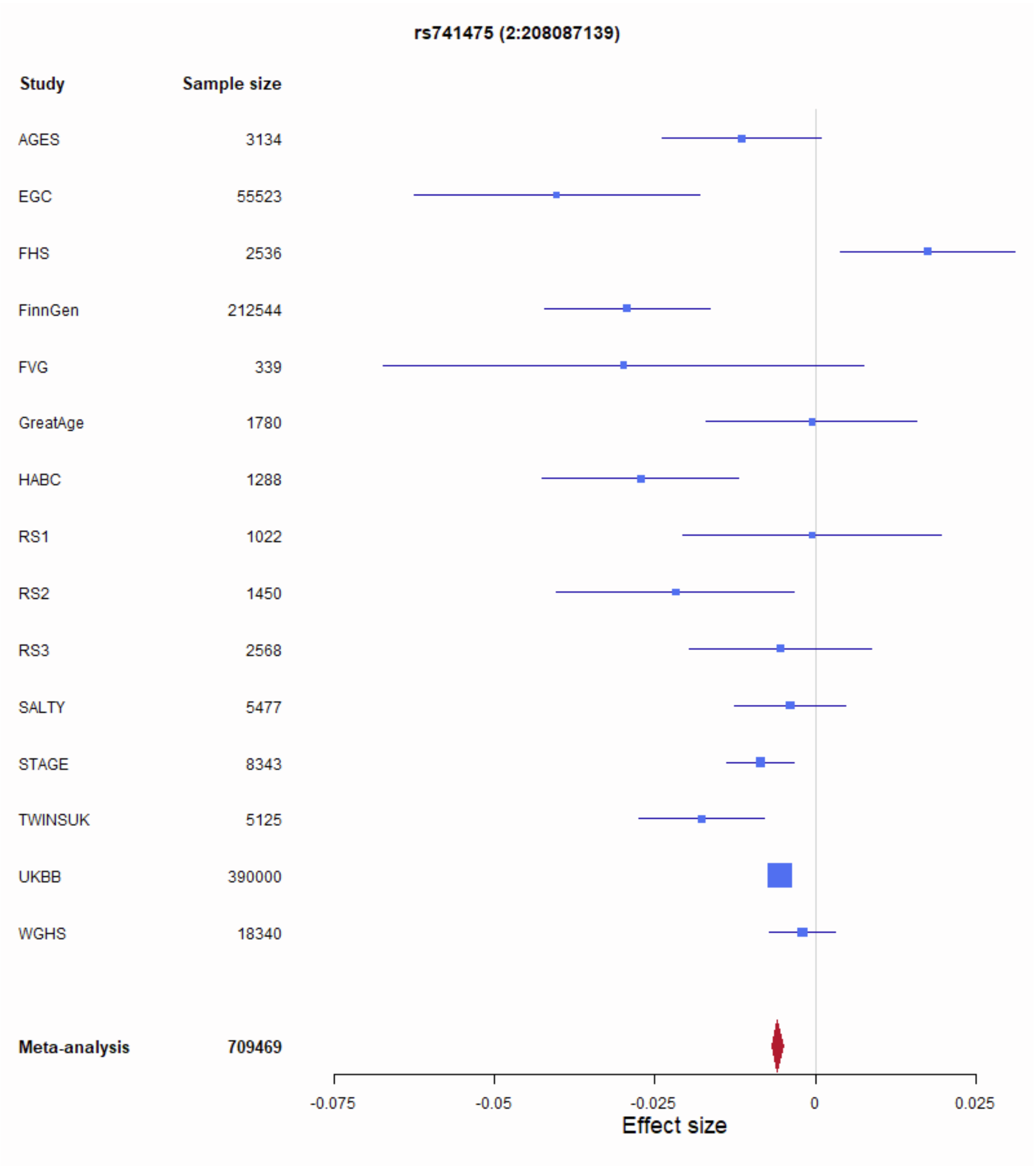

Locus annotation: KLF7-[x]--CREB1

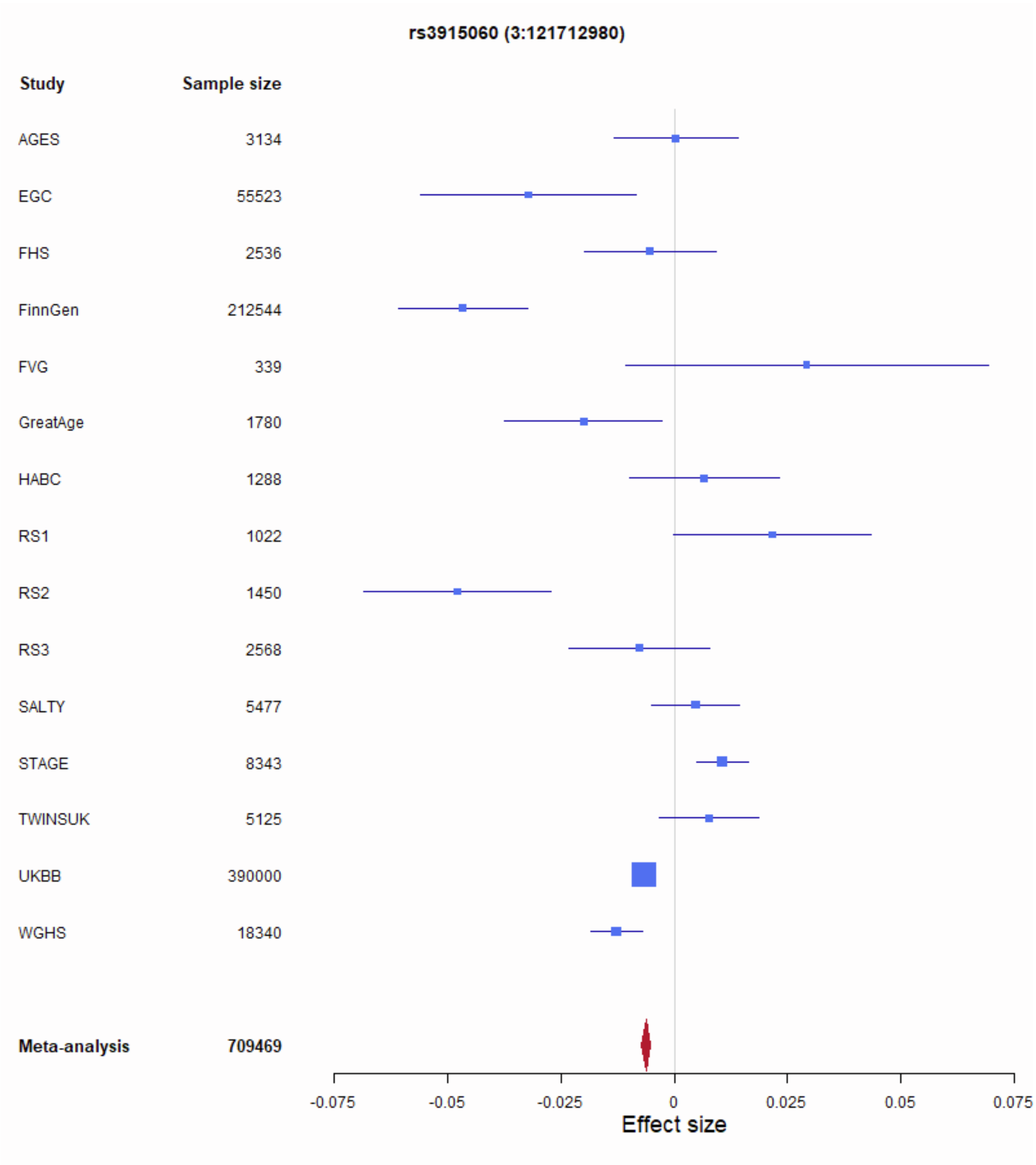

Locus annotation: [ILDR1] intronic

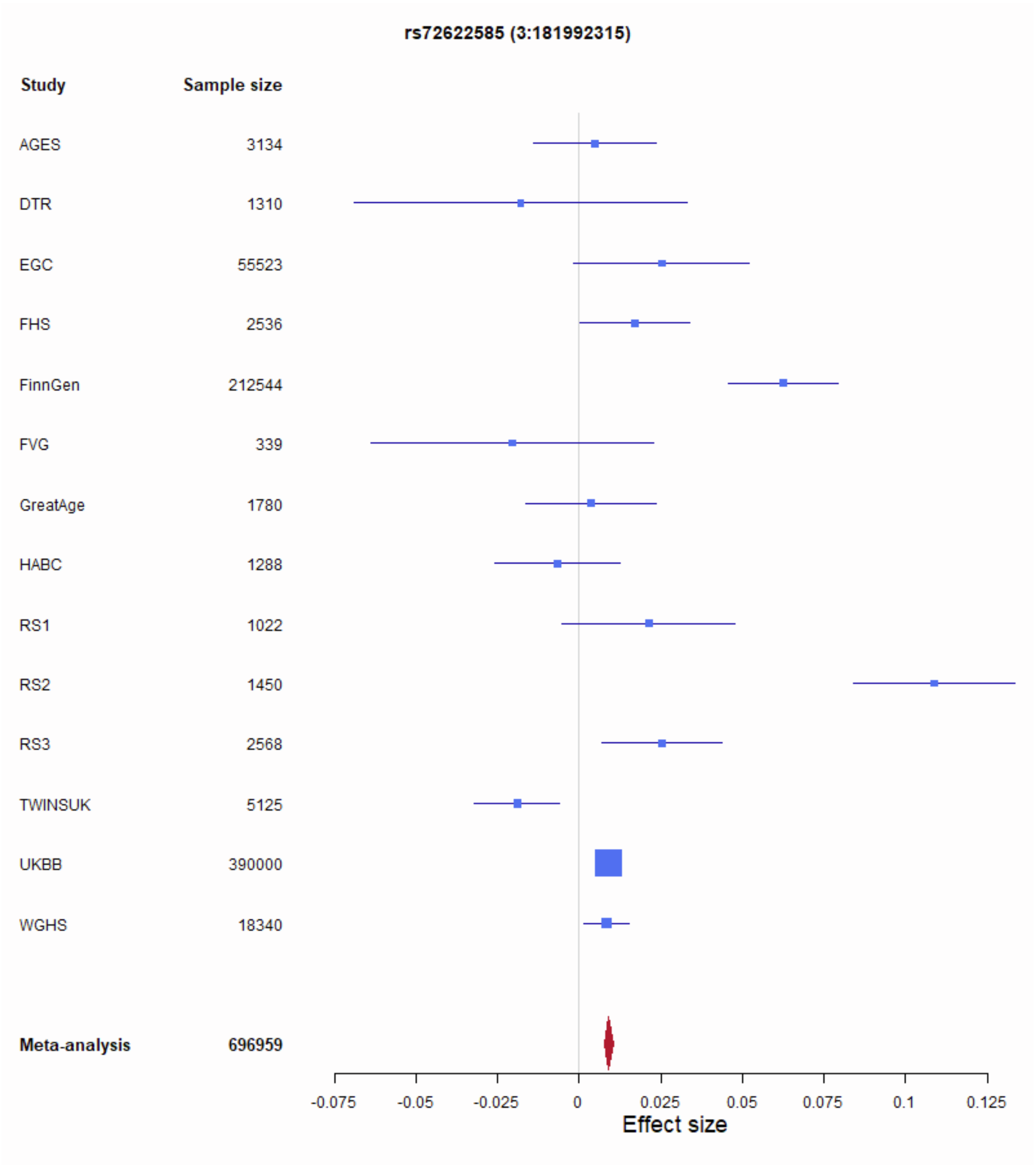

Locus annotation: SOX2---[x]---ATP11B

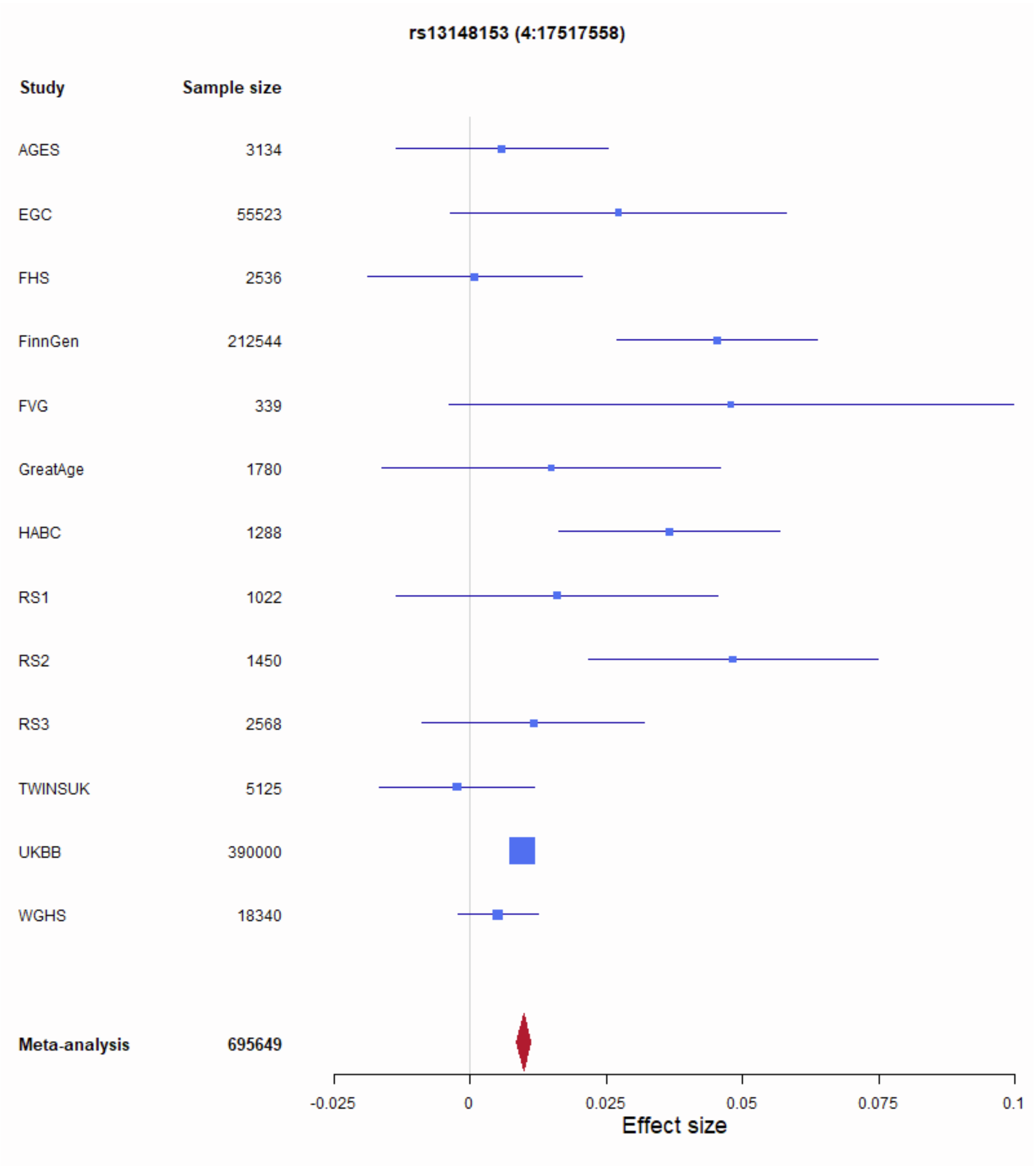

Locus annotation: [CLRN2] intronic

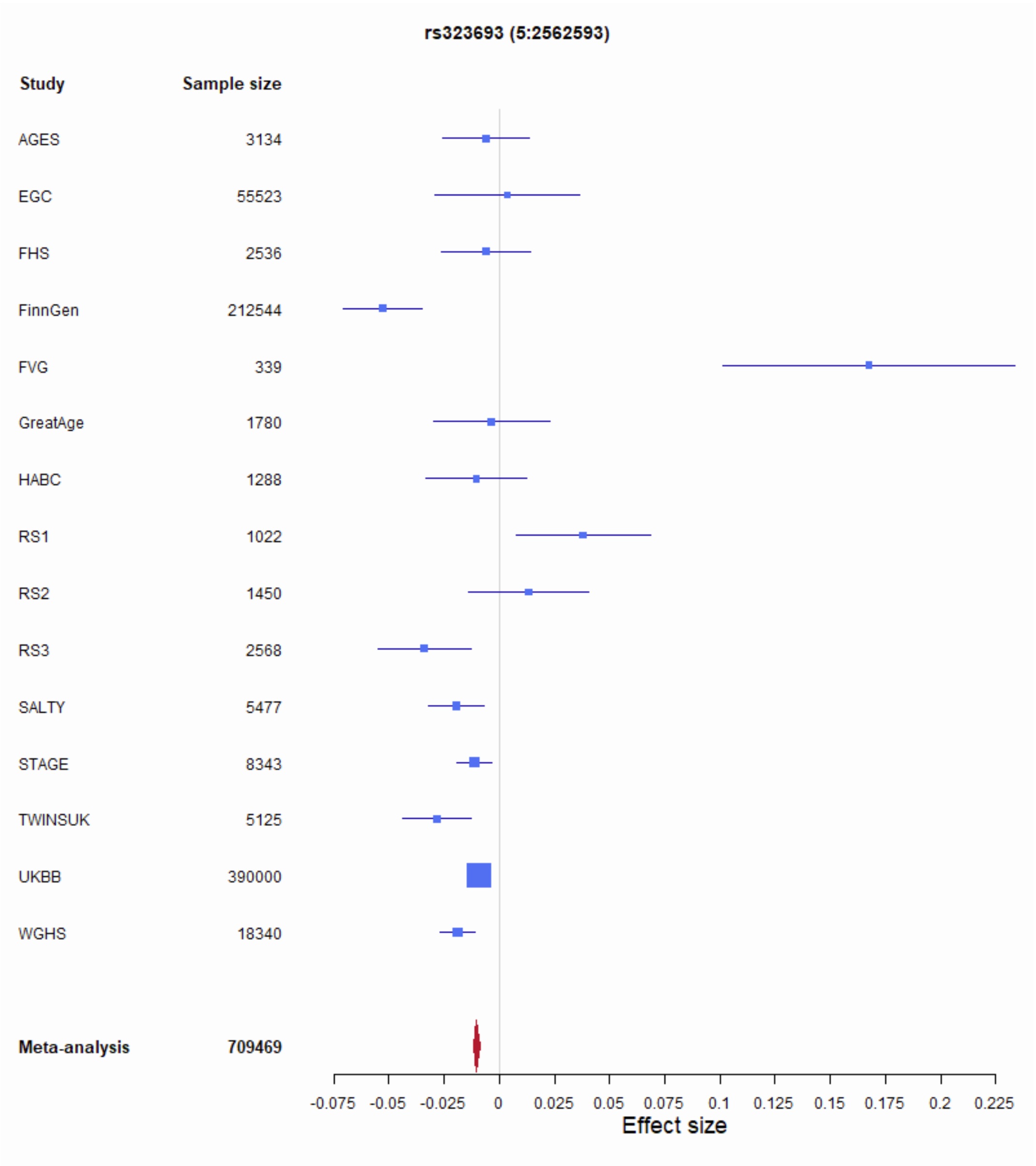

Locus annotation: IRX4---[x]--IRX2

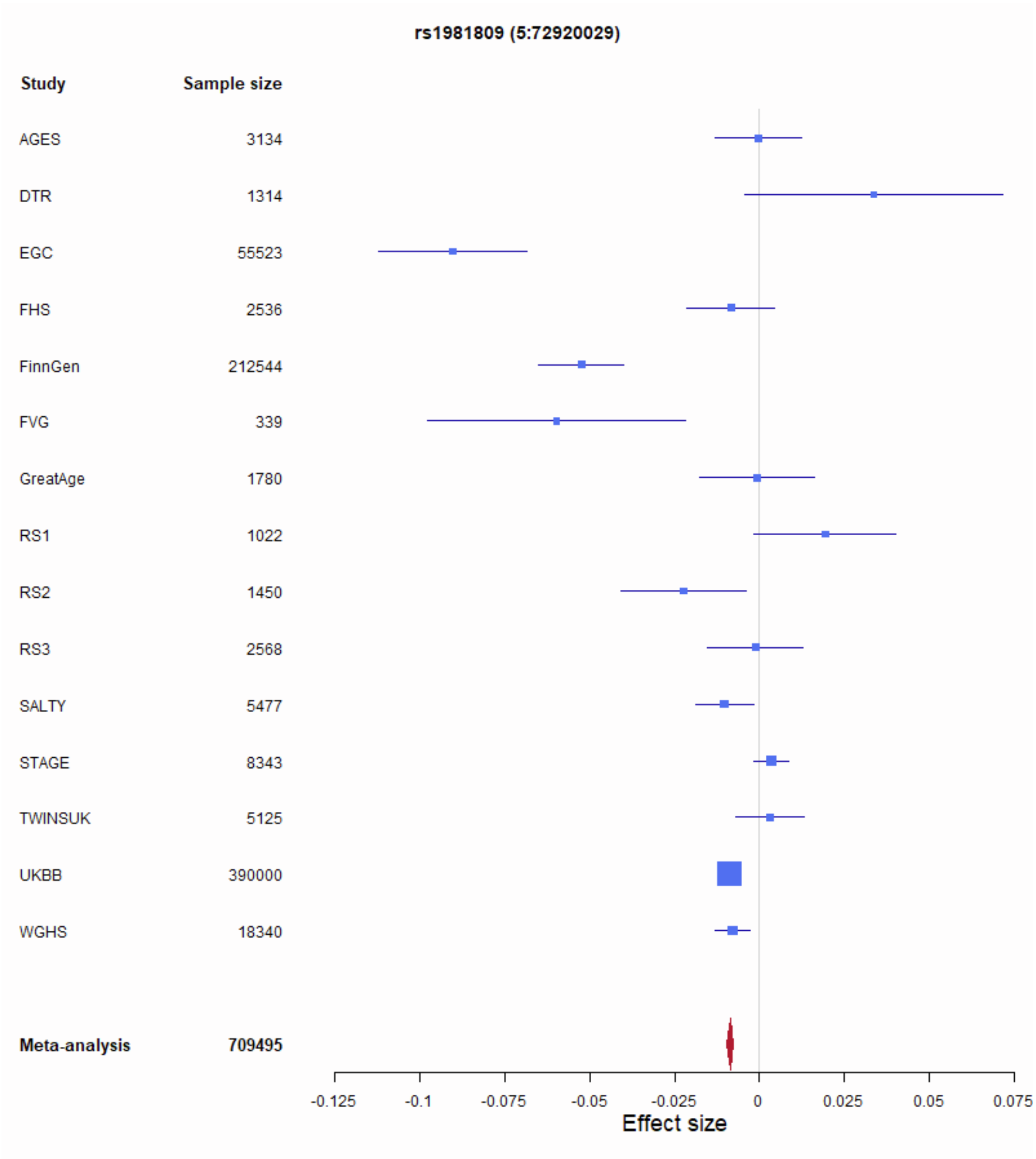

Locus annotation: UTP15-[x]-ARHGEF28

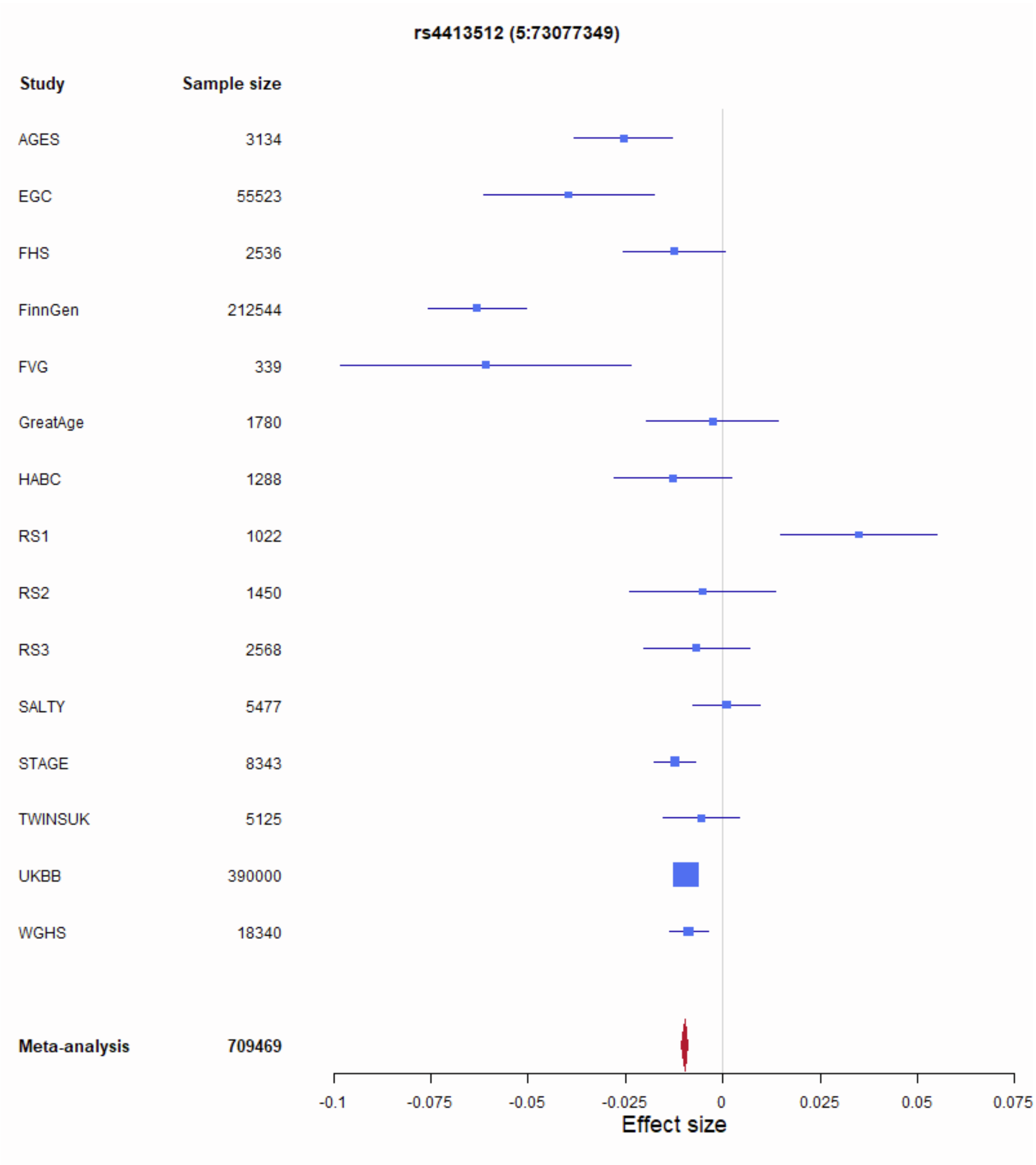

Locus annotation: [ARHGEF28] intronic

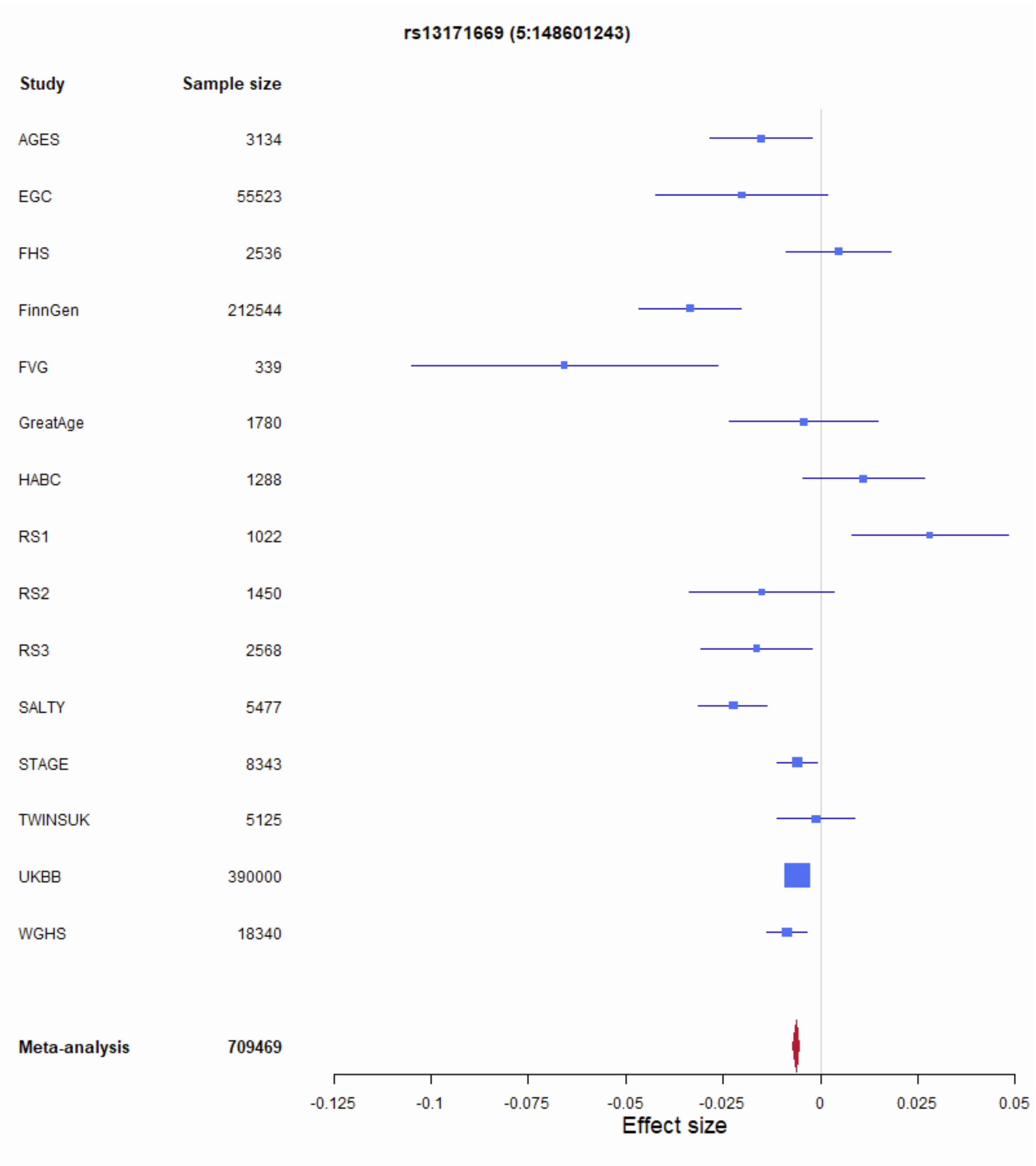

Locus annotation: [ABLIM3] intronic

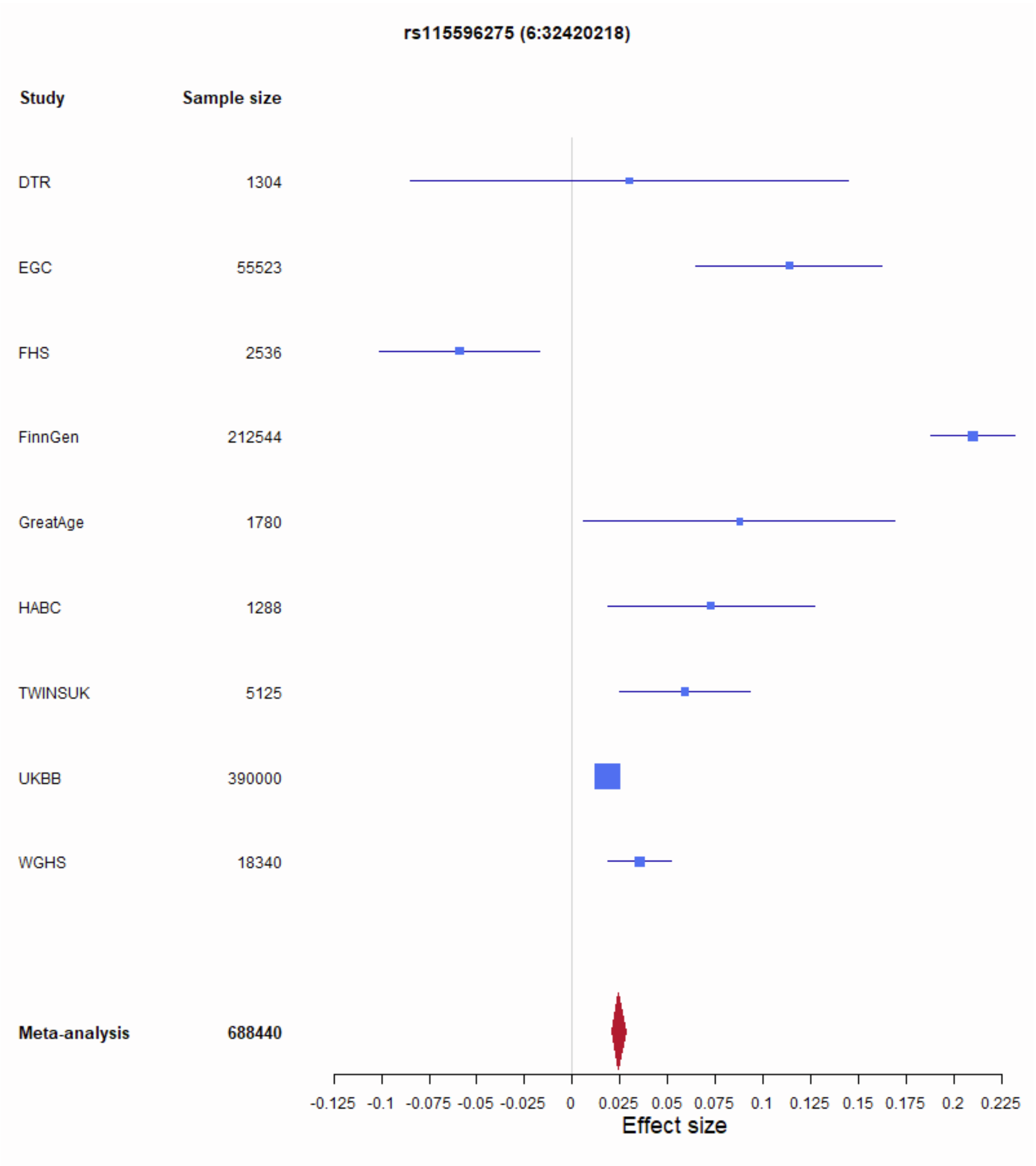

Locus annotation: HLA-DRA-[x]-HLA-DRB5

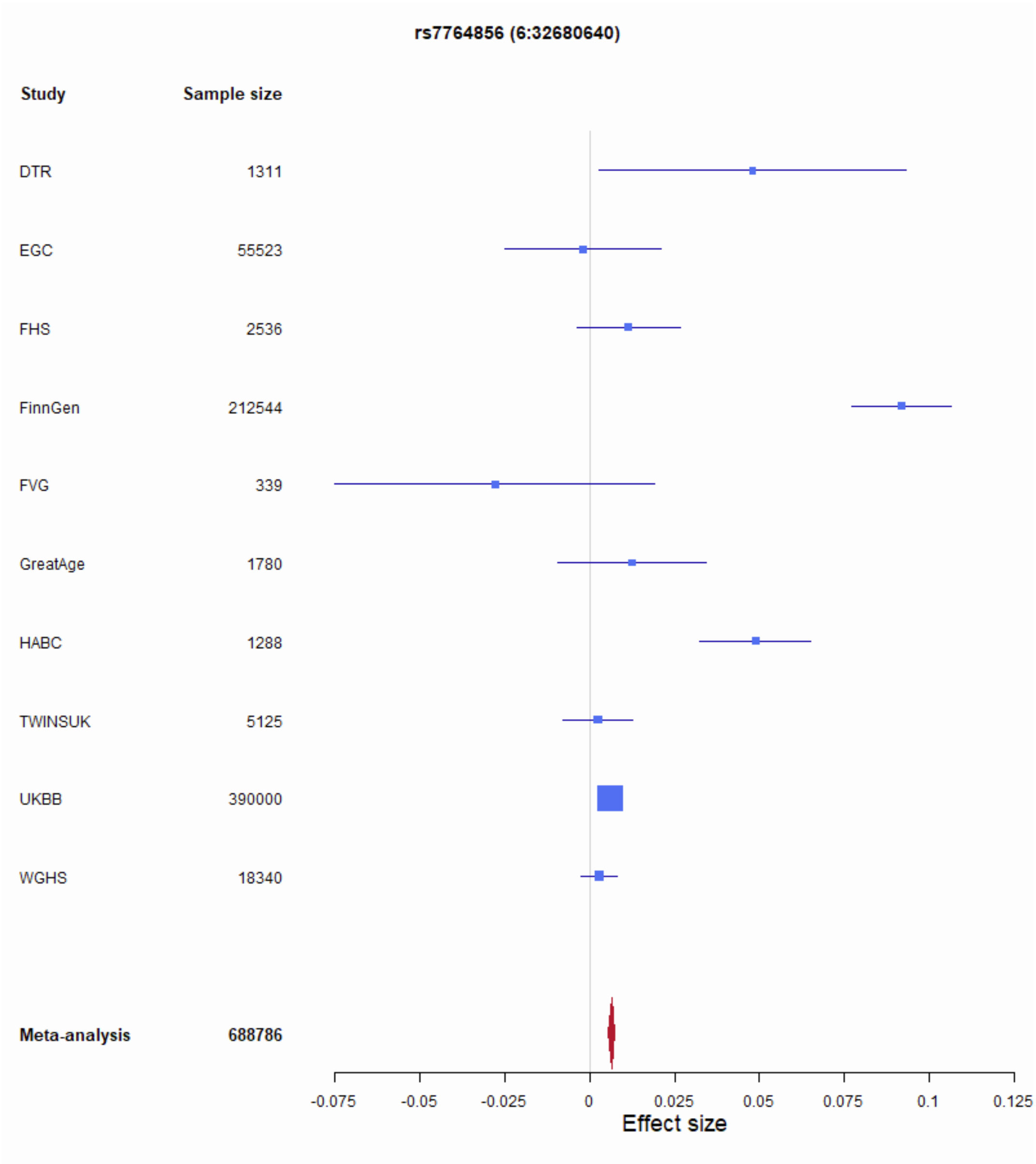

Locus annotation: HLA-DQB1-[x]-HLA-DQA2

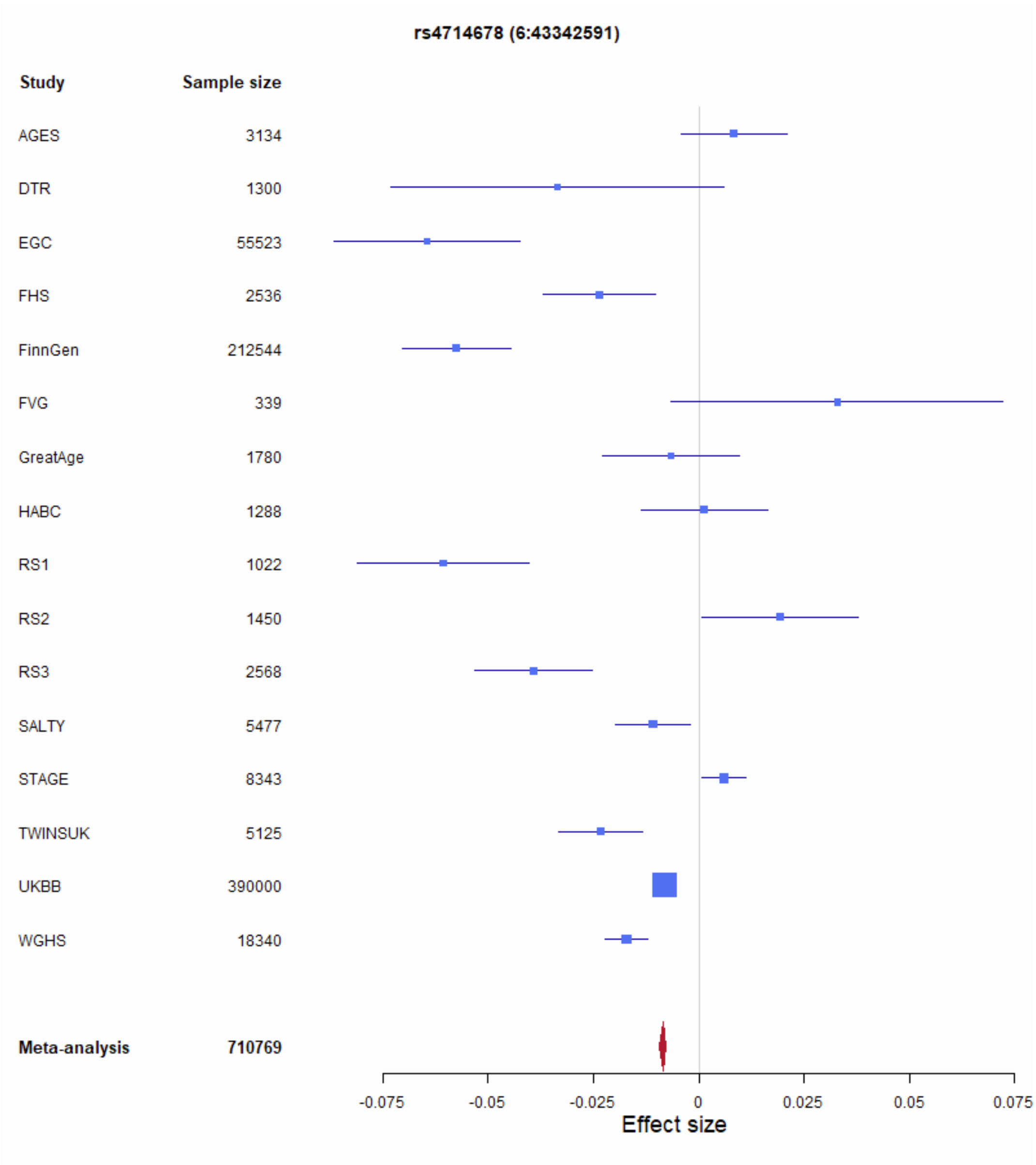

Locus annotation: ZNF318-[x]-ABCC10

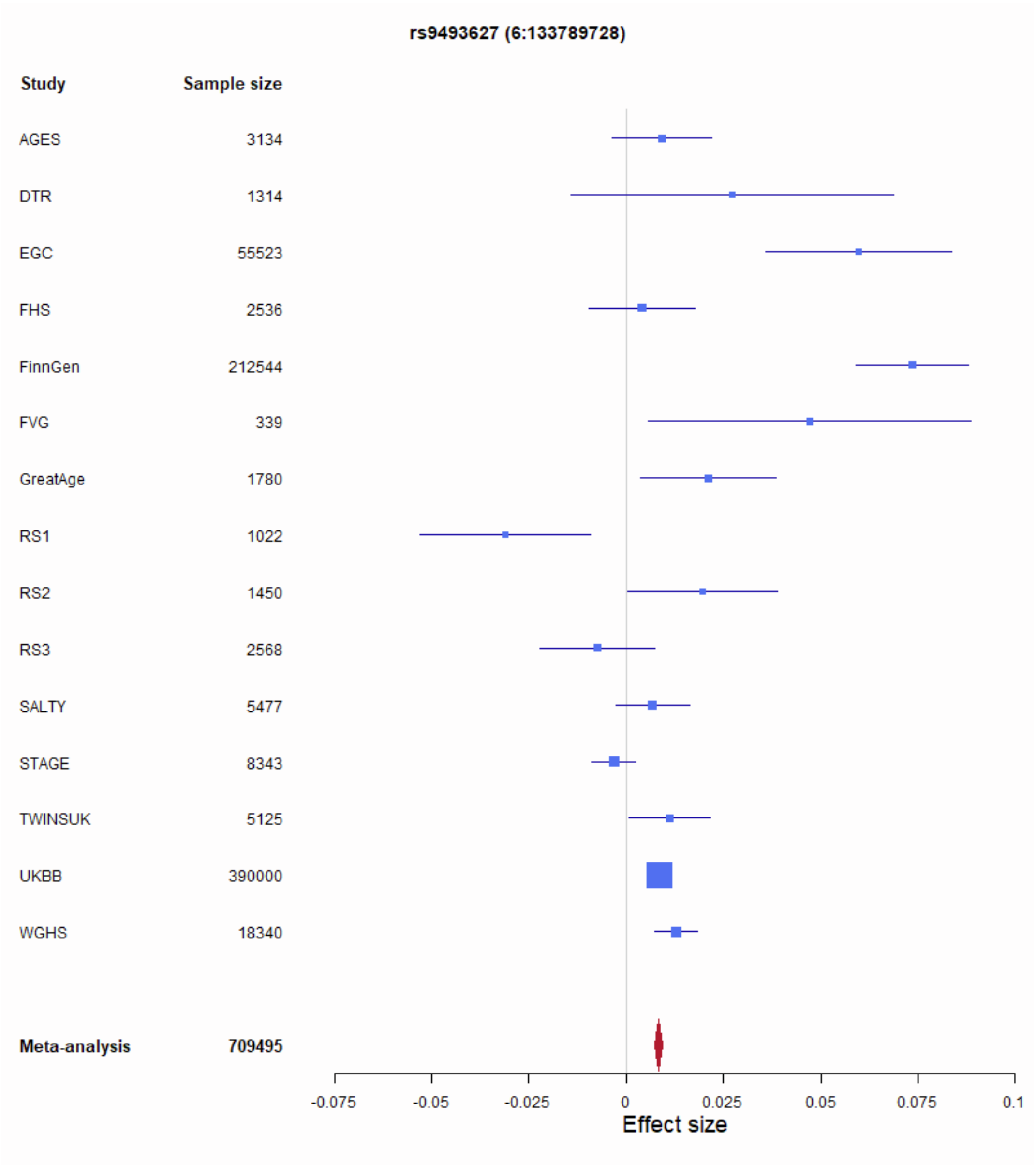

Locus annotation: [EYA4] G>S

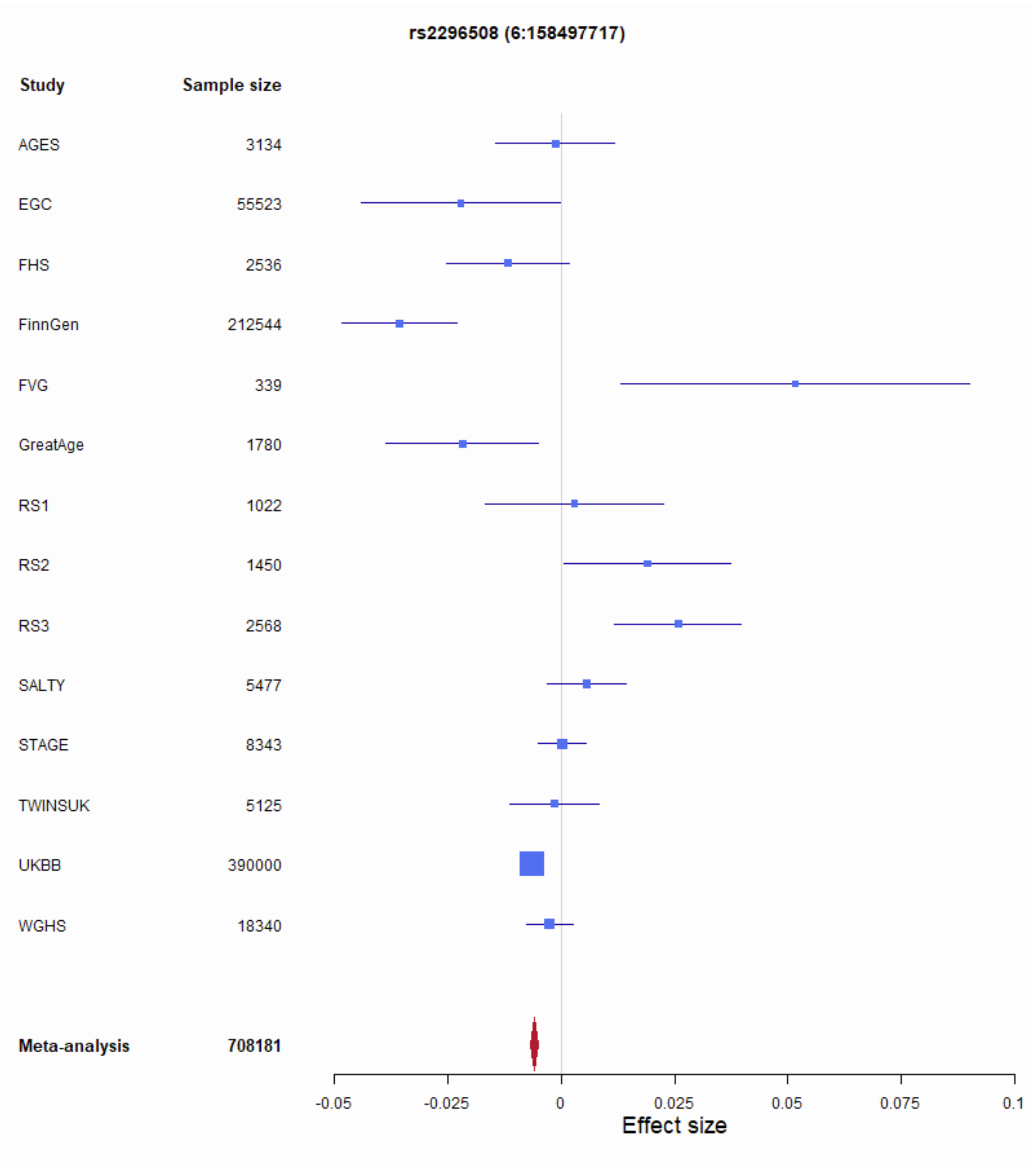

Locus annotation: [SYNJ2] V>V

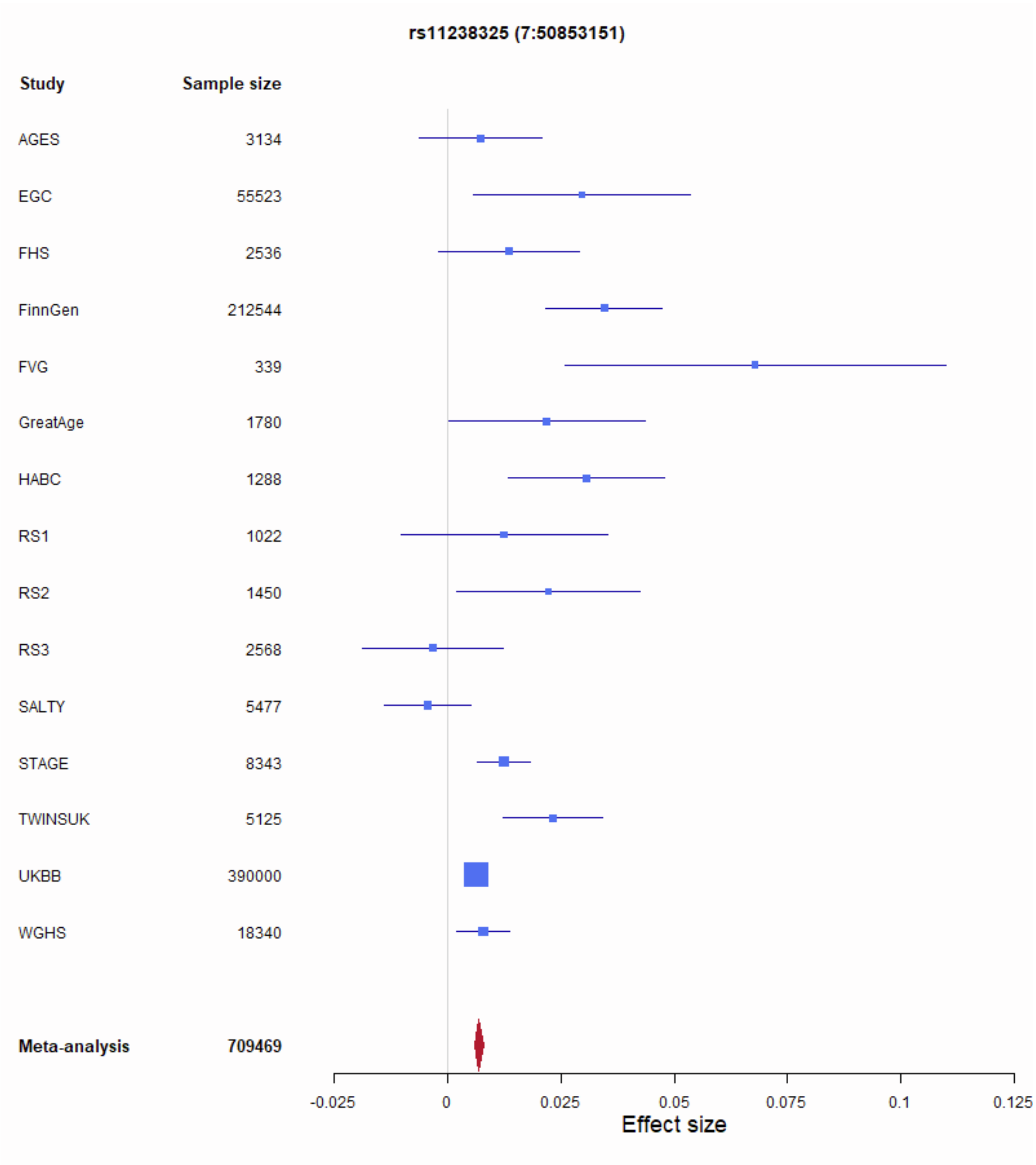

Locus annotation: [GRB10] intronic

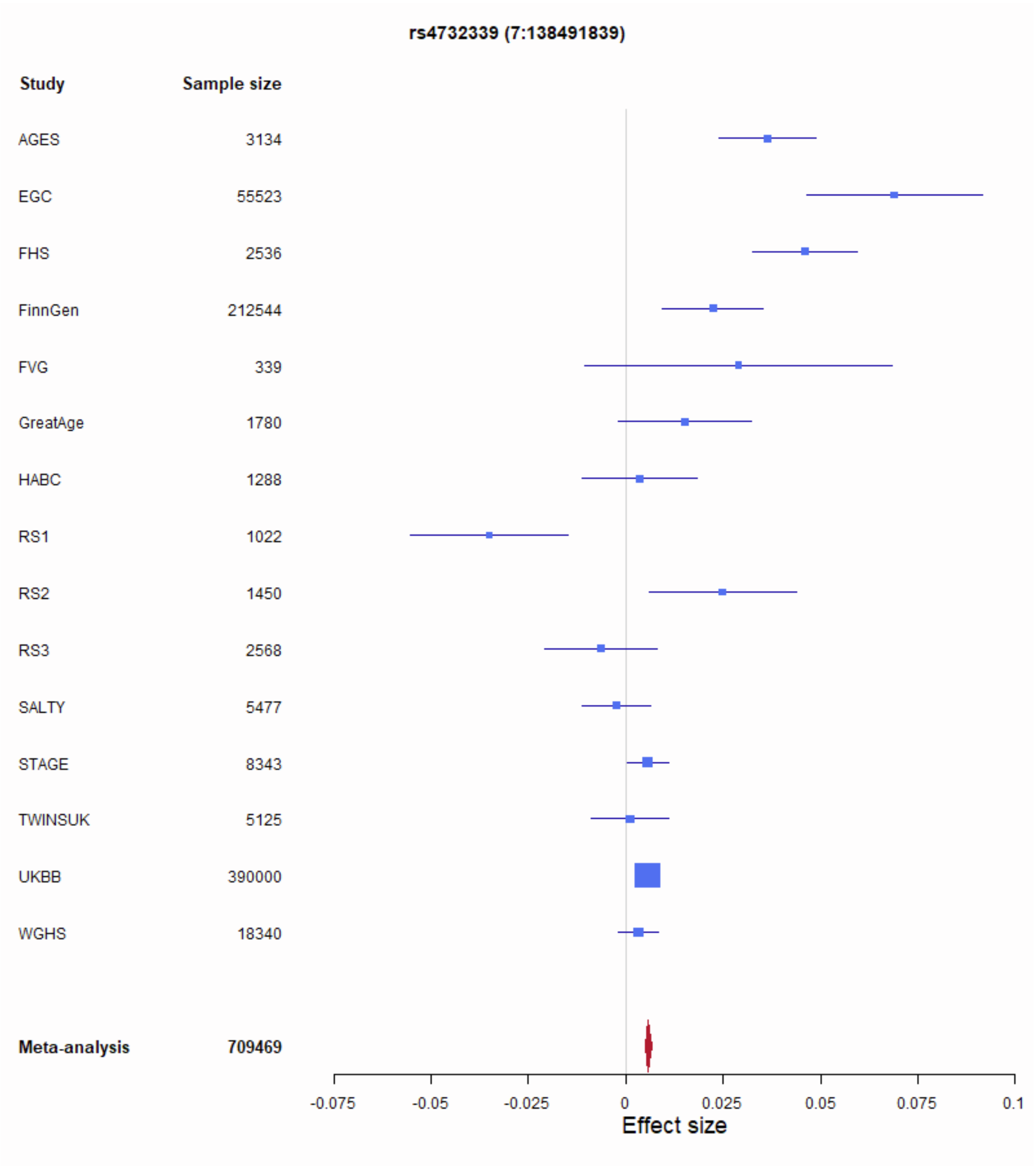

Locus annotation: TMEM213-[x]-KIAA1549

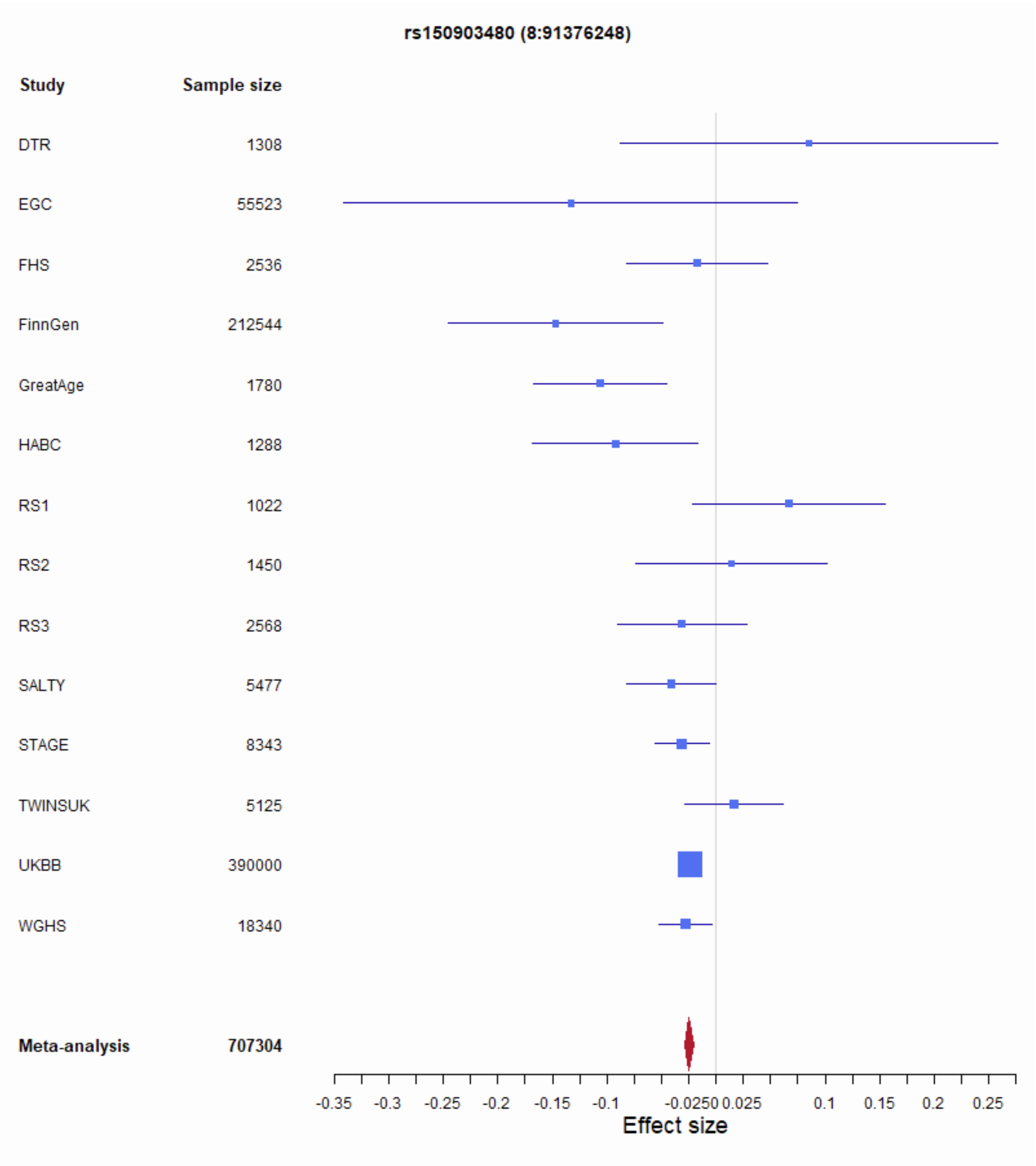

Locus annotation: [LINC00534]

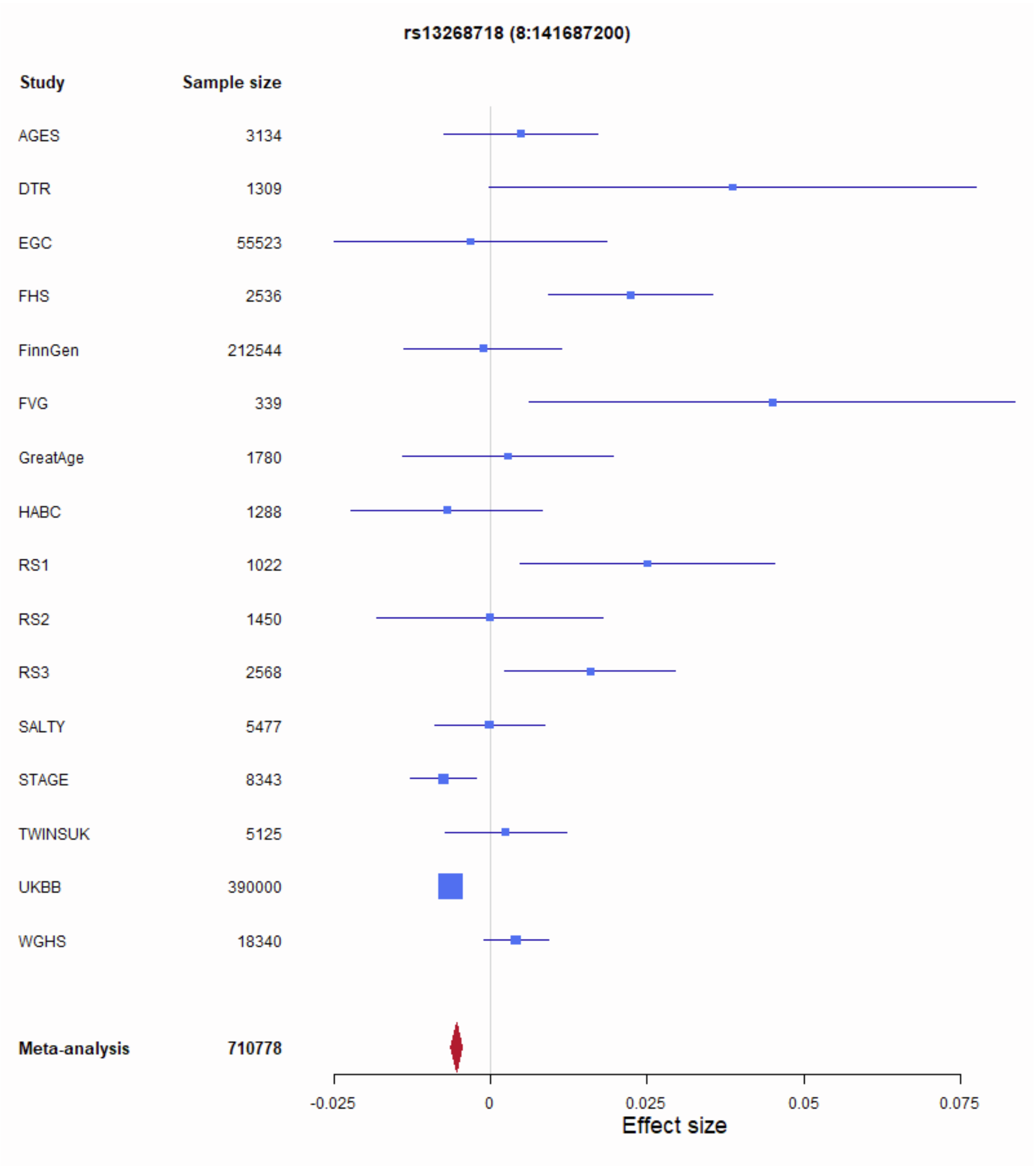

Locus annotation: [PTK2] intronic

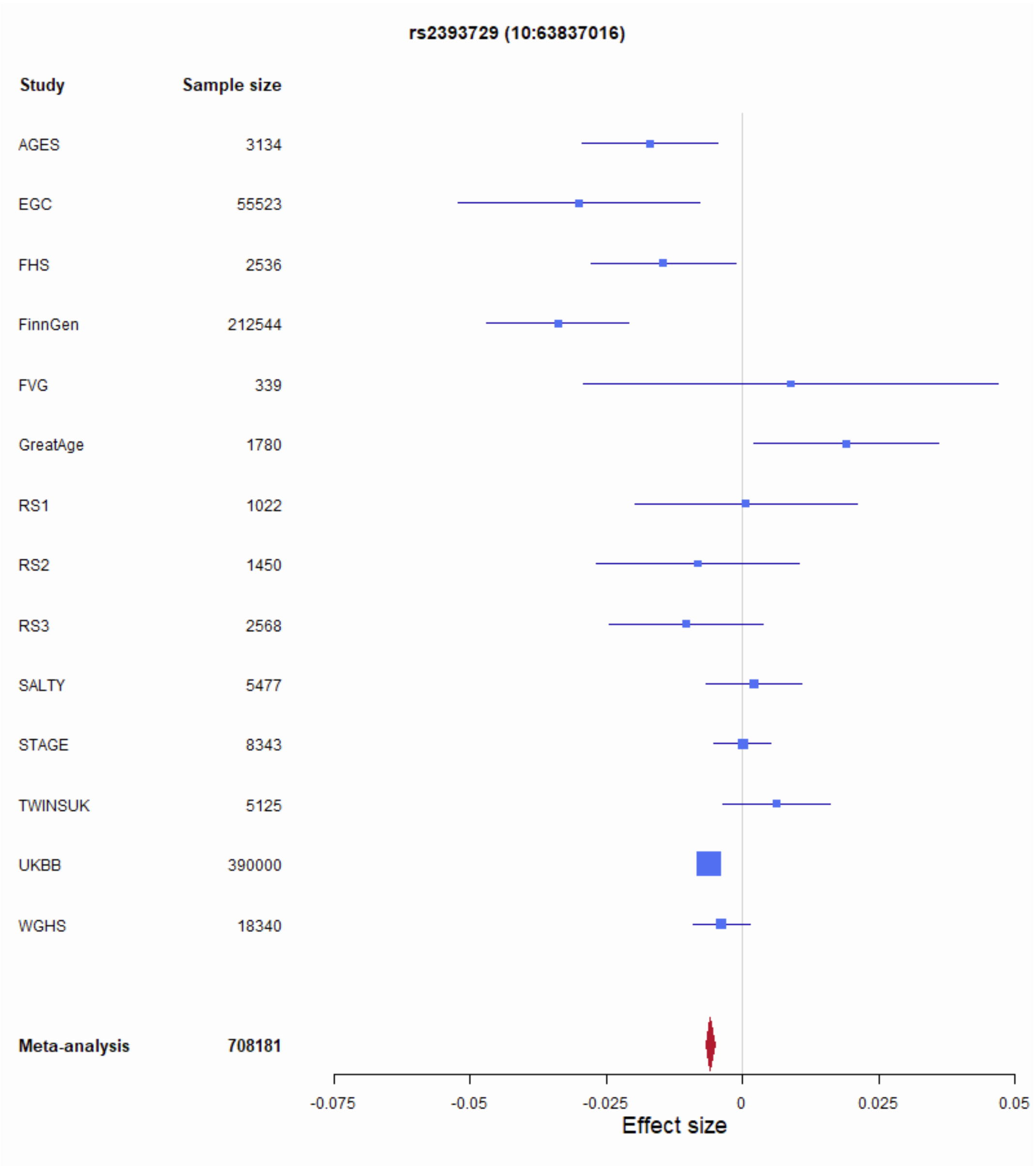

Locus annotation: [ARID5B] intronic

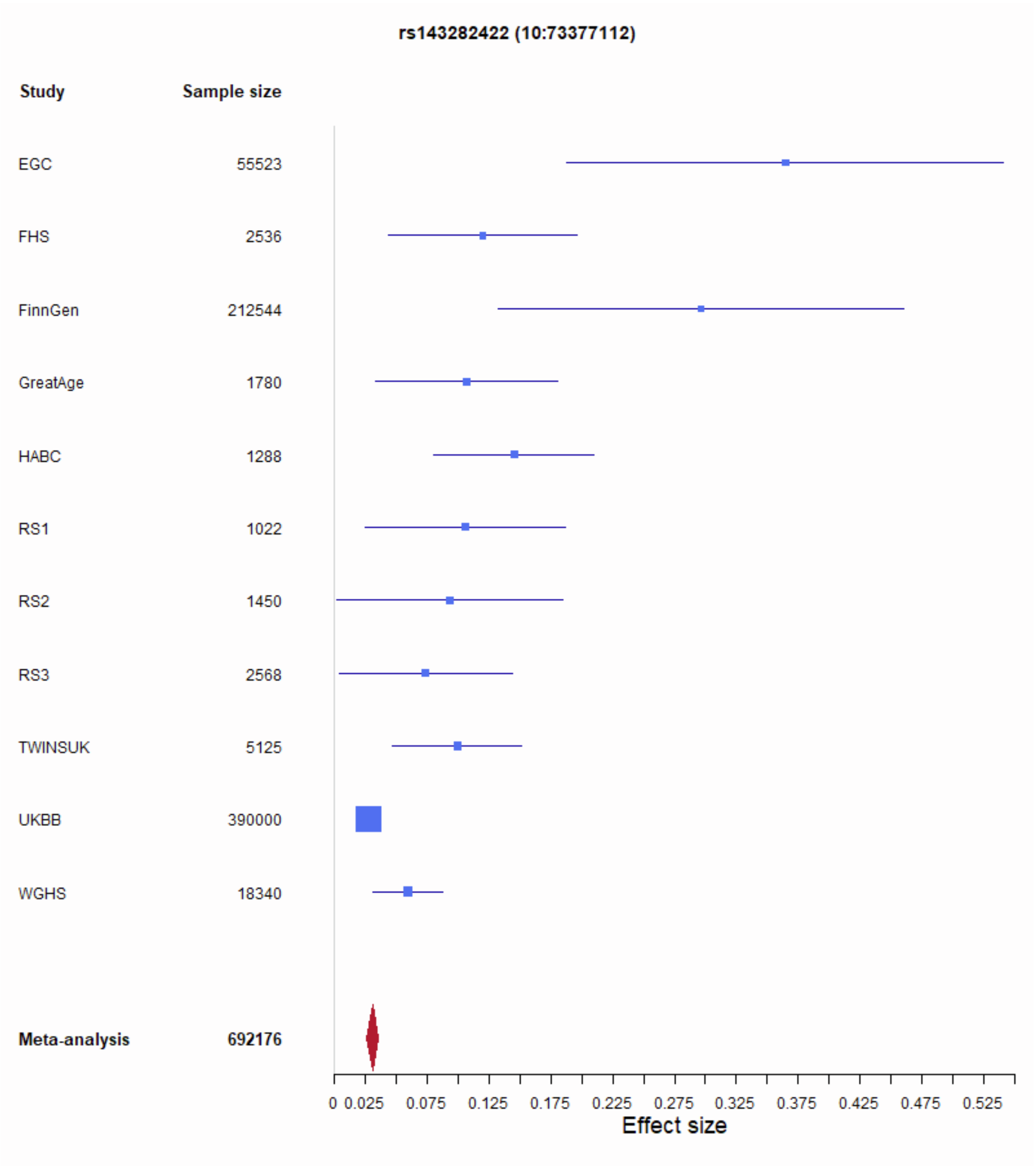

Locus annotation: [CDH23] A>T

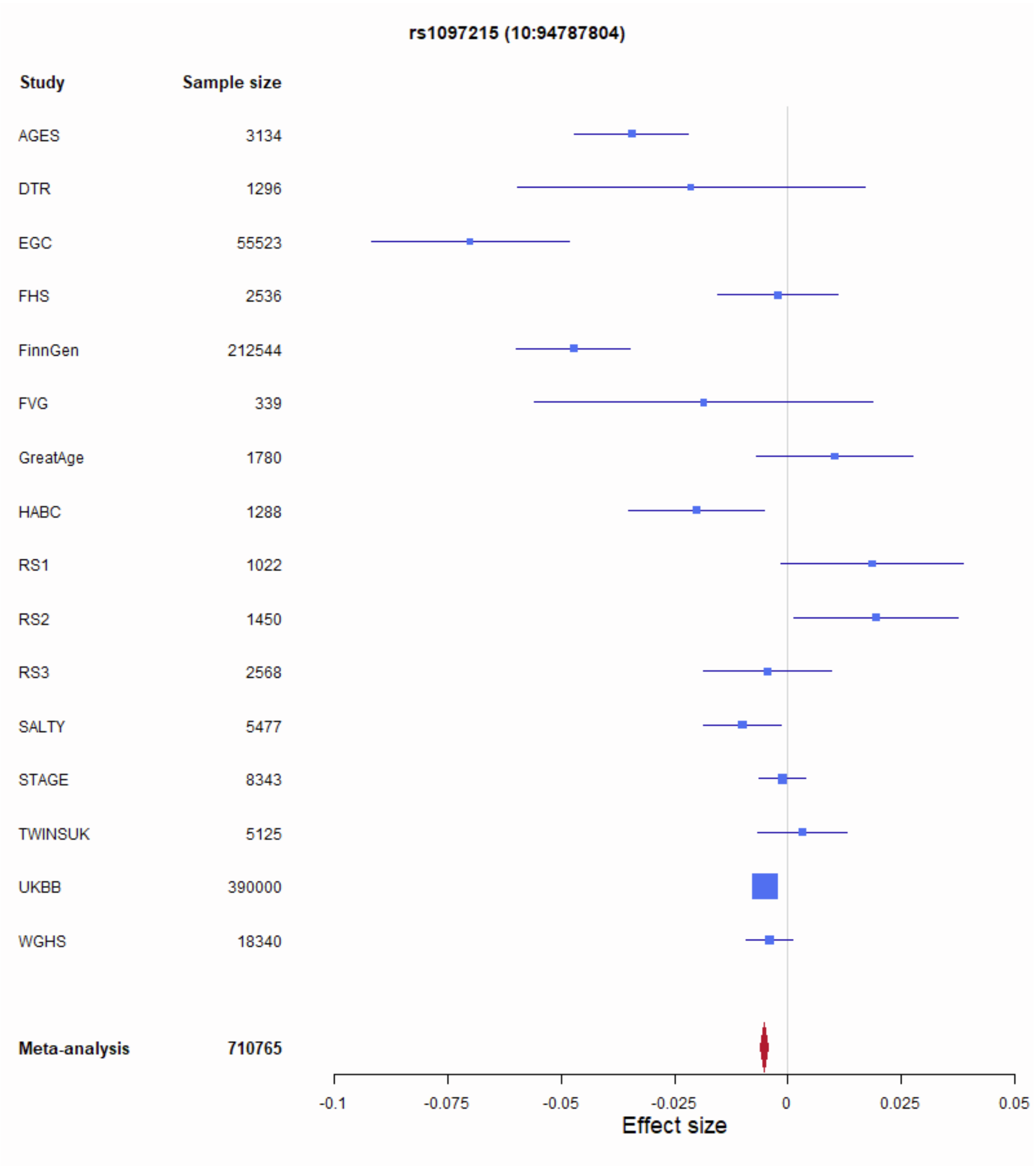

Locus annotation: [EXOC6] intronic

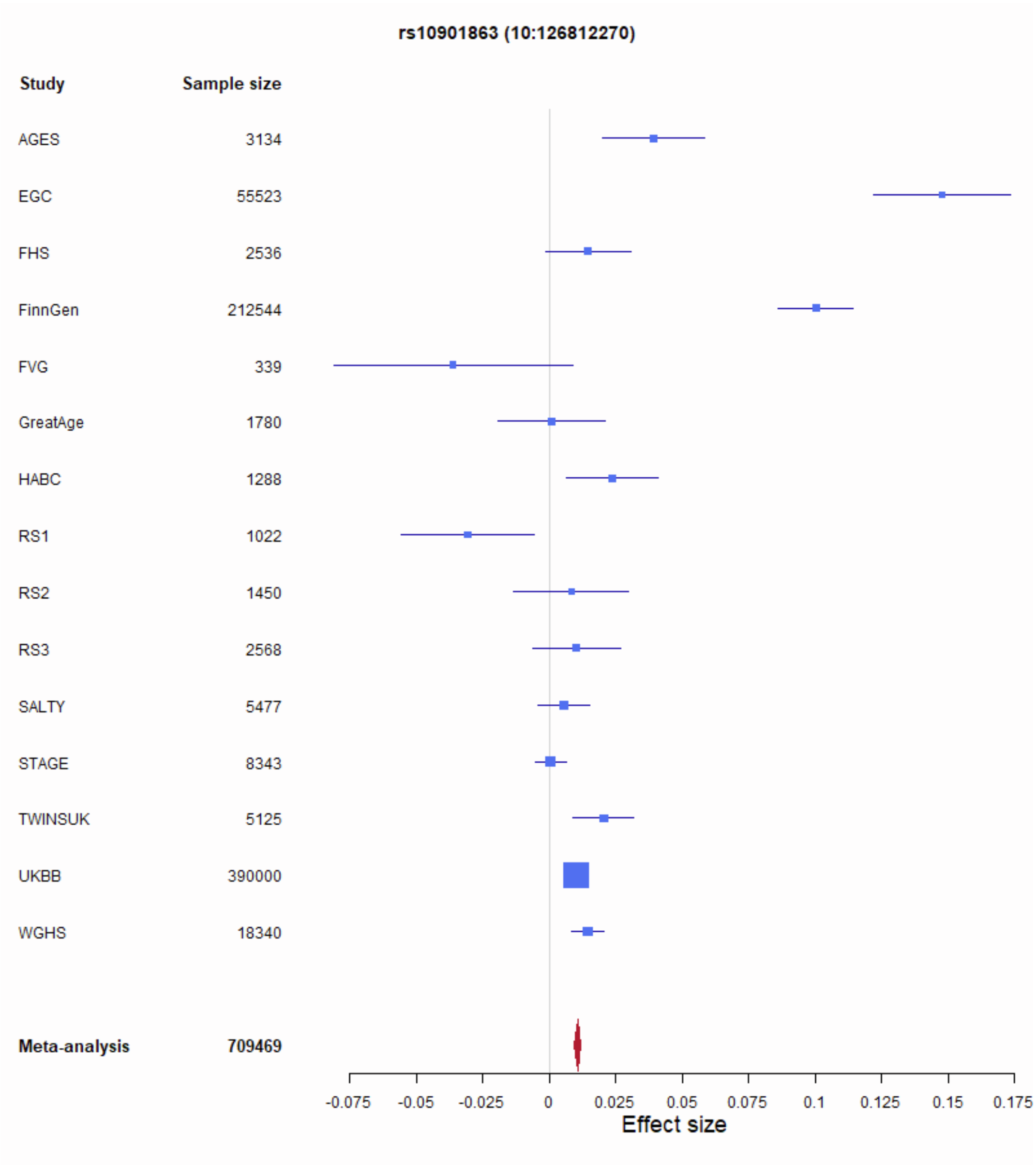

Locus annotation: [CTBP2] 5' UTR

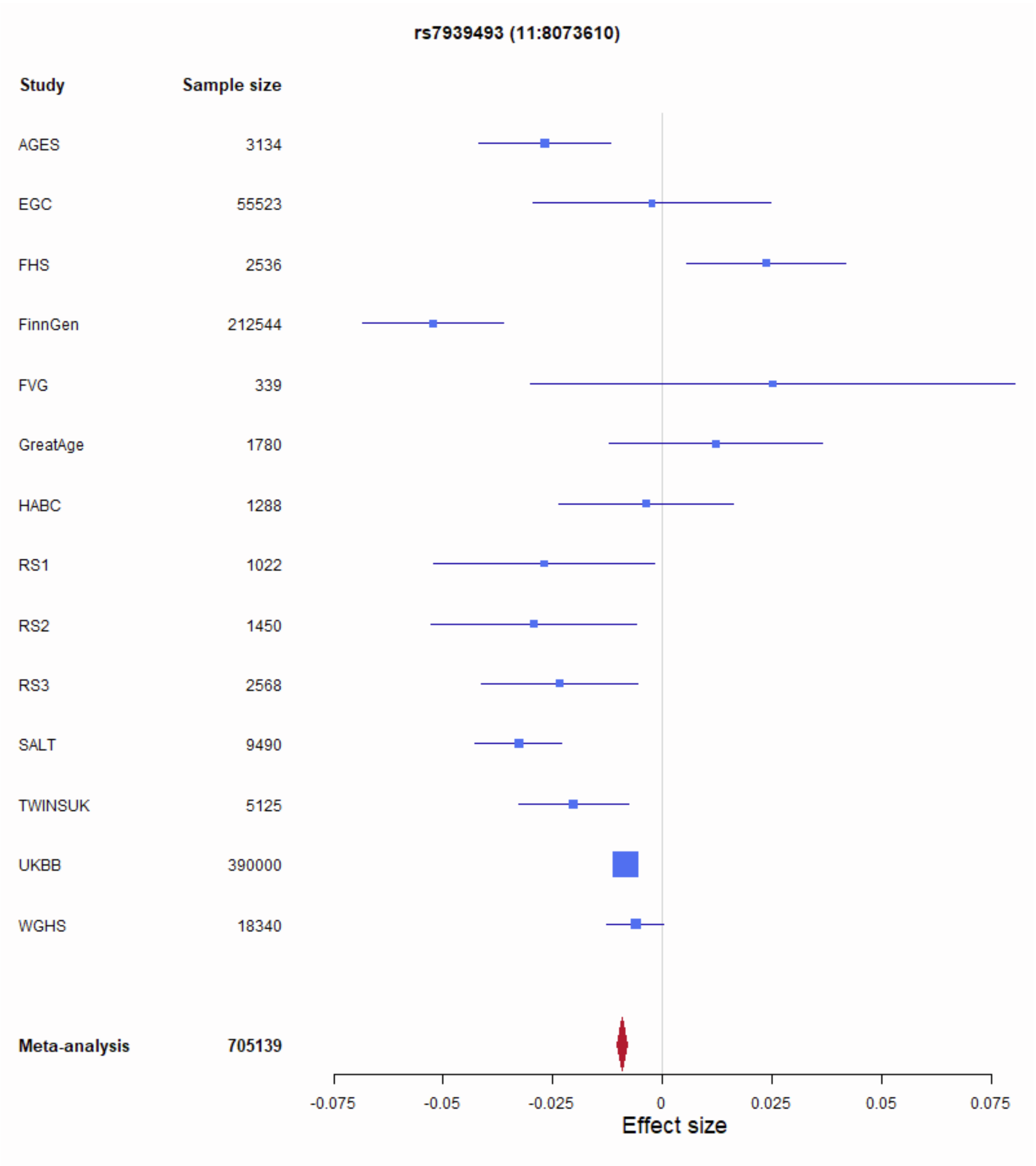

Locus annotation: [TUB] intronic

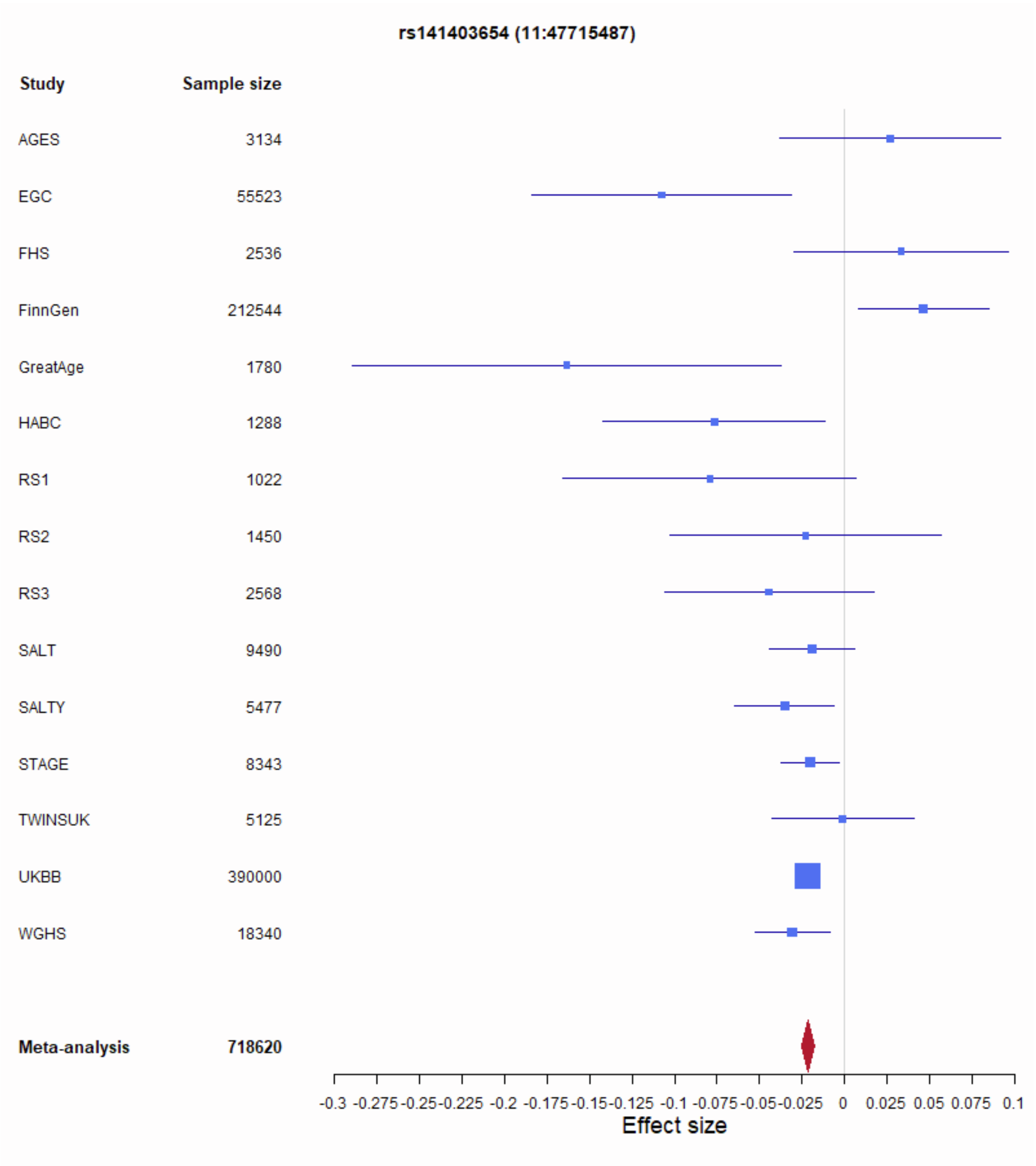

Locus annotation: [AGBL2] intronic

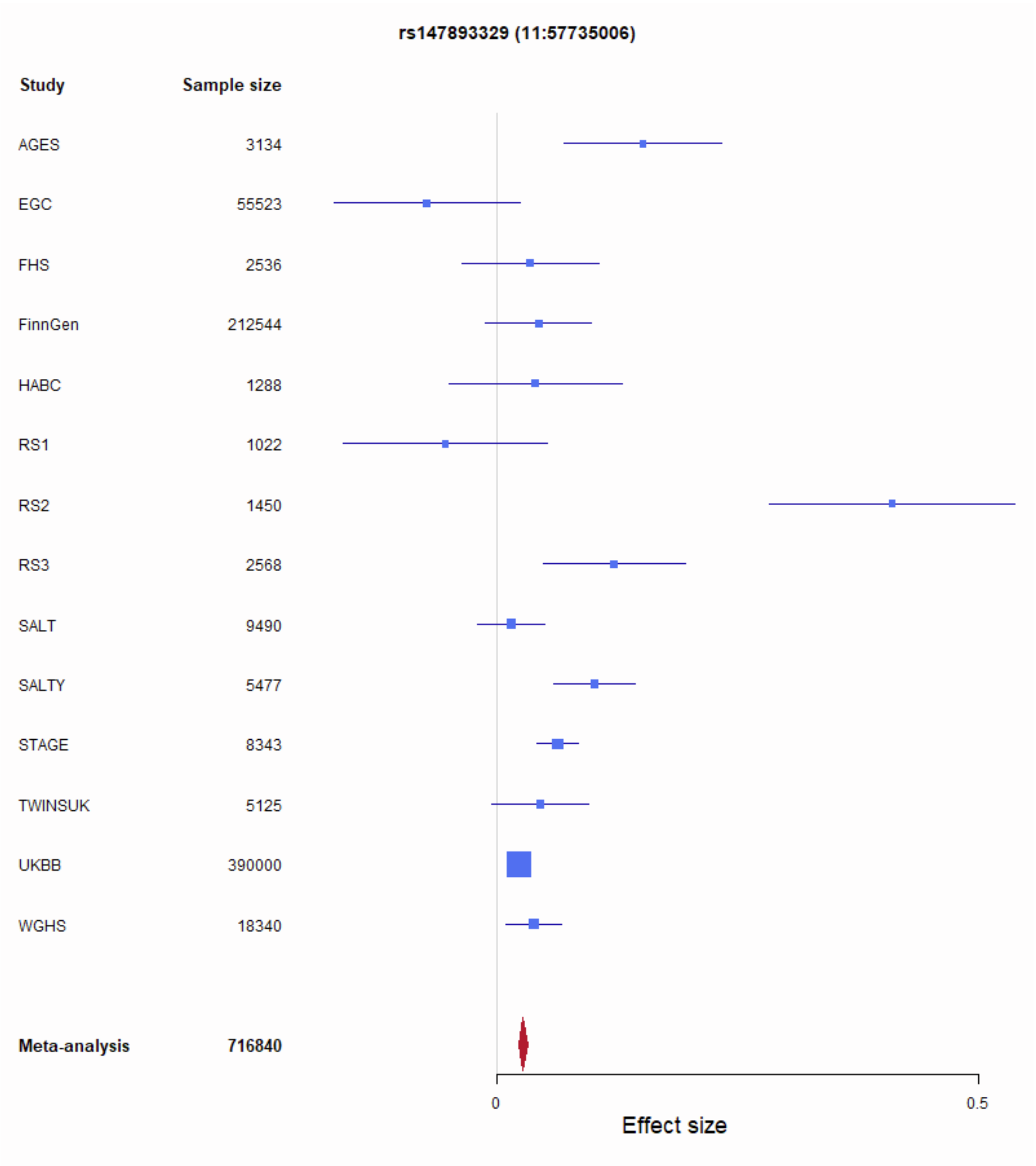

Locus annotation: CTNND1--[x]-OR9Q1

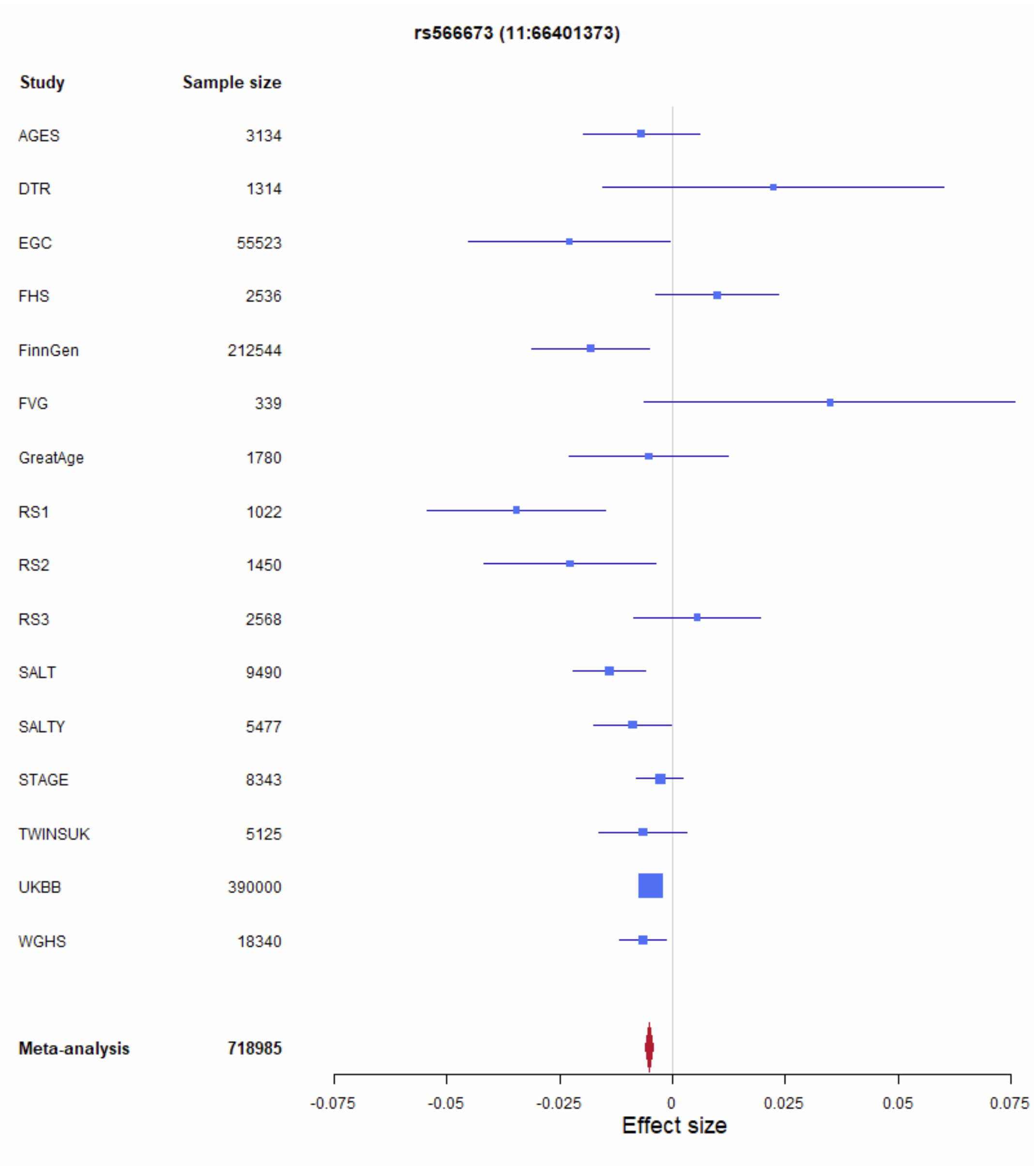

Locus annotation: RBM14-[x]-RBM4

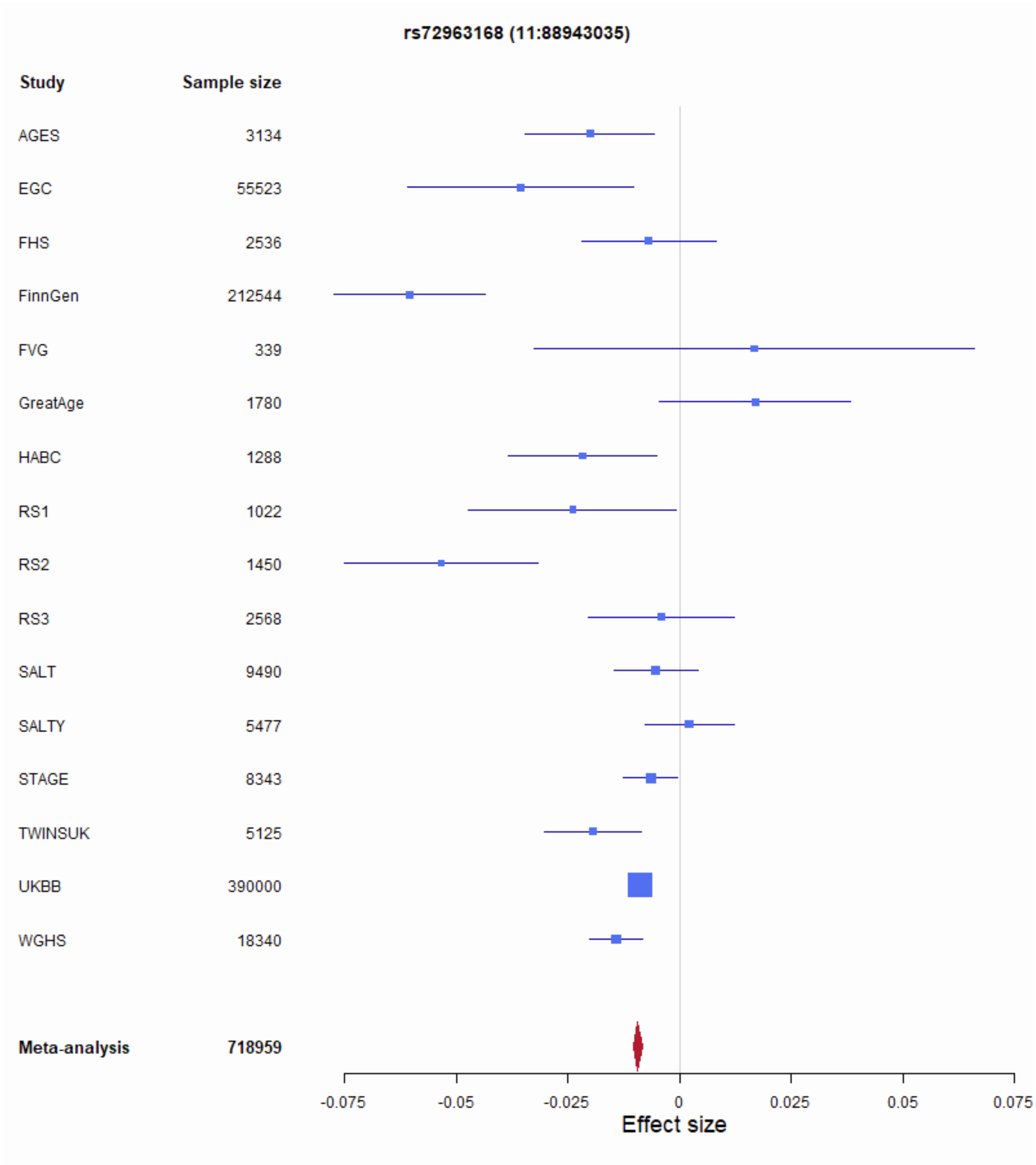

Locus annotation: [TYR] intronic

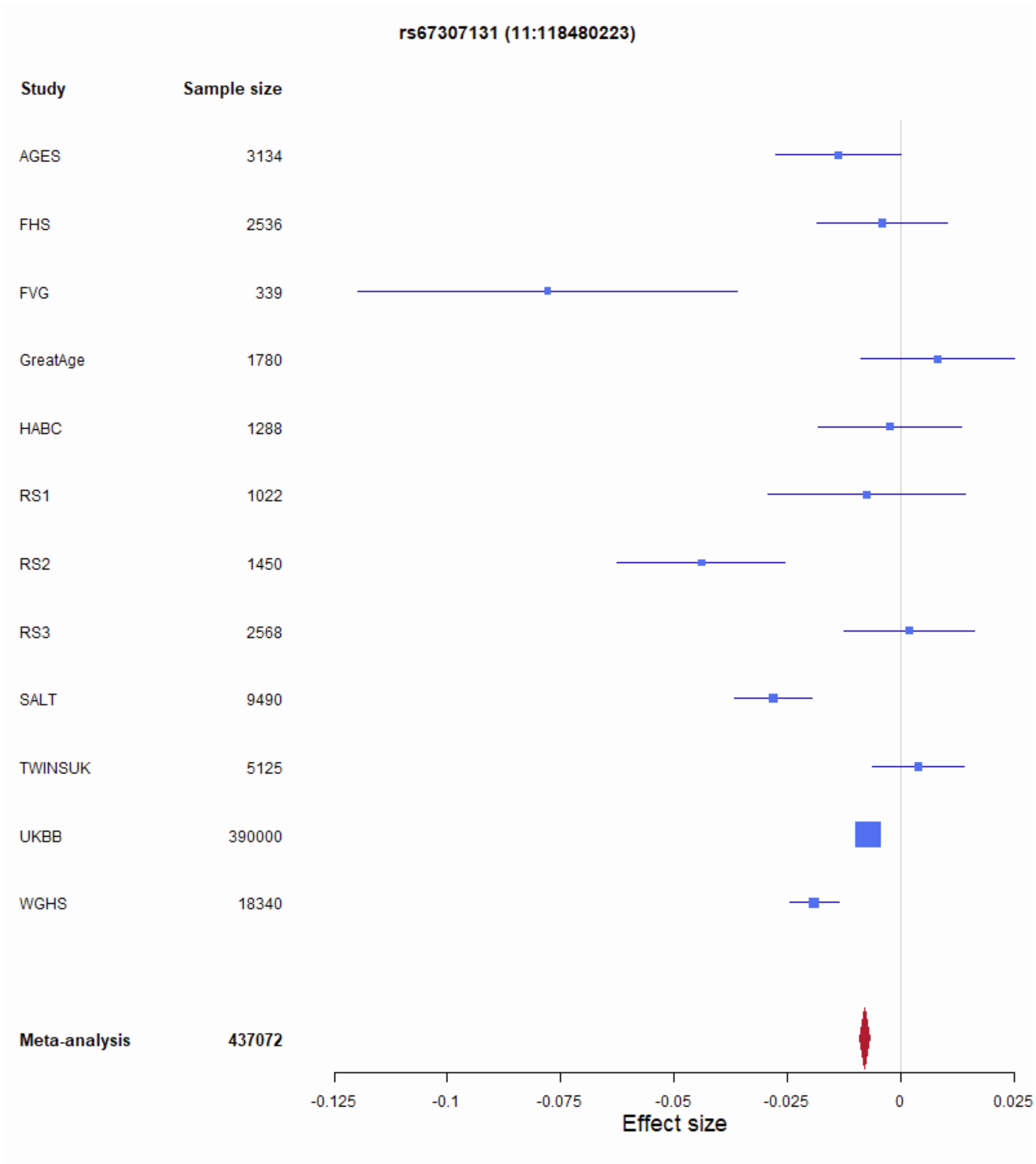

Locus annotation: [PHLDB1] intronic

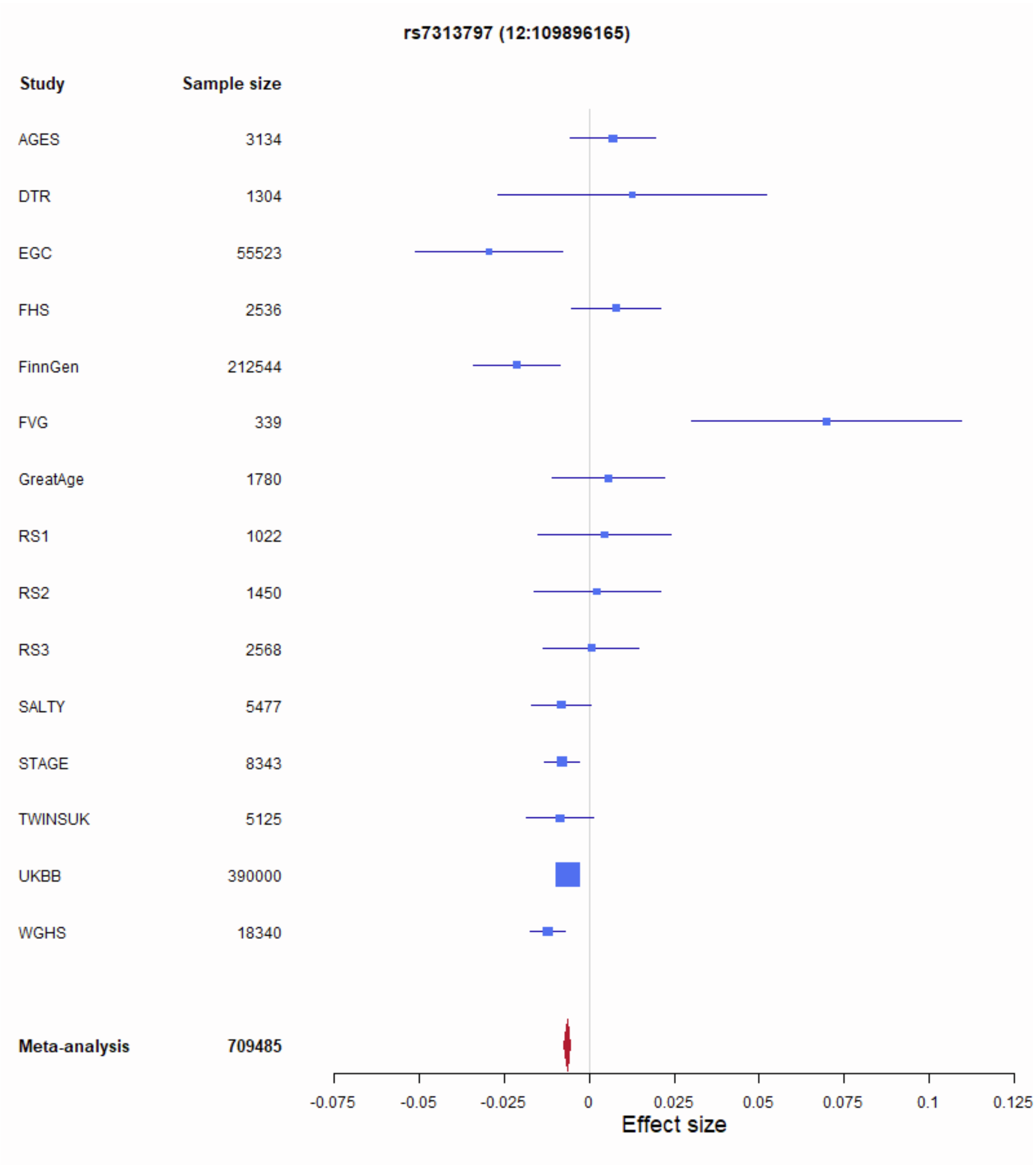

Locus annotation: [KCTD10] intronic

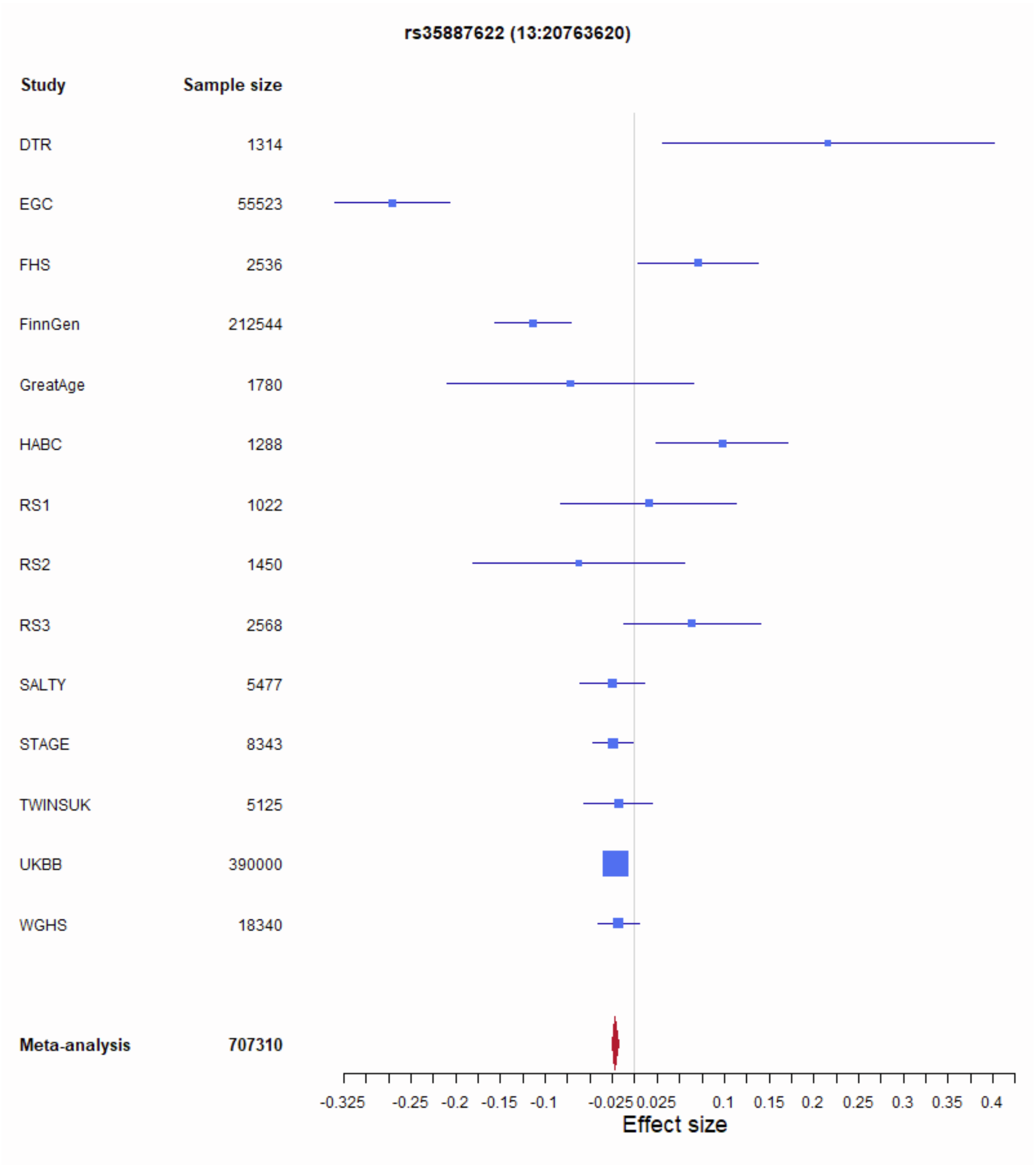

Locus annotation: [GJB2] M>T

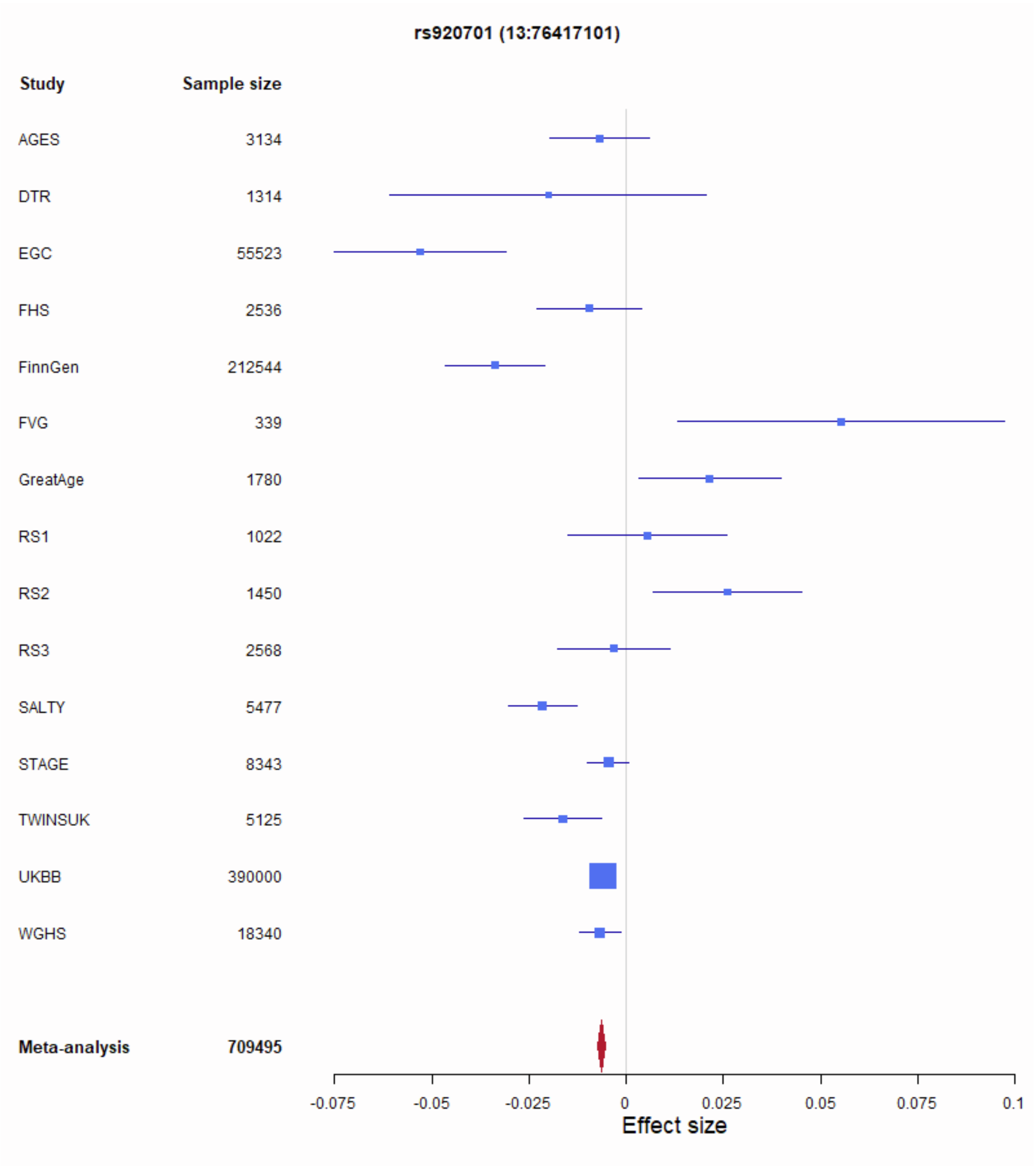

Locus annotation: [LMO7] intronic

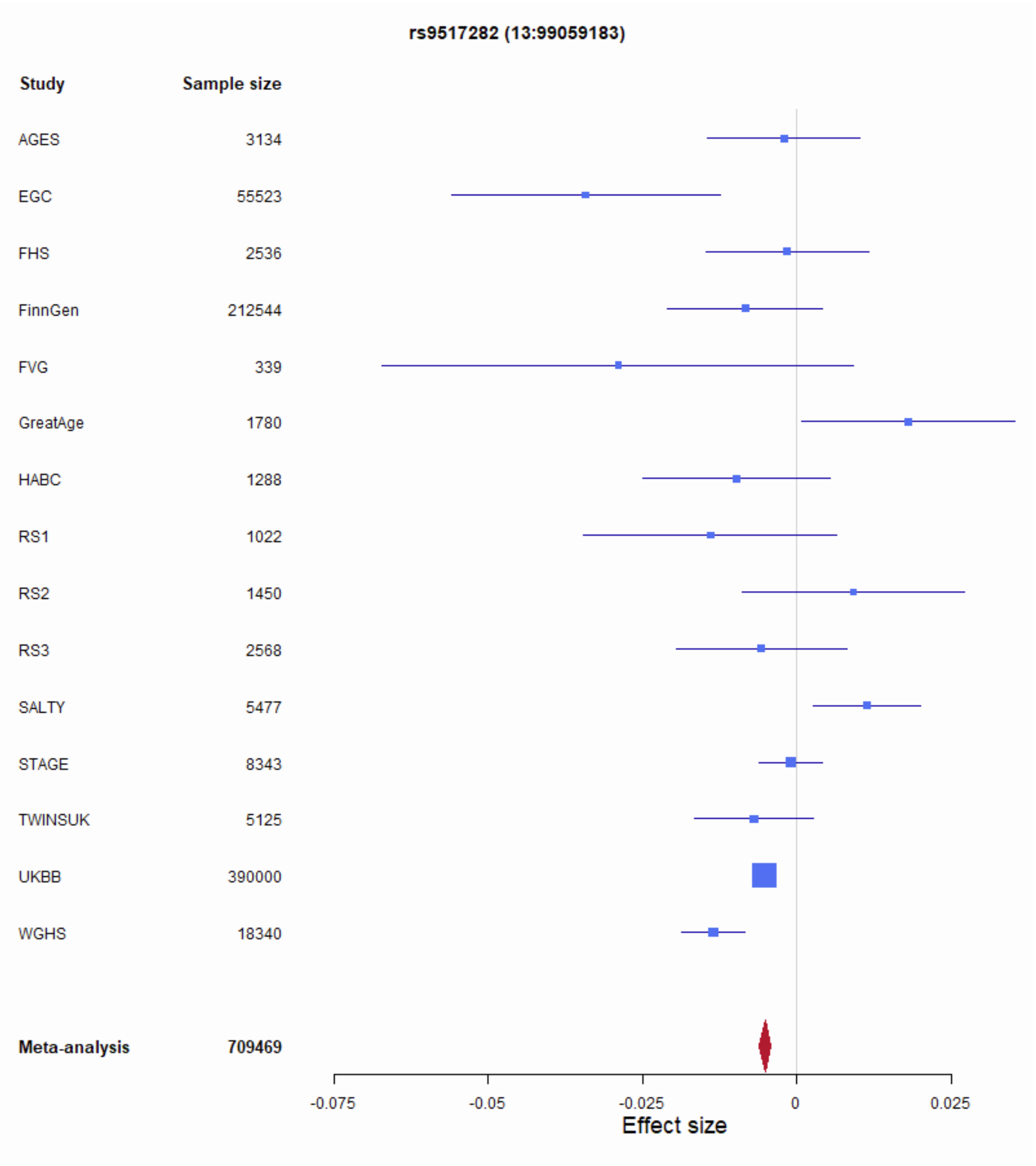

Locus annotation: [FARP1] intronic

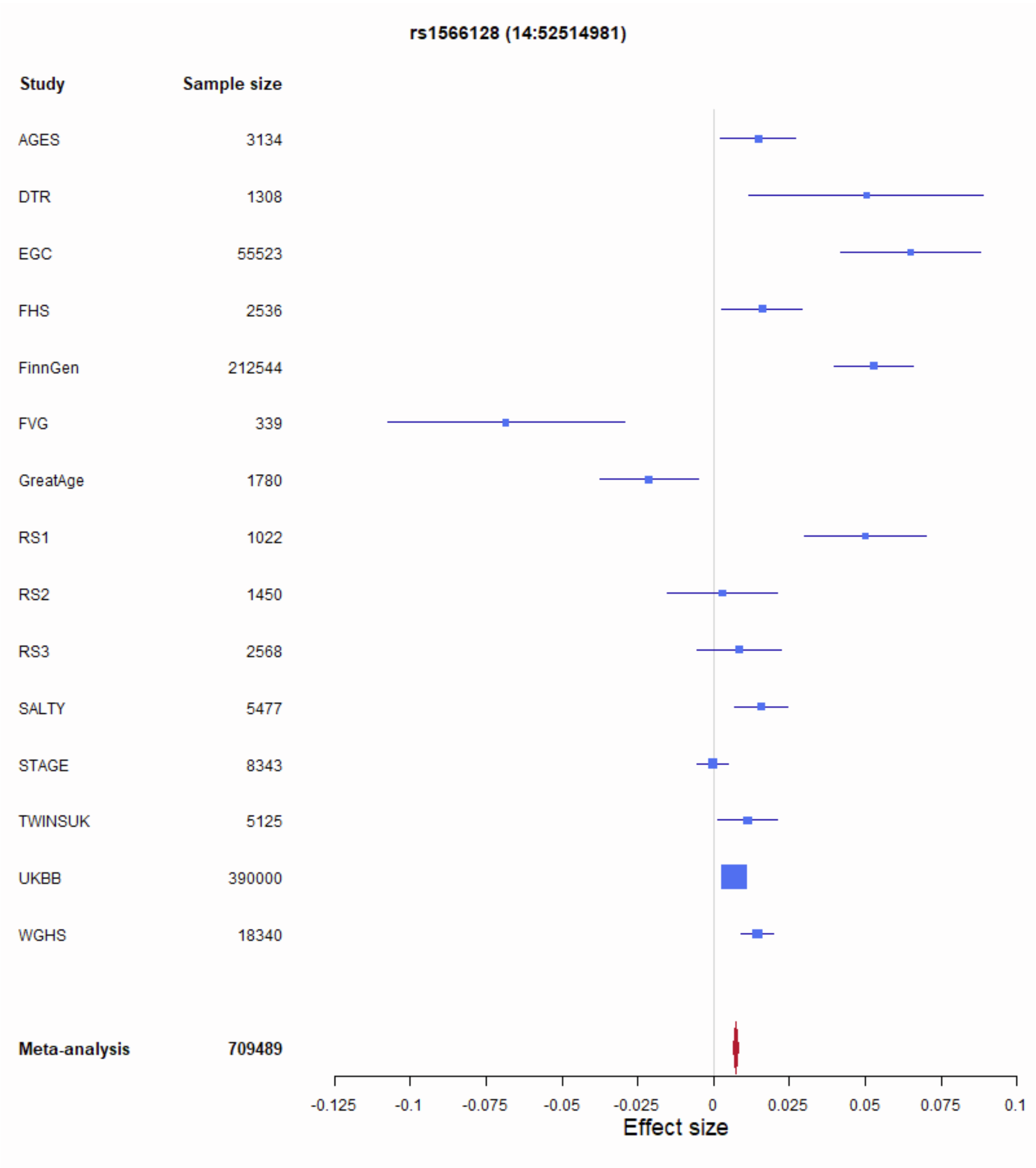

Locus annotation: [NID2] intronic

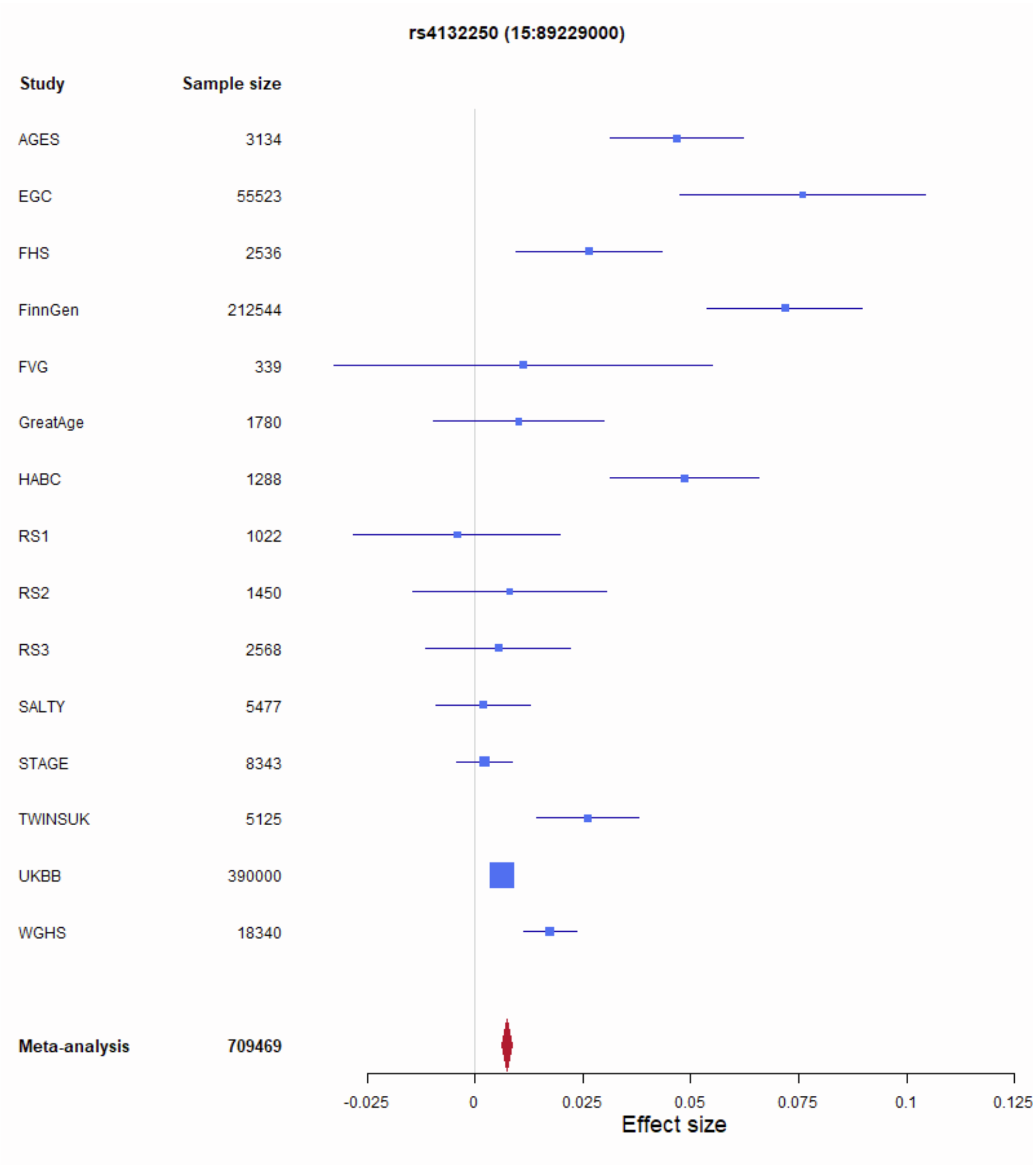

Locus annotation: ISG20-[x]--ACAN

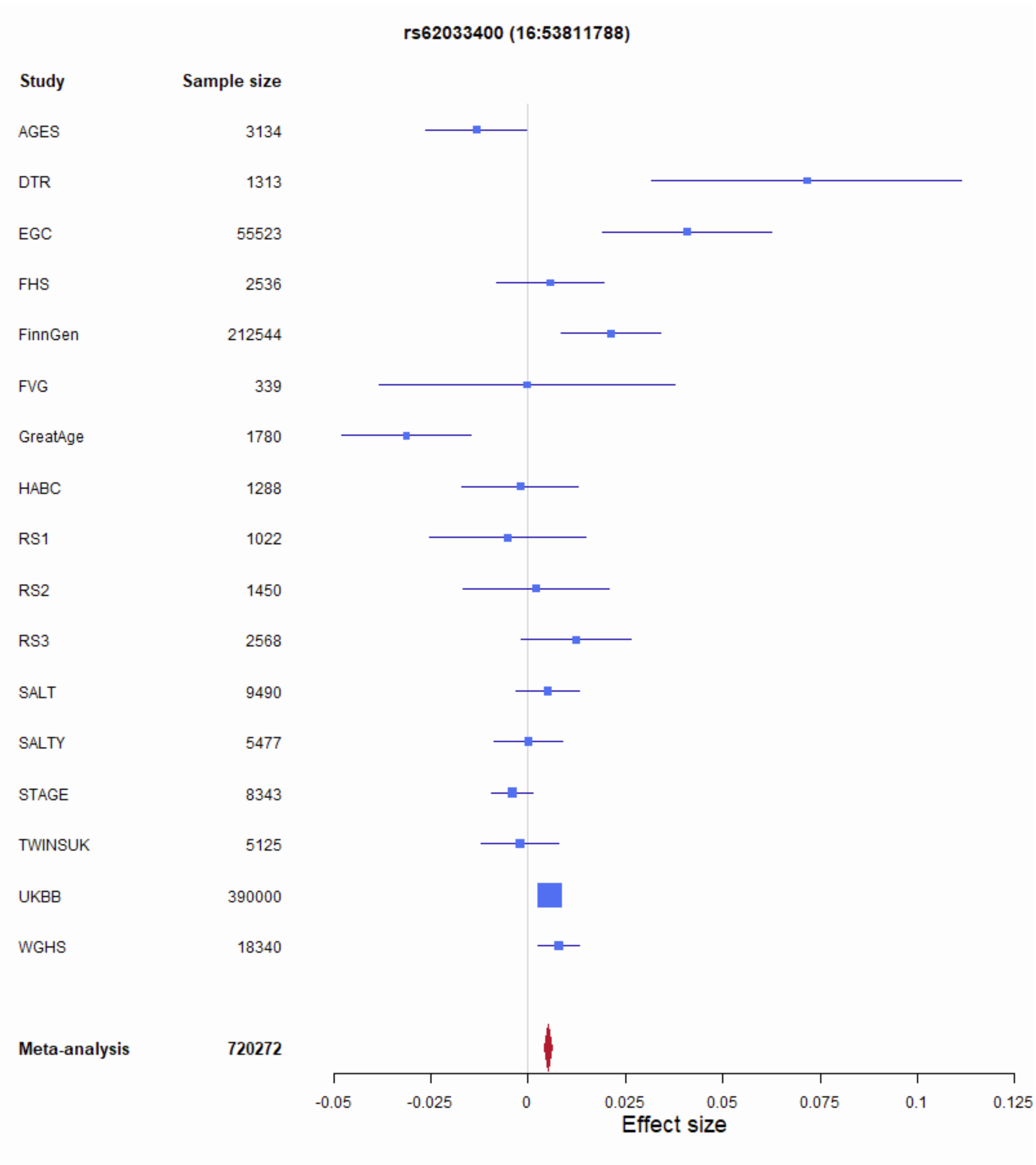

Locus annotation: [FTO] intronic

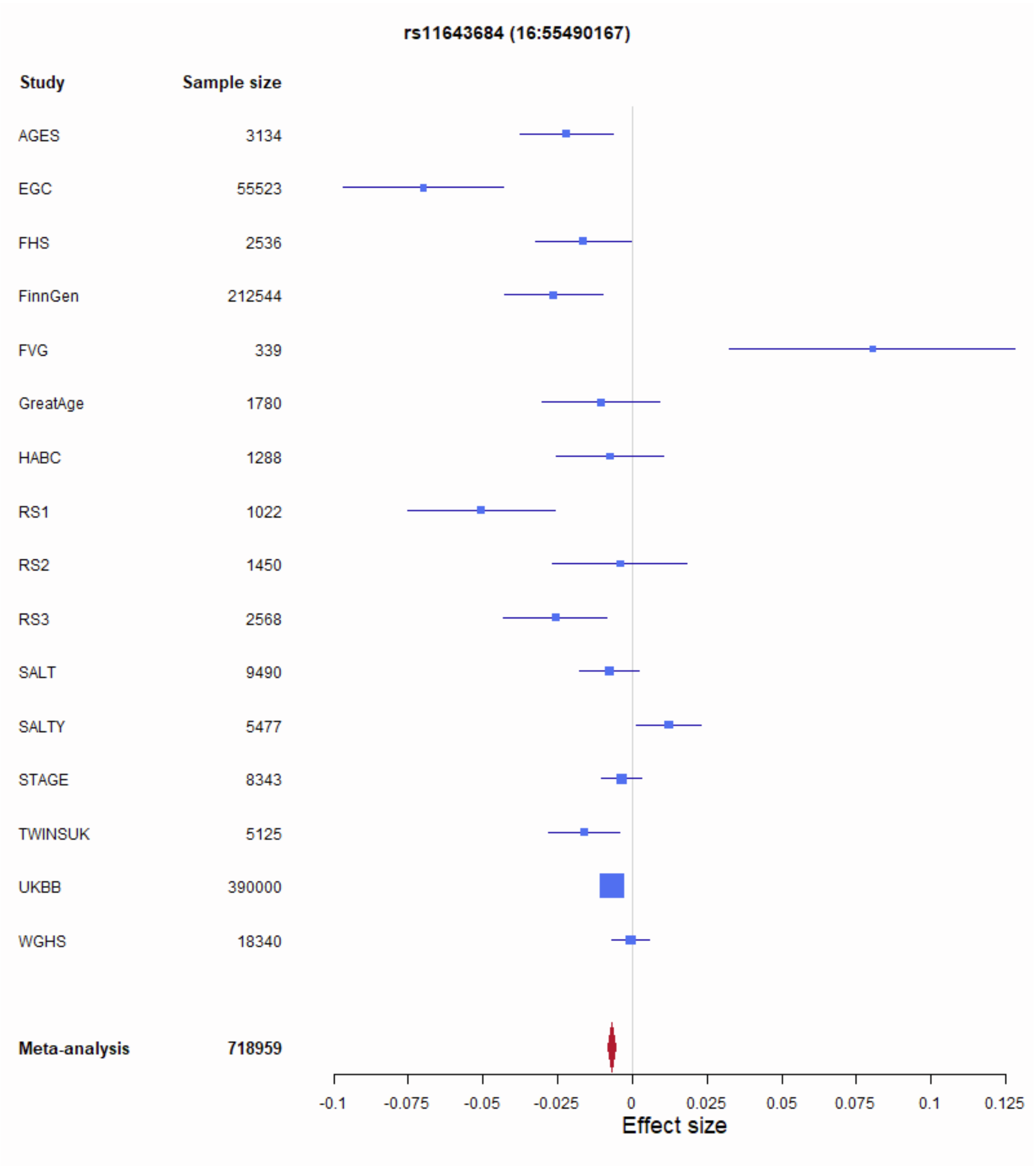

Locus annotation: IRX6--[x]-MMP2

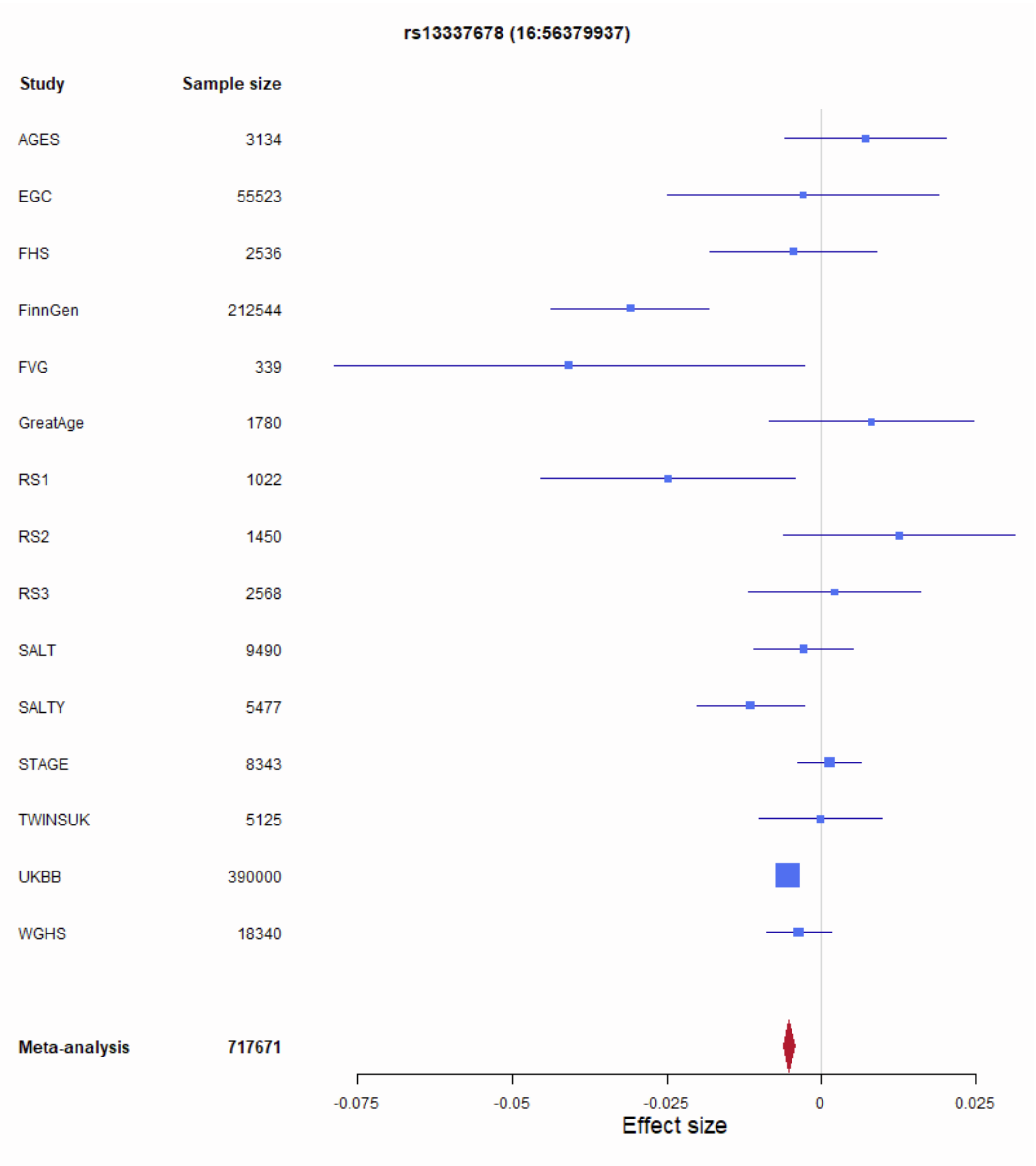

Locus annotation: [GNAO1] 3' UTR

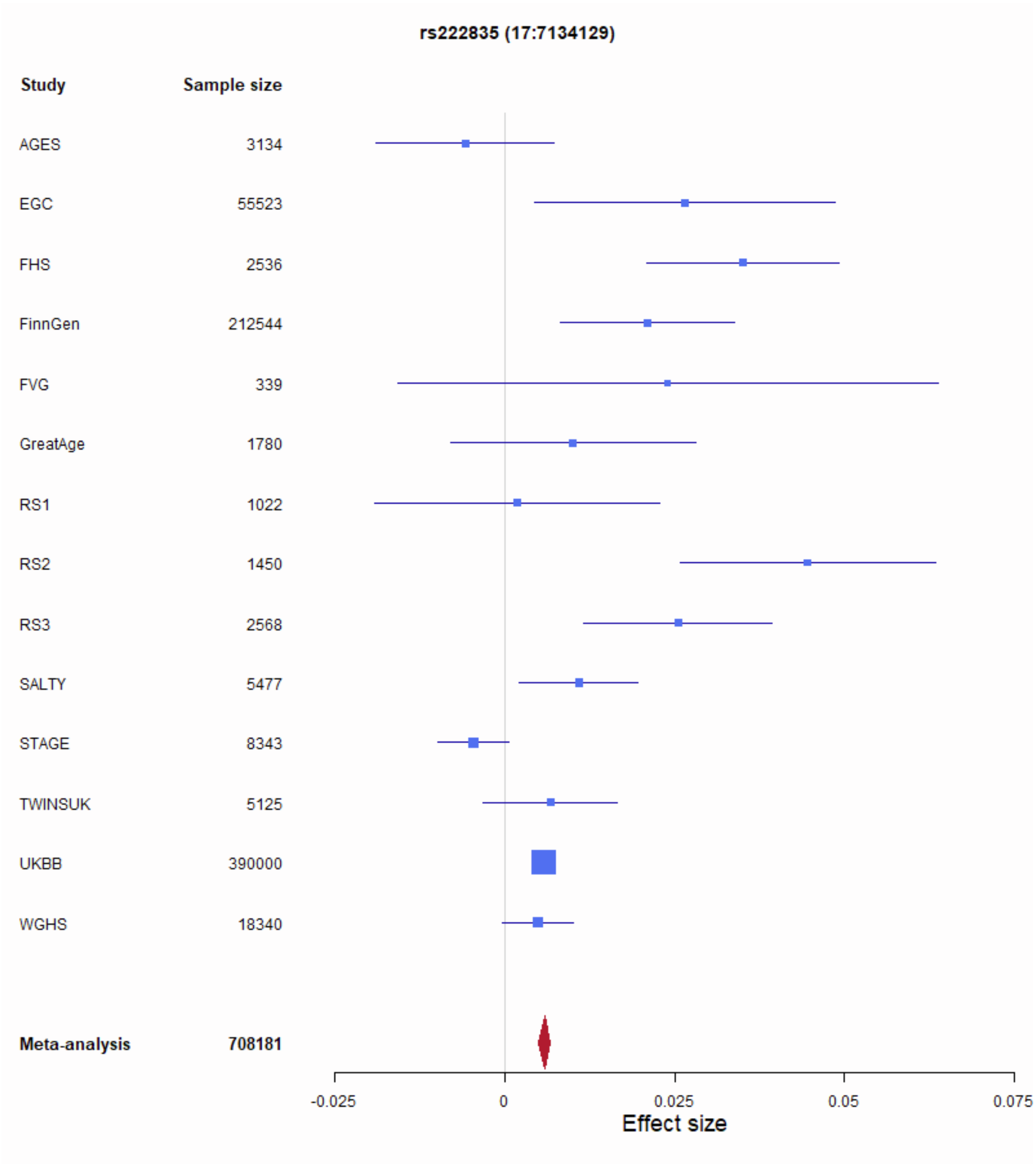

Locus annotation: [DVL2] intronic

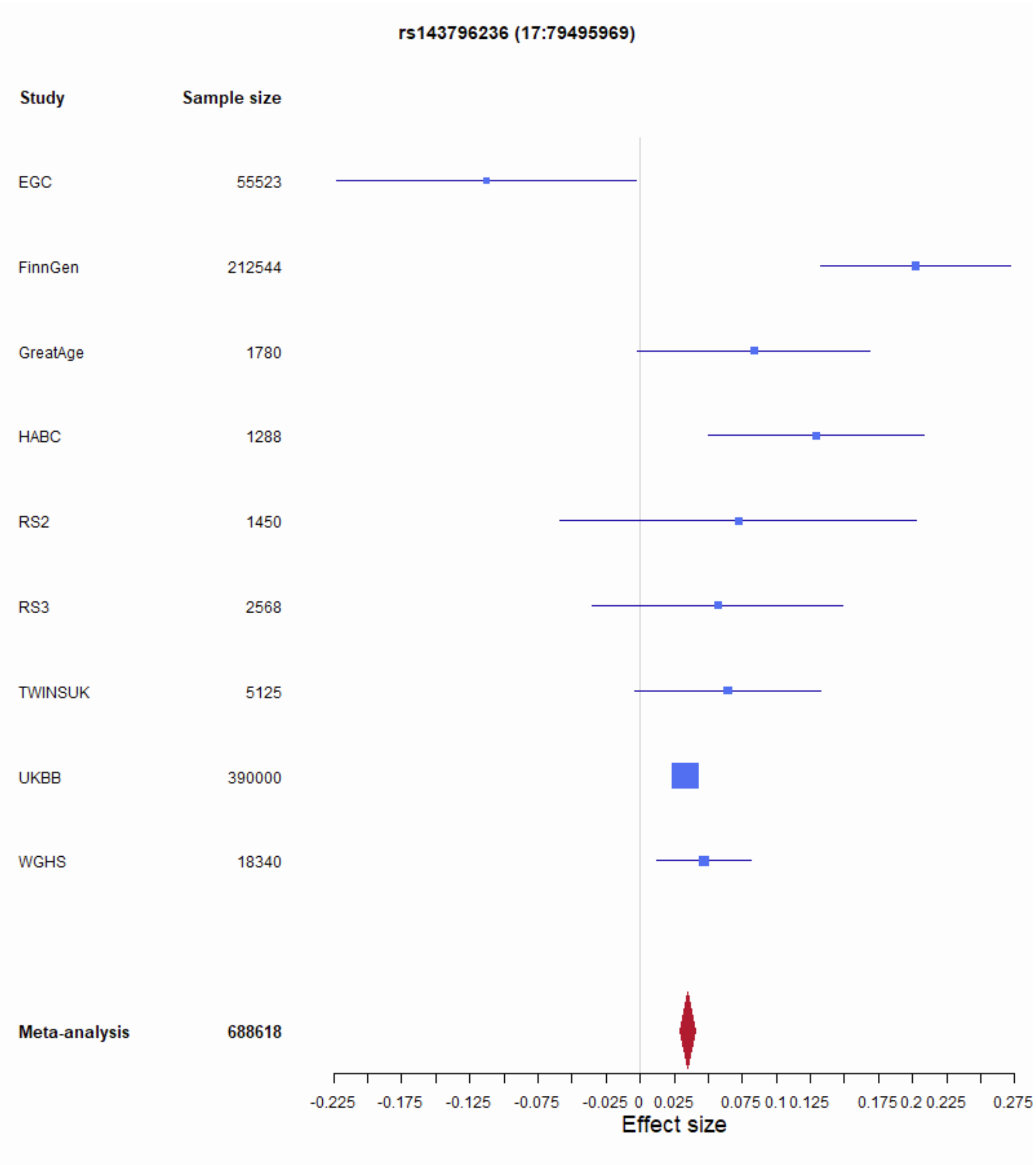

Locus annotation: [FSCN2] H>Y

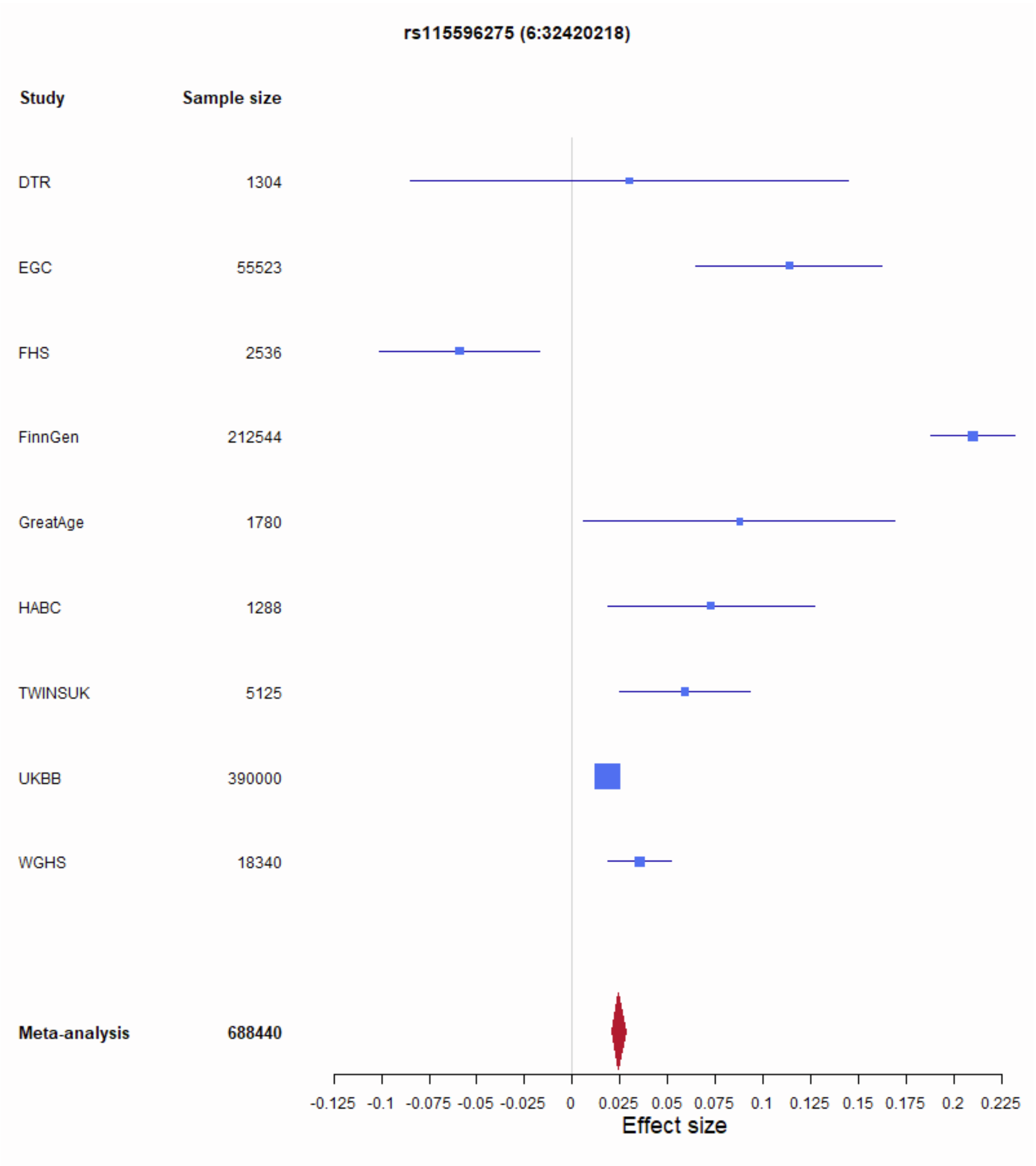

Locus annotation: [CCDC68] 5' UTR

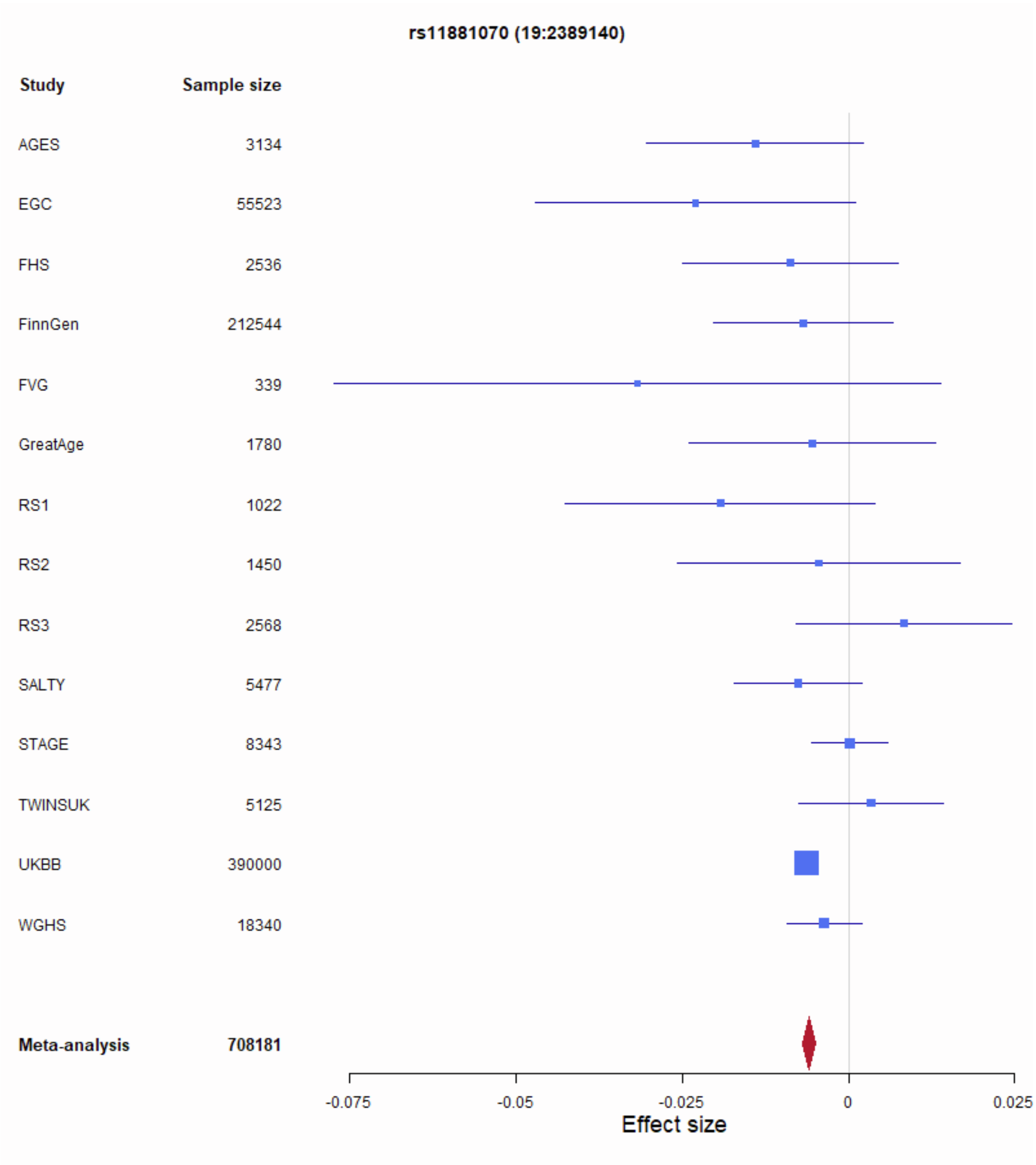

Locus annotation: SPPL2B-[x]-TMRPS9

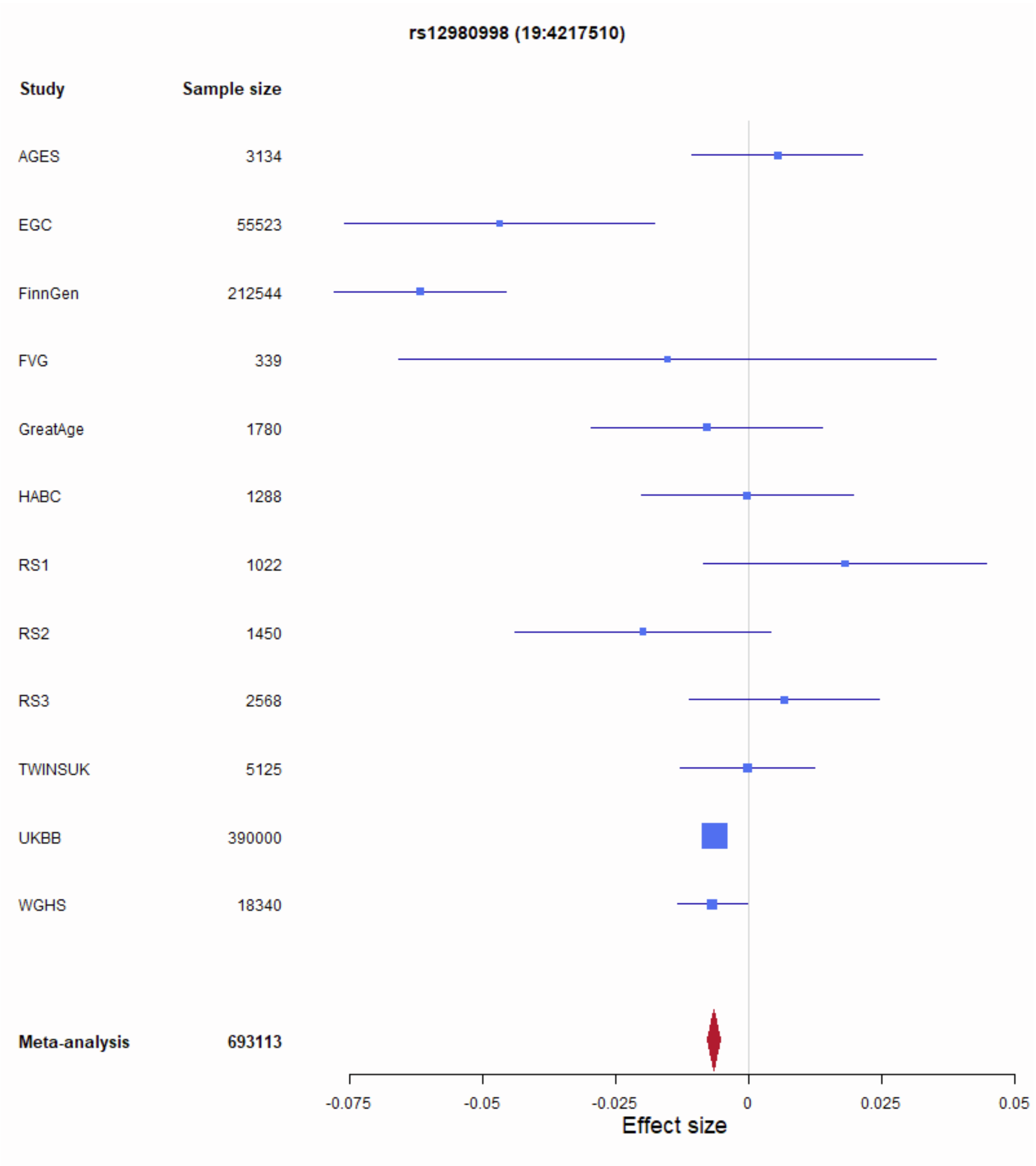

Locus annotation: [ANKRD24] T>S

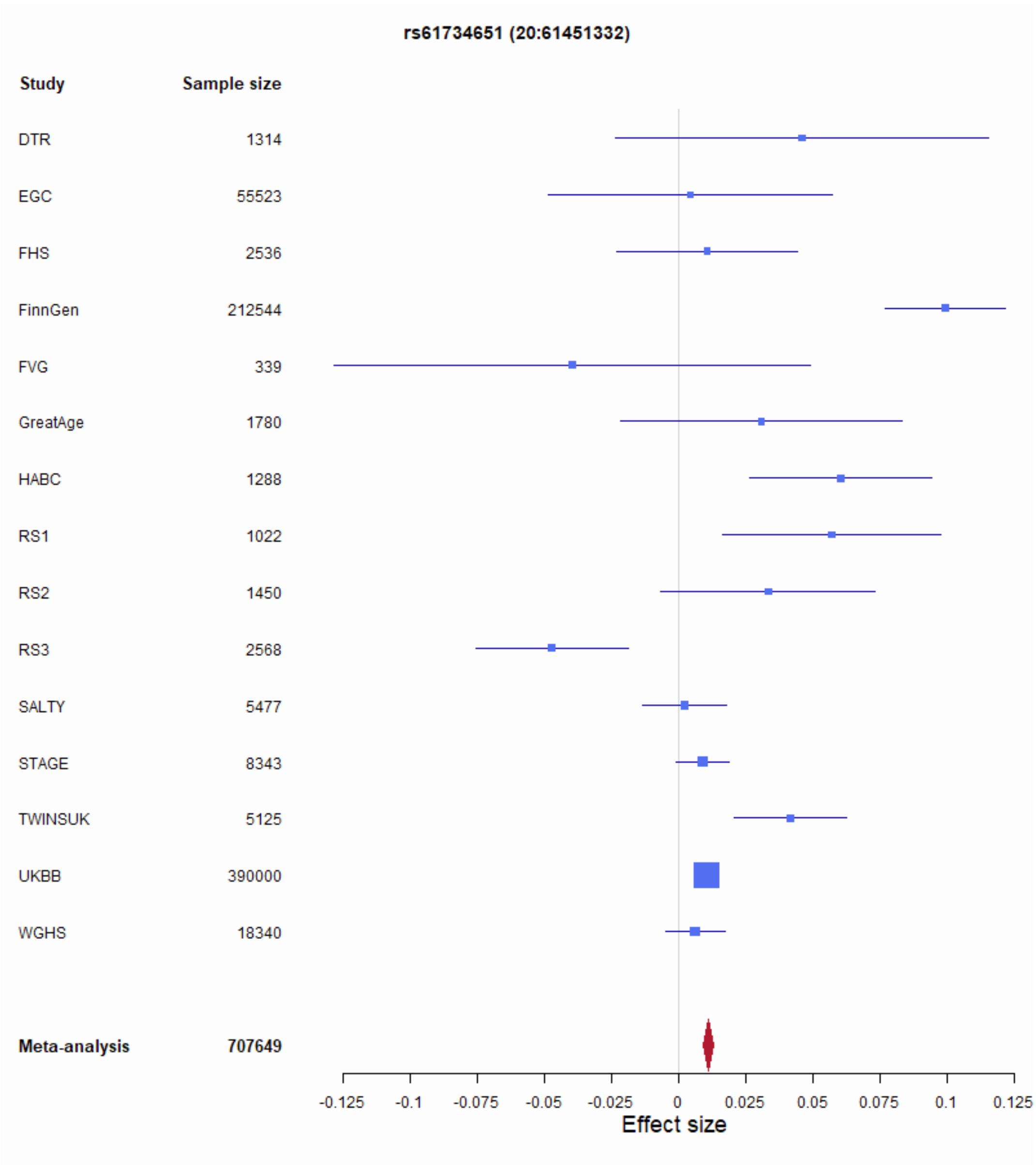

Locus annotation: [COL9A3] R>W

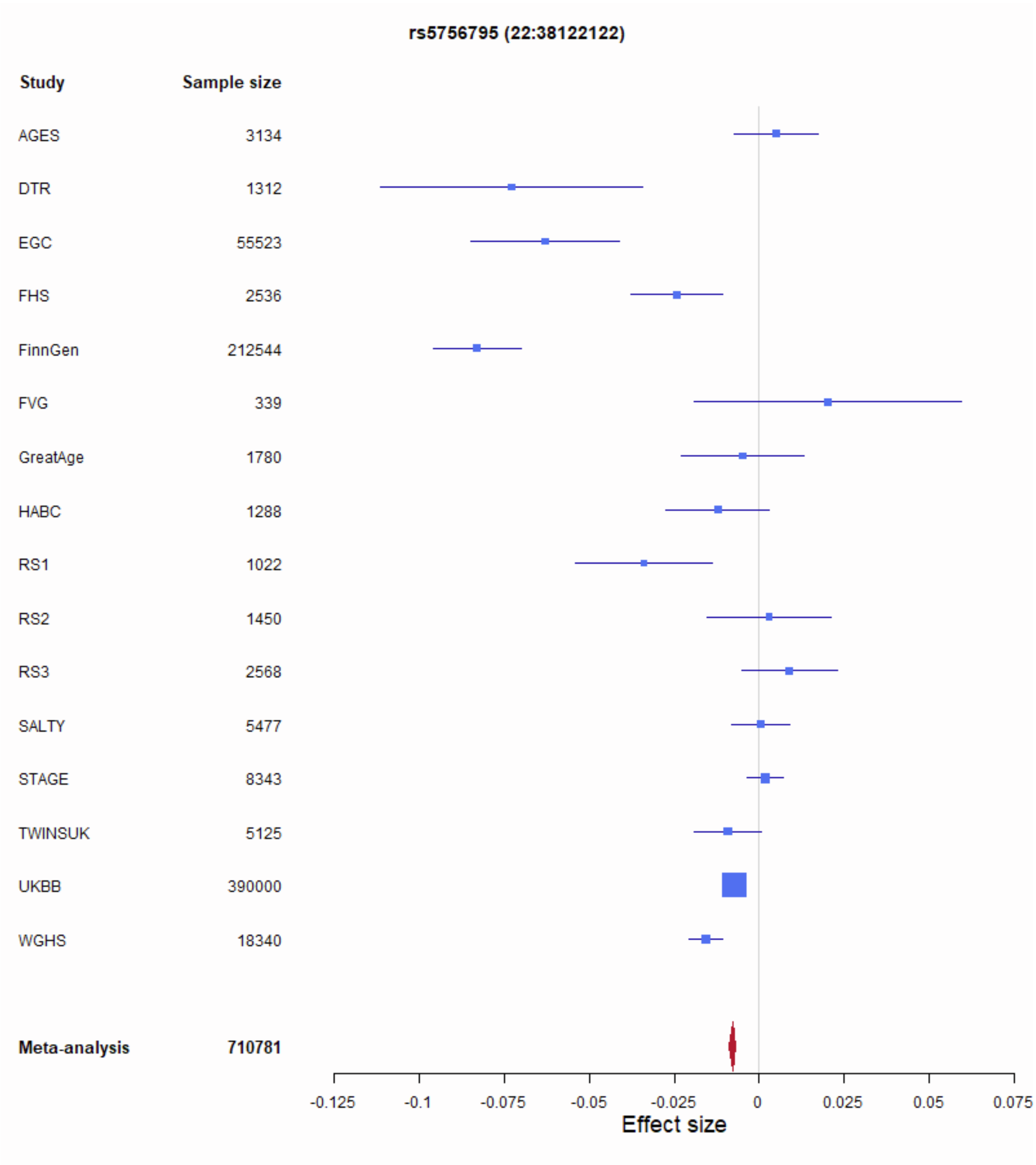

Locus annotation: [TRIOBP] F>I

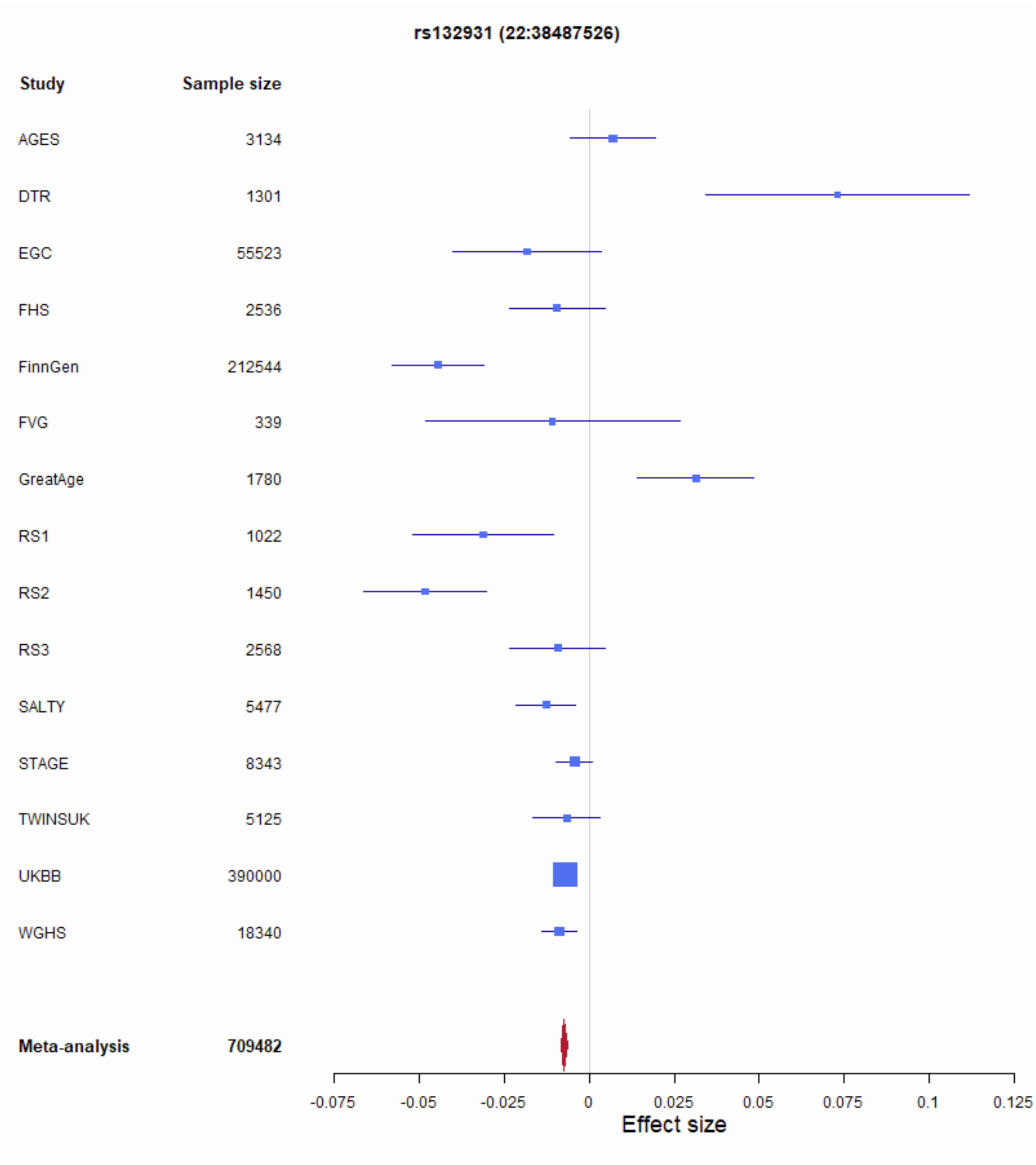

Locus annotation: [BAIAP2L2] intronic

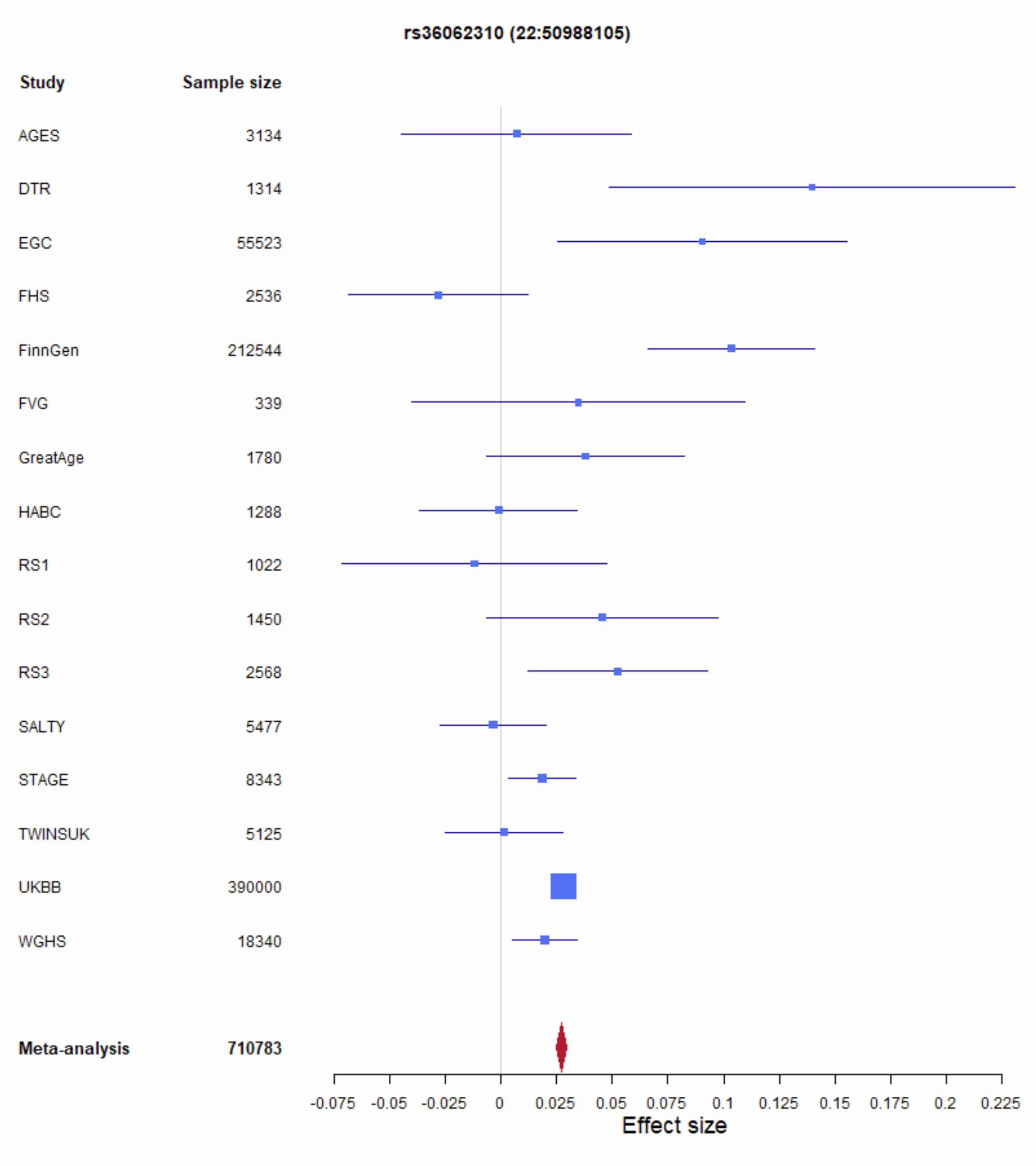

Locus annotation: [KLHDC7B] V>M

Figure S4. Manhattan plot for gene-based analysis. All significant genes (p<2.66e-6) identified by MAGMA (red) and genes identified by MAGMA and VEGAS simultaneously (blue).

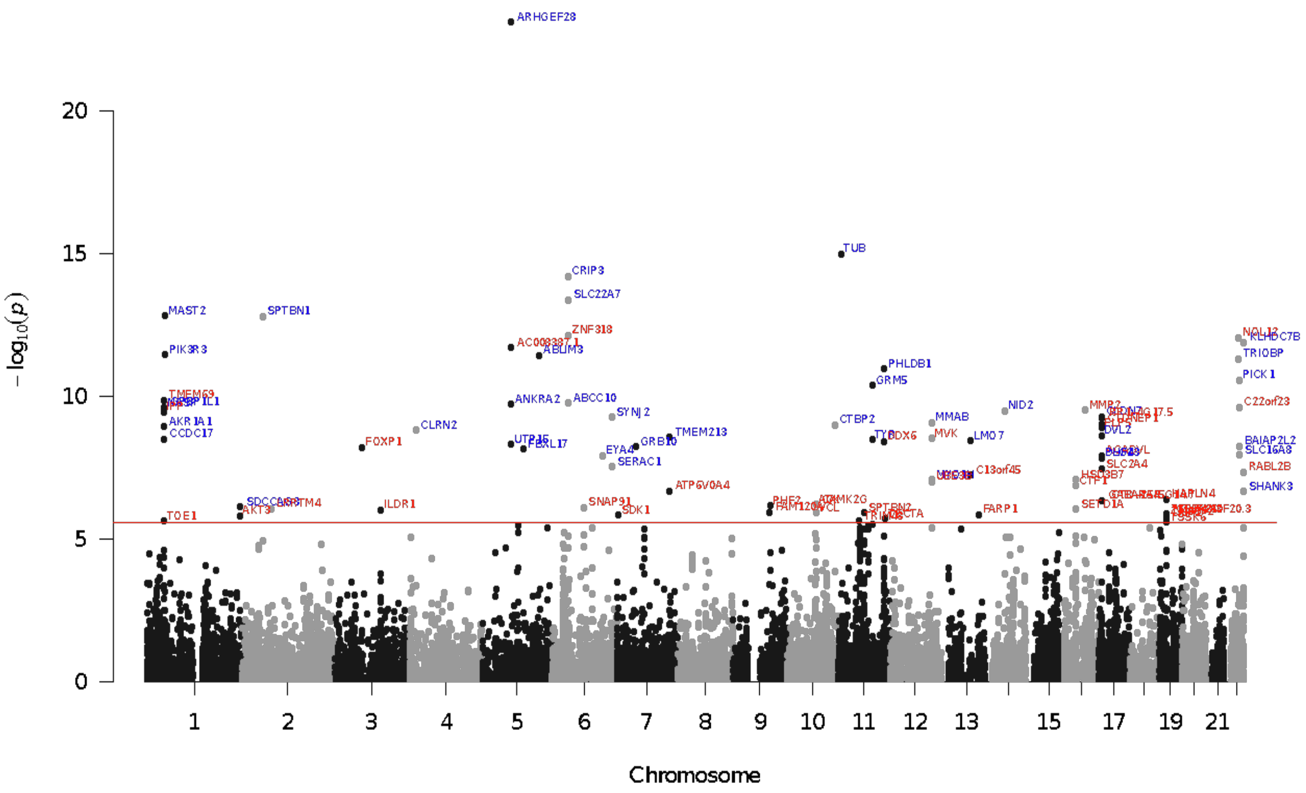

Figure S5. a) Protein structure and modeling of the missense variant effect on GJB2 transporter b) Hydrophilicity/hydrophobicity map

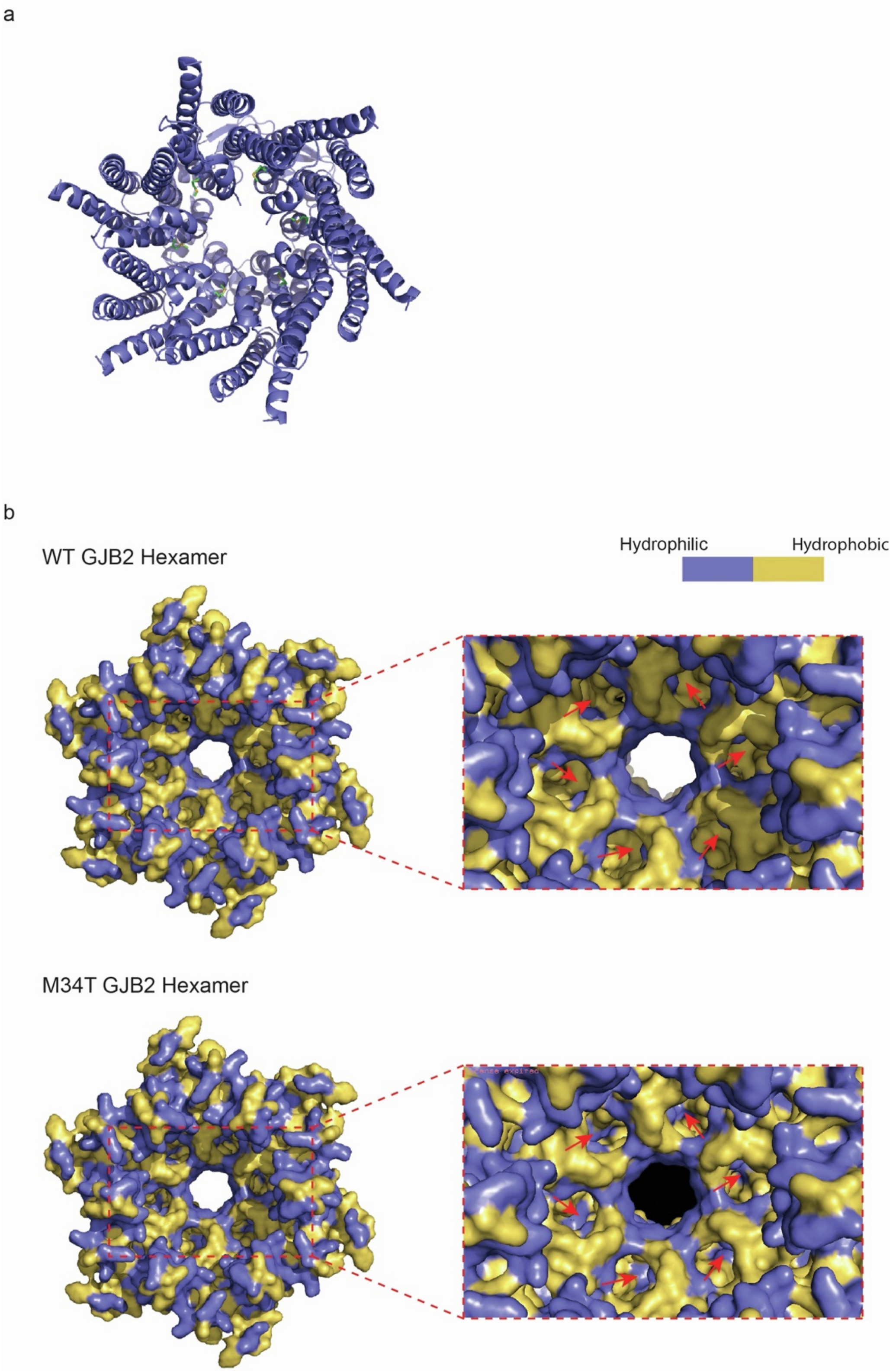

Figure S6. Genetic correlation between age-related hearing impairment and selected number of traits and diseases based on relevance in LDHub. Black filled circles pass the Bonferroni corrected significance threshold (p < 2e-04); bars represent standard error.

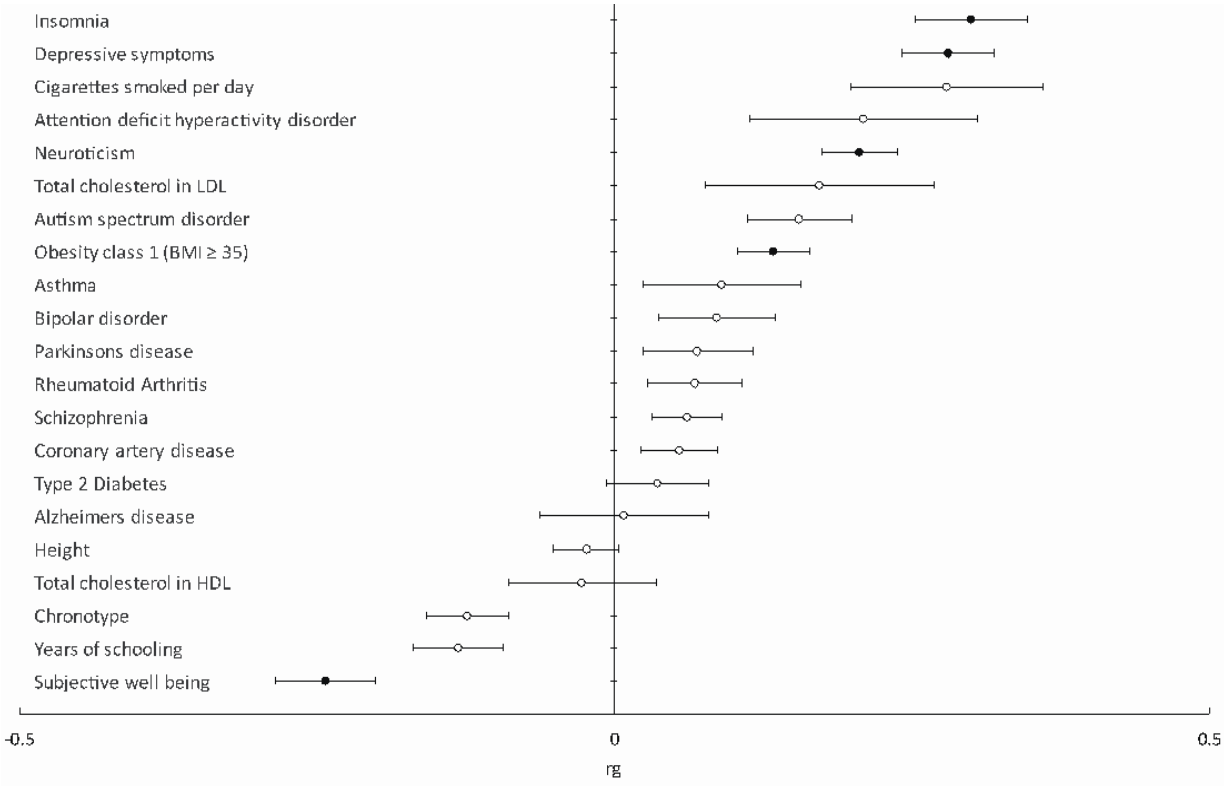

Figure S7. Genetic correlation between age-related hearing impairment and selected GWAS traits based on clinical relevance, collected by the Psychiatric Genomics Consortium. Black filled circles pass the Bonferroni corrected significance threshold (p < 0.001); bars represent standard error.

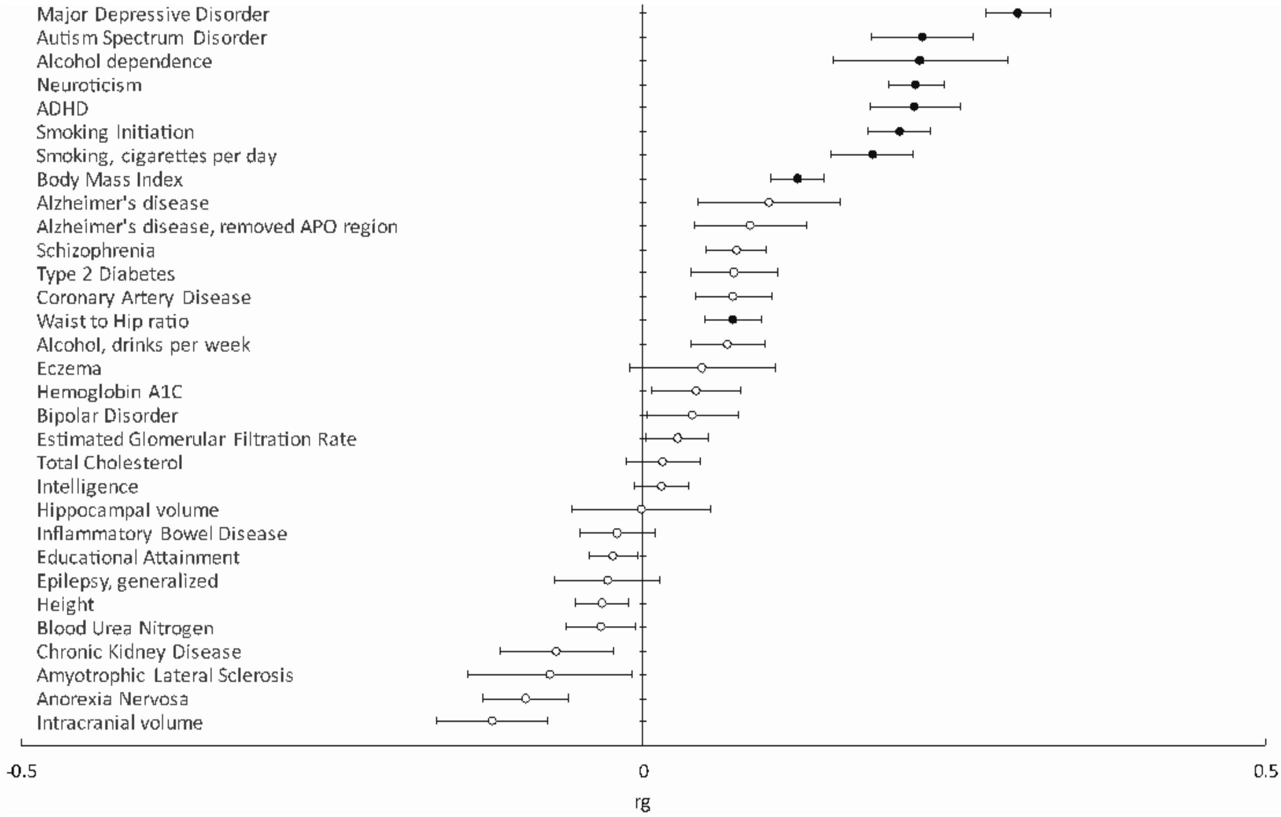

Figure S8. Tissue-set enrichment analysis of GTEx V8 tissues using MAGMA and partitioned LDSC. Bonferroni corrected significance threshold of -log10P-mean > 2.86. Bonferroni corrected significance threshold for MAGMA and LDSC P < 0.001

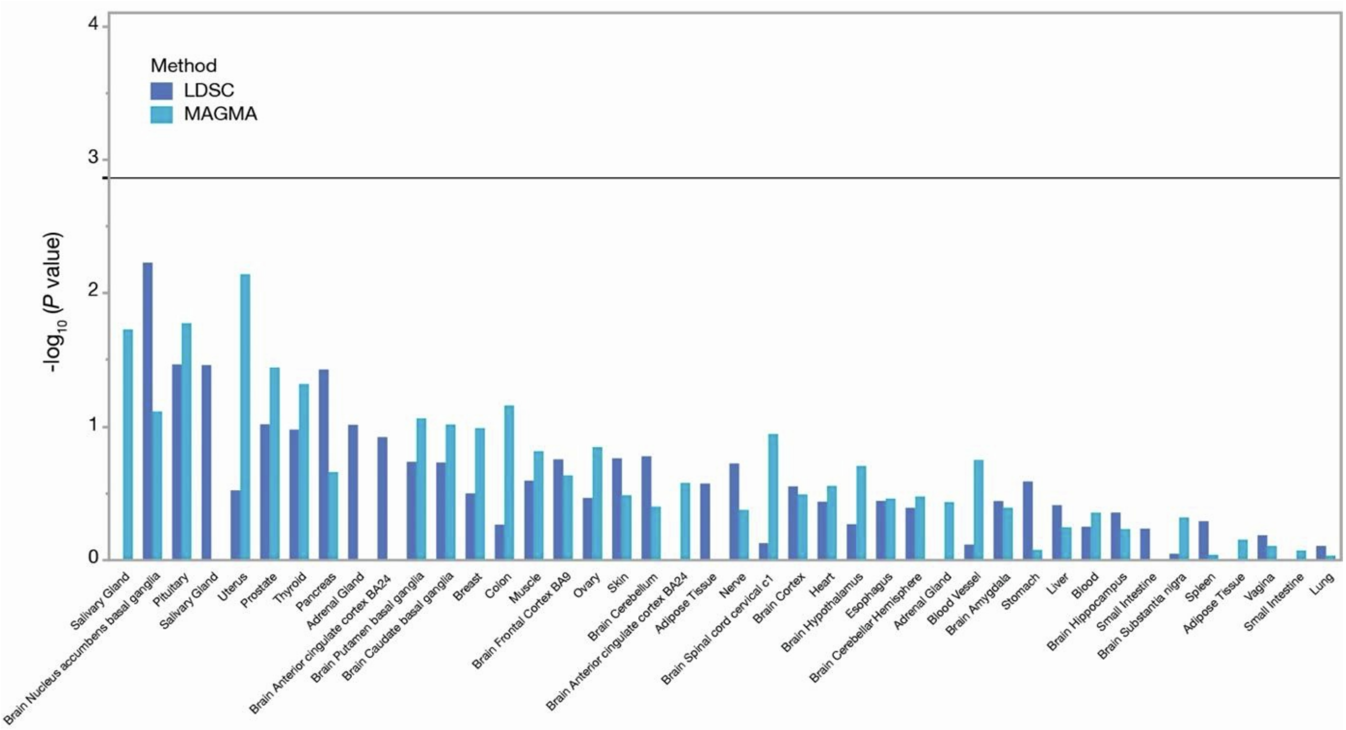

Figure S9. Density plot and b) bar plot demonstrating the proportion of missense variants in GWAS studies with more than 10 significant loci obtained from GWAS catalog. Fisher exact test p-values = 0.005

a)

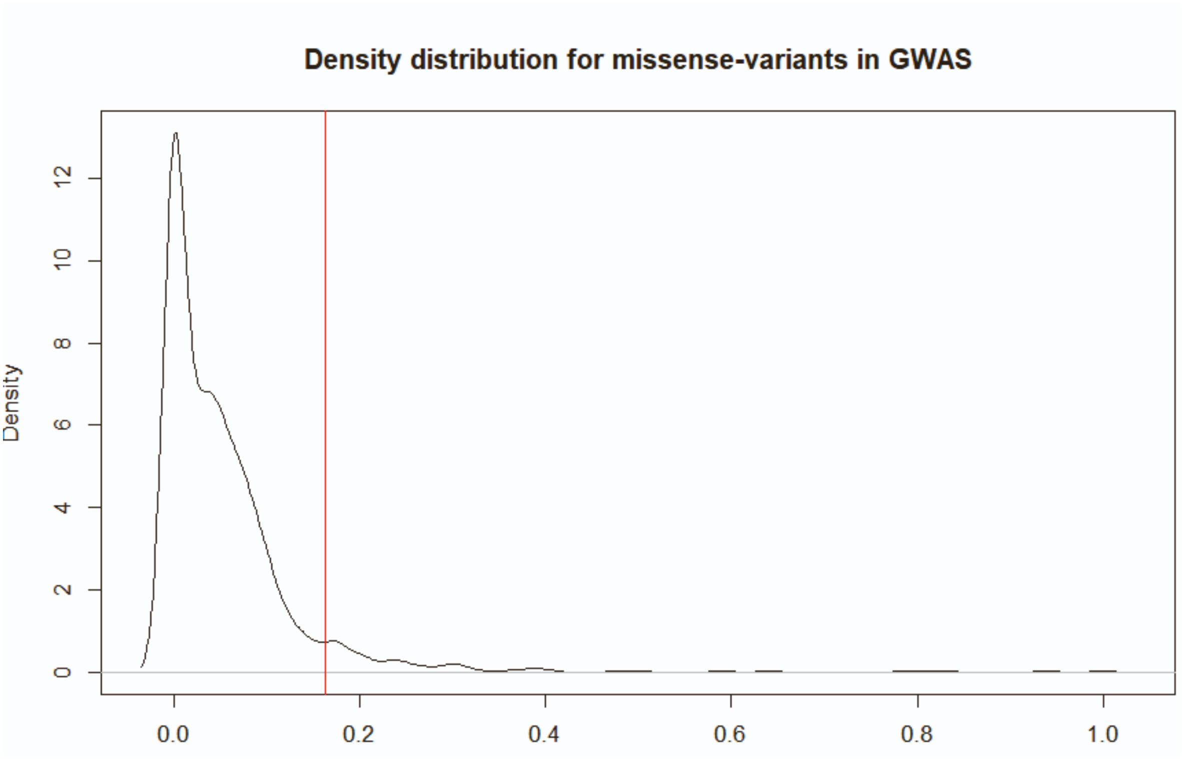

b)

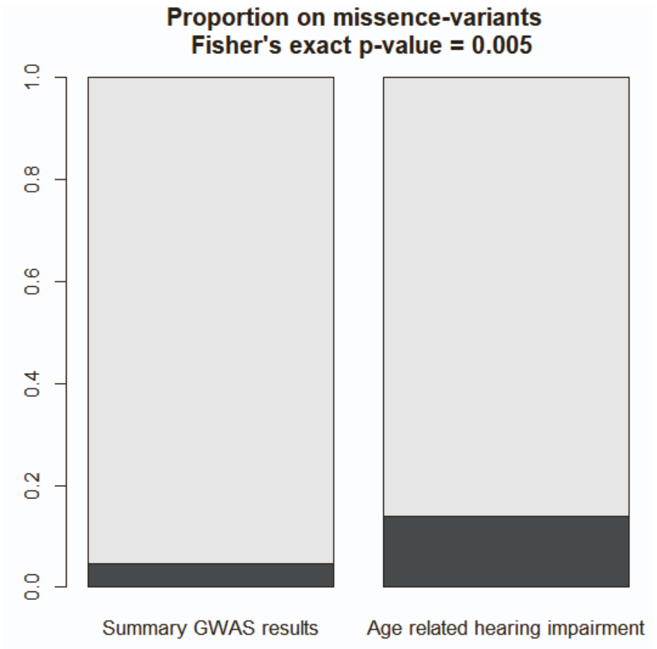

Supplementary Methods

Study Descriptions

Age, Gene/Environment Susceptibility Reykjavik Study: AGES-Reykjavik Study

The Reykjavik Study cohort originally comprised a random sample of 30,795 men and women born in 1907-1935 and living in Reykjavik in 1967[17]. A total of 19,381 people attended, resulting in 71% recruitment rate. The study sample was divided into six groups by birth year and birth date within month. One group was designated for longitudinal follow up and was examined in all stages. One group was designated a control group and was not included in examinations until 1991. Other groups were invited to participate in specific stages of the study. Between 2002 and 2006, the AGES-Reykjavik study re-examined 5764 survivors of the original cohort who had participated before in the Reykjavik Study.

The Danish Twin Registry

The Danish Twin Registry (DTR) sample included 1,314 individuals collected as part of the study of Middle Age Danish Twins (MADT, N=1,055) and the Longitudinal Study of Aging Danish Twins (LSADT, N=259)[18]. MADT was initiated in 1998 and includes 4,314 twins randomly chosen from the birth years 1931-1952. Surviving participants were revisited from 2008 to 2011, where the blood samples used in the present study were collected. The survey data used in the present study was obtained from the Omnibus 2 survey undertaken in 2002[18]. LSADT was initiated in 1995 and includes twins aged 70 years and older. Follow-up assessments were conducted every second year through 2005. The individuals included here all participated in the 1997 assessment, where blood samples were collected from same sex twin pairs, and in the 2001 assessment where the survey data used in the present study was collected[18].

Written informed consents were obtained from all participants. Collection and use of biological material, and survey and registry information were approved by the Regional Scientific Ethical Committees for Southern Denmark (MADT: S-VF-19980072, LSADT: S-VF-20040241). The study *is registered in SDU's internal list (notification no. 10.903) and complies with the rules in the General Data Protection Regulation.*

EGCUT

EstBB is a population-based cohort of 200,000 participants with a rich variety of phenotypic and health-related information collected for each individual[19]. At recruitment, participants have signed a consent to allow follow-up linkage of their electronic health records (EHR), thereby providing a longitudinal collection of phenotypic information. EstBB allows access to the records of the national Health Insurance Fund Treatment Bills (from 2004), Tartu University Hospital (from 2008) and North Estonia Medical Center (from 2005). For every participant there is information on diagnoses in ICD-10 coding and drug dispensing data, including drug ATC codes, prescription status and purchase date (if available).

At the time of this study 155,772 genotyped samples from the Estonian Biobank were available and genotyping was done at the Genotyping Core Facility of the Institute of Genomics, University of Tartu, using the Global Screening Array (GSAv1.0, GSAv2.0, and GSAv2.0_EST) from Illumina. Altogether 155,772 samples were genotyped and PLINK format files were exported using GenomeStudio v2.0.4. During the quality control all individuals with call-rate < 95% or mismatching sex that was defined based on the heterozygosity of X chromosome and sex in the phenotype data, were excluded from the analysis. Variants were filtered by call-rate < 95% and HWE p-value < 1e-4 (autosomal variants only). Variant positions were updated to Genome Reference Consortium Human Build 37 and all variants were changed to be from TOP strand using reference information

provided by Dr. Will Rayner from the University of Oxford (https://www.well.ox.ac.uk/~wrayner/strand/). Before imputation variants with MAF<1% and Indels were removed. Prephasing was done using the Eagle v2.3 software[20] (number of conditioning haplotypes Eagle2 uses when phasing each sample was set to: --Kpbwt=20000) and imputation was carried out using Beagle v.28Sep18.793[21,22] with an effective population size ne=20,000. As a reference, Estonian population specific imputation reference of 2,297 WGS samples was used[23]. For the current study, we determined 5,717 cases of hearing loss based on the participants EHRs as individuals with the ICD10 codes H90.3 or H91.1 and included only subjects of 45 years of age and above. We excluded among cases individuals with additional records of any of the following ICD10 diagnosis: H80, H81.0, H83, H90.4, H91.0, H91.2, H91.3, H93.0, H93.3, H94*, H95. Controls (n=49,806) were individuals 45 years of age and above and defined as undiagnosed participants. Individuals with records of H65, H66, H67, H68, H69, H70, H71, H72, H73, H74,H75,H80, H81.0, H83, H90, H91, H92, H93, H95, Q16, Z45.3,Z46.1,Z96.2,Z97.4 were removed among controls. We conducted the GWASes using the SAIGE software[24] adjusting for the first ten principal components of the genotype matrix, as well as for age and sex.

FinnGen

The FinnGen research project (www.finngen.fi) was launched in 2017 with an aim to improve human health through genetic research. The project combines genome information with digital health care data from national registries: the genotype data are linked to national hospital discharge, death, cancer, and medication reimbursement registries using the national personal identification numbers. The FinnGen study will combine approximately 200,000 existing samples from Finnish biobanks with approximately 300,000 samples from ongoing collections. Once final, the data resource will cover roughly 10% of the Finnish population. The present study comprised data of 212,544 Finnish adults (15,952 cases; 196,592 controls) from FinnGen Preparatory Phase Data Freeze 5.

Patients and control subjects in FinnGen provided informed consent for biobank research, based on the Finnish Biobank Act. Alternatively, separate research cohorts, collected prior the Finnish Biobank Act came into effect (in September 2013) and start of FinnGen (August 2017), were collected based on study-specific consents and later transferred to the Finnish biobanks after approval by Fimea, the National Supervisory Authority for Welfare and Health. Recruitment protocols followed the biobank protocols approved by Fimea. The Coordinating Ethics Committee of the Hospital District of Helsinki and Uusimaa (HUS) approved the FinnGen study protocol Nr HUS/990/2017.

The FinnGen study is approved by Finnish Institute for Health and Welfare (THL), approval number THL/2031/6.02.00/2017, amendments THL/1101/5.05.00/2017, THL/341/6.02.00/2018, THL/2222/6.02.00/2018, THL/283/6.02.00/2019, THL/1721/5.05.00/2019, Digital and population data service agency VRK43431/2017-3, VRK/6909/2018-3, VRK/4415/2019-3 the Social Insurance Institution (KELA) KELA 58/522/2017, KELA 131/522/2018, KELA 70/522/2019, KELA 98/522/2019, and Statistics Finland TK-53-1041-17.

The Biobank Access Decisions for FinnGen samples and data utilized in FinnGen Data Freeze 5 include: THL Biobank BB2017_55, BB2017_111, BB2018_19, BB_2018_34, BB_2018_67, BB2018_71, BB2019_7, BB2019_8, BB2019_26, Finnish Red Cross Blood Service Biobank 7.12.2017, Helsinki Biobank HUS/359/2017, Auria Biobank AB17-5154, Biobank Borealis of Northern Finland_2017_1013, Biobank of Eastern Finland 1186/2018, Finnish Clinical Biobank Tampere MH0004, Central Finland Biobank 1-2017, and Terveystalo Biobank STB 2018001.

Framingham Heart Study

The Framingham Heart Study is a prospective longitudinal investigation of the development of atherosclerosis and its clinical sequelae. Study participants were recruited at three time periods. The study was initiated in 1948-50 with the recruitment of 5209 individuals ages 28-62 (including some spouse pairs, parent-offspring pairs and siblings) for the purpose of investigating the multiple factors involved in the development of cardiovascular disease[25]. This group, known as the Original Cohort, has been examined every two years with a total of thirty-two examinations to date. In 1971-1975, offspring of the Original Cohort and the offspring spouses were recruited to examine among other goals the familial components of cardiovascular disease and its risk factors[26]. In 2002-2005, the third generation (children of the Offspring and grandchildren of the Original Cohort) was recruited[27]. The Offspring Cohort totalled 5124 and the Third Generation totalled 4095 at recruitment and have been examined every 4 to 8 years. The Offspring Cohort now has 9 examinations completed and the Third Generation has 2 examinations completed.
Between 1973 and 1975, hearing examinations were conducted on 2293 members of the original cohort, and between 1995 and 1999, identical examinations were conducted on 2262 members of the offspring cohort. Standard pure-tone audiograms were obtained on all participants using environments and meeting American National Standards Institute standards.

G-EAR

Within the International consortium called G-EAR, we used, in this study, individuals coming from two isolated cohorts: **Friuli Venezia Giulia (FVG)** Genetic Park and the **Salus in Apulia Study** (formerly known as Great Age Study).
The FVG cohort is a collection of samples coming from six small villages (Clauzetto, Erto, Illegio, Resia, San Martino del Carso and Sauris) located in north-eastern Italy, in the Friuli Venezia Giulia region[28]. The FVG Genetic Park is part of the INGI project, a collaboration between research institutions in Italy aimed at reconstructing the molecular bases of complex traits by investigating genetically isolated Italian populations. Studies were conducted referring to a common operational protocol. In each population, genotype samples were collected, alongside a detailed anamnesis, more than 120 biochemical parameters and 400 phenotypes, including anthropometric measures, lifestyle habits, diseases and pure-tone audiometry[29].
The "Salus in Apulia Study" is an ongoing population-based prospective cohort comprising 2,472 individuals aged >= 65 years and residents in Castellana Grotte, a town located near Bari, Puglia, in the Southeast of Italy. It focused on the sequence of lifestyle including diet, frailty, and other age-related impairments and age-related disease outcomes. In detail, Salus is a public health initiative funded by the Italian Ministry of Health and Apulia Regional Government and carried on at IRCCS "S. De Bellis" that combines data from two previous populations: the baseline data (MICOL3, M3) were recorded from 2003 to 2005 and the follow-up data from 2013 to 2015 (GreatAGE Study - MICOL4, M4). The GreatAGE-M4 study has been described elsewhere[30]. The invitation included also subjects of the MICOL studies that were in the respective age range above 64 years. In the GreatAge-M4 examination, in addition to the assessment of clinical and lifestyle aspects, sensory-related outcomes have been also evaluated together with neuropsychological features and genetic components. The study adhered to the "Standards for Reporting Diagnostic Accuracy Studies" (STARD) guidelines (http://www.stard-statement.org/), the "Strengthening the Reporting of Observational Studies in Epidemiology" (STROBE) guidelines (https://www.strobe-statement.org/).

Health, Aging, and Body Composition (HABC) Study

The HABC Study is a NIA-sponsored cohort study of the factors that contribute to incident disability and the decline in function of healthier older persons, with a particular emphasis on changes in body composition in old age. Between March 1997 and July 1998, 3075 70-79 year-old community-dwelling adults (41% African-American) were recruited to participate in the Health ABC Study.

Medicare beneficiary listings were used to recruit in metropolitan areas surrounding Pittsburgh, Pennsylvania, and Memphis, Tennessee. Eligibility criteria included having no difficulty walking one-quarter of a mile, climbing 10 steps, or performing activities of daily living (transferring, bathing, dressing, and eating); no history of active treatment for cancer in the prior 3 years; and no plans to move from the area within 3 years.

The Rotterdam Study

The Rotterdam study is a prospective, population-based cohort study among inhabitants of Ommoord, a district of Rotterdam, The Netherlands[31]. As of 2008, 14,926 subjects aged 45 years or over comprise the cohort. Since 2016, it is being expanded by persons aged 40 years and over. The Rotterdam study targets cardiovascular, endocrine, hepatic, neurological, ophthalmic, psychiatric, dermatological, otolaryngological, locomotor, and respiratory diseases. The participants were all examined in some detail at baseline. They were interviewed at home (2 h) and then had an extensive set of examinations (a total of 5 h) in a specially built research facility in the center of the district. Written informed consent was obtained from all participants and the Medical Ethics Committee of the Erasmus Medical Center, Rotterdam, approved the study.

The Swedish Twin Registry

The Swedish Twin Registry (STR) sample included in this study was recruited among Swedish born twins participating in two main national ascertainments; 1) the Screening Across the Lifespan Twin Study (SALT), which was a telephone interview study conducted in 1998-2002, covering twins born before 1959 and 2) Study of Twin Adults: Genes and Environment (STAGE), an online questionnaire study conducted 2005-2006, covering twins born 1959-1985[32]. Phenotypic information about hearing was collected though self-reports in these studies. Additional phenotypic information was available from a paper questionnaire (called SALTY) administered during 2009 to SALT participants born 1944-1985. DNA was collected from blood or saliva, and extracted by standard procedures. All genotyping was performed by SNP&SEQ genotyping facility in Uppsala, in three waves using three different Illumina chip arrays, OmniExpress for the blood DNA, Psychchip and Global screening array for the saliva DNA samples. Written informed consents were obtained from all participants.

TwinsUK

TwinsUK is the only adult twin registry in the UK, comprising of over 12,000 healthy twin volunteers aged 16-98[33]. Collection of data and biologic materials commenced in 1992 and is ongoing. Twins have completed detailed health and lifestyle questionnaires, and attended clinical evaluations. The pure tone audiometry data was collected on a subset of the cohort (N=1242) between April 2010 and November 2012. Participants were recruited with an aim to study aging in females. An air-conduction pure-tone audiogram was conducted by trained personnel using a Madsen XETA audiometer including TDH39 headphones. All research was conducted according to the ethical standards as defined by the Helsinki declaration. Ethical approval for this study was obtained from the National Research Ethics service London-Westminster (REC reference number: 07/H0802/84). Written informed consent was obtained from all participants prior to study conduction. Participants were excluded from analysis based on missing data, male, age <45. There were 819 female participants aged >45 remaining for analysis.

Women's Genome Health Study (WGHS)

WGHS is a prospective cohort of female North American health care professionals representing participants in the Women's Health Study (WHS) trial who provided a blood sample at baseline and consent for blood-based analyses[34]. Participants in the WHS were 45 years or older at enrollment and free of cardiovascular disease, cancer or other major chronic illness. The current data are derived from 23,294 WGHS participants for whom whole genome genotype information was available at the time of analysis and for whom self-reported European ancestry could be confirmed

by multidimensional scaling analysis of 1,443 ancestry informative markers in PLINK v. 1.06. At baseline, BP and lifestyle habits related to smoking, consumption of alcohol, and physical activity as well as other general clinical information were ascertained by a self-reported questionnaire, an approach which has been validated in the WGHS demographic, namely female health care professionals.

Information on hearing loss in the WHS (the WGHS parent cohort) was collected by self-report on the fourth observational questionnaire in 2008. Participants were asked: “As you age, do you have more trouble hearing in a crowded room?” with choices of “yes” or “no” as a response, encoded as 1 or 0, respectively.

Additional acknowledgements

The **AGES-Reykjavik** study has been funded by NIH contract N01-AG012100, the NIA Intramural Research Program, an Intramural Research Program Award (ZIAEY000401) from the National Eye Institute, an award from the National Institute on Deafness and Other Communication Disorders (NIDCD) Division of Scientific Programs (IAA Y2-DC_1004-02), Hjartavernd (the Icelandic Heart Association), and the Althingi (the Icelandic Parliament). The study is approved by the Icelandic National Bioethics Committee, VSN: 00-063. The researchers are indebted to the participants for their willingness to participate in the study.

DTR is supported by grants from The National Program for Research Infrastructure 2007 from the Danish Agency for Science, Technology and Innovation (09-063256) and the US National Institutes of Health (P01 AG08761). Genotyping was supported by NIH R01 AG037985 (Pedersen).

EstBB thanks all participants and staff of the Estonian biobank for their contribution to this research and the analytical work of EstBB was carried out in part in the High Performance Computing Center of the University of Tartu. We acknowledge the work of the Estonian Biobank Research Team: Andres Metspalu, Mari Nelis, Reedik Mägi and Tõnu Esko. The work by KK and LM was supported by the European Union through the European Regional Development Fund (Project No. 2014-2020.4.01.15-0012).

The **FinnGen** project is funded by two grants from Business Finland (HUS 4685/31/2016 and UH 4386/31/2016) and the following industry partners: AbbVie Inc., AstraZeneca UK Ltd, Biogen MA Inc., Bristol Myers Squibb (and Celgene Corporation & Celgene International II Sàrl), Genentech Inc., Merck Sharp & Dohme Corp, Pfizer Inc., GlaxoSmithKline Intellectual Property Development Ltd., Sanofi US Services Inc., Maze Therapeutics Inc., Janssen Biotech Inc, Novartis AG, and Boehringer Ingelheim. Following biobanks are acknowledged for delivering biobank samples to FinnGen: Auria Biobank (www.auria.fi/biopankki), THL Biobank (www.thl.fi/biobank), Helsinki Biobank (www.helsinginbiopankki.fi), Biobank Borealis of Northern Finland (https://www.ppshp.fi/Tutkimus-ja-opetus/Biopankki/Pages/Biobank-Borealis-briefly-in-English.aspx), Finnish Clinical Biobank Tampere (www.tays.fi/en-US/Research_and_development/Finnish_Clinical_Biobank_Tampere), Biobank of Eastern Finland (www.ita-suomenbiopankki.fi/en), Central Finland Biobank (www.ksshp.fi/fi-FI/Potilaalle/Biopankki), Finnish Red Cross Blood Service Biobank (www.veripalvelu.fi/verenluovutus/biopankkitoiminta) and Terveystalo Biobank (www.terveystalo.com/fi/Yritystietoa/Terveystalo-Biopankki/Biopankki/). All Finnish Biobanks are members of BBMRI.fi infrastructure (www.bbmri.fi). Finnish Biobank Cooperative -FINBB (https://finbb.fi/) is the coordinator of BBMRI-ERIC operations in Finland. The Finnish biobank data can be accessed through the Fingenious® services (https://site.fingenious.fi/en/) managed by FINBB.

The researchers would like to acknowledge all participants and investigators of the FinnGen study.

The **Framingham Heart Study** is conducted and supported by the National Heart, Lung, and Blood Institute (NHLBI) in collaboration with Boston University (Contract No. N01-HC-25195 and HHSN268201500001). This manuscript was not prepared in collaboration with investigators of the Framingham Heart Study and does not necessarily reflect the opinions or views of the Framingham Heart Study, Boston University, or NHLBI. Funding for SHARe Affymetrix genotyping was provided by NHLBI Contract N02-HL- 64278. Nancy L. Heard-Costa is supported by NIH/NHLBI HHSN268201500001 (Ramachandran). Further support was provided by P30AG066546, funded by the National Institute on Aging (HHS - NIH), awarded to University Of Texas Health Science Center Of San Antonio.

The Great Age study, Salus in Apulia study, was funded by Apulia Government and Italian Ministry of Health, under the Studies on Aging Network, at Italian Research Hospitals (IRCCS). The authors thank the MICOL Study group, the Salus in Apulia Research Team, and the General Practitioners of Castellana Grotte for the fundamental role in recruiting participants to this study.
The **Health, Aging and Body Composition Study** was supported by National Institute on Aging (NIA) Contracts N01-AG-6-2101; N01-AG-6-2103; N01-AG-6-2106; NIA grant R01-AG028050, and NINR grant R01-NR012459. This research was supported in part by the Intramural Research Program of the NIH, National Institute on Aging (NIA), National Institutes of Health, Department of Health and Human Services; project number ZO1 AG000535, as well as the National Institute of Neurological Disorders and Stroke.
The **Rotterdam Study** is funded by Erasmus Medical Center and Erasmus University, Rotterdam, Netherlands Organisation for the Health Research and Development (ZonMw), the Research Institute for Diseases in the Elderly (RIDE), the Ministry of Education, Culture and Science, the Ministry for Health, Welfare and Sports, the European Commission (DG XII), and the Municipality of Rotterdam. The authors are grateful to the study participants, the staff from the Rotterdam Study and the participating general practitioners and pharmacists. The generation and management of GWAS genotype data for the Rotterdam Study (RS I, RS II, RS III) was executed by the Human Genotyping Facility of the Genetic Laboratory of the Department of Internal Medicine, Erasmus MC, Rotterdam, The Netherlands. The GWAS datasets are supported by the Netherlands Organisation of Scientific Research NWO Investments (nr. 175.010.2005.011, 911-03-012), the Genetic Laboratory of the Department of Internal Medicine, Erasmus MC, the Research Institute for Diseases in the Elderly (014-93-015; RIDE2), the Netherlands Genomics Initiative (NGI)/Netherlands Organisation for Scientific Research (NWO) Netherlands Consortium for Healthy Aging (NCHA), project nr. 050-060-810. We thank Pascal Arp, Mila Jhamai, Marijn Verkerk, Lizbeth Herrera and Marjolein Peters, MSc, and Carolina Medina-Gomez, MSc, for their help in creating the GWAS database, and Karol Estrada, PhD, Yurii Aulchenko, PhD, and Carolina Medina-Gomez, MSc, for the creation and analysis of imputed data.
TwinsUK: The authors of this paper wish to express our appreciation to all study participants of the TwinsUK cohort. TwinsUK is funded by the Wellcome Trust, Medical Research Council, European Union, the National Institute for Health Research (NIHR)-funded BioResource, Clinical Research Facility and Biomedical Research Centre based at Guy's and St Thomas' NHS Foundation Trust in partnership with King's College London. HRRW is funded by Action on Hearing Loss, CJS is funded under a grant from the Chronic Disease Research Foundation (CDRF). FMKW is supported by Arthritis Research UK grant number 20682.
The **Women's Genome Health Study** is supported by the National Heart, Lung, and Blood Institute (HL043851 and HL080467) and the National Cancer Institute (CA047988 and UM1CA182913), with funding for genotyping provided by Amgen.

We are also thankful to all patients and participants in the various cohorts, and Fanny Stulz for graphic support. UK Biobank data has been used under the project # 11516. The computations and data handling were enabled by resources provided by the Swedish National Infrastructure for Computing (SNIC) at SNIC/UPPMAX partially funded by the Swedish Research Council through grant agreement no. 2018-05973. We acknowledge The Swedish Twin Registry for access to data. The Swedish Twin Registry is managed by Karolinska Institutet and receives funding through the Swedish Research Council under the grant no 2017-00641.

Supplementary references

1. Wesdorp, M.*, et al.* Heterozygous missense variants of LMX1A lead to nonsyndromic hearing impairment and vestibular dysfunction. *Hum Genet* **137**, 389-400 (2018).
2. Liu, Y.*, et al.* Critical role of spectrin in hearing development and deafness. *Sci Adv* **5**, eaav7803 (2019).
3. Xu, F., Shan, S., Sommerlad, S., Seddon, J.M. & Brenig, B. A Missense Mutation in the KLF7 Gene Is a Potential Candidate Variant for Congenital Deafness in Australian Stumpy Tail Cattle Dogs. *Genes (Basel)* **12**(2021).
4. Vona, B.*, et al.* A biallelic variant in CLRN2 causes non-syndromic hearing loss in humans. *Hum Genet* **140**, 915-931 (2021).
5. Manji, S.S.*, et al.* A mutation in synaptojanin 2 causes progressive hearing loss in the ENU-mutagenised mouse strain Mozart. *PLoS One* **6**, e17607 (2011).
6. Stover, E.H.*, et al.* Novel ATP6V1B1 and ATP6V0A4 mutations in autosomal recessive distal renal tubular acidosis with new evidence for hearing loss. *J Med Genet* **39**, 796-803 (2002).
7. Han, S.Y., Kim, S., Shin, D.H., Cho, J.H. & Nam, S.I. The Expression of AGO2 and DGCR8 in Idiopathic Sudden Sensorineural Hearing Loss. *Clin Exp Otorhinolaryngol* **7**, 269-274 (2014).
8. Uthaiah, R.C. & Hudspeth, A.J. Molecular anatomy of the hair cell's ribbon synapse. *J Neurosci* **30**, 12387-12399 (2010).
9. Ohlemiller, K.K.*, et al.* Progression of cochlear and retinal degeneration in the tubby (rd5) mouse. *Audiol Neurootol* **2**, 175-185 (1997).
10. Du, T.T.*, et al.* LMO7 deficiency reveals the significance of the cuticular plate for hearing function. *Nat Commun* **10**, 1117 (2019).
11. Fredrich, M. & Illing, R.B. MMP-2 is involved in synaptic remodeling after cochlear lesion. *Neuroreport* **21**, 324-327 (2010).
12. Wu, J.*, et al.* Matrix metalloproteinase-2 and -9 contribute to functional integrity and noiseinduced damage to the blood-labyrinth-barrier. *Mol Med Rep* **16**, 1731-1738 (2017).
13. Shin, J.B.*, et al.* The R109H variant of fascin-2, a developmentally regulated actin crosslinker in hair-cell stereocilia, underlies early-onset hearing loss of DBA/2J mice. *J Neurosci* **30**, 9683-9694 (2010).
14. Wang, Y.*, et al.* Blast-induced hearing impairment in rats is associated with structural and molecular changes of the inner ear. *Sci Rep* **10**, 10652 (2020).
15. Asamura, K., Abe, S., Fukuoka, H., Nakamura, Y. & Usami, S. Mutation analysis of COL9A3, a gene highly expressed in the cochlea, in hearing loss patients. *Auris Nasus Larynx* **32**, 113-117 (2005).
16. Carlton, A.J.*, et al.* Loss of Baiap2l2 destabilizes the transducing stereocilia of cochlear hair cells and leads to deafness. *J Physiol* **599**, 1173-1198 (2021).
17. Harris, T.B., Launer, L.J., Eiriksdottir, G., Kjartansson, O., Jonsson, P.V., Sigurdsson, G., Thorgeirsson, G., Aspelund, T., Garcia, M.E., Cotch, M.F., et al. (2007). Age, Gene/Environment Susceptibility-Reykjavik Study: multidisciplinary applied phenomics. Am J Epidemiol 165, 1076-1087.
18. Pedersen, D.A., Larsen, L.A., Nygaard, M., Mengel-From, J., McGue, M., Dalgard, C., Hvidberg, L., Hjelmborg, J., Skytthe, A., Holm, N.V., et al. (2019). The Danish Twin Registry: An Updated Overview. Twin Res Hum Genet 22, 499-507.

19. Leitsalu, L., Alavere, H., Tammesoo, M.L., Leego, E., and Metspalu, A. (2015). Linking a population biobank with national health registries-the estonian experience. J Pers Med 5, 96-106.
20. Loh, P.R., Danecek, P., Palamara, P.F., Fuchsberger, C., Y, A.R., H, K.F., Schoenherr, S., Forer, L., McCarthy, S., Abecasis, G.R., et al. (2016). Reference-based phasing using the Haplotype Reference Consortium panel. Nature genetics 48, 1443-1448.
21. Browning, B.L., Zhou, Y., and Browning, S.R. (2018). A One-Penny Imputed Genome from Next-Generation Reference Panels. Am J Hum Genet 103, 338-348.
22. Browning, S.R., and Browning, B.L. (2007). Rapid and accurate haplotype phasing and missing-data inference for whole-genome association studies by use of localized haplotype clustering. Am J Hum Genet 81, 1084-1097.
23. Mitt, M., Kals, M., Parn, K., Gabriel, S.B., Lander, E.S., Palotie, A., Ripatti, S., Morris, A.P., Metspalu, A., Esko, T., et al. (2017). Improved imputation accuracy of rare and low-frequency variants using population-specific high-coverage WGS-based imputation reference panel. Eur J Hum Genet 25, 869-876.
24. Zhou, W., Nielsen, J.B., Fritsche, L.G., Dey, R., Gabrielsen, M.E., Wolford, B.N., LeFaive, J., VandeHaar, P., Gagliano, S.A., Gifford, A., et al. (2018). Efficiently controlling for case-control imbalance and sample relatedness in large-scale genetic association studies. Nature genetics 50, 1335-1341.
25. Dawber, T.R., Kannel, W.B., and Lyell, L.P. (1963). An approach to longitudinal studies in a community: the Framingham Study. Ann N Y Acad Sci 107, 539-556.
26. Feinleib, M., Kannel, W.B., Garrison, R.J., McNamara, P.M., and Castelli, W.P. (1975). The Framingham Offspring Study. Design and preliminary data. Prev Med 4, 518-525.
27. Splansky, G.L., Corey, D., Yang, Q., Atwood, L.D., Cupples, L.A., Benjamin, E.J., D'Agostino, R.B., Sr., Fox, C.S., Larson, M.G., Murabito, J.M., et al. (2007). The Third Generation Cohort of the National Heart, Lung, and Blood Institute's Framingham Heart Study: design, recruitment, and initial examination. Am J Epidemiol 165, 1328-1335.
28. Esko, T., Mezzavilla, M., Nelis, M., Borel, C., Debniak, T., Jakkula, E., Julia, A., Karachanak, S., Khrunin, A., Kisfali, P., et al. (2013). Genetic characterization of northeastern Italian population isolates in the context of broader European genetic diversity. Eur J Hum Genet 21, 659-665.
29. Girotto, G., Pirastu, N., Sorice, R., Biino, G., Campbell, H., d'Adamo, A.P., Hastie, N.D., Nutile, T., Polasek, O., Portas, L., et al. (2011). Hearing function and thresholds: a genome-wide association study in European isolated populations identifies new loci and pathways. J Med Genet 48, 369-374.
30. Sardone, R., Battista, P., Donghia, R., Lozupone, M., Tortelli, R., Guerra, V., Grasso, A., Griseta, C., Castellana, F., Zupo, R., et al. (2020). Age-Related Central Auditory Processing Disorder, MCI, and Dementia in an Older Population of Southern Italy. Otolaryngol Head Neck Surg 163, 348-355.
31. Ikram, M.A., Brusselle, G., Ghanbari, M., Goedegebure, A., Ikram, M.K., Kavousi, M., Kieboom, B.C.T., Klaver, C.C.W., de Knegt, R.J., Luik, A.I., et al. (2020). Objectives, design and main findings until 2020 from the Rotterdam Study. Eur J Epidemiol 35, 483-517.
32. Zagai, U., Lichtenstein, P., Pedersen, N.L., and Magnusson, P.K.E. (2019). The Swedish Twin Registry: Content and Management as a Research Infrastructure. Twin Res Hum Genet 22, 672-680.
33. Moayyeri, A., Hammond, C.J., Valdes, A.M., and Spector, T.D. (2013). Cohort Profile: TwinsUK and healthy ageing twin study. Int J Epidemiol 42, 76-85.

34. Ridker, P.M., Chasman, D.I., Zee, R.Y., Parker, A., Rose, L., Cook, N.R., Buring, J.E., and Women's Genome Health Study Working, G. (2008). Rationale, design, and methodology of the Women's Genome Health Study: a genome-wide association study of more than 25,000 initially healthy american women. Clin Chem 54, 249-255.
