# Supplementary material for: Genome-wide association meta-analysis identifies 48 risk variants and highlights the role of the stria vascularis in hearing loss
Source: Am J Hum Genet. 2022 May 16;109(6):1077–91. doi: 10.1016/j.ajhg.2022.04.010 (PMC9247887; doi:10.1016/j.ajhg.2022.04.010)
Supplement: Document S2. Article plus supplemental information [file mmc3.pdf]

ARTICLE

Genome-wide association meta-analysis identifies 48 risk variants and highlights the role of the stria vascularis in hearing loss

Authors

Natalia Trpchevska, Maxim B. Freidin,
Linda Broer, ..., Christopher R. Cederroth,
Frances M.K. Williams, Andries Paul Nagtegaal

Correspondence

christopher.cederroth@ki.se

Trpchevska et al., 2022, The American Journal of Human Genetics *109*, 1077–1091
June 2, 2022 © 2022 The Authors.
https://doi.org/10.1016/j.ajhg.2022.04.010

Genome-wide association meta-analysis identifies 48 risk variants and highlights the role of the stria vascularis in hearing loss

Natalia Trpchevska,[1,55] Maxim B. Freidin,[2,55] Linda Broer,[3,55] Berthe C. Oosterloo,[4] Shuyang Yao,[5] Yitian Zhou,[1] Barbara Vona,[6,7,8] Charles Bishop,[9] Argyro Bizaki-Vallaskangas,[10,11] Barbara Canlon,[1] Fabio Castellana,[12] Daniel I. Chasman,[13,14] Stacey Cherny,[15] Kaare Christensen,[16,17,18] Maria Pina Concas,[19] Adolfo Correa,[20] Ran Elkon,[21] Estonian Biobank Research Team,[22] Jonas Mengel-From,[16,17] Yan Gao,[20,23] Anne B.S. Giersch,[24] Giorgia Girotto,[19,25] Alexander Gudjonsson,[26] Vilmundur Gudnason,[26,27] Nancy L. Heard-Costa,[28,29] Ronna Hertzano,[30,31,32] Jacob v.B. Hjelmborg,[16] Jens Hjerling-Leffler,[33] Howard J. Hoffman,[34] Jaakko Kaprio,[35]

(Author list continued on next page)

Summary

Hearing loss is one of the top contributors to years lived with disability and is a risk factor for dementia. Molecular evidence on the cellular origins of hearing loss in humans is growing. Here, we performed a genome-wide association meta-analysis of clinically diagnosed and self-reported hearing impairment on 723,266 individuals and identified 48 significant loci, 10 of which are novel. A large proportion of associations comprised missense variants, half of which lie within known familial hearing loss loci. We used single-cell RNA-sequencing data from mouse cochlea and brain and mapped common-variant genomic results to spindle, root, and basal cells from the stria vascularis, a structure in the cochlea necessary for normal hearing. Our findings indicate the importance of the stria vascularis in the mechanism of hearing impairment, providing future paths for developing targets for therapeutic intervention in hearing loss.

Introduction

The number of people with mild-to-complete hearing impairment is projected to increase to an estimated 2.45 billion worldwide by 2050, principally driven by age-related hearing impairment (ARHI).[1] Hearing impairment is ranked third for causes of global years lived with disability (YLDs) across all ages and the leading cause of YLDs in those older than 70, as compared with all other disease categories.[1] The overall global cost of unaddressed hearing loss exceeds $981 billion annually.[2] ARHI has been associated with social withdrawal, depression, anxiety, as well as cognitive decline and dementia.[3] There is no preventive treatment for hearing decline and therapeutics are currently available only in the form of hearing aids or cochlear implants. Moreover, the impact of untreated hearing loss remains underestimated as governmental and industry incentives are still very low in comparison to other diseases of equal prevalence.[4]

[1]Department of Physiology and Pharmacology, Karolinska Institutet, 17177 Stockholm, Sweden; [2]Department of Twin Research and Genetic Epidemiology, King's College London, London, UK; [3]Department of Internal Medicine, Erasmus Medical Center, 3015 CE Rotterdam, the Netherlands; [4]Department of Otorhinolaryngology, Erasmus Medical Center, 3015 CE Rotterdam, the Netherlands; [5]Department of Medical Epidemiology and Biostatistics, Karolinska Institutet, 17177 Stockholm, Sweden; [6]Institute of Human Genetics, University Medical Center Göttingen, 37073 Göttingen, Germany; [7]Institute for Auditory Neuroscience and InnerEarLab, University Medical Center Göttingen, 37075 Göttingen, Germany; [8]Department of Otolaryngology–Head & Neck Surgery, University of Tübingen Medical Center, 72076 Tübingen, Germany; [9]Department of Otolaryngology and Communicative Sciences, The University of Mississippi Medical Center, Jackson, MS 39216, USA; [10]Department of Otolaryngology, University of Tampere, 33100 Tampere, Finland; [11]Pirkanmaan Sairaanhoitopiiri, 33520 Tampere, Finland; [12]Unit of Data Sciences and Technology Innovation for Population Health, National Institute of Gastroenterology "Saverio de Bellis", Research Hospital, Castellana Grotte, 70124 Bari, Italy; [13]Division of Preventative Medicine, Brigham and Women's Hospital, Harvard Medical School, Boston, MA 02115, USA; [14]Broad Institute of MIT and Harvard, Cambridge, MA 02142, USA; [15]Department of Anatomy and Anthropology and Department of Epidemiology and Preventive Medicine, Sackler Faculty of Medicine, Tel Aviv University, 69978 Tel Aviv, Israel; [16]The Danish Twin Registry, Department of Public Health, University of Southern Denmark, 5000 Odense C, Denmark; [17]Department of Clinical Genetics, Odense University Hospital, 5000 Odense C, Denmark; [18]Department of Clinical Biochemistry and Pharmacology, Odense University Hospital, 5000 Odense C, Denmark; [19]Institute for Maternal and Child Health — IRCCS, Burlo Garofolo, 34127 Trieste, Italy; [20]Jackson Heart Study, The University of Mississippi Medical Center, Jackson, MS 39216, USA; [21]Department of Human Molecular Genetics & Biochemistry, Sackler School of Medicine, Tel Aviv University, 69978 Tel Aviv, Israel; [22]Estonian Genome Centre, Institute of Genomics, University of Tartu, Tartu, Estonia; [23]Department of Population Health Science, University of Mississippi Medical Center, Jackson, MS 39216, USA; [24]Department of Pathology, Brigham and Women's Hospital, Harvard Medical School, Boston, MA 02115, USA; [25]Department of Medicine, Surgery and Health Sciences, University of Trieste, 34139 Trieste, Italy; [26]Icelandic Heart Association, 201 Kopavogur, Iceland; [27]Faculty of Medicine, University of Iceland, 101 Reykjavik, Iceland; [28]Department of Neurology, Boston University School of Medicine, Boston, MA 02118, USA; [29]Framingham Heart Study, Framingham, MA 01702, USA; [30]Department of Otorhinolaryngology-Head and Neck Surgery, University of Maryland Baltimore, Baltimore, MD 21201, USA; [31]Department of Anatomy and Neurobiology, University of Maryland Baltimore, Baltimore, MD 21201, USA; [32]Institute for Genome Sciences, University of Maryland Baltimore, Baltimore, MD 21201, USA; [33]Department of Medical Biochemistry and Biophysics, Karolinska Institutet, 17177 Stockholm, Sweden; [34]Division of Scientific Programs, Epidemiology and Statistics Program, National Institute on Deafness and Other Communications Disorders (NIDCD), NIH, Bethesda, MD 20892, USA; [35]Institute for Molecular Medicine Finland (FIMM), University

(Affiliations continued on next page)

© 2022 The Authors. This is an open access article under the CC BY license (http://creativecommons.org/licenses/by/4.0/).

Johannes Kettunen,[36,37,38] Kristi Krebs,[22] Anna K. Kähler,[5] Francois Lallemend,[39] Lenore J. Launer,[40] I-Min Lee,[13] Hampton Leonard,[41,42,43] Chuan-Ming Li,[34] Hubert Lowenheim,[8] Patrik K.E. Magnusson,[5] Joyce van Meurs,[3] Lili Milani,[22] Cynthia C. Morton,[14,44,45] Antti Mäkitie,[46] Mike A. Nalls,[41,42,43] Giuseppe Giovanni Nardone,[19] Marianne Nygaard,[16,17] Teemu Palviainen,[35] Sheila Pratt,[47] Nicola Quaranta,[48] Joel Rämö,[35] Elmo Saarentaus,[35] Rodolfo Sardone,[12] Claudia L. Satizabal,[28,29,49] John M. Schweinfurth,[9] Sudha Seshadri,[28,29,49] Eric Shiroma,[50] Eldad Shulman,[21] Eleanor Simonsick,[51] Christopher Spankovich,[9] Anke Tropitzsch,[8] Volker M. Lauschke,[1] Patrick F. Sullivan,[5,52] Andre Goedegebure,[4] Christopher R. Cederroth,[1,53,54,56,*] Frances M.K. Williams,[2,56] and Andries Paul Nagtegaal[4,56]

Hearing thresholds tend to deteriorate gradually with age and ARHI is typically more pronounced in the higher frequencies. Knowledge of the pathophysiological mechanisms of hearing loss derives primarily from animal studies (particularly mouse models[5]), as well as clinical research on specific families with hearing loss.[6] Hearing loss is moderately heritable, with recent studies attributing 36%–70% of the variation in the heritability of hearing impairment to additive genetic effects.[7,8] Large genome-wide association studies (GWASs) have been recently conducted: UK Biobank (n = 87,056 individuals with self-reported hearing difficulty) revealed 44 independent loci associated with self-reported hearing difficulty and confirmed that hearing loss is a complex polygenic disorder.[9] A combined Icelandic cohort and UK Biobank (n = 121,934 individuals identified through pure-tone audiograms and self-reported hearing difficulty) yielded another 21 novel associations of which 13 were rare variants.[10] Kalra et al. performed a multi-trait analysis of GWASs (MTAG)[11] using UKB data from up to 337,000 participants with different hearing phenotypes and identified 8 novel hits supported with transcription data.[12] However, many loci were not replicated, which may be explained by differences in phenotyping (ICD diagnoses, self-report, hearing thresholds assessed by audiometry), imbalanced sample size with UK Biobank predominating, statistical power, or ancestral differences between samples. While early-onset genetic hearing loss is determined by monogenic factors, ARHI appearing in late adulthood develops from the interaction of environmental and polygenic factors.[13]

In order to gain fundamental knowledge on the genetic basis of hearing loss, we conducted a meta-analysis of 17 hearing loss GWASs using both ICD diagnoses and self-reported hearing loss. The latter has been demonstrated to be a good proxy for formal hearing assessment.[14] The study comprised 147,997 affected individuals and 575,269 control subjects including 60,941 affected individuals that were not in our previously published GWAS meta-analysis.[9] We compiled a dataset comprising multiple different European and US population-based cohorts (Figure 1A, Table S1).

Material and methods

Study design and phenotyping

Adult male and female participants were included from the following 17 population-based cohort studies: Age, Genes/Environment Susceptibility - Reykjavik (AGES; n = 3,134), the Danish Twin Registry (DTR; n = 1,314), the Estonian Genome Center at the University of Tartu (EGCUT; n = 55,523), FinnGen (n = 212,544), Framingham Heart Study (FHS; n = 2,536), Health Aging and Body Composition (HABC; n = 1,288), Italian Network of Genetic Isolates - Friuli Venezia Giulia (INGI-FVG; n = 339), the Rotterdam Study (RS, cohorts 1–3; n = 6,157), the Salus in Apulia study (SA; n = 1,780; formerly known as Great Age study), Screening Across the Lifespan Twin (SALT; n = 9,565, and SALTY - young; n = 5,133), Screening Twin Adults: Genes and Environment (STAGE; n = 8,345), TwinsUK (n = 5,125), UK Biobank (UKBB; n = 392,143), and the Women's Genome Health Study (WGHS; n = 18,340). All participants provided written informed consent; ethical approval was obtained locally. The declaration of Helsinki

of Helsinki, 00014 Helsinki, Finland; [36]Computational Medicine, Center for Life Course Health Research, Faculty of Medicine, University of Oulu, 90220 Oulu, Finland; [37]Biocenter Oulu, University of Oulu, 90220 Oulu, Finland; [38]Finnish Institute for Health and Welfare, 00271 Helsinki, Finland; [39]Department of Neuroscience, Karolinska Institutet, 17177 Stockholm, Sweden; [40]Laboratory of Epidemiology and Population Sciences, Intramural Research Program National Institute on Aging, Bethesda, MD 20892, USA; [41]Laboratory of Neurogenetics, National Institute on Aging, National Institutes of Health, Bethesda, MD 20892, USA; [42]Center for Alzheimer's and Related Dementias, National Institutes of Health, Bethesda, MD 20892, USA; [43]Data Tecnica International, Glen Echo, MD 20812, USA; [44]Department of Obstetrics and Gynecology and of Pathology, Brigham and Women's Hospital, Harvard Medical School, Boston, MA 02115, USA; [45]Manchester Centre for Audiology and Deafness, University of Manchester, Manchester M13 9PL, UK; [46]Department of Otorhinolaryngology - Head and Neck Surgery, University of Helsinki and Helsinki University Hospital, 00029 Helsinki, Finland; [47]Department of Communication Science & Disorders, University of Pittsburgh, Pittsburgh, PA 15260, USA; [48]Otolaryngology Unit, Department of Basic Medical Science, Neuroscience and Sense Organs, University of Bari Aldo Moro, 70121 Bari, Italy; [49]Glenn Biggs Institute for Alzheimer's & Neurodegenerative Diseases and Department of Population Health Sciences, University of Texas Health Sciences Center, San Antonio, TX 78229, USA; [50]Laboratory of Epidemiology and Population Sciences, National Institute on Aging, Baltimore, MD 21224, USA; [51]Longitudinal Studies Section, Translational Gerontology Branch, National Institute on Aging, Baltimore, MD 21224, USA; [52]Department of Genetics, University of North Carolina, Chapel Hill, NC 27516, USA; [53]National Institute for Health Research (NIHR) Nottingham Biomedical Research Centre, Nottingham University Hospitals NHS Trust, Ropewalk House, NG1 5DU Nottingham, UK; [54]Hearing Sciences, Division of Clinical Neuroscience, School of Medicine, University of Nottingham, NG7 2UH Nottingham, UK
[55]These authors contributed equally
[56]These authors contributed equally
*Correspondence: christopher.cederroth@ki.se
https://doi.org/10.1016/j.ajhg.2022.04.010.

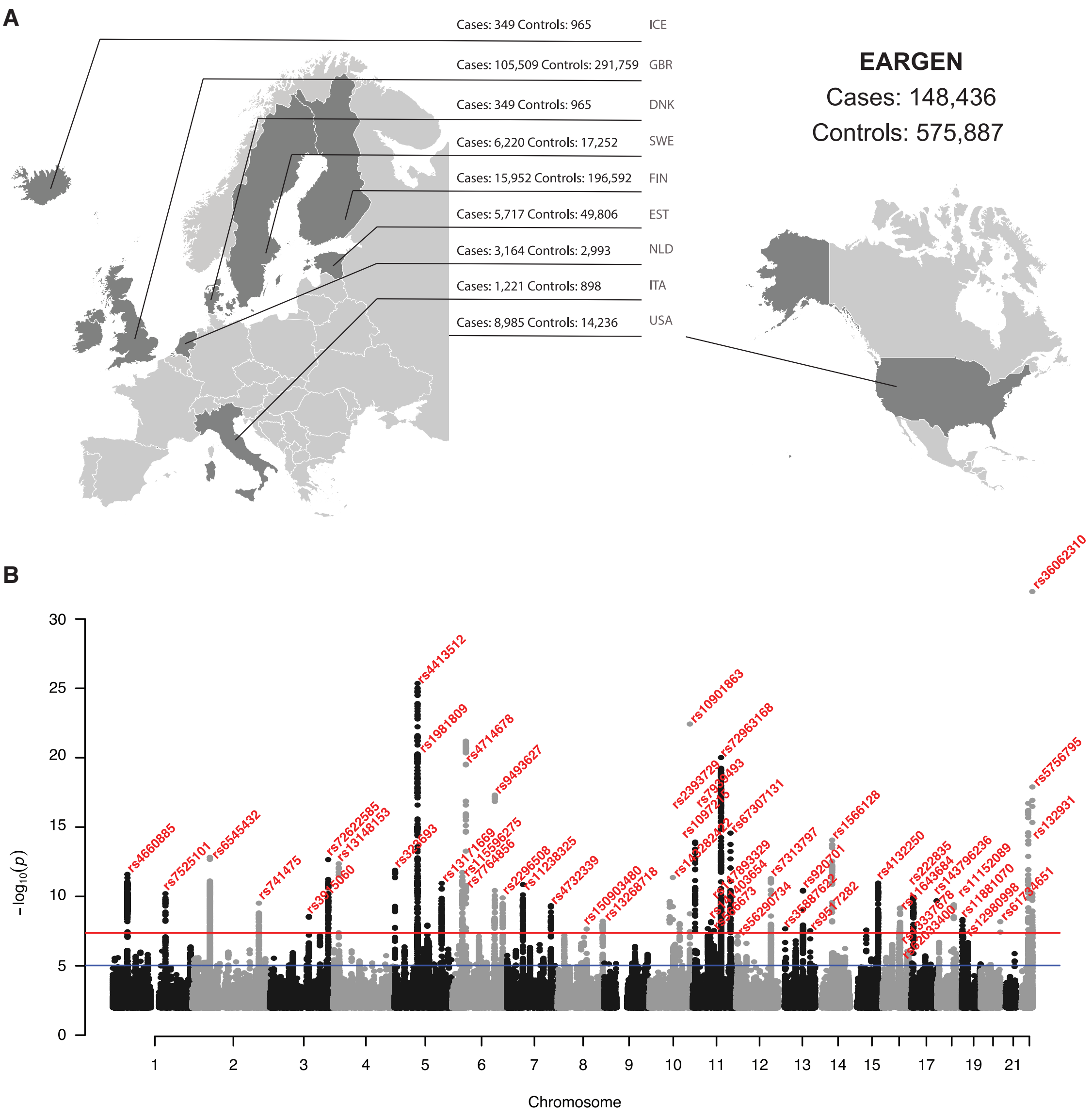

Figure 1. GWAS meta-analysis for ARHI (n = 723,266)
(A) Origin of the datasets used for the meta-analysis of the ARHI GWAS: 8 European countries and the United States.
(B) Manhattan plot displays all associations per variant ordered according to their genomic position on the x axis and showing the strength of the association with the $-\log_{10}$ transformed p values on the y axis. The threshold for genome-wide significance ($p < 5 \times 10^{-8}$) is indicated by the red line, while the blue line represents the suggestive threshold ($p < 1 \times 10^{-5}$).

was adhered to. Phenotype was based on ICD9 & 10 diagnoses of hearing loss (EGCUT and FinnGen) or questions on hearing loss (all other cohorts). Mean age overall was 59.6. A detailed description of phenotype definition case control distribution and age range for each study can be found in Table S1. Study details and cohort descriptions are available in supplemental methods. An umbrella ethics license was granted by the local ethics committee, Regionala etikprövningsnämnden in Stockholm (2015/2129-31/1). Informed consent was obtained from all participants. Individual ethical license from all contributing cohorts are included in supplemental methods.

Genome-wide association studies and meta-analysis

GWASs have been carried out for each cohort locally and summary statistics were collected for each study. Standardized quality control was performed using EasyQC software,[15] followed by meta-analysis using METAL software.[16] Briefly, the quality control steps with EasyQC were excluding monomorphic SNPs, SNP missingness <0.05, filtering out duplicate SNPs and SNPs with imputation score <0.5. After harmonizing allele coding and marker names, uniformed summary statistics were produced. LD score regression[17] was applied to estimate the impact of population stratification and other confounders on test-statistic inflation for cohorts with sample size $n > 5{,}000$ for QC-ed and harmonized GWAS data. The genome build was hg19.

Meta-analysis of GWAS summary statistics was conducted using an inverse-variance-weighted fixed-effect model in METAL.[16] To control for population stratification and other confounders, individual cohorts were adjusted for either genomic control (cohort with sample size >5,000) or intercept. A genome-wide significance threshold was defined as $p < 5 \times 10^{-8}$. Conditional and joint

association analysis (COJO) was carried out to reveal independent lead SNPs for genome-wide significant loci using GCTA software.[18] We randomly selected 50,000 individuals of European ancestry from UK Biobank as a reference sample for COJO. We examined whether the SNPs have been previously associated with hearing loss in a large-scale GWAS. A locus was designated "new" when LD with previous associated variants was <0.6. In the case of missense SNPs, the Combined Annotation Dependent Depletion (CADD) score (GRCh37-v1.6) was used to estimate the deleterious effect of the SNP.[19]

Gene prioritization and pathway analysis

For gene prioritization in the genome-wide significant loci, we used MAGMA[20] (significance threshold $p < 2.66 \times 10^{-6}$) implemented in FUMA[21] and VEGAS2 software[22] (Bonferroni corrected $p < 0.05$). We used the offline version of VEGAS2 and analyzed the most associated 10 SNPs flanking 10 kb upstream and downstream the genes. The list of genes was obtained from the VEGAS2 website (https://vegas2.qimrberghofer.edu.au/glist-hg19) and included 26,056 genes. Given that the number of genes in the output would depend on the analysis parameters, we chose to correct for multiple testing assuming 20,000 independent tests by Bonferroni approach.

Genes within 500 Mb from the top SNP were checked for any known association with hearing loss in either humans or mice. For that purpose, existing literature and the website of the International Mouse Phenotyping Consortium (www.mousephenotype.org) were consulted.[23] The Shared Inner Ear Laboratory Database (SHIELD)[24] was used to examine whether candidate genes were expressed in inner and outer hair cells of the cochlea in adult mice (P25–P30), designated positive when expression levels (measured by fluorescent intensity readings) exceeded 10.9, as described in the referenced paper.[25]

We used VEGAS2 for pathway analysis based on the results of gene prioritization as described above and the list of pathways provided as part of VEGAS2 distribution (https://vegas2.qimrberghofer.edu.au/biosystems20160324.vegas2pathSYM).[26]

Variant analysis

All variants identified in genes of interest in the gnomAD v.2.1.1 were downloaded, and minor allele frequencies (MAFs) were filtered to select for variants that were common (MAF $\geq$1%) in the non-Finnish European (EUR) population. Transcripts were selected based on consensus with the Deafness Variation Database transcript catalog,[27] where possible, or otherwise we used the longest transcript according to Ensembl. Variant effects were analyzed using an array of partially orthogonal computational prediction algorithms, PolyPhen-2,[28] CADD,[19] DANN,[29] PROVEAN,[30] REVEL,[31] VEST3,[32] and Eigen,[33] that consider genetic, evolutionary, structural, and biochemical information to infer variant pathogenicity and deleteriousness. The individual algorithmic assessments were aggregated into a consensus ensemble score normalized to the range from zero (variant unanimously predicted to be deleterious) to one (unanimously predicted to be benign). The secondary structure of GJB2 was obtained from the Protein Data Bank (PDB: 2ZW3) and the structural consequences of *GJB2* variants were modeled using PyMOL v..1.1.

Fine-mapping was carried out using CAUSALdb-finemapping-pip pipeline (https://github.com/mulinlab/CAUSALdb-finemapping-pip). We analyzed 1 Mb regions surrounding lead SNPs in each genome-wide significant locus. The output includes credible sets and posterior probabilities for SNPs to be causal as per PAINTOR, CAVIARBF, and FINEMAP algorithms.

Genetic correlations

In LD Hub[34] we used LD score regression to estimate the genetic correlation between hearing loss and a range of other disorders and traits, to evaluate the extent of shared genetic architecture based on common gene variants and hypothesize about association with potential risk factors. After excluding all UK Biobank-based phenotypes, the list comprised 256 phenotypes. Significance was thus set at $p < 2 \times 10^{-04}$ after Bonferroni correction.

In the internal genome-wide association library (Omnibus data) collated by the Psychiatric Genomics Consortium, we used LDSC to get SNP-based genetic correlation between hearing loss and psychiatric and anthropometric traits.

Expression data sets

We obtained expression specificity data in 37 GTEx v8[35] human tissues processed by previous research.[36] Briefly, the following tissue filters were applied: (1) tissues with fewer than 100 donors, (2) non-natural tissues (e.g., cancer tissue and cell lines), and (3) testis tissues (expression outlier). Since GTEx does not contain cochlear data, we sought expression data from 36,616 cells originating from 2 datasets from adult mouse cochlea (post-natal day 60, CBA/CaJ) published by Milon et al.[37] (one from the stria vascularis and the other from spiral ganglion neuron [SGN]) summing to 15 different cell types (Table S2). Since these two datasets were genotyped using exactly the same technique in the same technical infrastructure, we merged them first by aggregating the count per gene per cell type and normalized to 1 TPM per cell type to account for the variation of cell counts per cell type in each dataset, while preserving the relative expression pattern per cell type (Table S2). Monocytes and neutrophils were found in both the stria vascularis and in the SGN data, and the correlations of normalized expression between the two datasets were high (0.90 for monocytes and 0.98 for neutrophils based on 15,798 genes), supporting the appropriateness of the merging step. Additionally, since the data from Milon et al. did not contain data from the organ of Corti (e.g., hair cells and and Deiters' cells), we relied on single-cell data extracted by Ranum et al.[38] (post-natal day 15, C3HeB/FeJ). We note that the murine cochlea is functionally mature at P14. Finally, we also used expression data from Zeisel et al.[39] consisting in 160,796 cells of 39 broad cell types sampled from 19 regions in the entire mouse neural system (post-natal day P12–P30, as well as 6 and 8 weeks old, CD-1 and Swiss) that were processed to get cell type expression specificity[36] (Table S2). Only genes with 1:1 orthology between human and mouse were preserved for calculating the expression specificity.

Calculation of cell-type expression specificity

We processed and calculated the expression specificity as previously described.[36] Briefly, in each tissue expression dataset (i.e., organ of Corti,[38] stria vascularis/SGNs,[37] and neural tissue[39]), we first aggregated the count per gene per cell type and excluded genes that are (1) not expressed in any cell type, (2) with duplicated identifier, or (3) not 1:1 orthologous between mouse and human. We then normalized the expression to 1 TPM (transcripts per million) per cell type. Next, gene expression specificity was calculated per gene per cell type as:

$$Specificity = \frac{Normalized\ expression\ in\ the\ cell\ type}{Sum\ of\ normalized\ expression\ in\ all\ cell\ types}$$

**Table 1. Summary statistics of significantly associated loci identified in the genome-wide association meta-analysis of AHRI**										
**SNP**	**Chr**	**Pos (hg19)**	**EA**	**OA**	**EAF**	**Beta**	**SE**	**p value**	**Direction[a]**	**Locus annotation**
rs4660885	1	46243756	a	G	0.4344	−0.007	9.00E−04	3.74E−12	–+–?——?-+——	IPP-[x]-MAST2
rs7525101	1	165109131	t	C	0.4424	0.006	9.00E−04	8.64E−11	++++-?++++?–++++	PBX1—[x]-LMX1A
rs6545432	2	54817683	a	G	0.5091	0.007	9.00E−04	2.36E−13	++++++?-++?++++++	[SPTBN1] intronic
rs741475	2	208087139	t	C	0.5771	−0.006	9.00E−04	4.02E−10	———?-+–?————	KLF7-[x]–CREB1
rs3915060	3	121712980	t	C	0.7272	−0.006	1.00E−03	3.96E−09	+-+–?+-+-?+++—	[ILDR1] intronic
rs72622585[b]	3	181992315	t	C	0.8252	0.009	1.30E−03	3.41E−13	++-++–+++???-+++	SOX2—[x]—ATP11B
rs13148153	4	17517558	t	c	0.1342	0.010	1.40E−03	2.64E−12	+++++?++++???-+++	[CLRN2] intronic
rs323693	5	2562593	t	c	0.882	−0.010	1.40E−03	1.91E−12	–++-?–++?————	IRX4—[x]–IRX2
rs1981809	5	72920029	t	c	0.4526	−0.009	9.00E−04	1.36E−20	———+?-+-?+-+—	UTP15-[x]-ARHGEF28
rs4413512[b]	5	73077349	a	g	0.5289	−0.010	9.00E−04	1.28E−25	———?–+-?-+——	[ARHGEF28] intronic
rs13171669	5	148601243	a	g	0.5682	−0.006	9.00E−04	1.61E−11	———?+++-?————	[ABLIM3] intronic
rs115596275	6	32420218	c	g	0.0213	0.024	3.50E−03	2.73E−12	?+???++-?+???++++	HLA-DRA-[x]-HLA-DRB5
rs7764856[b]	6	32680640	a	t	0.3435	0.007	1.00E−03	1.10E−10	?+-??+++?-???++++	HLA-DQB1-[x]-HLA-DQA2
rs4714678	6	43342591	a	g	0.4031	−0.009	9.00E−04	7.20E−20	+-++–+—?+———	ZNF318-[x]-ABCC10
**rs9493627**	**6**	**133789728**	**a**	**g**	**0.3191**	**0.009**	**1.00E−03**	**9.56E−18**	**++++-+?+-+?-+++++**	**[EYA4] G>S**
rs2296508	6	158497717	t	c	0.4795	−0.006	9.00E−04	4.34E−10	–+++??-+-?++——	[SYNJ2] V>V
rs11238325	7	50853151	t	c	0.7315	0.007	1.00E−03	1.97E−11	++++-?++++?+-++++	[GRB10] intronic
rs4732339	7	138491839	a	g	0.5864	0.006	9.00E−04	6.10E−10	++++-?++-+?+-++++	TMEM213-[x]-KIAA1549
rs150903480	8	91376248	a	g	0.0114	−0.025	4.40E−03	2.70E−08	?-?+-+–+-?–+—	[LINC00534]
rs13268718	8	141687200	t	g	0.5072	−0.005	9.00E−04	7.47E−09	+++-++-++-?–++–	[PTK2] intronic
rs2393729	10	63837016	t	c	0.4218	−0.006	9.00E−04	3.07E−10	-++–??-+-?+++—	[ARID5B] intronic
**rs143282422**	**10**	**73377112**	**a**	**g**	**0.0112**	**0.032**	**4.60E−03**	**6.27E−12**	**?+?++?++++???++++**	**[CDH23] A>T**
rs1097215	10	94787804	a	g	0.4752	−0.005	9.00E−04	1.11E−08	-+-+——+-?–+—	[EXOC6] intronic
rs10901863	10	126812270	t	c	0.2683	0.011	1.10E−03	9.30E−23	++-++?++-+?++++++	[CTBP2] 5′ UTR
rs7939493	11	8073610	a	t	0.1911	−0.009	1.20E−03	2.47E−14	-++–?-+—??——	[TUB] intronic
rs141403654	11	47715487	a	t	0.9837	−0.022	3.90E−03	2.52E−08	+-?–?-+——————+-	[AGBL2] intronic
rs147893329[b]	11	57735006	c	g	0.0107	0.028	4.80E−03	8.17E−09	+??++?++–+++++++	CTNND1–[x]-OR9Q1
rs566673	11	66401373	t	g	0.5339	−0.005	9.00E−04	3.41E−08	–+-++?+———————	RBM14-[x]-RBM4
rs72963168	11	88943035	t	c	0.7254	−0.009	1.00E−03	3.73E−19	-++–?—————+——	[TYR] intronic
rs67307131	11	118480223	t	c	0.654	−0.008	1.00E−03	4.62E−15	-+–+?—?-??+-?-	[PHLDB1] intronic
rs7313797[b]	12	109896165	t	c	0.5604	−0.006	9.00E−04	7.38E−12	++++++?++-?————	[KCTD10] intronic
**rs35887622[b]**	**13**	**20763620**	**a**	**g**	**0.9854**	**−0.022**	**3.90E−03**	**2.59E−08**	**?-?-++++++-?————**	**[GJB2] M>T**
rs920701	13	76417101	t	c	0.6357	−0.006	1.00E−03	5.06E−11	-+++–?-+-?————	[LMO7] intronic
rs9517282[b]	13	99059183	a	c	0.548	−0.005	9.00E−04	3.54E−08	-+-+-?——?-+——	[FARP1] intronic
rs1566128	14	52514981	a	g	0.4126	0.007	9.00E−04	1.42E−14	+–+++?+++?-+++++	[NID2] intronic
rs4132250	15	89229000	c	g	0.778	0.007	1.10E−03	3.18E−11	+++++?++-+?++++++	ISG20-[x]–ACAN
rs62033400	16	53811788	a	g	0.6044	0.005	9.00E−04	4.52E−08	—+++-+-++-+-+++	[FTO] intronic
rs11643684	16	55490167	t	g	0.2031	−0.007	1.10E−03	2.26E−09	–+–?—————+——	IRX6–[x]-MMP2
rs13337678[b]	16	56379937	t	c	0.5711	−0.005	9.00E−04	3.72E−08	++-++??——+———	[GNAO1] 3′ UTR
rs222835	17	7134129	a	g	0.4247	0.006	9.00E−04	4.81E−10	-++++??+++?-+++++	[DVL2] intronic
**rs143796236**	**17**	**79495969**	**t**	**c**	**0.0076**	**0.035**	**5.60E−03**	**2.73E−10**	**?+?++?+??-???++++**	**[FSCN2] H>Y**

(*Continued on next page*)

Table 1. *Continued*

SNP	Chr	Pos (hg19)	EA	OA	EAF	Beta	SE	p value	Direction[a]	Locus annotation
rs11152089	18	52625943	t	c	0.2134	0.007	1.10E−03	9.24E−10	+-++++++++?++-+++	[CCDC68] 5′ UTR
rs11881070	19	2389140	t	c	0.2882	−0.006	1.00E−03	5.72E−09	——+??—?+-+—	SPPL2B-[x]-TMRPS9
**rs12980998[b]**	**19**	**4217510**	**a**	**t**	**0.8135**	**−0.007**	**1.20E−03**	**1.02E−07**	**+—+?-?+-???——**	**[ANKRD24] T>S**
**rs61734651[b]**	**20**	**61451332**	**t**	**c**	**0.0721**	**0.011**	**1.90E−03**	**8.16E−09**	**?+-+-+++++?++++++**	**[COL9A3] R>W**
**rs5756795**	**22**	**38122122**	**t**	**c**	**0.5419**	**−0.008**	**9.00E−04**	**3.65E−17**	**+-+++———?++——**	**[TRIOBP] F>I**
rs132931	22	38487526	a	g	0.5869	−0.007	0.0009	1.59E−14	++—+?—?————	[BAIAP2L2] intronic
**rs36062310**	**22**	**50988105**	**a**	**G**	**0.0427**	**0.027**	**0.0023**	**4.25E−32**	**++++++—+?+-++++**	**[KLHDC7B] V>M**

48 loci significantly ($p < 5 \times 10^{-8}$) associated with hearing loss. Abbreviations: Chr, chromosome; Pos, genomic position (bp); EA, effect allele; OA, other allele; EAF, effect allele frequency; Beta, effect size for EA; SE, standard error of effect size. Missense SNPs are listed in bold, with corresponding amino acid change.
[a]Summary of effect direction for each study: + is risk increasing, - is risk decreasing, ? indicated the SNP was not present in sample cohort sequence: AGES, SA, FVG, RS2, RS3, DTR, HABC, FHS, RS1, EGC, SALT, STAGE, SALTY, TWINSUK, WGHS, FinnGen, UKBB. Locus annotation: single dash (-), <100 kb; double dash (–), 100–500 kb; triple dash (—), >500 kb.
[b]No previous association with hearing loss in a GWAS.

Specificity ranges from 0 to 1; a higher value indicates that the gene is more specific to the corresponding cell types compared to its expression profiles across all included cell types. We selected genes with the top 10% specificity values in each cell type as the gene list for the cell type that was used for the heritability enrichment analyses.

SNP-heritability enrichment

We used MAGMA (v.1.08)[20] and partitioned LDSC[40] to evaluate whether the top 10% specifically expressed genes per tissue/cell type were enriched of the SNP-based h^2 of hearing loss.

MAGMA evaluated whether the cell-type-specific genes were enriched in hearing loss gene-level associations in two steps. In the first step (SNP-wise gene analysis), we filtered out SNPs with minor allele frequency <1% and poor imputation quality (INFO < 0.6) from the ARHI summary statistics and calculated the p value for per gene association with hearing loss using SNP p values (35 kb upstream and 10 kb downstream window per gene).[36] In the second step (gene-set analysis), the p values were converted to Z-scores, and one-sided tests were performed to compare whether the Z-scores in the gene set (i.e., cell-type-specific genes) were higher than those not in the gene set, which indicated enrichment of SNP-heritability in the gene set.[20,41]

We then applied partitioned LDSC adjusting for the baseline annotations.[42] Partitioned LDSC evaluates whether the per-SNP heritability is higher in the SNPs in an SNP list (in our study the SNPs within ±100 kb of the cell-type-specific genes) compared to the other SNPs.[40] We calculated p values from one-sided *Z* score coefficient for tissue/cell-type-specific genes.

Results

We curated summary statistics from 17 independent cohorts, a total of 723,266 individuals of European descent comprising 147,997 hearing loss cases (20.5%) and 575,269 controls (79.5%). Affected individuals were defined by either clinical diagnosis of hearing loss (ICD9 and 10; FinnGen and EGCUT, 37% of participants) or self-reported hearing impairment (all other cohorts, 63% of participants). We completed a genome-wide meta-analysis of 8,244,938 imputed SNPs that passed quality control (QC) and identified 48 significant loci ($p < 5 \times 10^{-8}$). There was no evidence of residual population stratification in the results of meta-analysis ($\lambda_{GC} = 1.2764$, λ_{GC} scaled to 1,000 cases and 1,000 controls = 1.001173). LD score regression intercept is 1.0039 (0.0095), indicating that the inflation of the GWAS test statistics was due to polygenicity (Figure S1). SNP heritability (h^2) on the observed scale was $h^2 = 0.0252$ (SE = 0.0013) and on the liability scale was estimated to be between 0.033 (SE = 0.002) and 0.061 (SE = 0.003) given the case/control ratio in our sample (20%) and populational prevalence in the range of 5%–40%. Next, we employed conditional and joint analysis (COJO) to identify lead independent signals in the loci (Table S3). Of 48 lead significant SNPs, 10 were considered novel associations (Table 1), defined as LD < 0.6. LDSC genetic correlations attributable to genome-wide SNPs (rg) were estimated across all hearing loss cohorts (Table S4). Regional locus zoom plots and forest plots of significant loci are presented in Figures S2 and S3, respectively.

Gene prioritization and pathway analysis

We used MAGMA v.1.08 and VEGAS2 for gene-set analysis and prioritization of genes at associated loci (Figure S4). Genes were examined for their relationship with hearing loss in human or mice. Seventeen loci were in or near genes with known associations to hearing loss (Table S5). Pathway analysis using VEGAS2 revealed strong enrichment in sensory perception of mechanical stimulus, sensory perception of sound, actin binding, and negative regulation of actin filament polymerization (Table S6). Interestingly, sensory perception pathways included *KCNQ4, OTOF, POU4F3, PDH15,* and *GRIN2B*, genes known to play a role in many different aspects of hearing function. Additional fine-mapping analysis identified credible sets of SNPs for each locus with 95% probability of being causal (total of 5,605 SNPs, Table S7).

Table 2. Missense SNPs in genes associated with hearing loss

SNP	rs9493627	rs143282422	rs35887622	rs143796236	rs12980998	rs61734651	rs5756795	rs36062310
**Gene**	***EYA4***	***CDH23***	***GJB2***	***FSCN2***	***ANKRD24***	***COL9A3***	***TRIOBP***	***KLHDC7B***
**SNP characteristics**								
Chr	6	10	13	17	19	20	22	22
Pos (hg19)	133789728	73377112	20763620	79495969	4217510	61451332	38122122	50988105
Locus	*DFNA10*	*DFNB12*	*DFNA3A DFNB1A*	–	–	–	*DFNB28*	–
Alleles (major>minor)	G>A	G>A	A>G	C>T	A>T	C>T	T>C	G>A
MAF	0.319	0.011	0.015	0.008	0.187	0.072	0.458	0.043
AA change	p.Gly277Ser	p.Ala366Thr	p.Met34Thr	p.His138Tyr	p.Thr785Ser	p.Arg103Trp	p.Phe1187Leu	p.Val1145Met
Pathogenicity score	0.29	0.43	0.71	0	1	0	1	0.71
Phenotype	hearing loss, AD	hearing loss, AR/Usher syndrome	hearing loss, AD and AR	hearing loss in mice[44]	abnormal ABR in mice[23]	Stickler syndrome, AR	hearing loss, AR	abnormal ABR in mice[23]
**Gene characteristics**								
Transcript	NM_004100.5	NM_022124.6	NM_00400.6	NM_001077182.3	NM_133475.1	NM_001853.4	NM_001039141.3	–
Gene length (bp)	5,699	10,085	2,250	1,665	4,026	2,485	10,129	2,990
Translation length	639	3,359	226	492	1,146	684	2,365	594
Number of exons	20	70	2	5	22	32	24	1
Total variants gnomAD v2.1.1	1,081	6,088	345	836	1,793	2,251	3,281	609
All $\geq$1% (total)	14	84	4	1	48	54	38	7
MAF $\geq$1% in EUR	10	60	1	6	36	53	38	7
Total unique gnomAD SNPs in same exon as GWAS SNP	0	1	0	1	6	1	14	6
**Pathogenicity**								
DM	52	353	270	0	0	9	49	0
DM?	8	67	77	0	0	1	14	1
SUM	60	420	347	0	0	10	63	1
Exon containing SNP	11	11	2	1	18	5	7	1

Abbreviations: Chr, chromosome; Pos, genomic position; MAF, minor allele frequency in the current study; AA change, amino acid change; AD, autosomal dominant; AR, autosomal recessive; bp, base pair; EUR, European (non-Finnish); MAF, minor allele frequency; SNP, single-nucleotide polymorphism; DM, disease causing mutation. Pathogenicity score is estimated from an aggregated score detailed in Table S6. Aggregated pathogenicity score is normalized from 0 (variant predicted to be deleterious) to 1 (predicted to be benign). Phenotype: in humans, except where noted otherwise. DM?, likely disease causing mutation based on the Human Gene Mutation Database (HGMD) Professional version 2021.3.[46]

Missense SNPs

Eight SNPs encoded missense mutations (Table 2). The proportion of missense SNPs overall was 17%, which is significantly higher than the average of 5.4% found in other GWAS results (GWAS Catalog accessed 19th October 2021; 1,107 studies with at least 10 genome-wide significant loci were included; Fisher exact test $p = 0.005$). Four of the identified genes have an established connection to deafness: *EYA4* (Deafness, autosomal dominant 10, DFNA10 [MIM: 601316]), *CDH23* (DFNB12 [MIM: 601543]), *GJB2* (DFNA3A [MIM 601544] and DFNB1A [MIM: 220290]), and *TRIOBP* (DFNB28 [MIM: 609823]).[43] Another three are related to hearing loss in mice: *FSCN2*,[44] *ANKRD24*, and *KLHDC7B* (https://www.

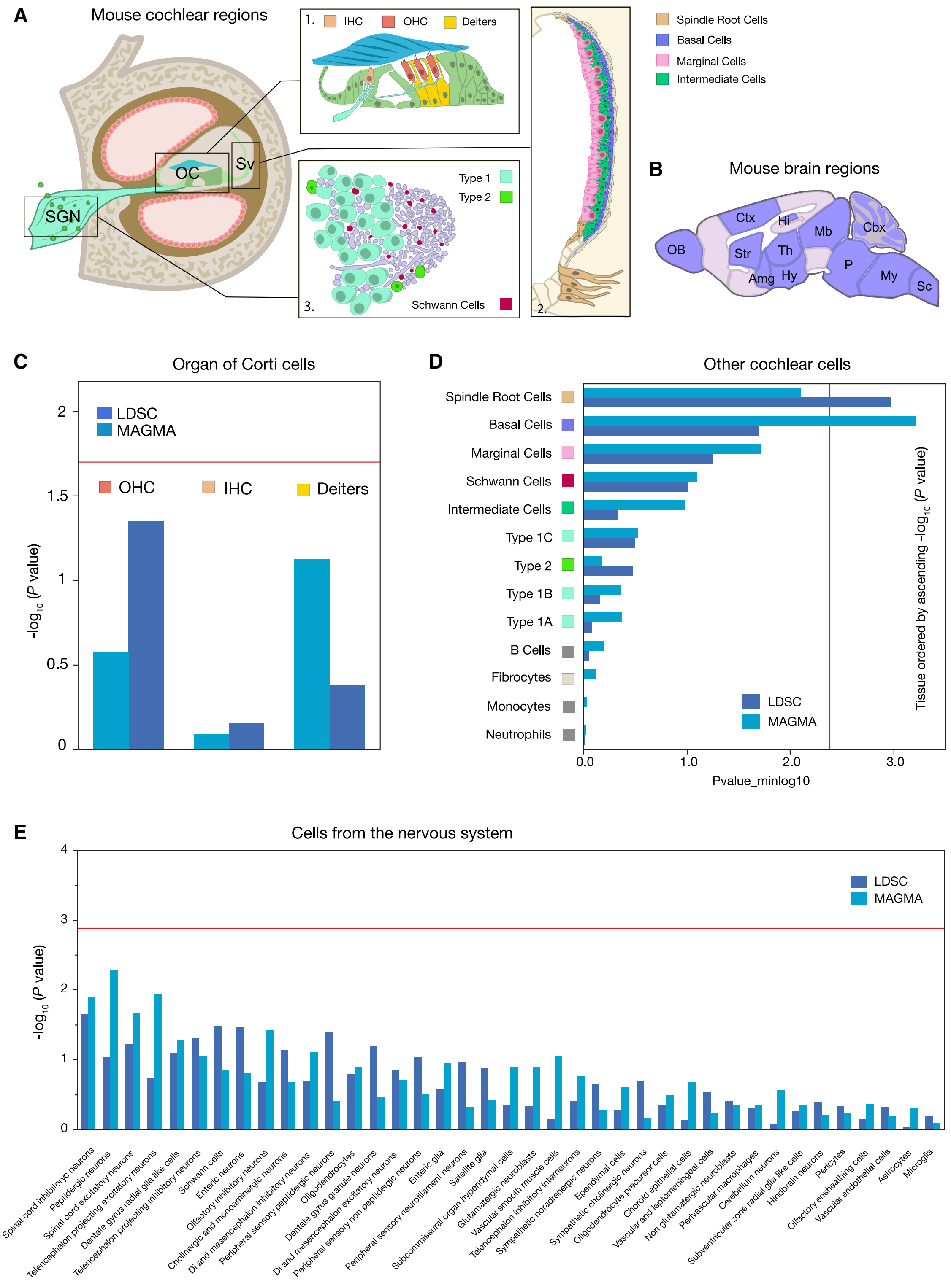

Figure 2. Evaluation of enrichment of common-variant hearing loss GWAS results in scRNA-seq mouse datasets
Schematic of the mouse cochlea (A) and the mouse brain (B) regions used for the enrichment analysis. Abbreviations: Amg, amygdala; Cbx, cerebellum; Ctx, cerebral cortex; DC, Deiters' cells; Hi, hippocampus; Hy, hypothalamus; IHC, inner hair cells; Mb, midbrain; My, medulla; OB, olfactory bulb; OC, organ of Corti; OHC, outer hair cells; P, pons; Sc, spinal cord; Str, striatum; Th, thalamus. OC is magnified in box 1 and illustrates the IHC, OHC, and DC, whose enrichment is shown in (C). A color box links a specific cell to the schematic. The red line is the Bonferroni significance threshold ($-\log_{10}$ p value 1.77). The enrichment analysis using cells from the stria vascularis (box 2) and the spiral ganglion neuron region (box 3) reveals a significant enrichment for spindle root cells and basal cells (D). All type 1 spiral ganglion neurons (type 1a, b, c) were all labeled the same color for sake of clarity. Given the broad and scarce distribution of

(legend continued on next page)

mousephenotype.org/about-impc/). Mutations in *COL9A3* cause autosomal-recessive Stickler syndrome,[45] a disorder affecting connective tissue (such as the spiral ligament) and commonly leading to hearing loss. With one exception (*ANKRD24* [CADD score = 0.241]), CADD scores of the missense SNPs (18.45 to 31) were among the top ~1%–0.1% of deleterious variants in the human genome. We conducted an additional *in silico* functional analysis on these 8 missense variants and the results provide strong evidence that variants in *FSCN2* and *COL9A3* are highly deleterious and likely impact gene function (Table S8). High-resolution protein structures were only available for *GJB2*; however, the utilized algorithms were not unanimous regarding the functional consequences of the identified variant *GJB2* p.Met34Thr (rs35887622) (Figure S5A). The GJB2 transporter, also named connexin-26 (Cx26), is a hexamer with the altered amino acid being located at the core of the channel (Figure S5A). The amino acid exchange results in a substitution of hydrophilic arginine for hydrophobic methionine, which alters the surface energy of the channel and likely affects substrate translocation (Figure S5B).

Genetic correlations

A significant positive genetic correlation was found between hearing loss and insomnia, depressive symptoms, neuroticism, and obesity (LD Hub, Figure S6, Table S9). A significant negative genetic correlation was found with subjective well-being. No significant correlation was established between hearing loss and several neurological disorders or medical conditions. Additionally, we implemented the same approach to investigate SNP-based correlation between hearing loss and other traits using recent results from the Psychiatric Genomics Consortium and found significant genetic correlation with major depressive disorder, autism spectrum disorder, alcohol dependence, neuroticism, attention deficit hyperactivity disorder, and smoking initiation/smoking (Figure S7, Table S10).

GTEx tissue enrichment analysis and cell-type specificity of hearing loss genetic associations

We next attempted to identify the specific somatic tissues implicated by our GWAS and define a spatial topography of hearing loss heritability-associated gene expression. Expression datasets for multiple human tissues GTEx v.8 do not contain inner ear tissue and, unsurprisingly, LDSC and MAGMA revealed no significant enrichment of SNP-h^2 in GTEx tissues (Figure S8; Table S11). We relied on mouse cochlear[37] and brain[39] cell-specific expression profiles to determine cell types matching the common variants identified. This approach enabled us to prioritize cells that are fundamental to the etiology of hearing loss. Mouse cochlea scRNA-seq originated from Milon et al.,[37] which included the spiral ganglion region and the stria vascularis (total of 36,616 cells; Figure 2A, Table S2), but since this dataset did not include scRNA-seq data from cells of the organ of Corti (that harbor hair cells and Deiters' cells), we included an additional dataset from Ranum et al. containing Deiters' cells and inner and outer hair cells from the mouse cochleae[38] (total of 3,189 cells; Figure 2A; Table S2). Since these two cochlea studies used different methods, these were analyzed separately. Nervous system scRNA-seq included 39 broad cell types from 19 regions of the mouse central, peripheral, and enteric nervous system (total of 160,796 cells; Figure 2B).[39]

If hearing loss is associated with a particular cell type, we would expect more of the genome-wide association signal to be concentrated in genes with greater specificity for that cell type. To show evidence connecting hearing loss GWASs to cell type, we used two different methods accounting for gene size and linkage disequilibrium: LDSC,[40] assessing the enrichment of the common SNP heritability of hearing loss in the most cell-type-specific genes and MAGMA,[20] evaluating whether gene-level genetic association with hearing loss linearly increases with cell-type expression specificity. We found no enrichment in cells from the organ of Corti (Deiters' cells, inner and outer hair cells; Figure 2C, Table S12). Arguably, the lack of results in this enrichment analysis could be due to the fact that specificity is a relative measure between these three cell types. This assumes that the "effective" genes have similar expression pattern in all three cell types and therefore would not be captured by the specificity measure. However, this reasoning is not supported by current findings since Deiters' cells and hair cells differ considerably in gene expression signature.[38] When assessing the enrichment in SGN and cells from the cochlear lateral wall (stria vascularis), LDSC analysis revealed the involvement of spindle cells of the stria vascularis and root cells of the outer sulcus, whereas MAGMA analysis highlighted the involvement of basal cells of the stria vascularis in hearing loss (Figure 2D, Table S13). Here, spindle and root cells could not be distinguished molecularly one from another, which is why they were labeled "spindle root cells." In contrast, no enrichment was found in any cell type from the mouse nervous system (Figure 2E, Table S14). These findings strongly support a prominent role of the stria vascularis in hearing loss.

To further gain insights into the potential molecular mechanisms involved in strial dysfunction, we investigated the top 10% specifically expressed genes in basal (342 genes, Table S15) and spindle root (380 genes, Table S16) cells. In basal cells, 10 genes were associated with SNPs that were GWAS significant, but none of them

immune cells (monocytes, neutrophils, and B cells), these are not shown on the schematic. The red line shows the Bonferroni significance threshold ($-\log_{10}$ p value 2.42) (E). Mouse nervous system cell type enrichment showing no significant enrichment. The red line shows the Bonferroni significance threshold ($-\log_{10}$ p value 2.89). Images from (A) and (B) were reproduced from previous work[47–49] with permission from Nature Springer.

were found in the significant pathways associated with hearing loss and listed in Table S6. Among these genes, the evidence for an involvement in hearing loss is sparse. For instance, *NID2*, which has been associated in humans with the Landau-Kleffner syndrome, a rare language disorder with suspicions of hearing loss.[50] *PC* encodes a pyruvate carboxylase that requires biotin and ATP for catalyzing gluconeogenesis. Pyruvate carboxylase deficiency is a rare severe metabolic disease that in some cases can be manifested with hearing loss.[51] *CCS* encodes a copper ion binding protein and its mutations are associated with disfunctions of copper metabolism resulting in Wilson disease, a rare inherited disorder that causes excess accumulation of copper in several organs.[52] Individuals with Wilson disease display abnormal auditory brainstem responses.[53] Similarly, *AHDC1* is most probably involved in DNA binding, and loss-of-function mutations result in Xia-Gibbs syndrome—a neurodevelopmental disorder with rare presentation of hearing loss.[54] In spindle root cells, *EYA4* and *HOMER2* were identified in pathways that were found significant in VEGAS2—sensory perception of sound and sensory perception of mechanical stimulus, respectively. The contribution of *EYA4* variants to hearing loss has been well established.[55] HOMER2 is involved in intracellular homeostasis of calcium and cytoskeletal organization and has been previously associated with hearing loss,[56] but its function within the stria vascularis remains unknown. *TMPRSS9* encodes a membrane-bound serine polyprotease involved in the proliferation of inner ear progenitor cells in the mouse cochlea.[57] *GAS2* encodes an actin filament binding protein that plays a role in cell shape and regulating microfilament rearrangements. In mice, *Gas2* is expressed in supporting cells but also in the stria vascularis from the post-natal cochlea and its disruption causes hearing loss in mice and human.[58] Taken together, these findings suggest that dysfunctions in the stria vascularis involve a large range of molecular mechanisms, globally impacting strial function.

Discussion

The present genome-wide meta-analysis is among the largest conducted in hearing genetics to date and provides an association catalog that helps to refine the fundamental basis of hearing loss. We find evidence of association for 48 common genetic loci, of which 10 were novel and highlight the role of genes expressed in cochlear lateral wall, consisting of the spiral ligament and stria vascularis as an important contributor to hearing loss. We employed a pragmatic, clinically informed approach by including cohorts that met empirical criteria for sufficient genetic and phenotypic similarity, based on both self-report and medical registries. We previously verified a high genetic correlation between objective measures of hearing loss and questionnaires.[14] Second, our findings point to multiple genes that have been reported to cause Mendelian forms of hearing loss previously, including *EYA4, CDH23, TRIOBP*, and *GJB2*, the latter being the most commonly reported gene in autosomal-recessive non-syndromic hearing loss.[59] This study is part of an expanding number of hearing loss GWASs with ever increasing sample size and the emerging functional and cellular bioinformatics tools enable us to unravel more of its pathophysiological pathways. Our study confirms as do other recent GWASs that hearing loss is driven by multiple common variants in known hearing genes.

Our results allow us to draw several broad conclusions. Of importance, a large proportion of potentially disruptive missense variants were found in contrast to other disease-related GWASs (Fischer's exact test; $p = 0.005$; Figure S9), suggesting that a burden of common and rare yet impactful variants may drive the risk of hearing loss.

Second, our results do not point toward a large involvement of the brain within hearing loss. Although this is not entirely unexpected, proficient hearing acuity requires the functional integration of signals that are captured at the level of the cochlea, which are transduced to provide signal down the VIIIth cranial nerve and further propagate via the brainstem toward the thalamus and the auditory cortex. Abnormal CNS streaming of signal to noise has been implicated in ARHI[60] and is supported by the association with cognitive decline, but our GTEx and scRNA-seq analyses revealed no enrichment of GWA signals in the brain, nor in its regions or its major cell types.

Third, we found significant positive genetic correlations with depressive symptoms, obesity, and smoking, but not with Alzheimer disease. The latter is interesting as hearing impairment is an established risk factor for cognitive decline and dementia,[61] suggesting that hearing impairment rather than shared underlying genetic factors contribute to the development of dementia. A more complete analysis of the drivers of this relationship is needed, but the inference is that shared environmental factors contributing to hearing loss and dementia will predominate, rather than shared genetic factors.

Fourth, our findings in hearing loss pathway analyses implicate the processes involved in cytoskeleton organization and actin binding, two broad features of the mechano-transduction apparatus of the sensory hair cells.[62] Indeed, these findings are consistent with a recent GWAS performed on the UKBB that localized a number of lead SNPs in cells from the post-natal mouse cochleae using scRNA-seq data[12] or in the human cochlea (mainly in type I SGN, or hair cells) using immunohistochemistry on samples collected from individuals with life-threatening posterior cranial fossa meningioma compressing the brain stem.[63] These included *EYA4, LMX1A, PTK2/FAK, UBE3B, MMP2, SYNJ2, GRM5, TRIOBP, LMO-7,* and *NOX4*. Consistent with their findings, we also identified *SPTBN1*, a mouse ortholog Spectrin expressed in the cuticular plate at the base of the stereocilia, deletion of which causes profound deafness.[64] Interestingly, synaptic plasticity genes were also found such *CTBP2*, which is an

important marker of the pre-synaptic machinery—namely the synaptic ribbon—gathering the glutamate-filled vesicles prior to their release. Alterations in ribbon abundance has been associated with cochlear synaptopathy in mouse models of noise-induced hearing loss.[65–68] In these models a decrease in ribbon abundance in the absence of hearing loss (the so-called hidden hearing loss) is thought to be associated with problems in speech in noise recognition and tinnitus.[69,70]

Fifth, although lead association SNPs are related to sensory hair cell function and their involvement in hearing loss is well established, our cell-specific enrichment analysis revealed hearing loss being also driven, at least in part, by basal cells and spindle cells in the stria vascularis and root cells in the outer sulcus. Indeed, the lead SNPs mainly related to hair cell and auditory neuron function represent the tip of the iceberg (e.g., *STBPN1, CLRN2, EYA4, SYNJ2, CDH23, CTBP2, LMO7, FSCN2, ANKRD24, TRIOBP,* and *BAIAP2L2*) influencing hearing loss with the greatest probability, while the whole iceberg is pictured by the contribution of all GWAS signals, pointing to genes expressed in cells from the lateral wall, namely basal cells and spindle cells of the stria vascularis and root cells in the outer sulcus (e.g., *EYA4, MMP2, GJB2*, and *GJB6*). These three cell types are primarily involved in endolymph ion homeostasis.[47,71–73] The basal cells are coupled to each other by tight junctions to prevent leakage of ions[74] and to keep the stria vascularis separate from the spiral ligament. In addition, the spindle cells have recently been shown through gene regulatory networks to have a role in responses to inflammation.[47] However, we were unable to differentiate the spindle cells from the root cells unlike Gu et al.[47] who used single-nucleus RNA-seq and could identify a differential expression between these two cell types.

Our findings are in opposition to a recent study suggesting that outer and inner hair cell loss is the main contributor to ARHI and that strial tissue loss does not correlate with audiologic patterns of ARHI.[75] The human otopathologic analysis of this study focused on cellular loss of the stria vascularis and may have captured loss of basal cells that cover the full extension of stria. However, cellular loss of the other two cell types is not covered by this analysis. The spindle cells reside at the edges of the stria and the root cells are outside of the stria in the outer sulcus region. Furthermore, genetically caused functional loss cannot be recognized at the histopathologic level and may precede cellular loss. The precise contribution of the sensory cell and stria vascularis mechanisms to the development of hearing loss needs to be further elucidated using molecular techniques and at a time before cell death is apparent.

The lack of cochlear tissue in the GTEx biobank is indeed a major limitation to all genetic studies of hearing and communication disorders that needs to be addressed. Having access to eQTL data from human cochlear tissue would also provide significant advances in understanding the biology of hearing loss. These limitations were partially addressed in our study by gathering a unique combination of datasets of mouse cochlear scRNA-seq. Given the complexity of the organ and its ossification, the number of compartments, and the variety of constituent cell types, such comprehensive knowledge was not available until recently. Using expression data from rodents to infer on human auditory physiology may be seen as a limitation, since mouse expression data are not fully representative of human cells. However, from a total of 48 loci we identified, 18 harbor genes that have been associated with hearing loss. From these 18 loci, 16 are related to genes disruption of which causes hearing loss in mice. Thus, these findings strongly argue in favor of the translational reliability of the present findings.

We also note that part of the scRNA-seq mouse data used here was generated from 10X Genomics, which has a sequencing resolution that may have yielded insufficient number of genes to reveal enrichments (e.g., when compared to new methodologies such as Smart-Seq2). For instance, using 1,100 proprioceptive neurons and Smart-seq2, Wu et al. detected 11,000 genes per cell and identified 8 cell types,[76] while previous studies using 10X Genomics favoring a higher number of cells but with lower coverage could not differentiate proprioceptive neurons in any subtypes.[77] In the study from Milon et al., only one type of fibrocyte was identified, whereas there are 5 known types of fibrocytes (type I–V) present in both man and experimental animals, but that have quite different function and molecular expression.[78,79] Thus, new sequencing technologies may offer increased resolution and statistical power to reveal more accurate predictions of the involvement of more specific cells in hearing loss.

Hearing loss is a heterogeneous disorder with many contributing factors during life. A potential implication for future genetic studies is the elucidation of the bulk of common variants using a cost-effective shortcut involving on-line self-reports combined with auditory tests, such as automated speech and noise test.[80] The use of online assessment would allow for a comprehensive recording of phenotypes and environmental exposures in millions of individuals. Conversely, current clinical audiologic phenotyping is mostly limited to pure tone thresholds and by far not exhaustive. Carefully phenotyped individuals with hearing loss in combination with next-generation sequencing may increase the resolution of the genetic coverage and reduce the sample size to thousands, something that has been shown highly effective in the context of schizophrenia.[81]

Conclusions

This study of hearing loss identified 48 associated loci, including 10 novel associations and 8 missense SNPs. Our work highlights the role of the cochlear lateral wall including the stria vascularis and the outer sulcus as a contributor to hearing loss. The results provide a valuable resource for the selection of promising genes for further functional validation in pre-clinical models and define

targets for screening purposes, drug development, gene therapy, or stratification approaches. We believe such experiments will serve as a solid foundation for ultimately improving therapies against hearing loss.

Data and code availability

The GWAS summary statistics are deposited and available in Zenodo (https://zenodo.org/record/5769707#.Ybm6v33MKhx) and codes are available on GitHub (https://github.com/translational-audiology-lab/GWAS_ARHL).

Supplemental information

Supplemental information can be found online at https://doi.org/10.1016/j.ajhg.2022.04.010.

Consortia

The Estonian Biobank Research Team is composed of Andres Metspalu, Mari Nelis, Reedik Mägi, and Tõnu Esko.

Acknowledgments

This study was supported by the GENDER-Net Co-Plus Fund (GNP-182), the European Union's Horizon 2020 Research and Innovation Programme, Grant Agreement No 848261 and No 722046 to C.R.C. C.R.C. received additional funding from Forschung Für Leben, Svenska Läkaresällskapet (SLS-779681), Hörselforskningsfonden (503), and Tysta Skolan. J.H.L. received funding from Swedish Brain Foundation (Hjärnfonden) and Swedish Research Council. F.L. received funding from Knut and Alice Wallenberg Foundation, Tysta Skolan, Swedish Research Council, StratRegen, and Swedish Brain Foundation. R.H. received funding from NIDCD/NIH R01DC013817, NIDCD/NIH R01DC019370, the Hearing Restoration Project, and CDMRP/DOD W81XWH-21-1-0578. S.S. received funding from the National Heart, Lung, and Blood Institute contract for the Framingham Heart Study (contract No. N01-HC-25195, No. HHSN268201500001I, and No. 75N92019D00031), the National Institute on Aging (R01 AG054076, R01 AG049607, U01 AG052409, R01 AG059421, RF1 AG063507, RF1 AG066524, U01 AG058589), and the National Institute of Neurological Disorders and Stroke (R01 NS017950 and UH2 NS100605).

Additional funding was obtained from the German Research Foundation through the Collaborative Research Center 889 (BV), Academy of Finland (grant #312073, J.K.); R01 AG059727, P30 AG066546, and UF1 NS125513 (S.L.S.B.); and Beneficentia Stiftung and D70-RESRICGIROTTO (G.G.). TwinsUK is funded by the Wellcome Trust, Medical Research Council, European Union, Chronic Disease Research Foundation (CDRF), Zoe Global Ltd and the National Institute for Health Research (NIHR)-funded BioResource, Clinical Research Facility and Biomedical Research Center based at Guy's and St Thomas' NHS Foundation Trust in partnership with King's College London. Hearing research within The Rotterdam Study has been founded by the Dutch Hearing Health Foundation.

Author contributions

Cohort-specific data collection, I.M.L., L.M., P.K.E.M., E.S., T.P., S.P., C.C.M., A.B.S.G., H.L., A.T., C.L.S., D.I.C.; analysis, N.T., M.B.F., L.B., S.Y., E.B.R.T., K.K., Y.Z., M.N., D.I.C., F.M.K.W., A.G., J.K., E.S., R.E., M.P.C., S.C., G.G.N., H.L., N.L.H.C., F.C.; critical revisions of the manuscript, V.G., L.J.L., J.H.L., R.H., T.P., E.S., J.R., A.M., J.K., A.B.V., H.L., A.T., A.G.; writing editing, Y.G., H.J.H., L.B., M.B.F., A.P.N., C.R.C., B.C.O., N.T., C.B., A.K.K., B.C., S.Y., E.S., J.R., A.M., J.K., A.B.V., C.C.M., A.B.S.G., D.I.C., F.M.K.W.; overseeing project, L.B., A.P.N., C.R.C., B.C.O., P.F.S., S.S., L.M., P.K.E.M., J.v.B.H., J.M.F., E.S., T.P., E.S., M.A.N.; study concept and design, Y.G., H.J.H., C.M.L., V.G., A.G., J.M.S., J.v.M., A.C., K.C., V.M.L., C.S., L.J.L., F.L., J.H.L., R.H., L.B., M.B.F., R.S., G.G., N.Q., A.P.N., C.R.C., B.C.O., N.T., P.F.S., S.S., E.S., J.R., A.M., J.K., A.B.V.

Declaration of interests

The funders had no role in study design, data collection and analysis, decision to publish, or preparation of the manuscript. C.R.C. is supported by the UK National Institute for Health Research (NIHR) Biomedical Research Center but the views expressed herein are his own and do not represent those of NIHR nor the UK Department of Health and Social Care. Y.Z. and V.M.L. are co-founders and shareholders of PersoMedix AB. In addition, V.M.L. is CEO and shareholder of HepaPredict AB and discloses support by the Robert Bosch Foundation, Merck KGaA, and Eli Lilly and Company. J.H.L. is co-founder and share-holder of Oscellaria AB. H.L. receives support from a consulting contract between Data Tecnica International and the National Institute on Aging (NIA), National Institutes of Health (NIH). M.A.N. received a competitive contract awarded to Data Tecnica International LLC by the National Institutes of Health to support open science research, and he also currently serves on the scientific advisory board for Clover Therapeutics and is an advisor to Neuron23 Inc as a data science fellow. P.F.S. is consultant and shareholder of Neumora.

Received: January 4, 2022
Accepted: April 15, 2022
Published: May 16, 2022

References

1. GBD 2019 Hearing Loss Collaborators (2021). Hearing loss prevalence and years lived with disability, 1990-2019: findings from the Global Burden of Disease Study 2019. Lancet *397*, 996–1009.
2. McDaid, D., Park, A.L., and Chadha, S. (2021). Estimating the global costs of hearing loss. Int. J. Audiol. *60*, 162–170. https://doi.org/10.1080/14992027.2021.1883197.
3. Li, C.M., Zhang, X., Hoffman, H.J., Cotch, M.F., Themann, C.L., and Wilson, M.R. (2014). Hearing impairment associated with depression in US adults, national Health and nutrition examination survey 2005-2010. JAMA Otolaryngol. Head Neck Surg. *140*, 293–302. https://doi.org/10.1001/jamaoto.2014.42.
4. Cederroth, C.R., Canlon, B., and Langguth, B. (2013). Hearing loss and tinnitus–are funders and industry listening? Nat. Biotechnol. *31*, 972–974. https://doi.org/10.1038/nbt.2736.
5. Bowl, M.R., and Dawson, S.J. (2014). The mouse as a model for age-related hearing loss - a mini-review. Gerontology *61*, 149–157. https://doi.org/10.1159/000368399.
6. Vona, B., Doll, J., Hofrichter, M.A.H., Haaf, T., and Varshney, G.K. (2020). Small fish, big prospects: using zebrafish to unravel

the mechanisms of hereditary hearing loss. Hearing Res. *397*, 107906. https://doi.org/10.1016/j.heares.2020.107906.

7. Kvestad, E., Czajkowski, N., Krog, N.H., Engdahl, B., and Tambs, K. (2012). Heritability of hearing loss. Epidemiology *23*, 328–331. https://doi.org/10.1097/ede.0b013e318245996e.
8. Wolber, L.E., Steves, C.J., Spector, T.D., and Williams, F.M.K. (2012). Hearing ability with age in northern European women: a new web-based approach to genetic studies. PLoS One *7*, e35500. https://doi.org/10.1371/journal.pone.0035500.
9. Wells, H.R.R., Freidin, M.B., Zainul Abidin, F.N., Payton, A., Dawes, P., Munro, K.J., Morton, C.C., Moore, D.R., Dawson, S.J., and Williams, F.M.K. (2019). GWAS identifies 44 independent associated genomic loci for self-reported adult hearing difficulty in UK biobank. Am. J. Hum. Genet. *105*, 788–802. https://doi.org/10.1016/j.ajhg.2019.09.008.
10. Ivarsdottir, E.V., Holm, H., Benonisdottir, S., Olafsdottir, T., Sveinbjornsson, G., Thorleifsson, G., Eggertsson, H.P., Halldorsson, G.H., Hjorleifsson, K.E., Melsted, P., et al. (2021). The genetic architecture of age-related hearing impairment revealed by genome-wide association analysis. Commun. Biol. *4*, 706. https://doi.org/10.1038/s42003-021-02224-9.
11. Turley, P., Walters, R.K., Maghzian, O., Okbay, A., Lee, J.J., Fontana, M.A., Nguyen-Viet, T.A., Wedow, R., Zacher, M., Furlotte, N.A., et al.; Social Science Genetic Association Consortium (2018). Multi-trait analysis of genome-wide association summary statistics using MTAG. Nat. Genet. *50*, 229–237. https://doi.org/10.1038/s41588-017-0009-4.
12. Kalra, G., Milon, B., Casella, A.M., Herb, B.R., Humphries, E., Song, Y., Rose, K.P., Hertzano, R., and Ament, S.A. (2020). Biological insights from multi-omic analysis of 31 genomic risk loci for adult hearing difficulty. PLoS Genet. *16*, e1009025. https://doi.org/10.1371/journal.pgen.1009025.
13. Vuckovic, D., Mezzavilla, M., Cocca, M., Morgan, A., Brumat, M., Catamo, E., Concas, M.P., Biino, G., Franze, A., Ambrosetti, U., et al. (2018). Whole-genome sequencing reveals new insights into age-related hearing loss: cumulative effects, pleiotropy and the role of selection. Eur. J. Hum. Genet. *26*, 1167–1179. https://doi.org/10.1038/s41431-018-0126-2.
14. Cherny, S.S., Livshits, G., Wells, H.R.R., Freidin, M.B., Malkin, I., Dawson, S.J., and Williams, F.M.K. (2020). Self-reported hearing loss questions provide a good measure for genetic studies: a polygenic risk score analysis from UK Biobank. Eur. J. Hum. Genet. *28*, 1056–1065. https://doi.org/10.1038/s41431-020-0603-2.
15. Winkler, T.W., Day, F.R., Croteau-Chonka, D.C., Wood, A.R., Locke, A.E., Magi, R., Ferreira, T., Fall, T., Graff, M., Justice, A.E., et al. (2014). Quality control and conduct of genome-wide association meta-analyses. Nat. Protoc. *9*, 1192–1212. https://doi.org/10.1038/nprot.2014.071.
16. Willer, C.J., Li, Y., and Abecasis, G.R. (2010). METAL: fast and efficient meta-analysis of genomewide association scans. Bioinformatics *26*, 2190–2191. https://doi.org/10.1093/bioinformatics/btq340.
17. Bulik-Sullivan, B.K., Loh, P.R., Finucane, H.K., Ripke, S., Yang, J., Schizophrenia Working Group of the Psychiatric Genomics Consortium, Patterson, N., Daly, M.J., Price, A.L., and Neale, B.M. (2015). LD Score regression distinguishes confounding from polygenicity in genome-wide association studies. Nat. Genet. *47*, 291–295. https://doi.org/10.1038/ng.3211.
18. Yang, J., Lee, S.H., Goddard, M.E., and Visscher, P.M. (2011). GCTA: a tool for genome-wide complex trait analysis. Am. J. Hum. Genet. *88*, 76–82. https://doi.org/10.1016/j.ajhg.2010.11.011.
19. Kircher, M., Witten, D.M., Jain, P., O'Roak, B.J., Cooper, G.M., and Shendure, J. (2014). A general framework for estimating the relative pathogenicity of human genetic variants. Nat. Genet. *46*, 310–315. https://doi.org/10.1038/ng.2892.
20. de Leeuw, C.A., Mooij, J.M., Heskes, T., and Posthuma, D. (2015). MAGMA: generalized gene-set analysis of GWAS data. PLoS Comput. Biol *11*, e1004219. https://doi.org/10.1371/journal.pcbi.1004219.
21. Watanabe, K., Taskesen, E., van Bochoven, A., and Posthuma, D. (2017). Functional mapping and annotation of genetic associations with FUMA. Nat. Commun. *8*, 1826. https://doi.org/10.1038/s41467-017-01261-5.
22. Mishra, A., and Macgregor, S. (2015). VEGAS2: software for more flexible gene-based testing. Twin Res. Human Genet. *18*, 86–91. https://doi.org/10.1017/thg.2014.79.
23. Dickinson, M.E., Flenniken, A.M., Ji, X., Teboul, L., Wong, M.D., White, J.K., Meehan, T.F., Weninger, W.J., Westerberg, H., Adissu, H., et al. (2016). High-throughput discovery of novel developmental phenotypes. Nature *537*, 508–514. https://doi.org/10.1038/nature19356.
24. Shen, J., Scheffer, D.I., Kwan, K.Y., and Corey, D.P. (2015). SHIELD: an integrative gene expression database for inner ear research. Database (Oxford) *2015*, bav071. https://doi.org/10.1093/database/bav071.
25. Liu, H., Pecka, J.L., Zhang, Q., Soukup, G.A., Beisel, K.W., and He, D.Z.Z. (2014). Characterization of transcriptomes of cochlear inner and outer hair cells. J. Neurosci. *34*, 11085–11095. https://doi.org/10.1523/jneurosci.1690-14.2014.
26. Mishra, A., and MacGregor, S. (2017). A novel approach for pathway analysis of GWAS data highlights role of BMP signaling and muscle cell differentiation in colorectal cancer susceptibility. Twin Res. Hum. Genet. *20*, 1–9. https://doi.org/10.1017/thg.2016.100.
27. Azaiez, H., Booth, K.T., Ephraim, S.S., Crone, B., Black-Ziegelbein, E.A., Marini, R.J., Shearer, A.E., Sloan-Heggen, C.M., Kolbe, D., Casavant, T., et al. (2018). Genomic landscape and mutational signatures of deafness-associated genes. Am. journal Hum. Genet. *103*, 484–497. https://doi.org/10.1016/j.ajhg.2018.08.006.
28. Adzhubei, I.A., Schmidt, S., Peshkin, L., Ramensky, V.E., Gerasimova, A., Bork, P., Kondrashov, A.S., and Sunyaev, S.R. (2010). A method and server for predicting damaging missense mutations. Nat. Methods *7*, 248–249. https://doi.org/10.1038/nmeth0410-248.
29. Quang, D., Chen, Y., and Xie, X. (2015). DANN: a deep learning approach for annotating the pathogenicity of genetic variants. Bioinformatics *31*, 761–763. https://doi.org/10.1093/bioinformatics/btu703.
30. Choi, Y., Sims, G.E., Murphy, S., Miller, J.R., and Chan, A.P. (2012). Predicting the functional effect of amino acid substitutions and indels. PLoS One *7*, e46688. https://doi.org/10.1371/journal.pone.0046688.
31. Ioannidis, N.M., Rothstein, J.H., Pejaver, V., Middha, S., McDonnell, S.K., Baheti, S., Musolf, A., Li, Q., Holzinger, E., Karyadi, D., et al. (2016). REVEL: an ensemble method for predicting the pathogenicity of rare missense variants. Am. J. Hum. Genet. *99*, 877–885. https://doi.org/10.1016/j.ajhg.2016.08.016.
32. Carter, H., Douville, C., Stenson, P.D., Cooper, D.N., and Karchin, R. (2013). Identifying Mendelian disease genes with

the variant effect scoring tool. BMC Genomics *14*, S3. https://doi.org/10.1186/1471-2164-14-s3-s3.
33. Ionita-Laza, I., McCallum, K., Xu, B., and Buxbaum, J.D. (2016). A spectral approach integrating functional genomic annotations for coding and noncoding variants. Nat. Genet. *48*, 214–220. https://doi.org/10.1038/ng.3477.
34. Zheng, J., Erzurumluoglu, A.M., Elsworth, B.L., Kemp, J.P., Howe, L., Haycock, P.C., Hemani, G., Tansey, K., Laurin, C., Pourcain, B.S., et al.; Early Genetics and Lifecourse Epidemiology EAGLE Eczema Consortium (2017). LD Hub: a centralized database and web interface to perform LD score regression that maximizes the potential of summary level GWAS data for SNP heritability and genetic correlation analysis. Bioinformatics *33*, 272–279. https://doi.org/10.1093/bioinformatics/btw613.
35. GTEx Consortium; Laboratory, Data Analysis &Coordinating Center (LDACC)—Analysis Working Group, Statistical Methods groups-Analysis Working Group, Enhancing GTEx (eGTEx) groups; NIH Common Fund; NIH/NCI; NIH/NHGRI; NIH/NIMH; NIH/NIDA; Biospecimen Collection Source Site—NDRI; et al. (2017). Genetic effects on gene expression across human tissues. Nature 550, 204-213, https://doi.org/10.1038/nature24277.
36. Bryois, J., Skene, N.G., Hansen, T.F., Kogelman, L.J.A., Watson, H.J., Liu, Z., Eating Disorders Working Group of the Psychiatric Genomics Consortium, International Headache Genetics Consortium, International Headache Genetics Consortium, 23andMe Research Team, et al. (2020). Genetic identification of cell types underlying brain complex traits yields insights into the etiology of Parkinson's disease. Nat. Genet. *52*, 482–493. https://doi.org/10.1038/s41588-020-0610-9.
37. Milon, B., Shulman, E.D., So, K.S., Cederroth, C.R., Lipford, E.L., Sperber, M., Sellon, J.B., Sarlus, H., Pregernig, G., Shuster, B., et al. (2021). A cell-type-specific atlas of the inner ear transcriptional response to acoustic trauma. Cell Rep. *36*, 109758. https://doi.org/10.1016/j.celrep.2021.109758.
38. Ranum, P.T., Goodwin, A.T., Yoshimura, H., Kolbe, D.L., Walls, W.D., Koh, J.Y., He, D.Z.Z., and Smith, R.J.H. (2019). Insights into the biology of hearing and deafness revealed by single-cell RNA sequencing. Cell Rep. *26*, 3160–3171.e3. https://doi.org/10.1016/j.celrep.2019.02.053.
39. Zeisel, A., Hochgerner, H., Lonnerberg, P., Johnsson, A., Memic, F., van der Zwan, J., Haring, M., Braun, E., Borm, L.E., La Manno, G., et al. (2018). Molecular architecture of the mouse nervous system. Cell *174*, 999–1014.e22. https://doi.org/10.1016/j.cell.2018.06.021.
40. Finucane, H.K., Bulik-Sullivan, B., Gusev, A., Trynka, G., Reshef, Y., Loh, P.R., Anttila, V., Xu, H., Zang, C., Farh, K., et al.; Schizophrenia Working Group of the Psychiatric Genomics Consortium; and The RACI Consortium (2015). Partitioning heritability by functional annotation using genome-wide association summary statistics. Nat. Genet. *47*, 1228–1235. https://doi.org/10.1038/ng.3404.
41. Brown, M.B. (1975). 400: a method for combining non-independent, one-sided tests of significance. Biometrics *31*, 987–992. https://doi.org/10.2307/2529826.
42. Gazal, S., Loh, P.R., Finucane, H.K., Ganna, A., Schoech, A., Sunyaev, S., and Price, A.L. (2018). Functional architecture of low-frequency variants highlights strength of negative selection across coding and non-coding annotations. Nat. Genet. *50*, 1600–1607. https://doi.org/10.1038/s41588-018-0231-8.
43. Van Camp, G., and Smith, R.J.H. Hereditary Hearing Loss Homepage: hereditaryhearingloss.Org.
44. Shin, J.B., Longo-Guess, C.M., Gagnon, L.H., Saylor, K.W., Dumont, R.A., Spinelli, K.J., Pagana, J.M., Wilmarth, P.A., David, L.L., Gillespie, P.G., et al. (2010). The R109H variant of fascin-2, a developmentally regulated actin crosslinker in hair-cell stereocilia, underlies early-onset hearing loss of DBA/2J mice. J. Neurosci. *30*, 9683–9694. https://doi.org/10.1523/jneurosci.1541-10.2010.
45. Faletra, F., D'Adamo, A.P., Bruno, I., Athanasakis, E., Biskup, S., Esposito, L., and Gasparini, P. (2014). Autosomal recessive Stickler syndrome due to a loss of function mutation in the COL9A3 gene. Am. journal Med. Genet. *164*, 42–47. https://doi.org/10.1002/ajmg.a.36165.
46. Stenson, P.D., Mort, M., Ball, E.V., Shaw, K., Phillips, A.D., and Cooper, D.N. (2014). The Human Gene Mutation Database: building a comprehensive mutation repository for clinical and molecular genetics, diagnostic testing and personalized genomic medicine. Hum. Genet. *133*, 1–9. https://doi.org/10.1007/s00439-013-1358-4.
47. Gu, S., Olszewski, R., Taukulis, I., Wei, Z., Martin, D., Morell, R.J., and Hoa, M. (2020). Characterization of rare spindle and root cell transcriptional profiles in the stria vascularis of the adult mouse cochlea. Sci. Rep. *10*, 18100. https://doi.org/10.1038/s41598-020-75238-8.
48. Skene, N.G., Bryois, J., Bakken, T.E., Breen, G., Crowley, J.J., Gaspar, H.A., Giusti-Rodriguez, P., Hodge, R.D., Miller, J.A., Munoz-Manchado, A.B., et al. (2018). Genetic identification of brain cell types underlying schizophrenia. Nat. Genet. *50*, 825–833. https://doi.org/10.1038/s41588-018-0129-5.
49. Lang, H. (2016). Loss, degeneration, and preservation of the spiral ganglion neurons and their processes. In The Primary Auditory Neurons of the Mammalian Cochlea, A. Dabdoub, B. Fritzsch, A. Popper, and R. Fay, eds. (Springer Handbook of Auditory Research).
50. Appleton, R.E. (1995). The Landau-Kleffner syndrome. Arch. Dis. Child. *72*, 386–387. https://doi.org/10.1136/adc.72.5.386.
51. Wang, D., and De Vivo, D. (1993). Pyruvate carboxylase deficiency. In GeneReviews((R)), M.P. Adam, H.H. Ardinger, R.A. Pagon, S.E. Wallace, L.J.H. Bean, K.W. Gripp, G.M. Mirzaa, and A. Amemiya, eds.. (Seattle (WA).
52. Czlonkowska, A., Litwin, T., Dusek, P., Ferenci, P., Lutsenko, S., Medici, V., Rybakowski, J.K., Weiss, K.H., and Schilsky, M.L. (2018). Wilson disease. Nat. Rev. Dis. primers *4*, 21. https://doi.org/10.1038/s41572-018-0018-3.
53. Butinar, D., Trontelj, J.V., Khuraibet, A.J., Khan, R.A., Hussein, J.M., and Shakir, R.A. (1990). Brainstem auditory evoked potentials in Wilson's disease. J. Neurol. Sci. *95*, 163–169. https://doi.org/10.1016/0022-510x(90)90239-j.
54. Chander, V., Wangler, M., Gibbs, R., and Murdock, D. (1993). Xia-gibbs syndrome. In GeneReviews((R)), M.P. Adam, H.H. Ardinger, R.A. Pagon, S.E. Wallace, L.J.H. Bean, K.W. Gripp, G.M. Mirzaa, and A. Amemiya, eds.. (Seattle (WA).
55. Wells, H.R.R., Newman, T.A., and Williams, F.M.K. (2020). Genetics of age-related hearing loss. J. Neurosci. Res. *98*, 1698–1704. https://doi.org/10.1002/jnr.24549.
56. Azaiez, H., Decker, A.R., Booth, K.T., Simpson, A.C., Shearer, A.E., Huygen, P.L.M., Bu, F., Hildebrand, M.S., Ranum, P.T., Shibata, S.B., et al. (2015). HOMER2, a stereociliary scaffolding protein, is essential for normal hearing in humans and mice. PLoS Genet. *11*, e1005137. https://doi.org/10.1371/journal.pgen.1005137.
57. Liu, Q., Chen, J., Gao, X., Ding, J., Tang, Z., Zhang, C., Chen, J., Li, L., Chen, P., and Wang, J. (2015). Identification of

stage-specific markers during differentiation of hair cells from mouse inner ear stem cells or progenitor cells in vitro. Int. J. Biochem. Cell Biol. *60*, 99–111. https://doi.org/10.1016/j.biocel.2014.12.024.

58. Chen, T., Rohacek, A.M., Caporizzo, M., Nankali, A., Smits, J.J., Oostrik, J., Lanting, C.P., Kucuk, E., Gilissen, C., van de Kamp, J.M., et al. (2021). Cochlear supporting cells require GAS2 for cytoskeletal architecture and hearing. Dev. Cell *56*, 1526–1540.e7. https://doi.org/10.1016/j.devcel.2021.04.017.
59. Chan, D.K., and Chang, K.W. (2014). GJB2-associated hearing loss: systematic review of worldwide prevalence, genotype, and auditory phenotype. The Laryngoscope *124*, E34–E53. https://doi.org/10.1002/lary.24332.
60. Presacco, A., Simon, J.Z., and Anderson, S. (2016). Evidence of degraded representation of speech in noise, in the aging midbrain and cortex. J. Neurophysiol. *116*, 2346–2355. https://doi.org/10.1152/jn.00372.2016.
61. Loughrey, D.G., Kelly, M.E., Kelley, G.A., Brennan, S., and Lawlor, B.A. (2018). Association of age-related hearing loss with cognitive function, cognitive impairment, and dementia: a systematic review and meta-analysis. JAMA Otolaryngol. Head Neck Surg. *144*, 115–126. https://doi.org/10.1001/jamaoto.2017.2513.
62. Drummond, M.C., Belyantseva, I.A., Friderici, K.H., and Friedman, T.B. (2012). Actin in hair cells and hearing loss. Hearing Res. *288*, 89–99. https://doi.org/10.1016/j.heares.2011.12.003.
63. Liu, W., Johansson, A., Rask-Andersen, H., and Rask-Andersen, M. (2021). A combined genome-wide association and molecular study of age-related hearing loss in H. sapiens. BMC Med. *19*, 302. https://doi.org/10.1186/s12916-021-02169-0.
64. Liu, Y., Qi, J., Chen, X., Tang, M., Chu, C., Zhu, W., Li, H., Tian, C., Yang, G., Zhong, C., et al. (2019). Critical role of spectrin in hearing development and deafness. Sci. Adv. *5*, eaav7803. https://doi.org/10.1126/sciadv.aav7803.
65. Furman, A.C., Kujawa, S.G., and Liberman, M.C. (2013). Noise-induced cochlear neuropathy is selective for fibers with low spontaneous rates. J. Neurophysiol. *110*, 577–586. https://doi.org/10.1152/jn.00164.2013.
66. Kujawa, S.G., and Liberman, M.C. (2009). Adding insult to injury: cochlear nerve degeneration after "temporary" noise-induced hearing loss. J. Neurosci. *29*, 14077–14085. https://doi.org/10.1523/jneurosci.2845-09.2009.
67. Cederroth, C.R., Park, J.S., Basinou, V., Weger, B.D., Tserga, E., Sarlus, H., Magnusson, A.K., Kadri, N., Gachon, F., and Canlon, B. (2019). Circadian regulation of cochlear sensitivity to noise by circulating glucocorticoids. Curr. Biol. *29*, 2477–2487.e6. https://doi.org/10.1016/j.cub.2019.06.057.
68. Meltser, I., Cederroth, C.R., Basinou, V., Savelyev, S., Lundkvist, G.S., and Canlon, B. (2014). TrkB-mediated protection against circadian sensitivity to noise trauma in the murine cochlea. Curr. Biol. : CB *24*, 658–663. https://doi.org/10.1016/j.cub.2014.01.047.
69. Monaghan, J.J.M., Garcia-Lazaro, J.A., McAlpine, D., and Schaette, R. (2020). Hidden hearing loss impacts the neural representation of speech in background noise. Curr. Biol. *30*, 4710–4721.e4. https://doi.org/10.1016/j.cub.2020.09.046.
70. Schaette, R., and McAlpine, D. (2011). Tinnitus with a normal audiogram: physiological evidence for hidden hearing loss and computational model. J. Neurosci. *31*, 13452–13457. https://doi.org/10.1523/jneurosci.2156-11.2011.
71. Christov, F., Nelson, E.G., Xu, L.J., Lopez, I.A., Ishiyama, A., and Gluth, M.B. (2020). Histology of the cochlear outer sulcus cells in normal human ears, presbycusis, and meniere's disease. Otol. Neurotol. *41*, e507–e515. https://doi.org/10.1097/mao.0000000000002535.
72. Jagger, D.J., and Forge, A. (2013). The enigmatic root cell - emerging roles contributing to fluid homeostasis within the cochlear outer sulcus. Hearing Res. *303*, 1–11. https://doi.org/10.1016/j.heares.2012.10.010.
73. Eckhard, A., Gleiser, C., Rask-Andersen, H., Arnold, H., Liu, W., Mack, A., Muller, M., Lowenheim, H., and Hirt, B. (2012). Co-localisation of K(ir)4.1 and AQP4 in rat and human cochleae reveals a gap in water channel expression at the transduction sites of endocochlear K(+) recycling routes. Cell Tissue Res. *350*, 27–43. https://doi.org/10.1007/s00441-012-1456-y.
74. Kitajiri, S.I., Furuse, M., Morita, K., Saishin-Kiuchi, Y., Kido, H., Ito, J., and Tsukita, S. (2004). Expression patterns of claudins, tight junction adhesion molecules, in the inner ear. Hearing Res. *187*, 25–34. https://doi.org/10.1016/s0378-5955(03)00338-1.
75. Wu, P.Z., O'Malley, J.T., de Gruttola, V., and Liberman, M.C. (2020). Age-related hearing loss is dominated by damage to inner ear sensory cells, not the cellular battery that powers them. J. Neurosci. *40*, 6357–6366. https://doi.org/10.1523/jneurosci.0937-20.2020.
76. Wu, H., Petitpre, C., Fontanet, P., Sharma, A., Bellardita, C., Quadros, R.M., Jannig, P.R., Wang, Y., Heimel, J.A., Cheung, K.K.Y., et al. (2021). Distinct subtypes of proprioceptive dorsal root ganglion neurons regulate adaptive proprioception in mice. Nat. Commun. *12*, 1026. https://doi.org/10.1038/s41467-021-21173-9.
77. Sharma, N., Flaherty, K., Lezgiyeva, K., Wagner, D.E., Klein, A.M., and Ginty, D.D. (2020). The emergence of transcriptional identity in somatosensory neurons. Nature *577*, 392–398. https://doi.org/10.1038/s41586-019-1900-1.
78. Kusunoki, T., Cureoglu, S., Schachern, P.A., Baba, K., Kariya, S., and Paparella, M.M. (2004). Age-related histopathologic changes in the human cochlea: a temporal bone study. Otolaryngol. Head Neck Surg. *131*, 897–903. https://doi.org/10.1016/j.otohns.2004.05.022.
79. Furness, D.N., Lawton, D.M., Mahendrasingam, S., Hodierne, L., and Jagger, D.J. (2009). Quantitative analysis of the expression of the glutamate-aspartate transporter and identification of functional glutamate uptake reveal a role for cochlear fibrocytes in glutamate homeostasis. Neuroscience *162*, 1307–1321. https://doi.org/10.1016/j.neuroscience.2009.05.036.
80. Smits, C., Theo Goverts, S., and Festen, J.M. (2013). The digits-in-noise test: assessing auditory speech recognition abilities in noise. J. Acoust. Soc. America *133*, 1693–1706. https://doi.org/10.1121/1.4789933.
81. Halvorsen, M., Huh, R., Oskolkov, N., Wen, J., Netotea, S., Giusti-Rodriguez, P., Karlsson, R., Bryois, J., Nystedt, B., Ameur, A., et al. (2020). Increased burden of ultra-rare structural variants localizing to boundaries of topologically associated domains in schizophrenia. Nat. Commun. *11*, 1842. https://doi.org/10.1038/s41467-020-15707-w.

The American Journal of Human Genetics, Volume *109*

Supplemental information

Genome-wide association meta-analysis identifies 48 risk variants and highlights the role of the stria vascularis in hearing loss

Natalia Trpchevska, Maxim B. Freidin, Linda Broer, Berthe C. Oosterloo, Shuyang Yao, Yitian Zhou, Barbara Vona, Charles Bishop, Argyro Bizaki-Vallaskangas, Barbara Canlon, Fabio Castellana, Daniel I. Chasman, Stacey Cherny, Kaare Christensen, Maria Pina Concas, Adolfo Correa, Ran Elkon, Estonian Biobank Research Team, Jonas Mengel-From, Yan Gao, Anne B.S. Giersch, Giorgia Girotto, Alexander Gudjonsson, Vilmundur Gudnason, Nancy L. Heard-Costa, Ronna Hertzano, Jacob v.B. Hjelmborg, Jens Hjerling-Leffler, Howard J. Hoffman, Jaakko Kaprio, Johannes Kettunen, Kristi Krebs, Anna K. Kähler, Francois Lallemend, Lenore J. Launer, I-Min Lee, Hampton Leonard, Chuan-Ming Li, Hubert Lowenheim, Patrik K.E. Magnusson, Joyce van Meurs, Lili Milani, Cynthia C. Morton, Antti Mäkitie, Mike A. Nalls, Giuseppe Giovanni Nardone, Marianne Nygaard, Teemu Palviainen, Sheila Pratt, Nicola Quaranta, Joel Rämö, Elmo Saarentaus, Rodolfo Sardone, Claudia L. Satizabal, John M. Schweinfurth, Sudha Seshadri, Eric Shiroma, Eldad Shulman, Eleanor Simonsick, Christopher Spankovich, Anke Tropitzsch, Volker M. Lauschke, Patrick F. Sullivan, Andre Goedegebure, Christopher R. Cederroth, Frances M.K. Williams, and Andries Paul Nagtegaal

Supplementary information

Figure S1. Quantile-Quantile plot of association test results from meta-analysis of hearing impairment

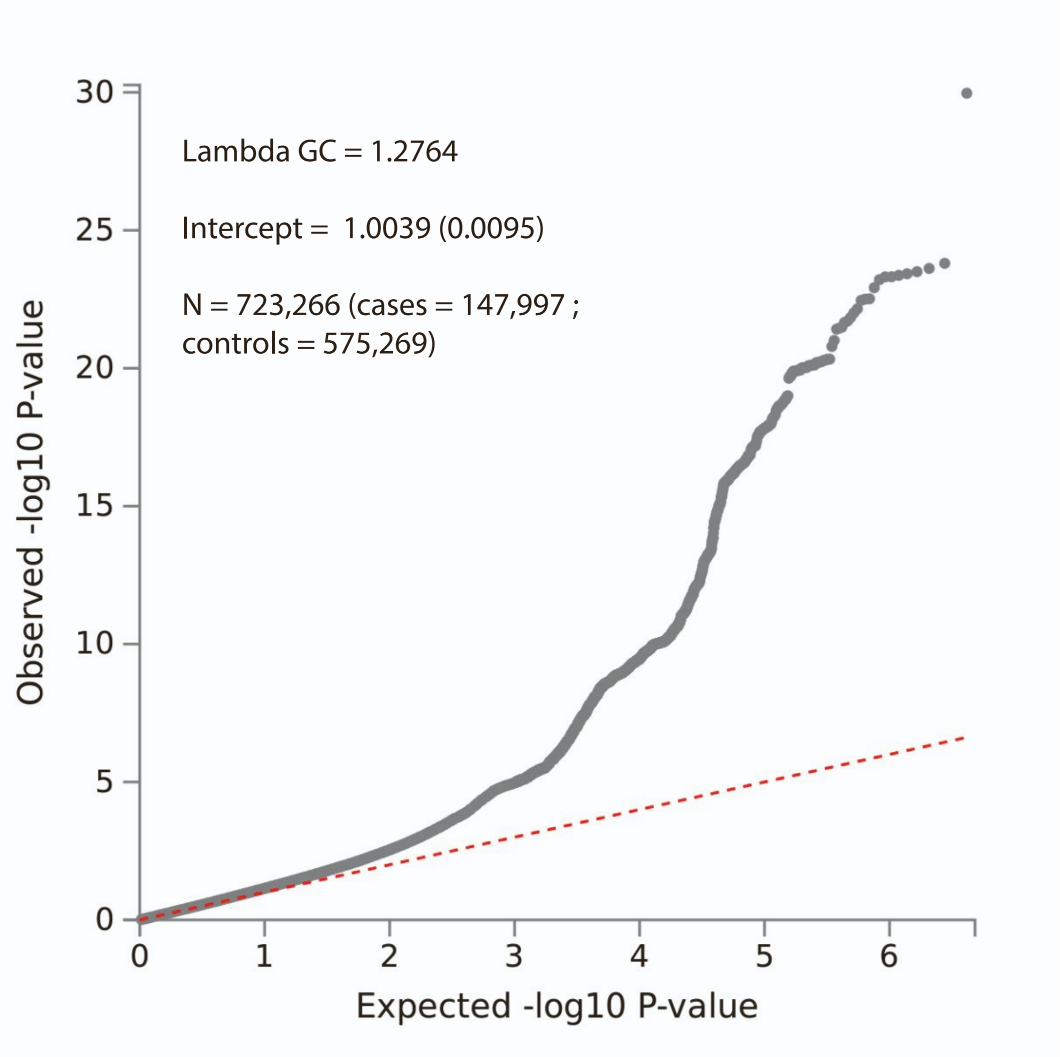

Figure S2 Locus Zoom plot of each statistically significant and independent SNP

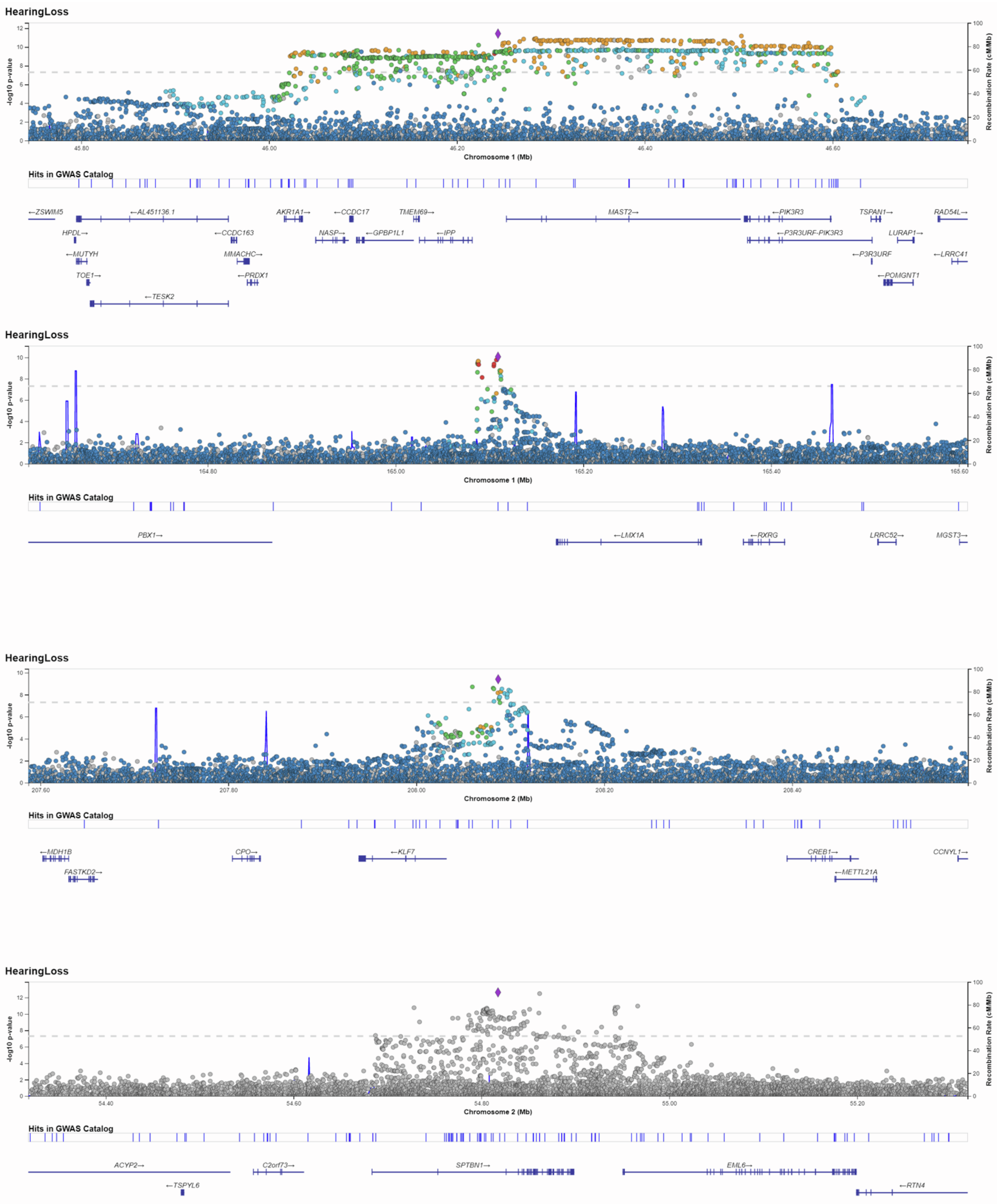

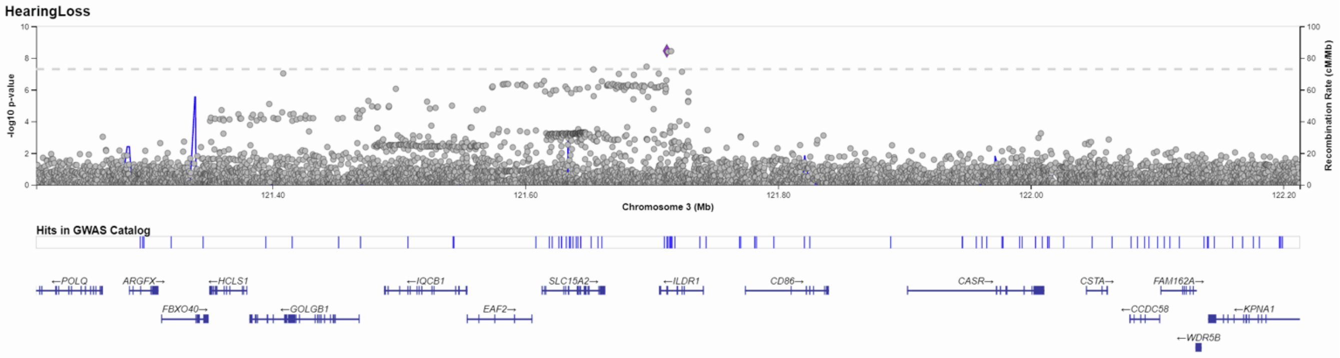
HearingLoss
-log10 p-value
Recombination Rate (cM/Mb)
Chromosome 3 (Mb)
121.40
121.60
121.80
122.00
122.20
Hits in GWAS Catalog
←POLQ
ARGFX→
←HCLS1
←IQCB1
SLC15A2→
←ILDR1
CD86→
CASR→
CSTA→
FAM162A→
FBXO40→
←GOLGB1
EAF2→
←CCDC58
←KPNA1
←WDR5B

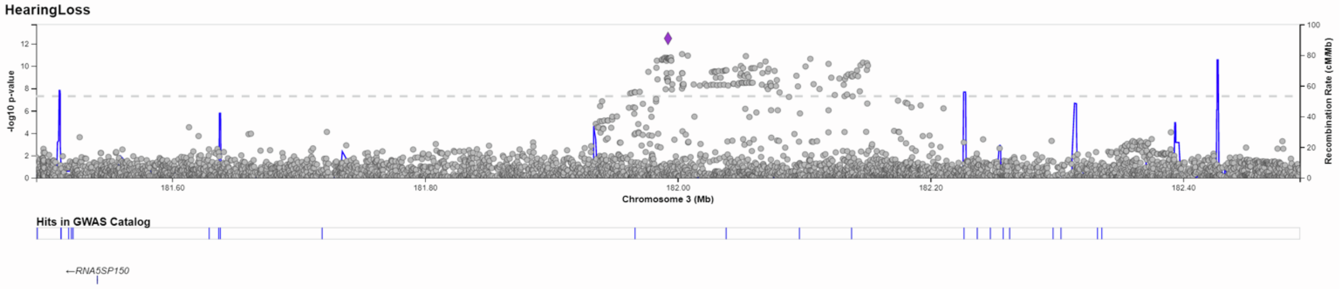
HearingLoss
-log10 p-value
Recombination Rate (cM/Mb)
Chromosome 3 (Mb)
181.60
181.80
182.00
182.20
182.40
Hits in GWAS Catalog
←RNA5SP150

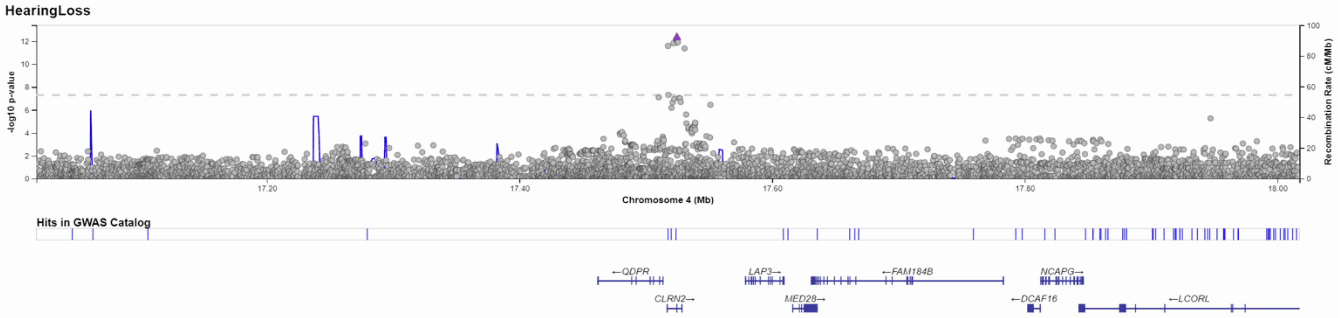
HearingLoss
-log10 p-value
Recombination Rate (cM/Mb)
Chromosome 4 (Mb)
17.20
17.40
17.60
17.80
18.00
Hits in GWAS Catalog
←QDPR
LAP3→
←FAM184B
NCAPG→
CLRN2→
MED28→
←DCAF16
←LCORL

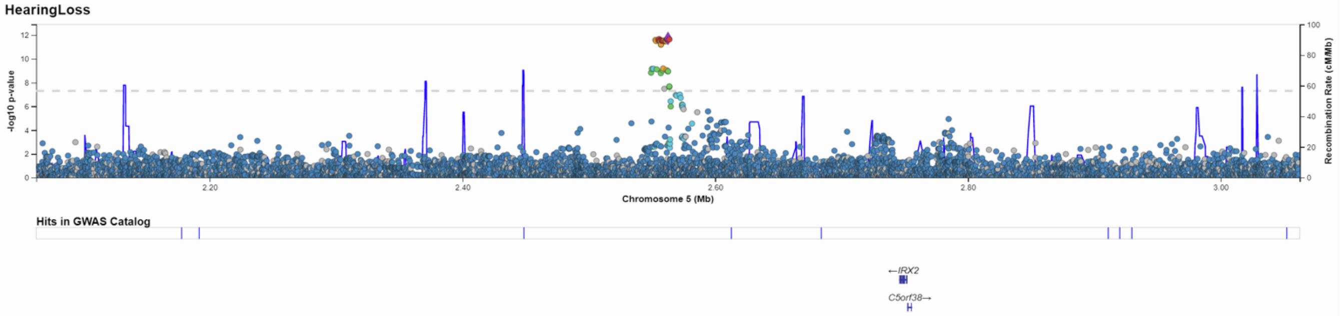
HearingLoss
-log10 p-value
Recombination Rate (cM/Mb)
Chromosome 5 (Mb)
2.20
2.40
2.60
2.80
3.00
Hits in GWAS Catalog
←IRX2
C5orf38→

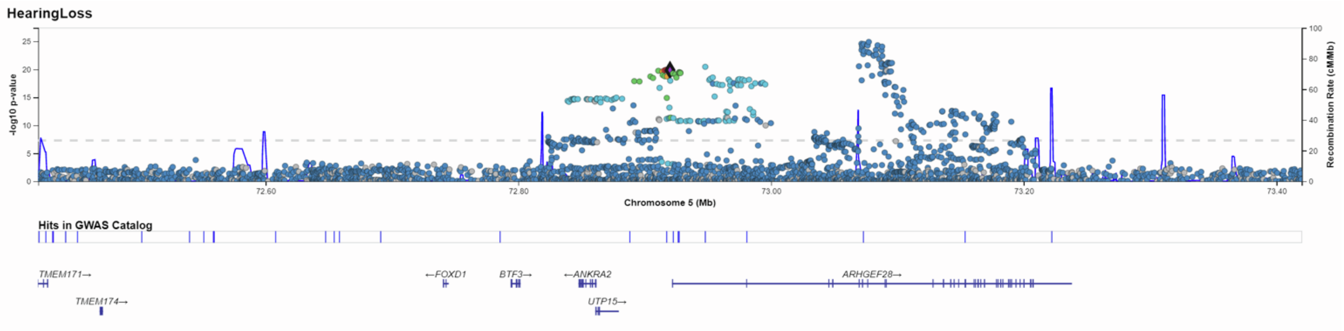
HearingLoss
-log10 p-value
Recombination Rate (cM/Mb)
Chromosome 5 (Mb)
Hits in GWAS Catalog
TMEM171→
TMEM174→
←FOXD1
BTF3→
←ANKRA2
UTP15→
ARHGEF28→

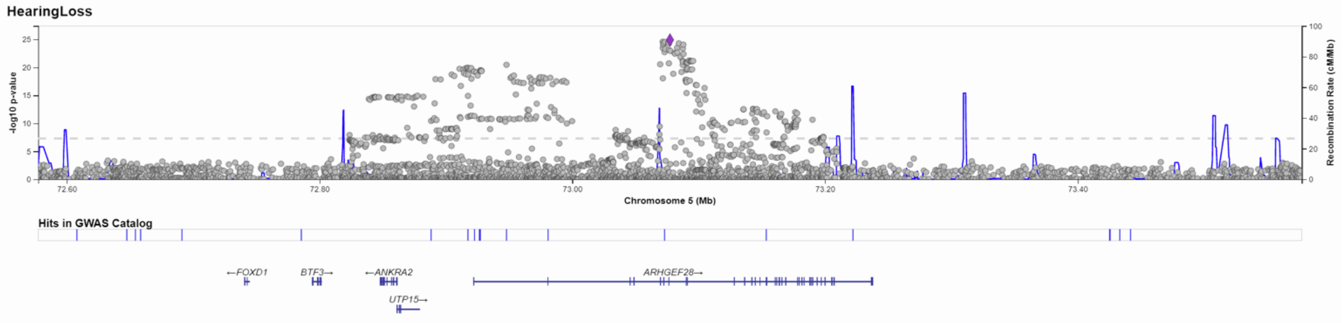
HearingLoss
-log10 p-value
Recombination Rate (cM/Mb)
Chromosome 5 (Mb)
Hits in GWAS Catalog
←FOXD1
BTF3→
←ANKRA2
UTP15→
ARHGEF28→

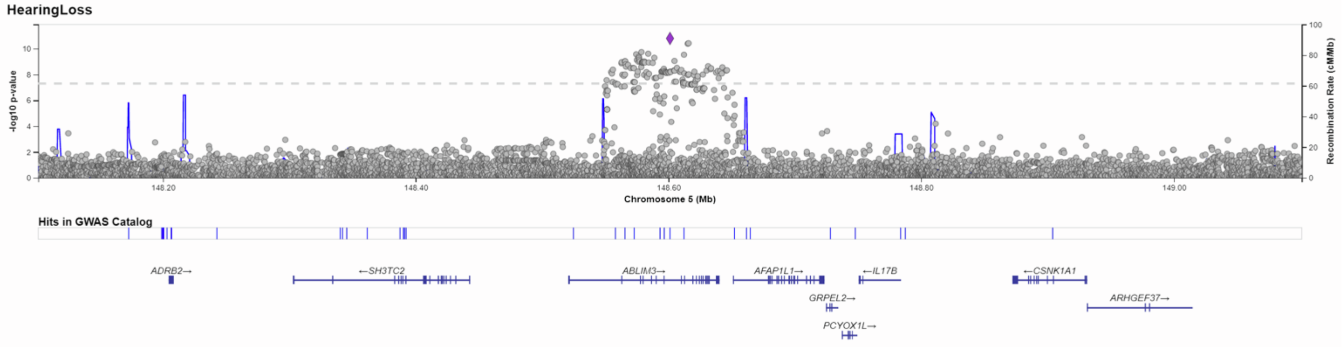
HearingLoss
-log10 p-value
Recombination Rate (cM/Mb)
Chromosome 5 (Mb)
Hits in GWAS Catalog
ADRB2→
←SH3TC2
ABLIM3→
AFAP1L1→
←IL17B
←CSNK1A1
GRPEL2→
PCYOX1L→
ARHGEF37→

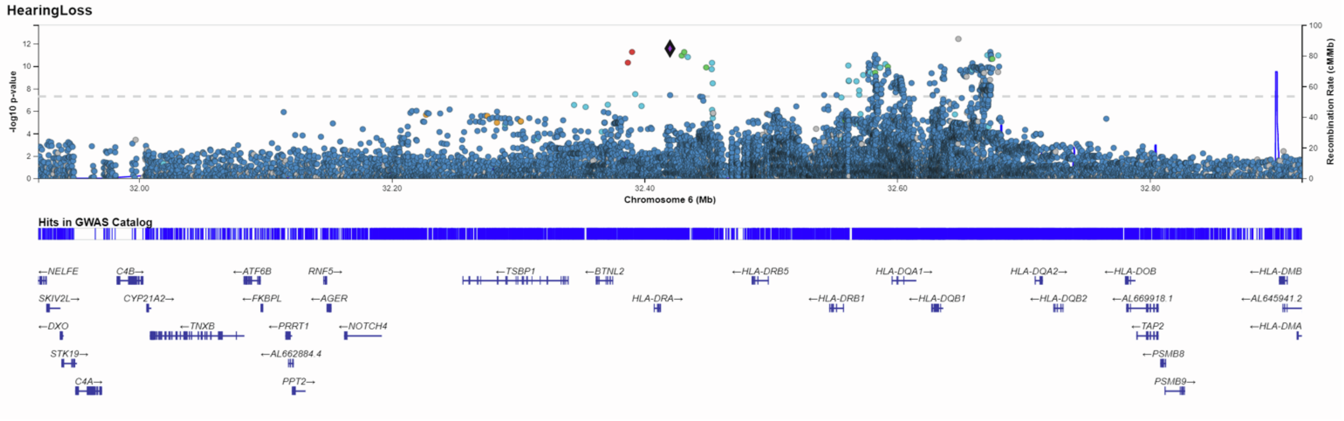
HearingLoss
-log10 p-value
Recombination Rate (cM/Mb)
Chromosome 6 (Mb)
Hits in GWAS Catalog
←NELFE
C4B→
←ATF6B
RNF5→
←TSBP1
←BTNL2
←HLA-DRB5
HLA-DQA1→
HLA-DQA2→
←HLA-DOB
←HLA-DMB
SKIV2L→
CYP21A2→
←FKBPL
←AGER
HLA-DRA→
←HLA-DRB1
←HLA-DQB1
←HLA-DQB2
←AL669918.1
←AL645941.2
←DXO
←TNXB
←PRRT1
←NOTCH4
←TAP2
←HLA-DMA
STK19→
←AL662884.4
←PSMB8
C4A→
PPT2→
PSMB9→

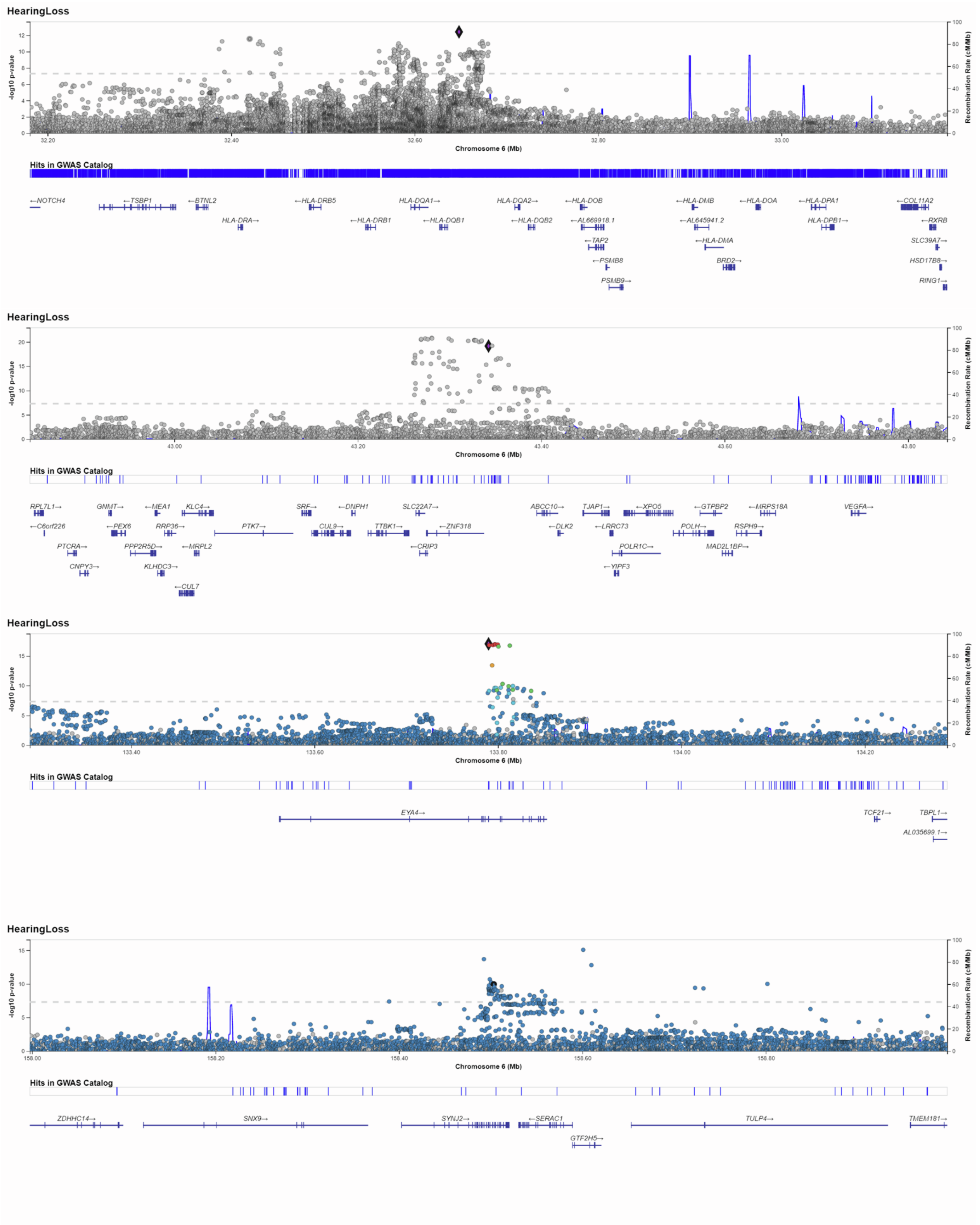
HearingLoss
-log10 p-value
Recombination Rate (cM/Mb)
Chromosome 6 (Mb)
Hits in GWAS Catalog
←NOTCH4
←TSBP1
←BTNL2
HLA-DRA→
←HLA-DRB5
←HLA-DRB1
HLA-DQA1→
←HLA-DQB1
HLA-DQA2→
←HLA-DQB2
←HLA-DOB
←AL669918.1
←TAP2
←PSMB8
PSMB9→
←HLA-DMB
←AL645941.2
←HLA-DMA
BRD2→
←HLA-DOA
←HLA-DPA1
HLA-DPB1→
←COL11A2
←RXRB
SLC39A7→
HSD17B8→
RING1→
HearingLoss
-log10 p-value
Recombination Rate (cM/Mb)
Chromosome 6 (Mb)
Hits in GWAS Catalog
RPL7L1→
←C6orf226
PTCRA→
CNPY3→
GNMT→
←PEX6
PPP2R5D→
←MEA1
RRP36→
KLHDC3→
KLC4→
←MRPL2
←CUL7
PTK7→
SRF→
CUL9→
←DNPH1
TTBK1→
SLC22A7→
←CRIP3
←ZNF318
ABCC10→
←DLK2
TJAP1→
←LRRC73
←YIPF3
←XPO5
POLR1C→
←GTPBP2
POLH→
←MRPS18A
RSPH9→
MAD2L1BP→
VEGFA→
HearingLoss
-log10 p-value
Recombination Rate (cM/Mb)
Chromosome 6 (Mb)
Hits in GWAS Catalog
EYA4→
TCF21→
TBPL1→
AL035699.1→
HearingLoss
-log10 p-value
Recombination Rate (cM/Mb)
Chromosome 6 (Mb)
Hits in GWAS Catalog
ZDHHC14→
SNX9→
SYNJ2→
←SERAC1
GTF2H5→
TULP4→
TMEM181→

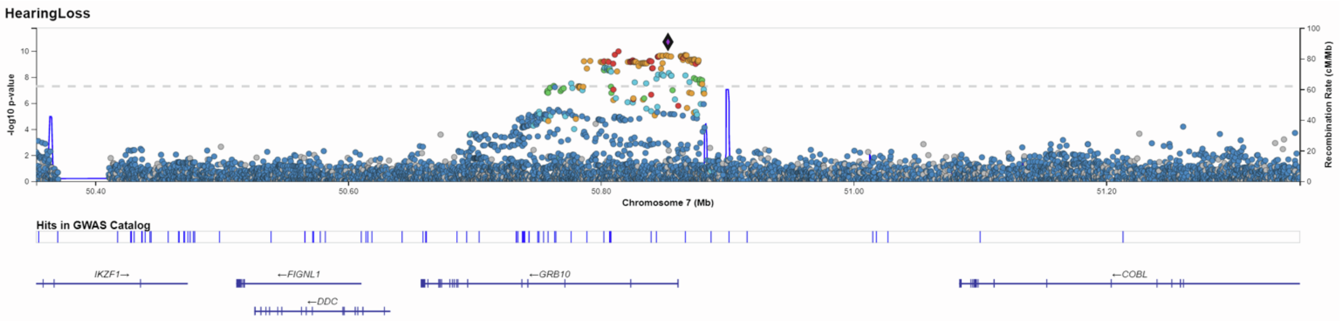
HearingLoss
-log10 p-value
Recombination Rate (cM/Mb)
Chromosome 7 (Mb)
Hits in GWAS Catalog
IKZF1→
←FIGNL1
←DDC
←GRB10
←COBL

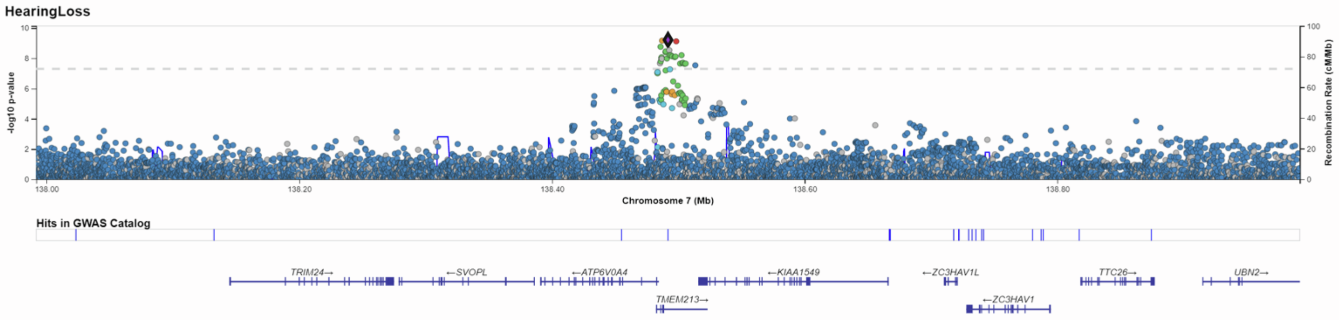
HearingLoss
-log10 p-value
Recombination Rate (cM/Mb)
Chromosome 7 (Mb)
Hits in GWAS Catalog
TRIM24→
←SVOPL
←ATP6V0A4
TMEM213→
←KIAA1549
←ZC3HAV1L
←ZC3HAV1
TTC26→
UBN2→

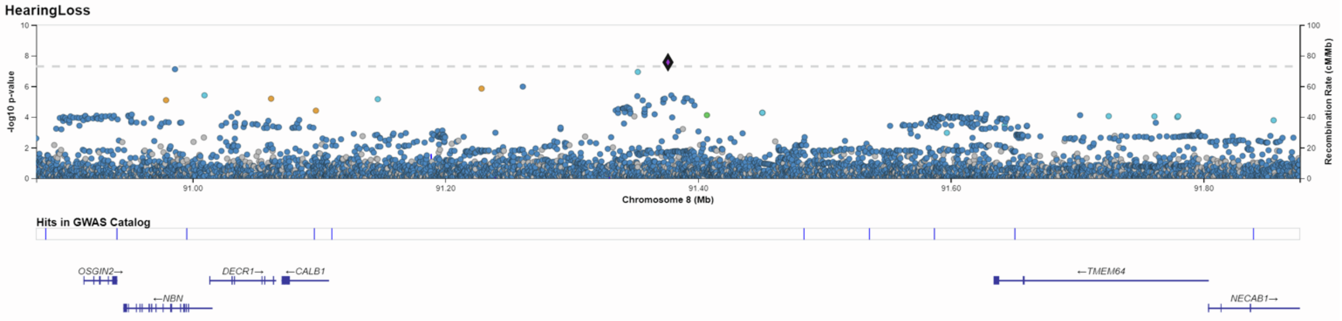
HearingLoss
-log10 p-value
Recombination Rate (cM/Mb)
Chromosome 8 (Mb)
Hits in GWAS Catalog
OSGIN2→
←NBN
DECR1→
←CALB1
←TMEM64
NECAB1→

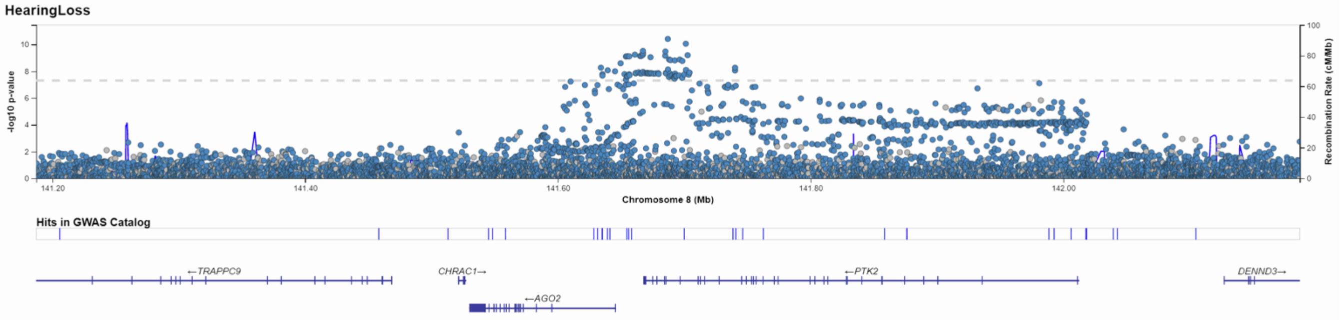
HearingLoss
-log10 p-value
Recombination Rate (cM/Mb)
Chromosome 8 (Mb)
Hits in GWAS Catalog
←TRAPPC9
CHRAC1→
←AGO2
←PTK2
DENND3→

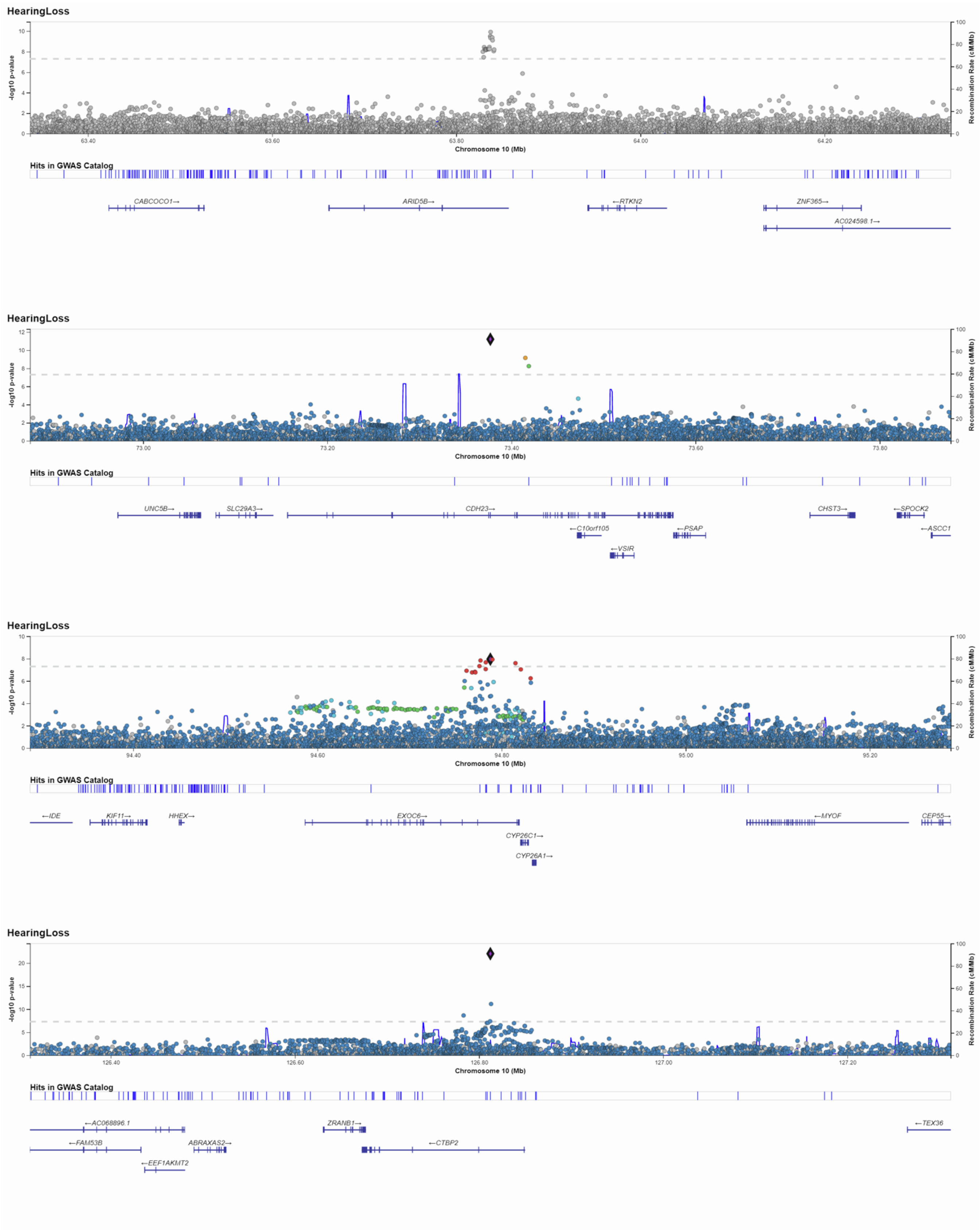
HearingLoss
-log10 p-value
Recombination Rate (cM/Mb)
Chromosome 10 (Mb)
Hits in GWAS Catalog
CABCOCO1→
ARID5B→
←RTKN2
ZNF365→
AC024598.1→
HearingLoss
-log10 p-value
Recombination Rate (cM/Mb)
Chromosome 10 (Mb)
Hits in GWAS Catalog
UNC5B→
SLC29A3→
CDH23→
CHST3→
←SPOCK2
←C10orf105
←PSAP
←ASCC1
←VSIR
HearingLoss
-log10 p-value
Recombination Rate (cM/Mb)
Chromosome 10 (Mb)
Hits in GWAS Catalog
←IDE
KIF11→
HHEX→
EXOC6→
←MYOF
CEP55→
CYP26C1→
CYP26A1→
HearingLoss
-log10 p-value
Recombination Rate (cM/Mb)
Chromosome 10 (Mb)
Hits in GWAS Catalog
←AC068896.1
ZRANB1→
←TEX36
←FAM53B
ABRAXAS2→
←CTBP2
←EEF1AKMT2

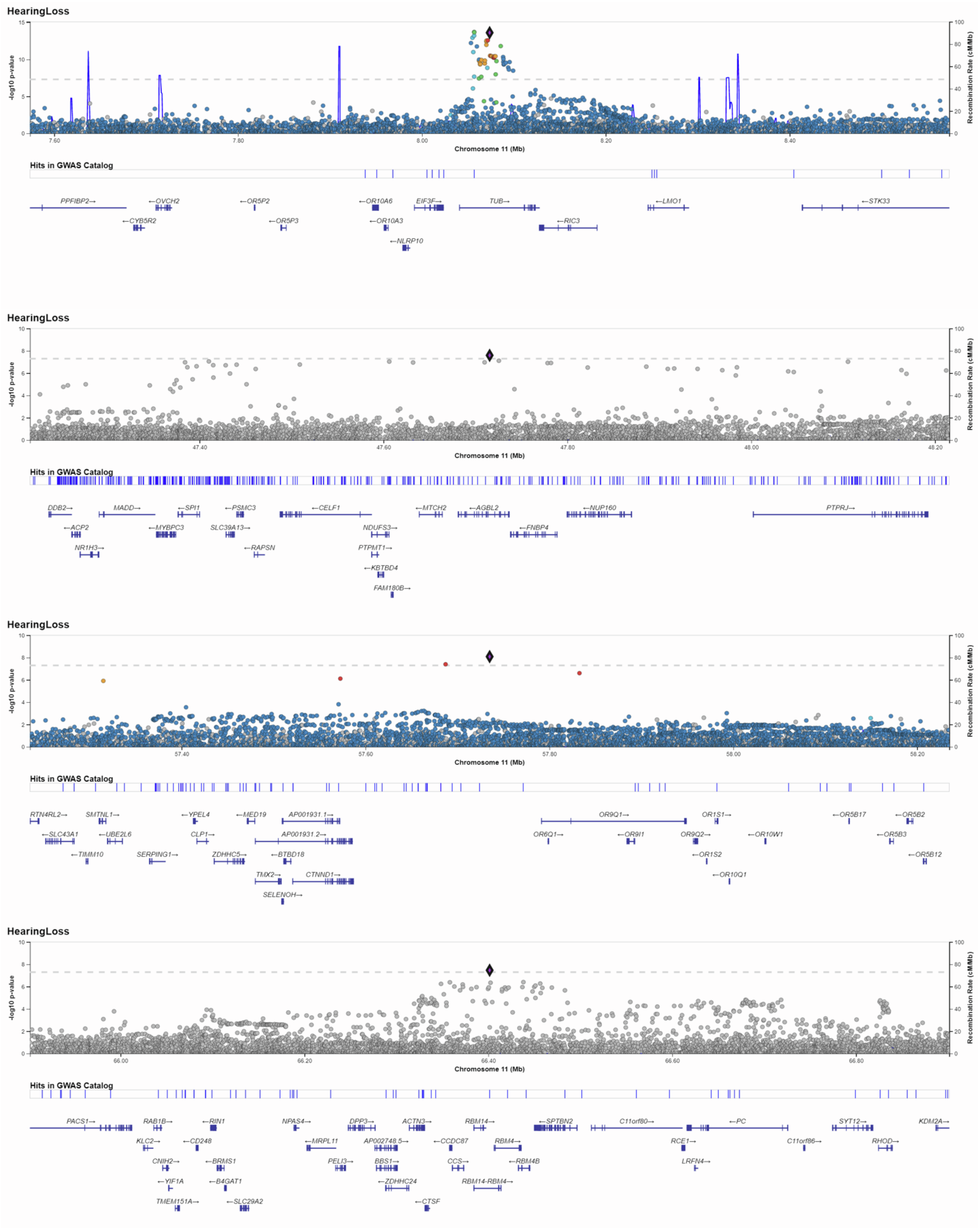
HearingLoss
-log10 p-value
Recombination Rate (cM/Mb)
Chromosome 11 (Mb)
Hits in GWAS Catalog
PPFIBP2→
←OVCH2
←CYB5R2
←OR5P2
←OR5P3
←OR10A6
←OR10A3
←NLRP10
EIF3F→
TUB→
←RIC3
←LMO1
←STK33
HearingLoss
-log10 p-value
Recombination Rate (cM/Mb)
Chromosome 11 (Mb)
Hits in GWAS Catalog
DDB2→
←ACP2
NR1H3→
MADD→
←MYBPC3
←SPI1
←PSMC3
SLC39A13→
←RAPSN
←CELF1
NDUFS3→
PTPMT1→
←KBTBD4
FAM180B→
←MTCH2
←AGBL2
←FNBP4
←NUP160
PTPRJ→
HearingLoss
-log10 p-value
Recombination Rate (cM/Mb)
Chromosome 11 (Mb)
Hits in GWAS Catalog
RTN4RL2→
←SLC43A1
←TIMM10
SMTNL1→
←UBE2L6
SERPING1→
←YPEL4
CLP1→
ZDHHC5→
←MED19
TMX2→
SELENOH→
←BTBD18
AP001931.1→
AP001931.2→
CTNND1→
OR9Q1→
OR6Q1→
←OR9I1
OR1S1→
OR9Q2→
←OR1S2
←OR10Q1
←OR10W1
←OR5B17
←OR5B3
←OR5B2
←OR5B12
HearingLoss
-log10 p-value
Recombination Rate (cM/Mb)
Chromosome 11 (Mb)
Hits in GWAS Catalog
PACS1→
RAB1B→
KLC2→
CNIH2→
←YIF1A
TMEM151A→
←RIN1
←CD248
←BRMS1
←B4GAT1
←SLC29A2
NPAS4→
←MRPL11
PELI3→
DPP3→
AP002748.5→
BBS1→
←ZDHHC24
ACTN3→
←CCDC87
CCS→
←CTSF
RBM14→
RBM4→
←RBM4B
RBM14-RBM4→
←SPTBN2
C11orf80→
RCE1→
LRFN4→
←PC
C11orf86→
SYT12→
RHOD→
KDM2A→

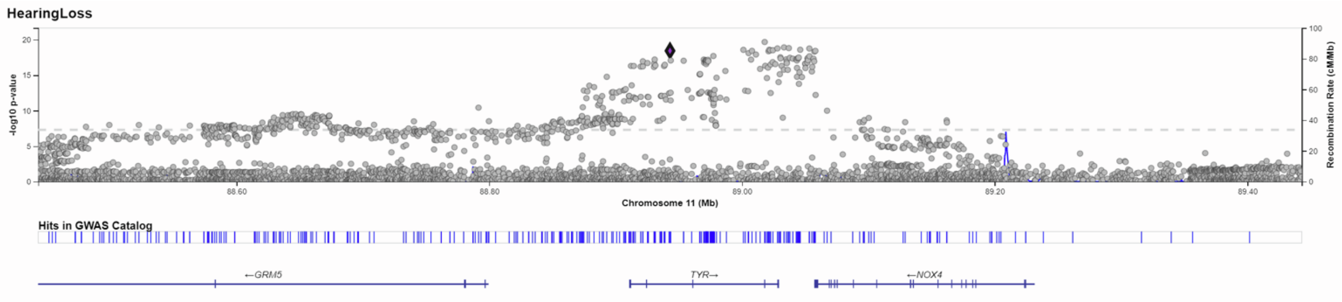
HearingLoss
-log10 p-value
Recombination Rate (cM/Mb)
Chromosome 11 (Mb)
Hits in GWAS Catalog
←GRM5
TYR→
←NOX4

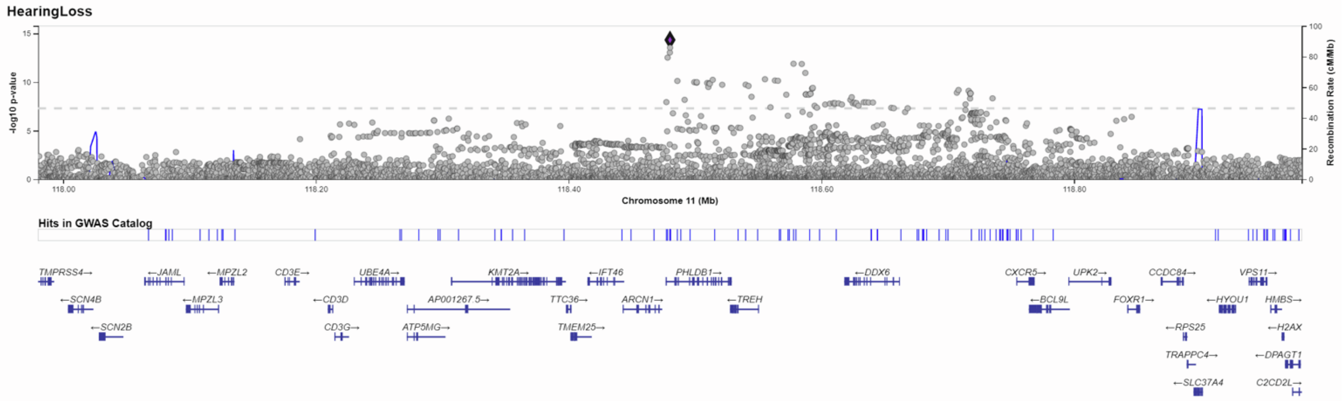
HearingLoss
-log10 p-value
Recombination Rate (cM/Mb)
Chromosome 11 (Mb)
Hits in GWAS Catalog
TMPRSS4→
←JAML
←MPZL2
CD3E→
UBE4A→
KMT2A→
←IFT46
PHLDB1→
←DDX6
CXCR5→
UPK2→
CCDC84→
VPS11→
←SCN4B
←MPZL3
←CD3D
AP001267.5→
TTC36→
ARCN1→
←TREH
←BCL9L
FOXR1→
←HYOU1
HMBS→
←SCN2B
CD3G→
ATP5MG→
TMEM25→
←RPS25
←H2AX
TRAPPC4→
←DPAGT1
←SLC37A4
C2CD2L→

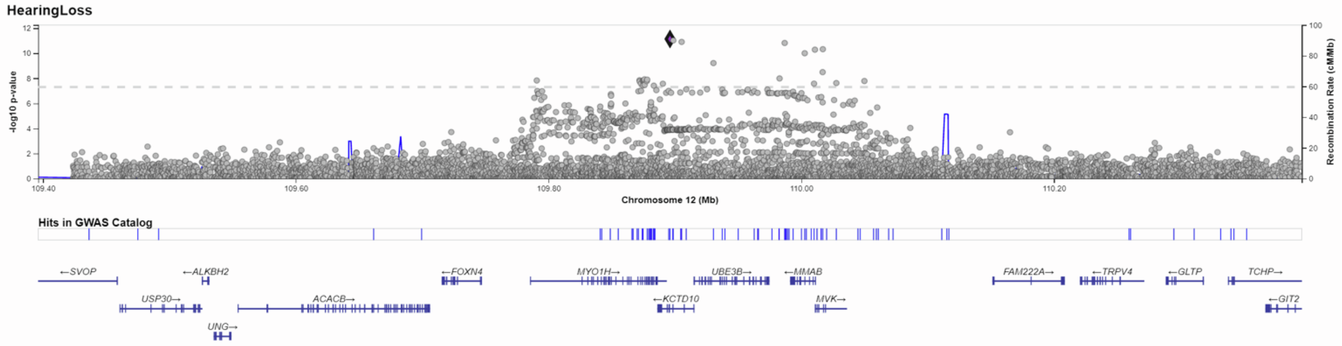
HearingLoss
-log10 p-value
Recombination Rate (cM/Mb)
Chromosome 12 (Mb)
Hits in GWAS Catalog
←SVOP
←ALKBH2
←FOXN4
MYO1H→
UBE3B→
←MMAB
FAM222A→
←TRPV4
←GLTP
TCHP→
USP30→
ACACB→
←KCTD10
MVK→
←GIT2
UNG→

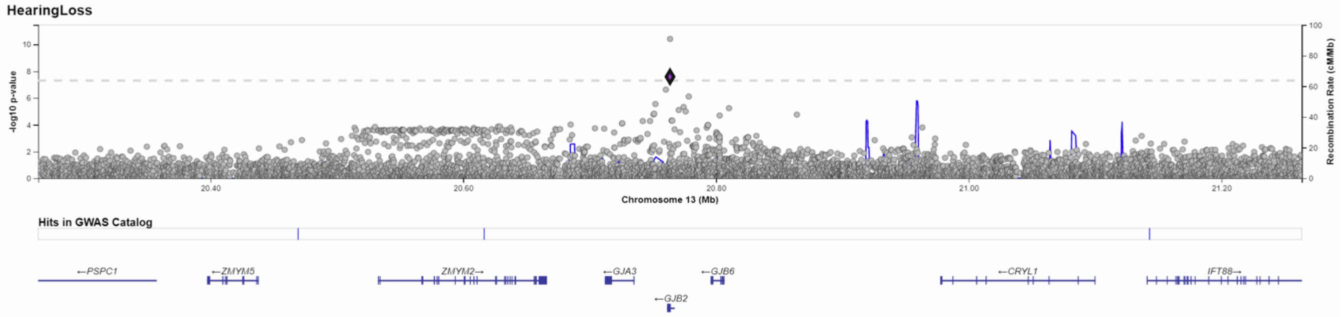
HearingLoss
-log10 p-value
Recombination Rate (cM/Mb)
Chromosome 13 (Mb)
Hits in GWAS Catalog
←PSPC1
←ZMYM5
ZMYM2→
←GJA3
←GJB6
←GJB2
←CRYL1
IFT88→

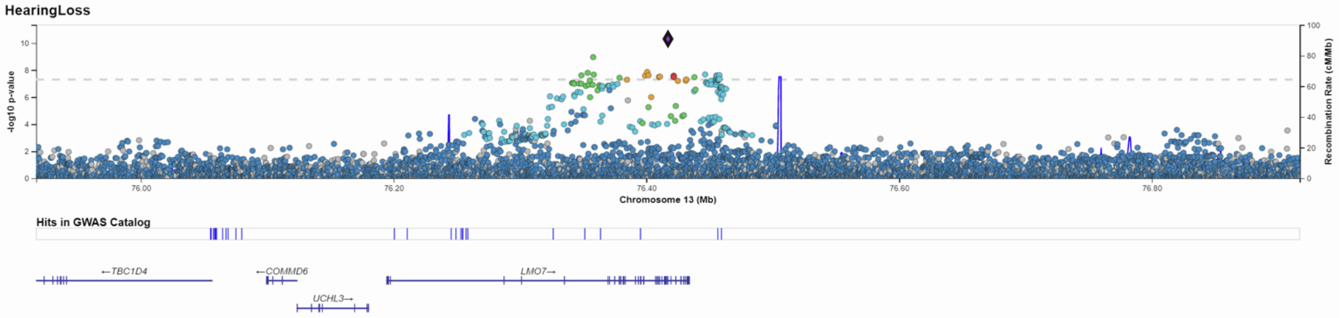
HearingLoss
-log10 p-value
Recombination Rate (cM/Mb)
Chromosome 13 (Mb)
Hits in GWAS Catalog
←TBC1D4
←COMMD6
UCHL3→
LMO7→

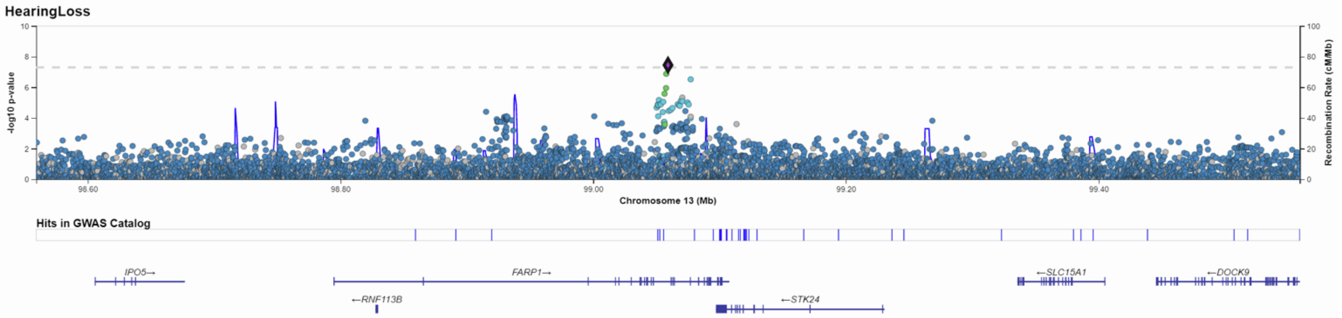
HearingLoss
-log10 p-value
Recombination Rate (cM/Mb)
Chromosome 13 (Mb)
Hits in GWAS Catalog
IPO5→
FARP1→
←RNF113B
←STK24
←SLC15A1
←DOCK9

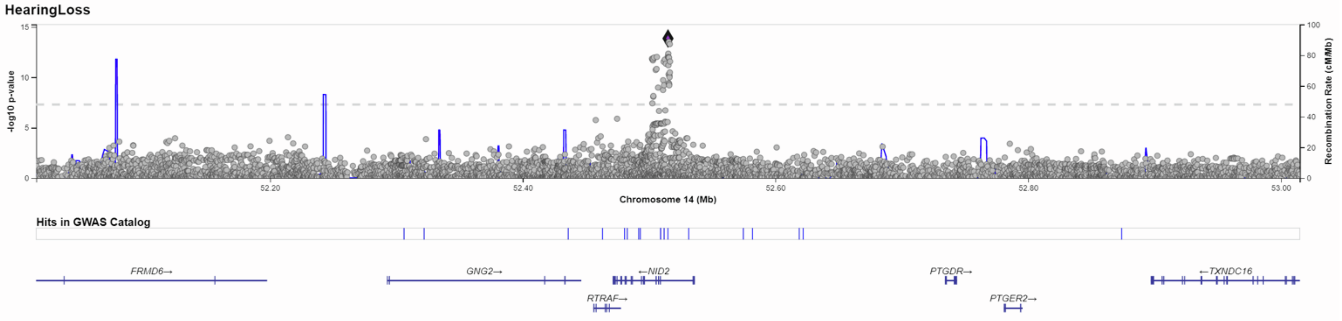
HearingLoss
-log10 p-value
Recombination Rate (cM/Mb)
Chromosome 14 (Mb)
Hits in GWAS Catalog
FRMD6→
GNG2→
←NID2
RTRAF→
PTGDR→
PTGER2→
←TXNDC16

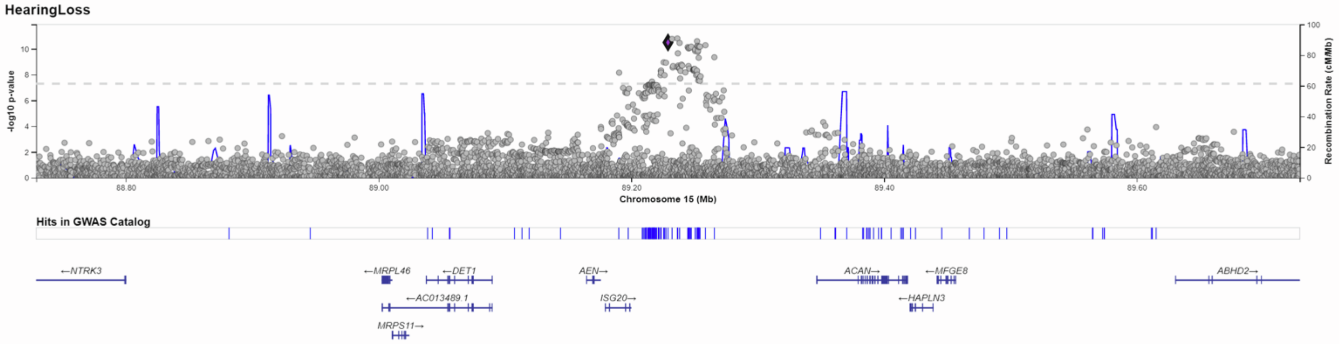
HearingLoss
-log10 p-value
Recombination Rate (cM/Mb)
Chromosome 15 (Mb)
Hits in GWAS Catalog
←NTRK3
←MRPL46
←DET1
←AC013489.1
MRPS11→
AEN→
ISG20→
ACAN→
←MFGE8
←HAPLN3
ABHD2→

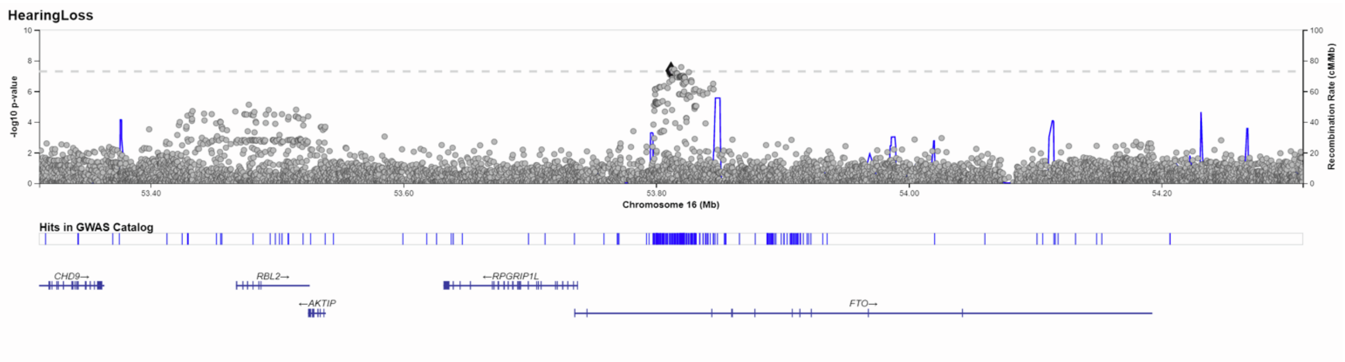
HearingLoss
-log10 p-value
Recombination Rate (cM/Mb)
Chromosome 16 (Mb)
53.40
53.60
53.80
54.00
54.20
Hits in GWAS Catalog
CHD9→
RBL2→
←AKTIP
←RPGRIP1L
FTO→

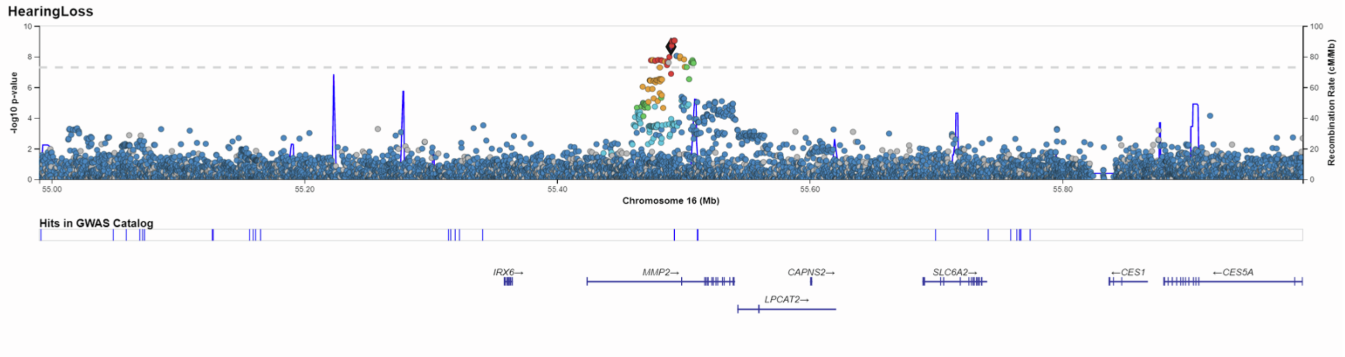
HearingLoss
-log10 p-value
Recombination Rate (cM/Mb)
Chromosome 16 (Mb)
55.00
55.20
55.40
55.60
55.80
Hits in GWAS Catalog
IRX6→
MMP2→
CAPNS2→
LPCAT2→
SLC6A2→
←CES1
←CES5A

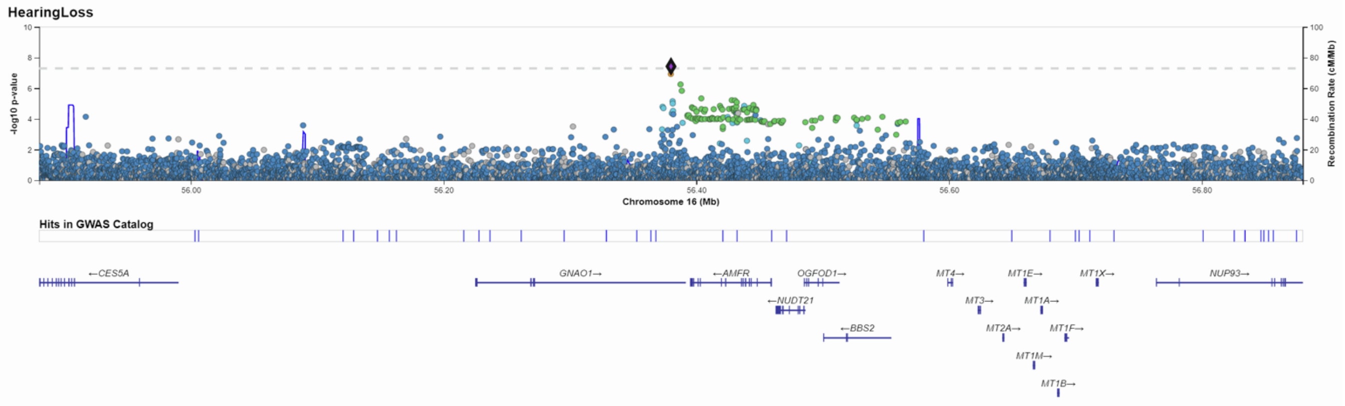
HearingLoss
-log10 p-value
Recombination Rate (cM/Mb)
Chromosome 16 (Mb)
56.00
56.20
56.40
56.60
56.80
Hits in GWAS Catalog
←CES5A
GNAO1→
←AMFR
OGFOD1→
←NUDT21
←BBS2
MT4→
MT3→
MT2A→
MT1E→
MT1M→
MT1A→
MT1B→
MT1X→
MT1F→
NUP93→

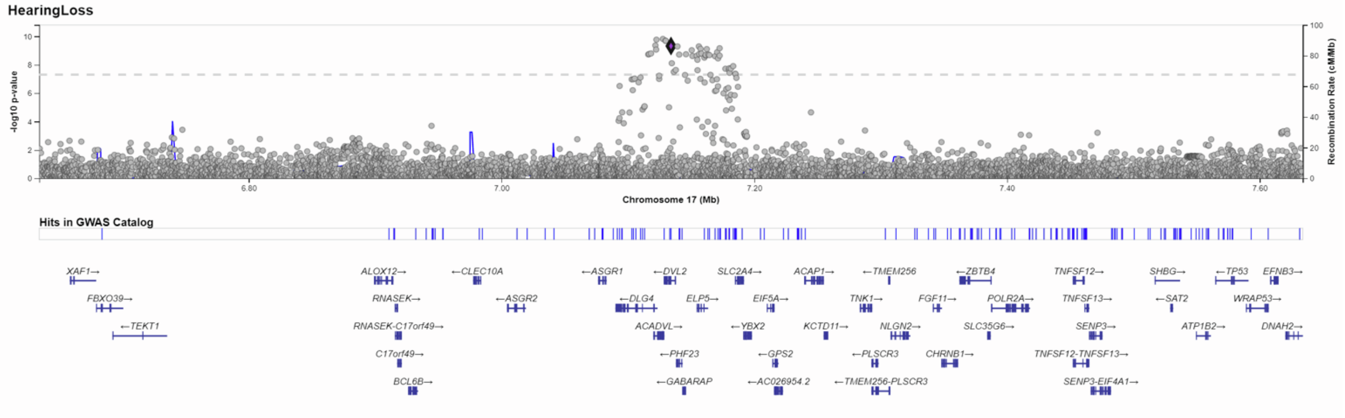
HearingLoss
-log10 p-value
Recombination Rate (cM/Mb)
Chromosome 17 (Mb)
6.80
7.00
7.20
7.40
7.60
Hits in GWAS Catalog
XAF1→
FBXO39→
←TEKT1
ALOX12→
RNASEK→
RNASEK-C17orf49→
C17orf49→
BCL6B→
←CLEC10A
←ASGR2
←ASGR1
←DLG4
ACADVL→
←DVL2
←PHF23
←GABARAP
ELP5→
SLC2A4→
←YBX2
EIF5A→
←GPS2
←AC026954.2
ACAP1→
KCTD11→
←TMEM256
TNK1→
←PLSCR3
←TMEM256-PLSCR3
NLGN2→
←ZBTB4
FGF11→
CHRNB1→
POLR2A→
SLC35G6→
TNFSF12→
TNFSF13→
SENP3→
TNFSF12-TNFSF13→
SENP3-EIF4A1→
SHBG→
←SAT2
←TP53
ATP1B2→
EFNB3→
WRAP53→
DNAH2→

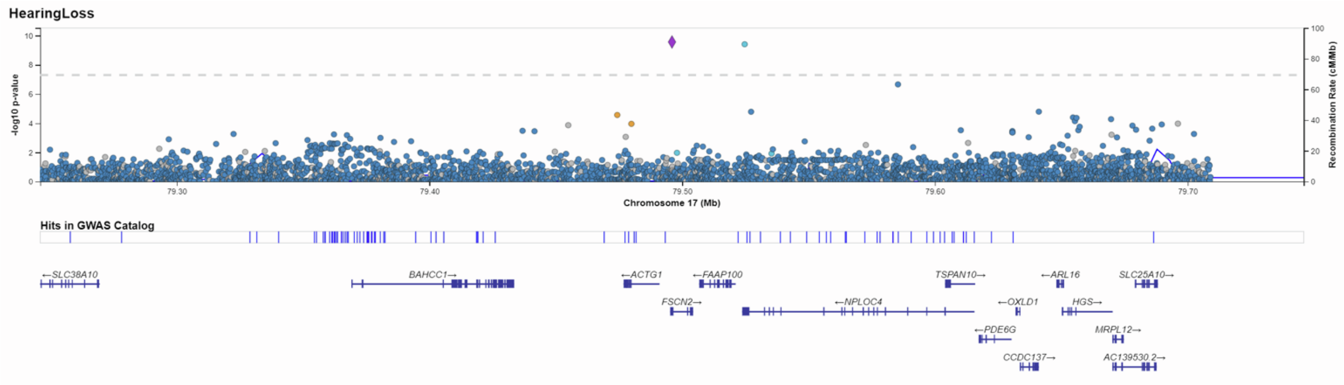
HearingLoss
-log10 p-value
Recombination Rate (cM/Mb)
Chromosome 17 (Mb)
Hits in GWAS Catalog
←SLC38A10
BAHCC1→
←ACTG1
←FAAP100
FSCN2→
←NPLOC4
TSPAN10→
←ARL16
SLC25A10→
←OXLD1
HGS→
←PDE6G
MRPL12→
CCDC137→
AC139530.2→

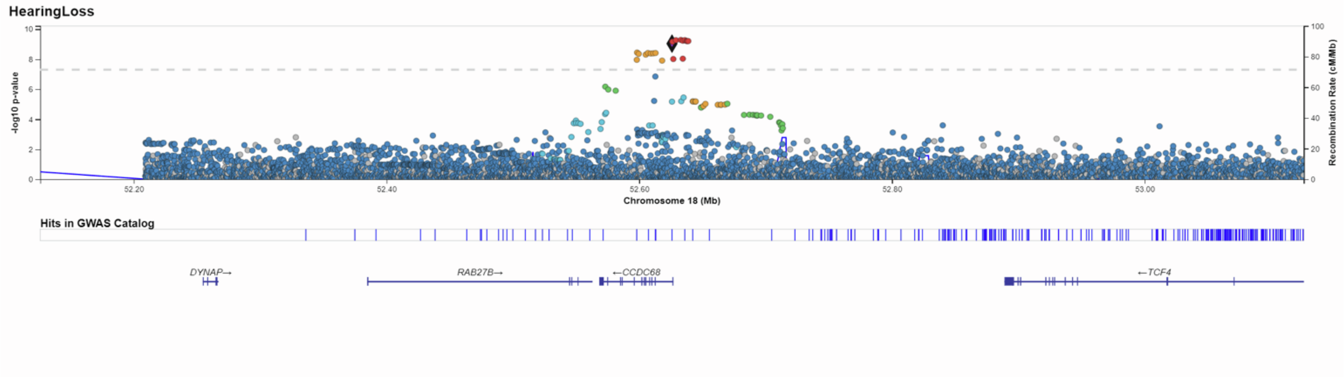
HearingLoss
-log10 p-value
Recombination Rate (cM/Mb)
Chromosome 18 (Mb)
Hits in GWAS Catalog
DYNAP→
RAB27B→
←CCDC68
←TCF4

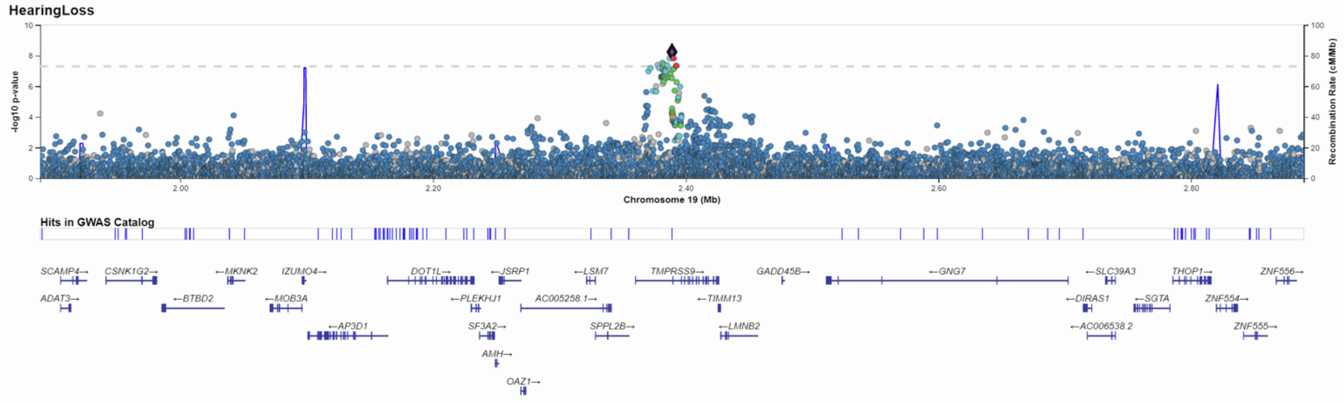
HearingLoss
-log10 p-value
Recombination Rate (cM/Mb)
Chromosome 19 (Mb)
Hits in GWAS Catalog
SCAMP4→
CSNK1G2→
←MKNK2
IZUMO4→
DOT1L→
←JSRP1
←LSM7
TMPRSS9→
GADD45B→
←GNG7
←SLC39A3
THOP1→
ZNF556→
ADAT3→
←BTBD2
←MOB3A
←PLEKHJ1
AC005258.1→
←TIMM13
←DIRAS1
←SGTA
ZNF554→
←AP3D1
SF3A2→
SPPL2B→
←LMNB2
←AC006538.2
ZNF555→
AMH→
OAZ1→

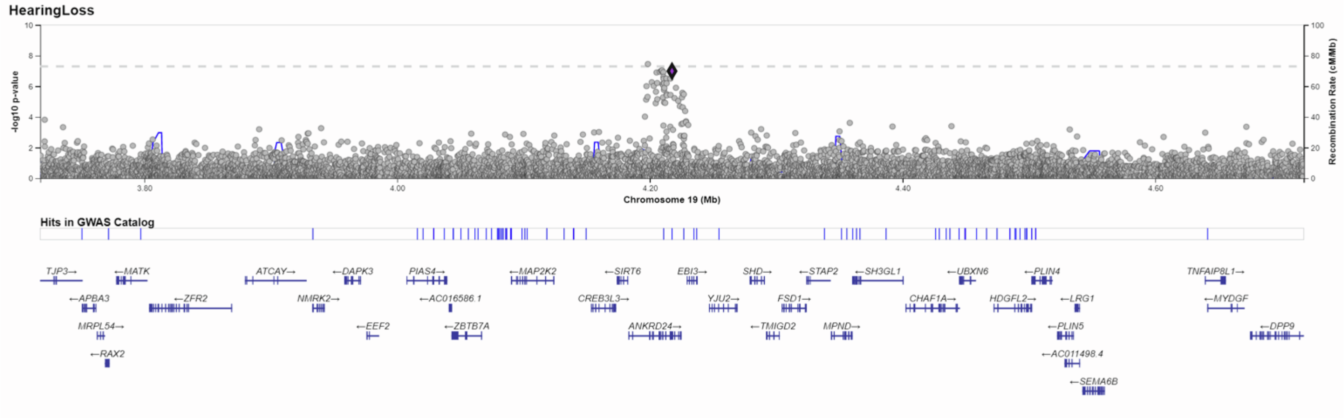
HearingLoss
-log10 p-value
Recombination Rate (cM/Mb)
Chromosome 19 (Mb)
Hits in GWAS Catalog
TJP3→
←MATK
ATCAY→
←DAPK3
PIAS4→
←MAP2K2
←SIRT6
EBI3→
SHD→
←STAP2
←SH3GL1
←UBXN6
←PLIN4
TNFAIP8L1→
←APBA3
←ZFR2
NMRK2→
←AC016586.1
CREB3L3→
YJU2→
FSD1→
CHAF1A→
HDGFL2→
←LRG1
←MYDGF
MRPL54→
←EEF2
←ZBTB7A
ANKRD24→
←TMIGD2
MPND→
←PLIN5
←DPP9
←RAX2
←AC011498.4
←SEMA6B

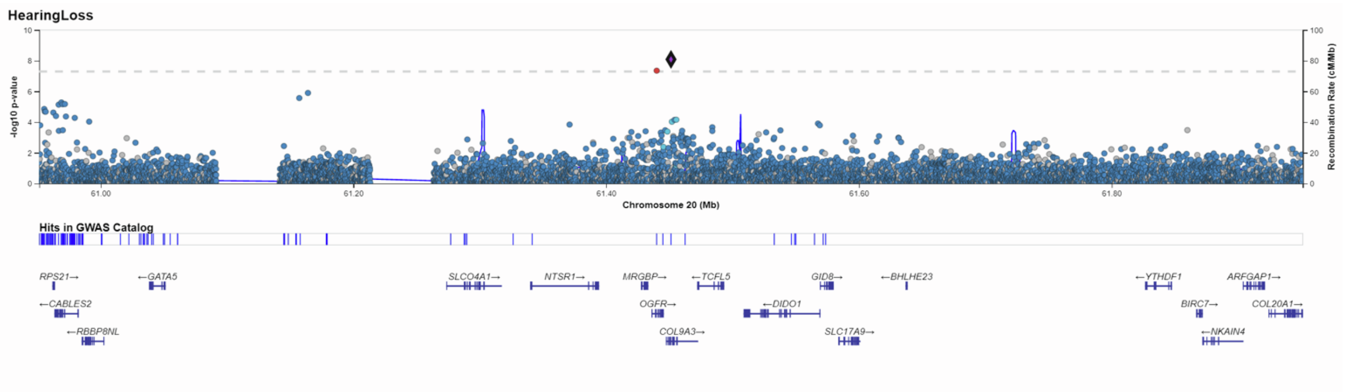
HearingLoss
-log10 p-value
Recombination Rate (cM/Mb)
Chromosome 20 (Mb)
Hits in GWAS Catalog
RPS21→
←GATA5
←CABLES2
←RBBP8NL
SLCO4A1→
NTSR1→
MRGBP→
←TCFL5
OGFR→
COL9A3→
←DIDO1
GID8→
SLC17A9→
←BHLHE23
←YTHDF1
ARFGAP1→
BIRC7→
COL20A1→
←NKAIN4

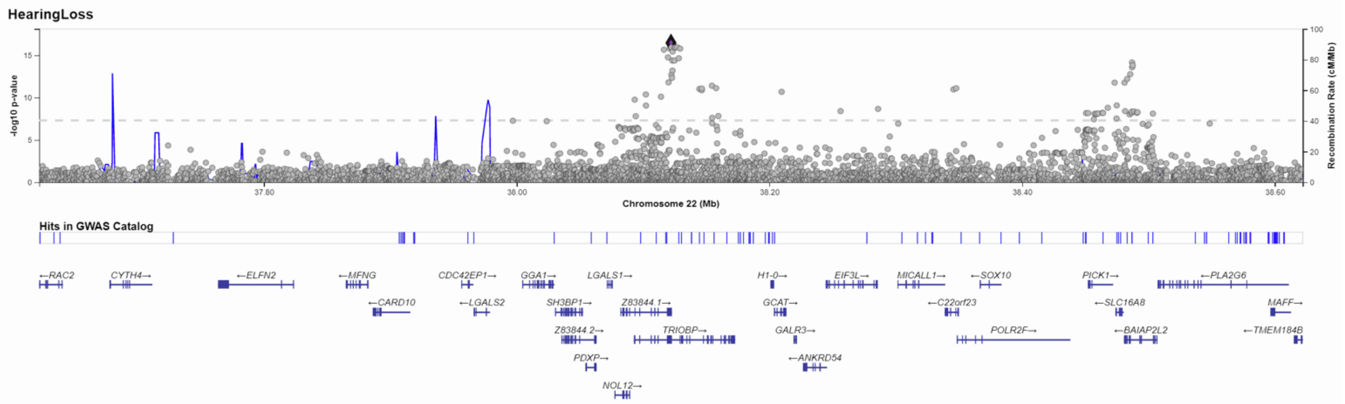
HearingLoss
-log10 p-value
Recombination Rate (cM/Mb)
Chromosome 22 (Mb)
Hits in GWAS Catalog
←RAC2
CYTH4→
←ELFN2
←MFNG
←CARD10
CDC42EP1→
←LGALS2
GGA1→
SH3BP1→
Z83844.2→
PDXP→
NOL12→
LGALS1→
Z83844.1→
TRIOBP→
H1-0→
GCAT→
GALR3→
←ANKRD54
EIF3L→
MICALL1→
←C22orf23
←SOX10
POLR2F→
PICK1→
←SLC16A8
←BAIAP2L2
←PLA2G6
MAFF→
←TMEM184B

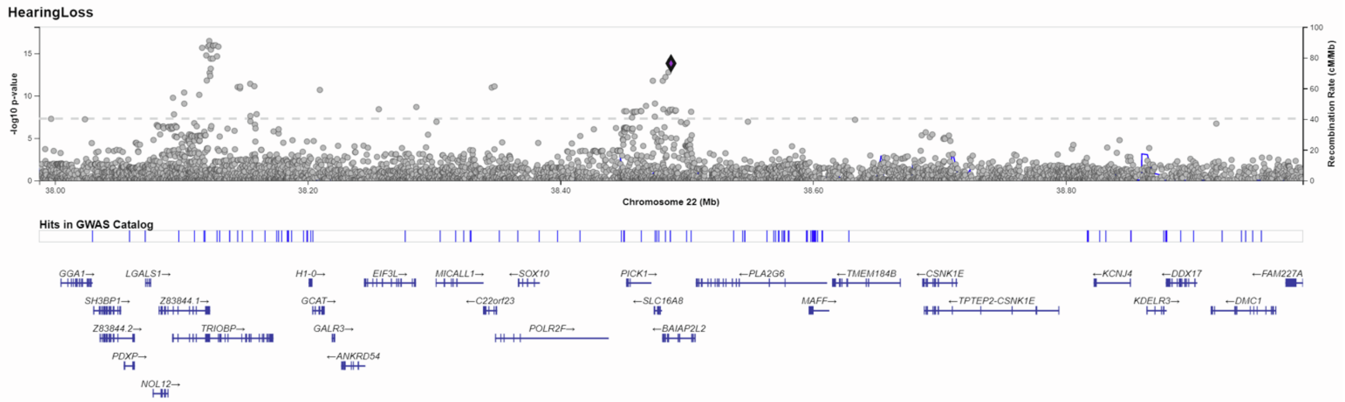
HearingLoss
-log10 p-value
Recombination Rate (cM/Mb)
Chromosome 22 (Mb)
Hits in GWAS Catalog
GGA1→
SH3BP1→
Z83844.2→
PDXP→
NOL12→
LGALS1→
Z83844.1→
TRIOBP→
H1-0→
GCAT→
GALR3→
←ANKRD54
EIF3L→
MICALL1→
←C22orf23
←SOX10
POLR2F→
PICK1→
←SLC16A8
←BAIAP2L2
←PLA2G6
MAFF→
←TMEM184B
←CSNK1E
←TPTEP2-CSNK1E
←KCNJ4
KDELR3→
←DDX17
←DMC1
←FAM227A

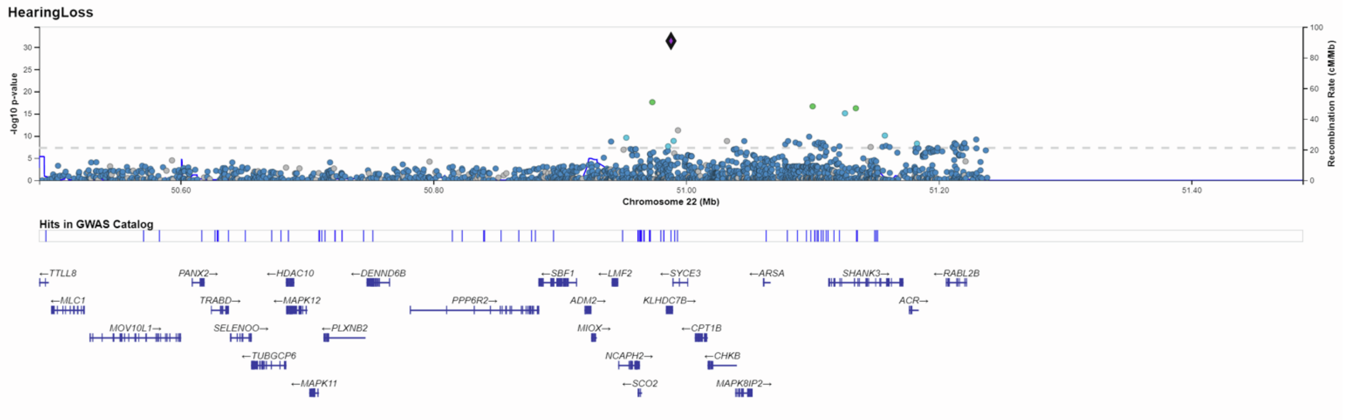
HearingLoss
-log10 p-value
Recombination Rate (cM/Mb)
Chromosome 22 (Mb)
Hits in GWAS Catalog
←TTLL8
←MLC1
MOV10L1→
PANX2→
TRABD→
SELENOO→
←TUBGCP6
←HDAC10
←MAPK12
←MAPK11
←PLXNB2
←DENND6B
PPP6R2→
←SBF1
ADM2→
MIOX→
←LMF2
NCAPH2→
←SCO2
←SYCE3
KLHDC7B→
←CPT1B
←CHKB
MAPK8IP2→
←ARSA
SHANK3→
←RABL2B
ACR→

Figure S3. Forest plots for each statistically significant and independent SNP

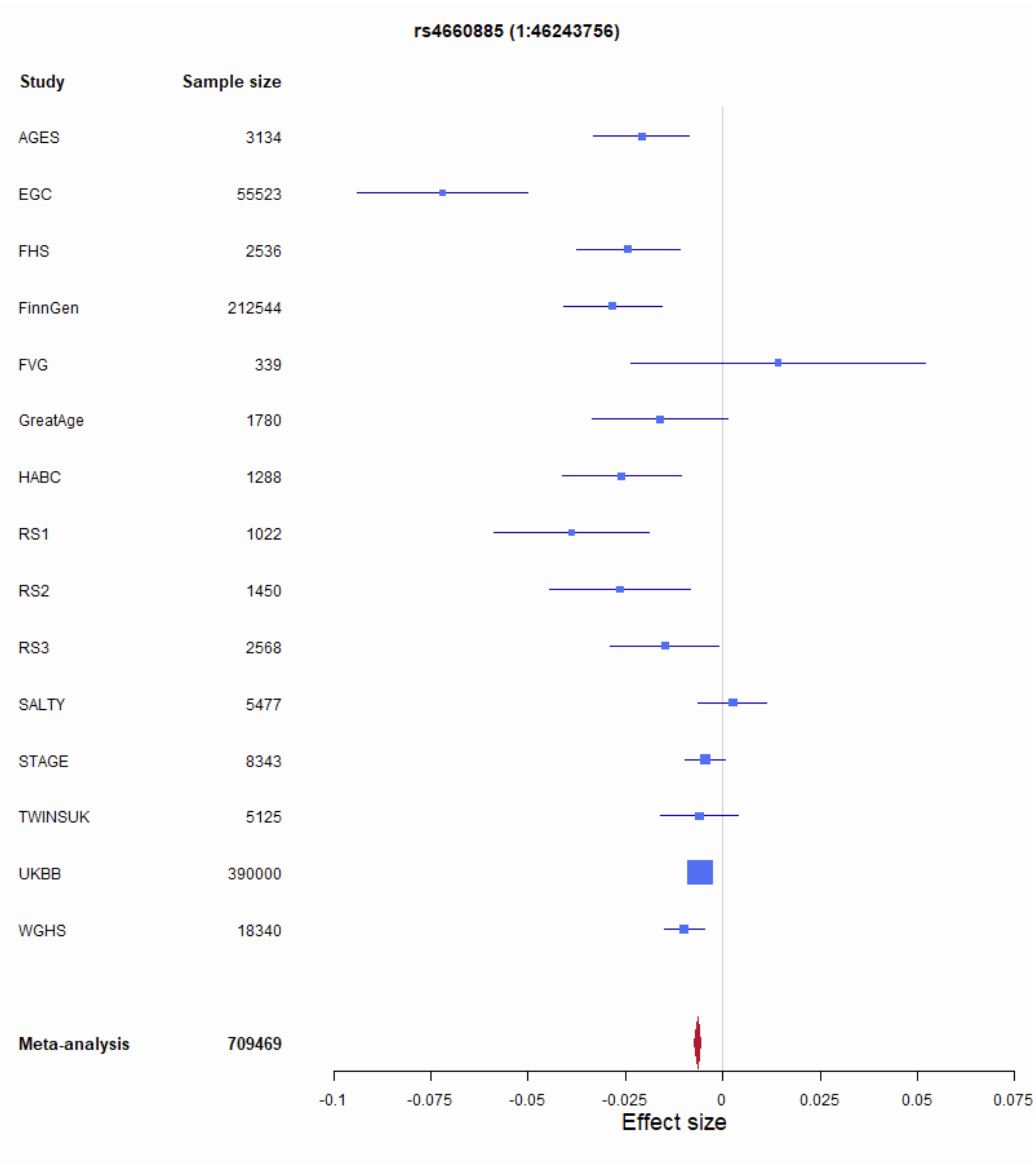

Locus annotation: IPP-[x]-MAST2

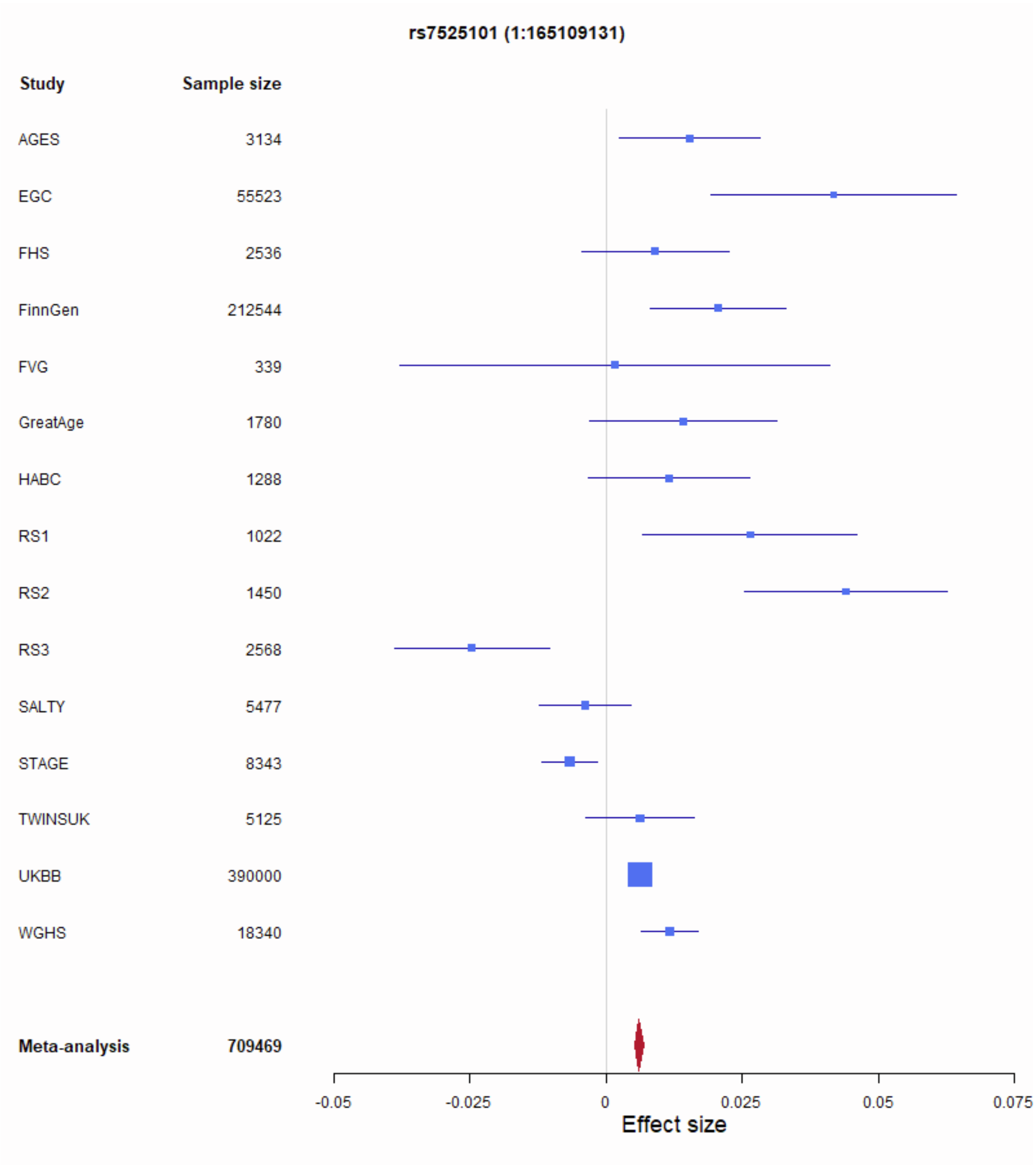

Locus annotation: PBX1---[x]-LMX1A

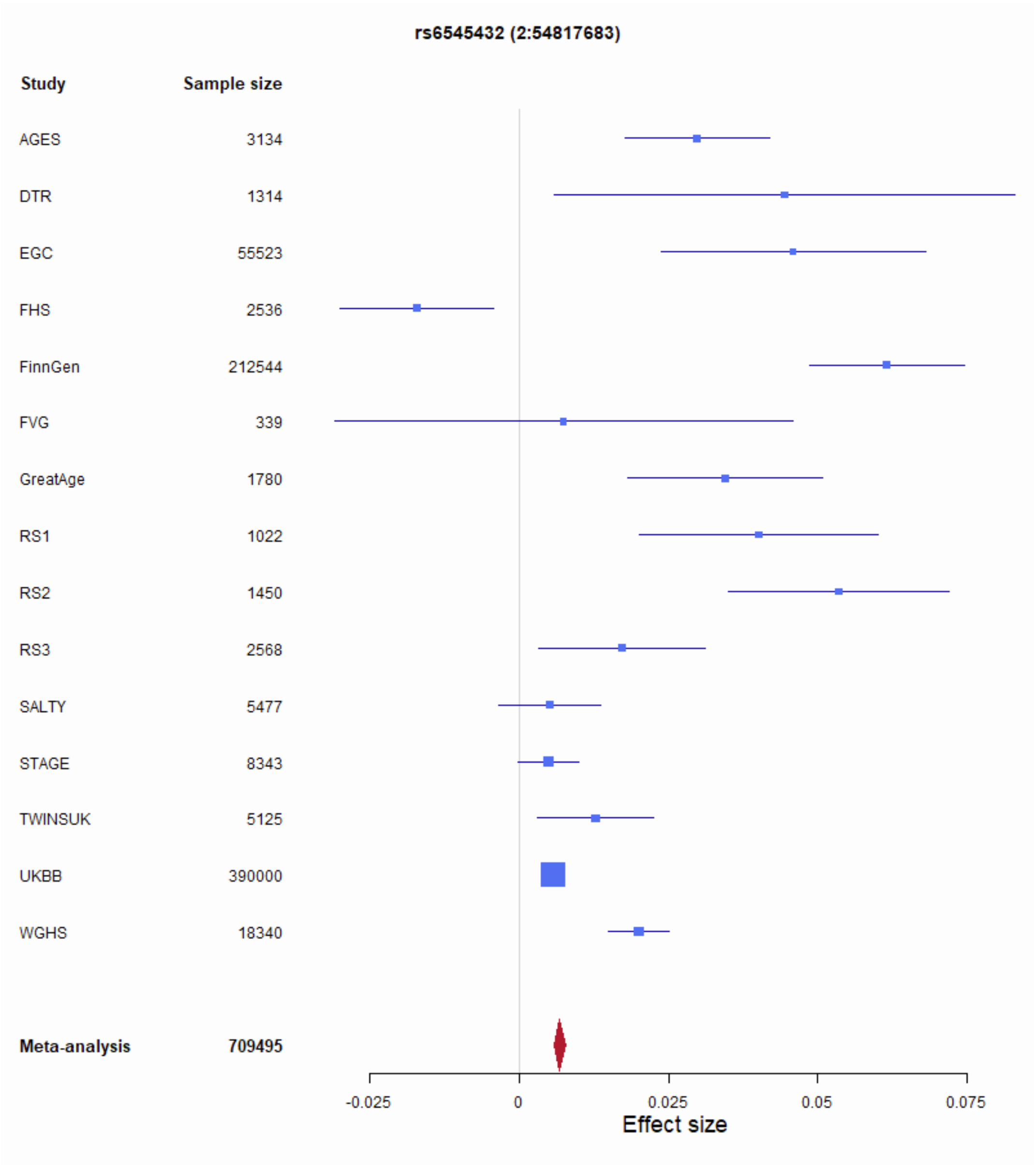

Locus annotation: [SPTBN1] intronic

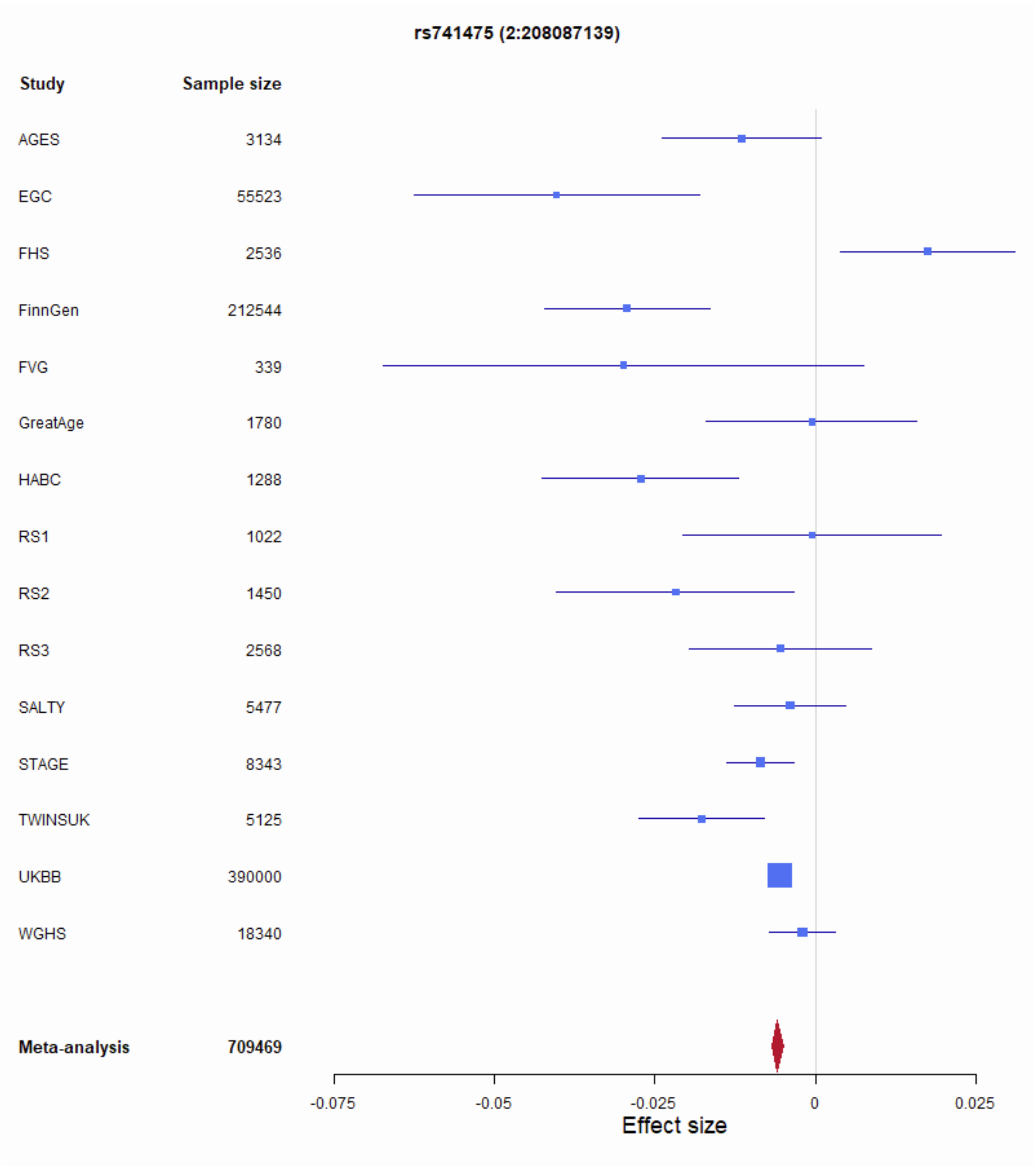

Locus annotation: KLF7-[x]--CREB1

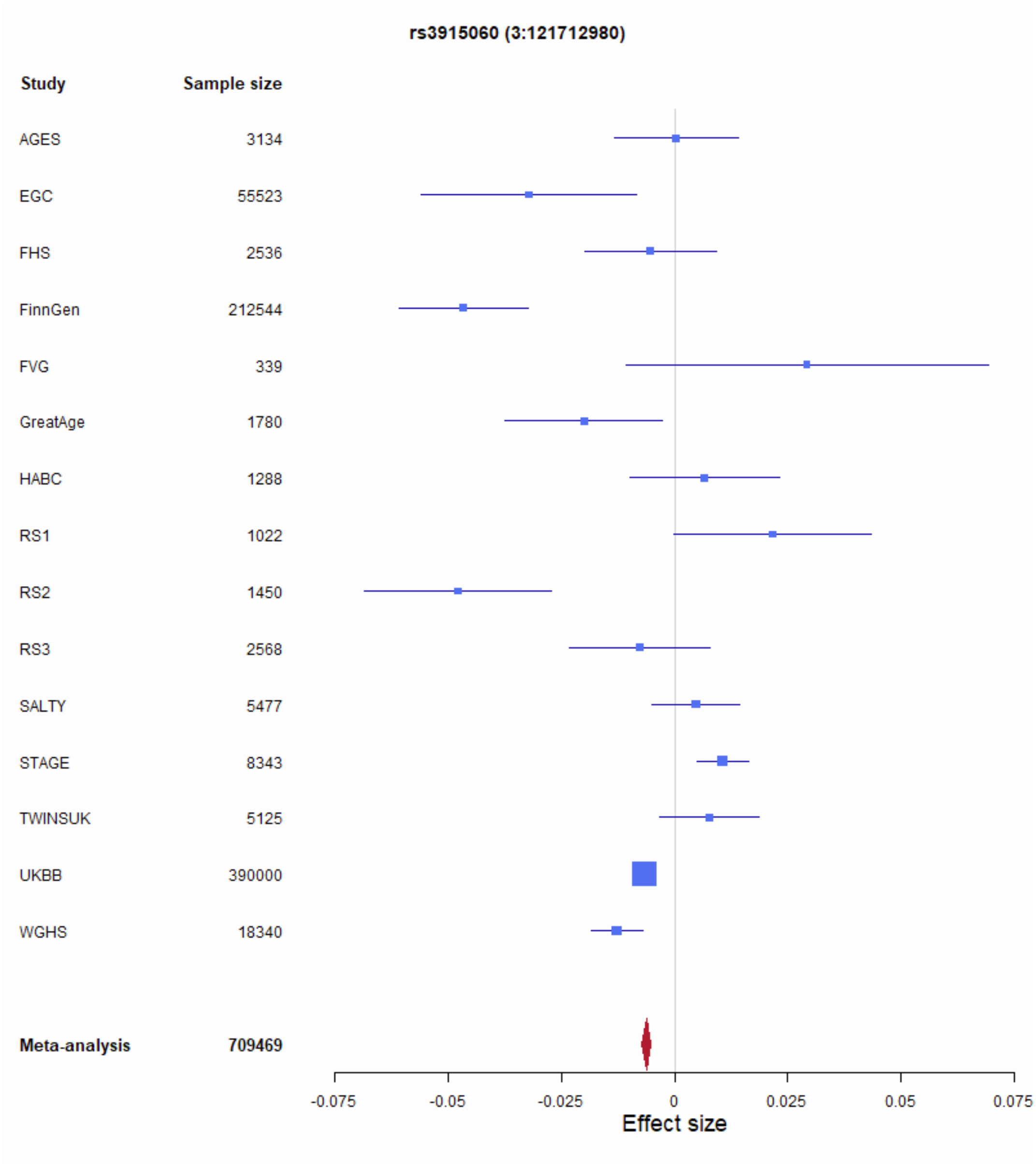

Locus annotation: [ILDR1] intronic

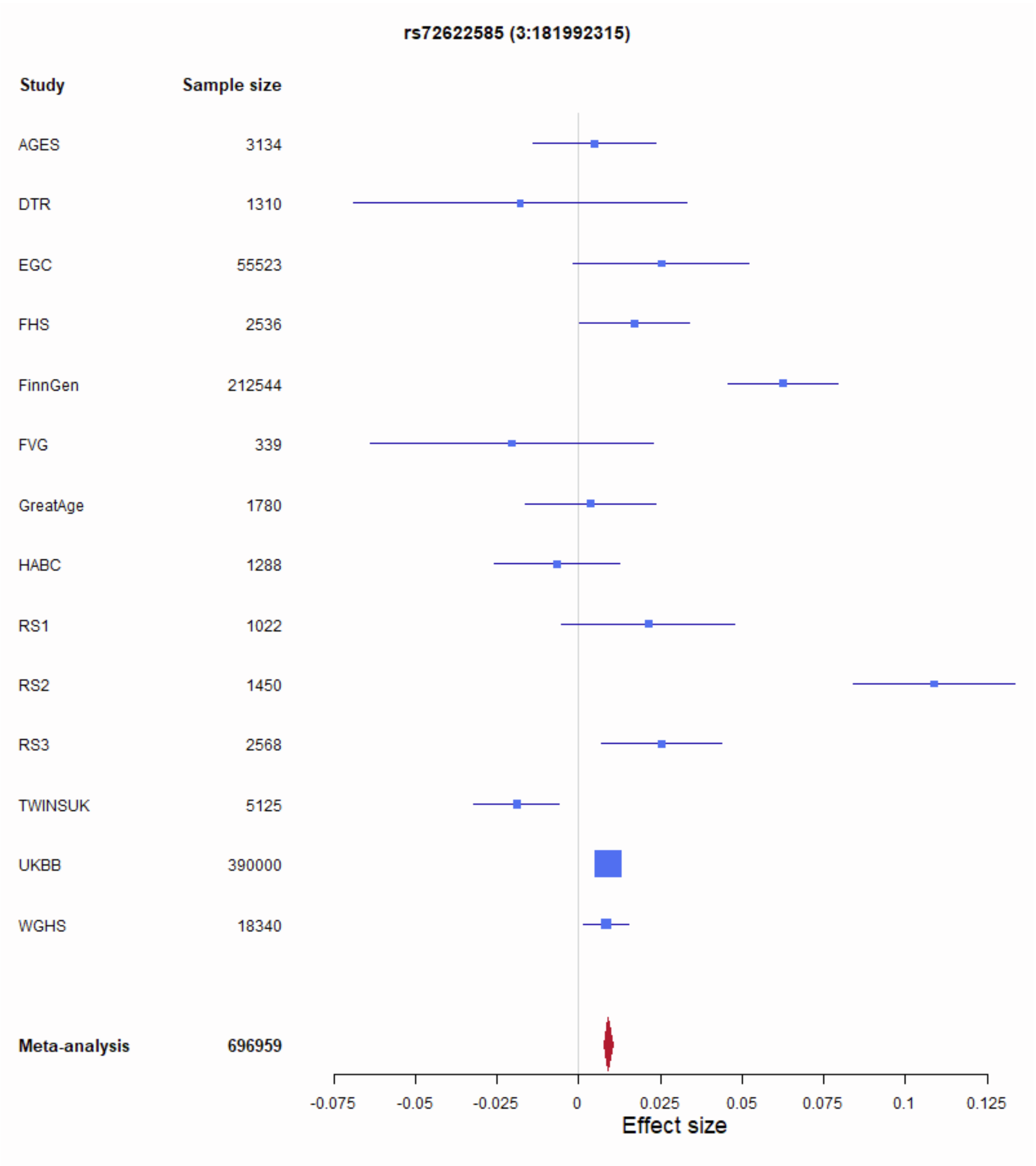

Locus annotation: SOX2---[x]---ATP11B

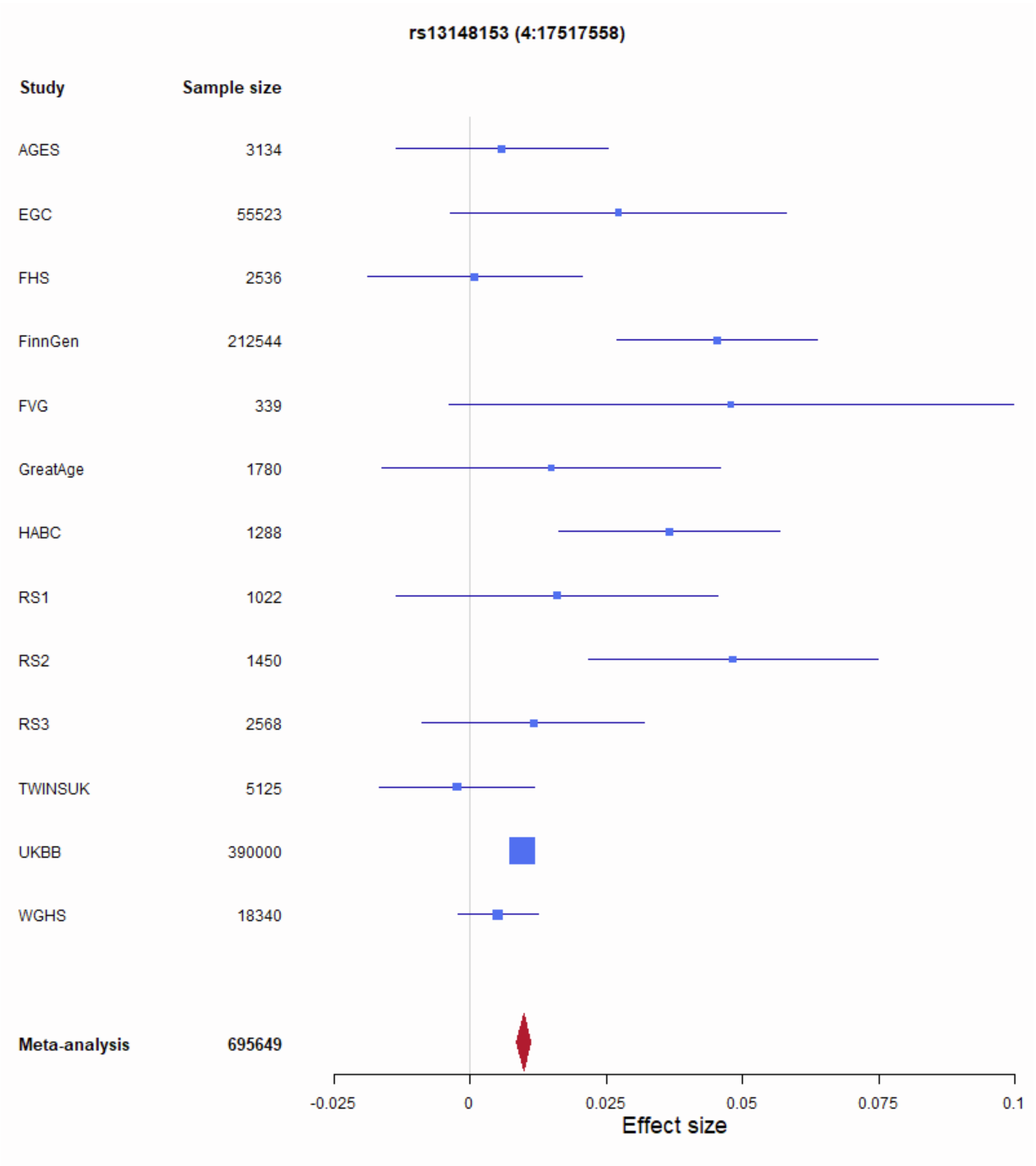

Locus annotation: [CLRN2] intronic

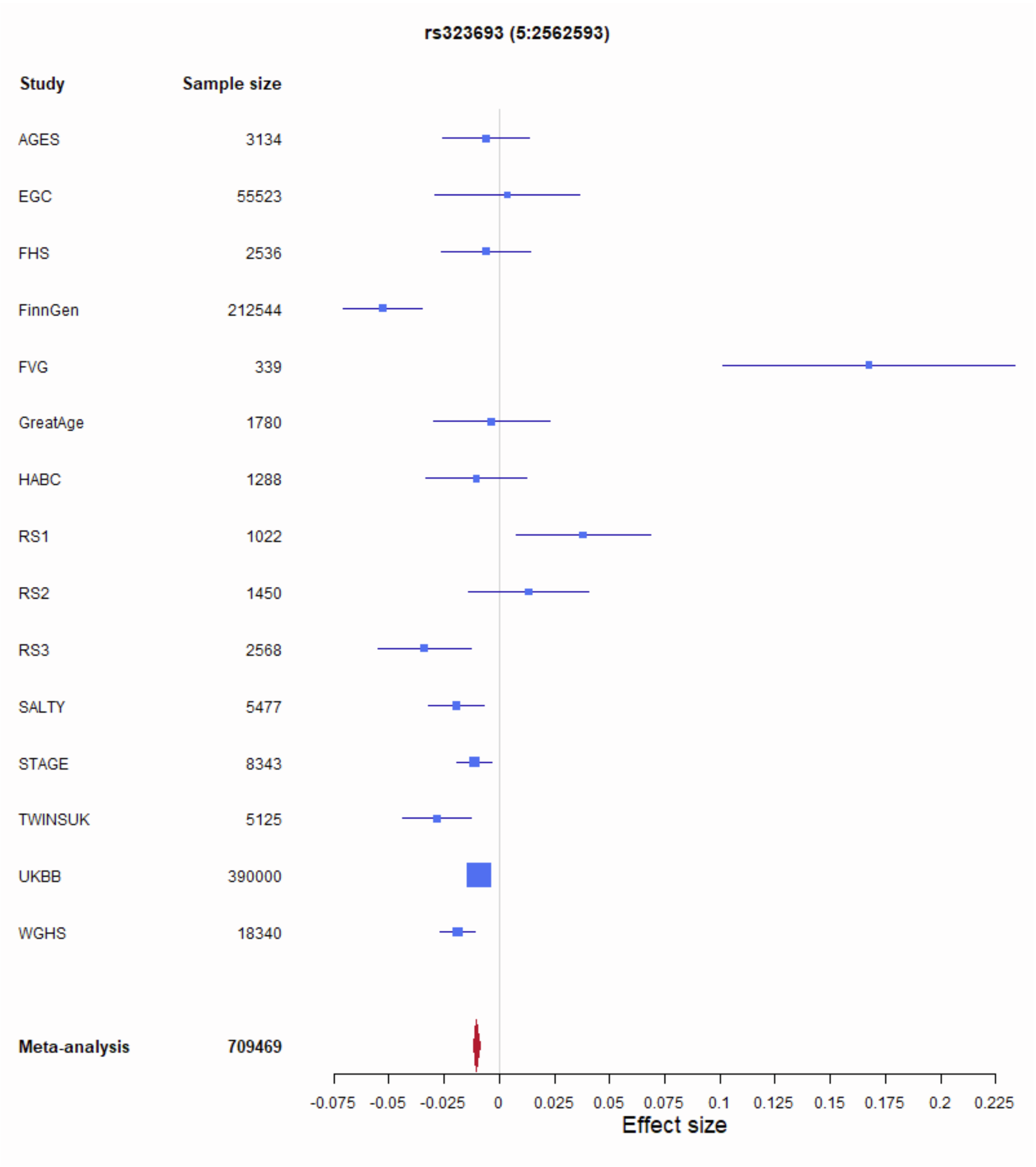

Locus annotation: IRX4---[x]--IRX2

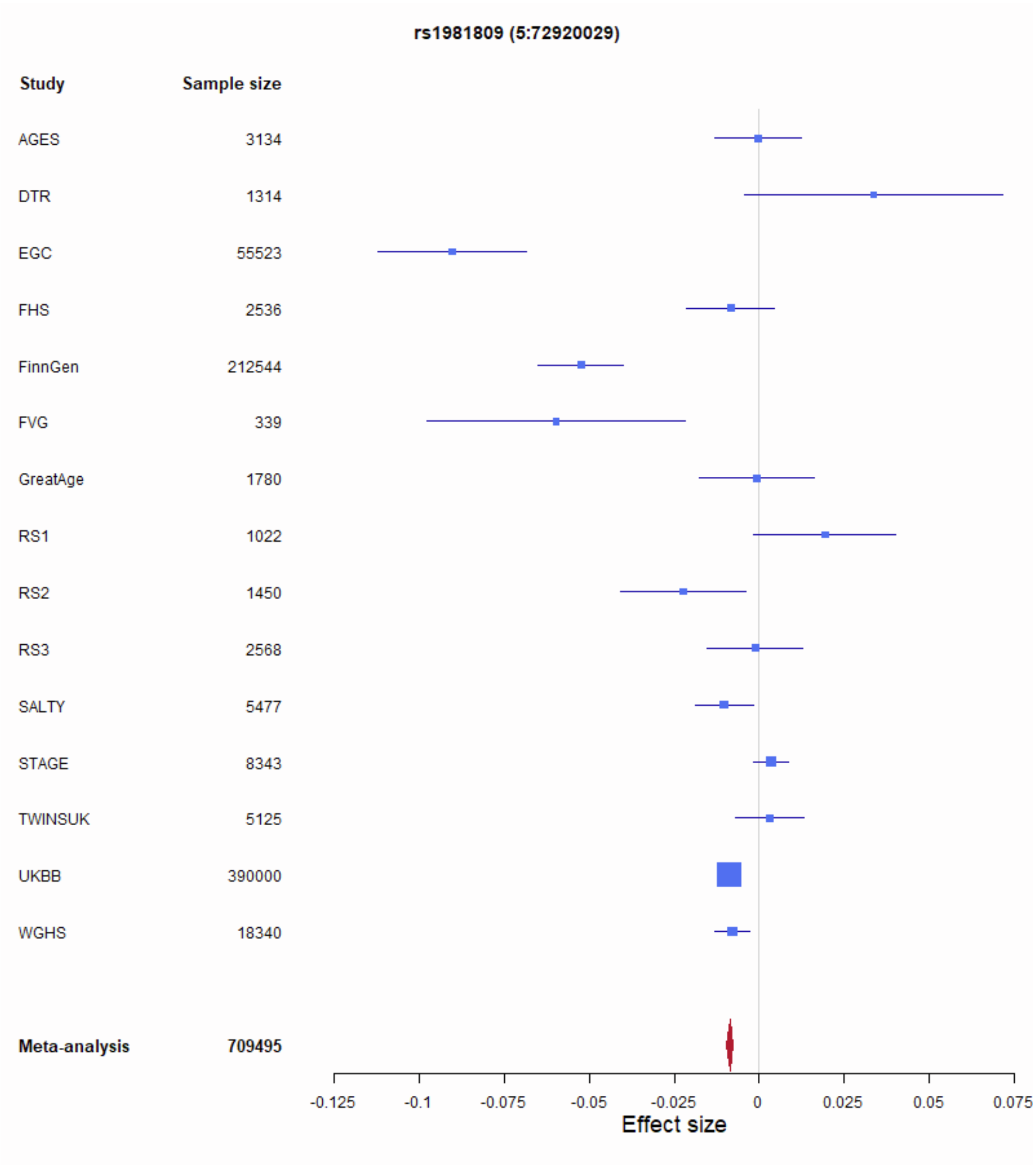

Locus annotation: UTP15-[x]-ARHGEF28

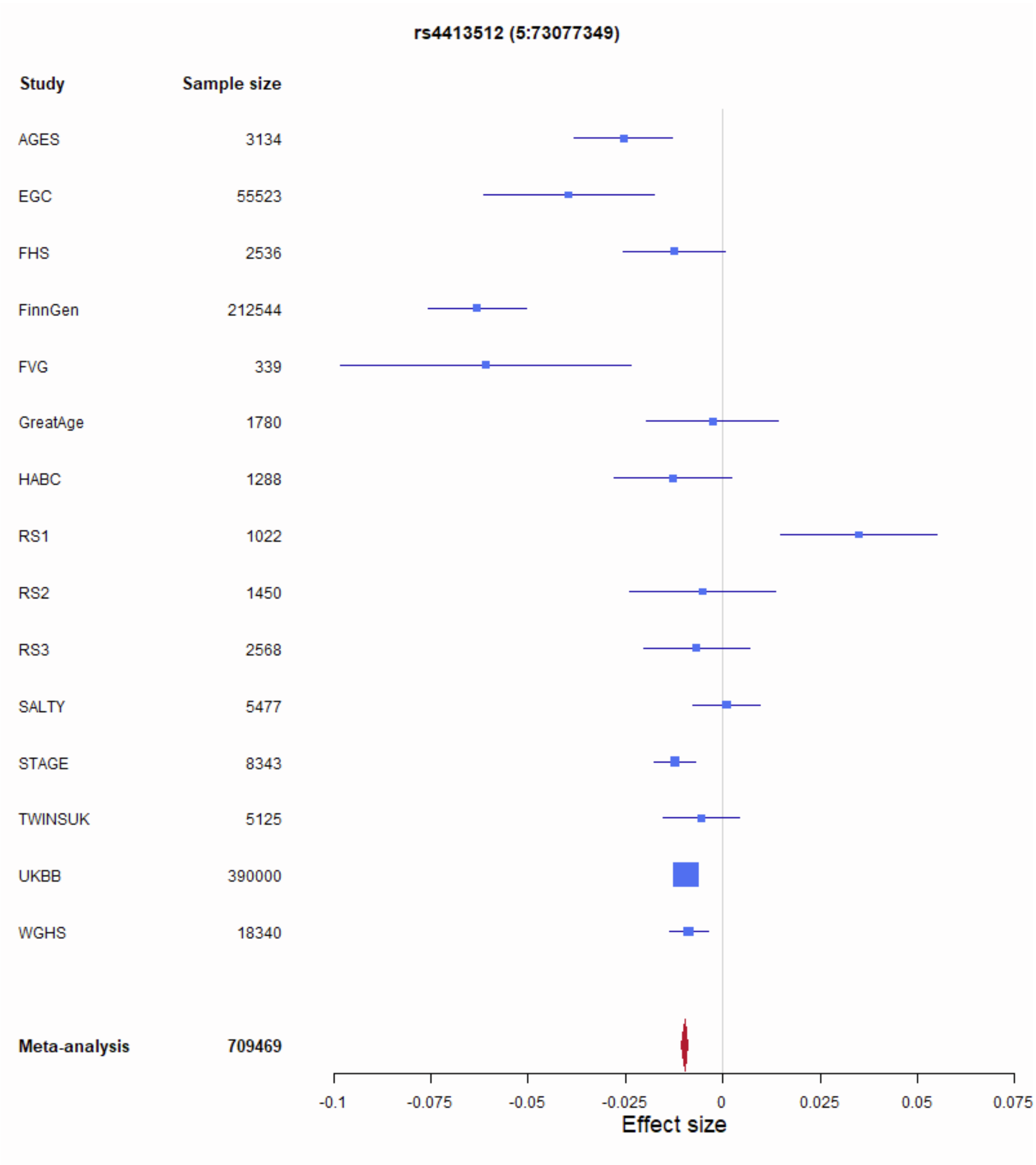

Locus annotation: [ARHGEF28] intronic

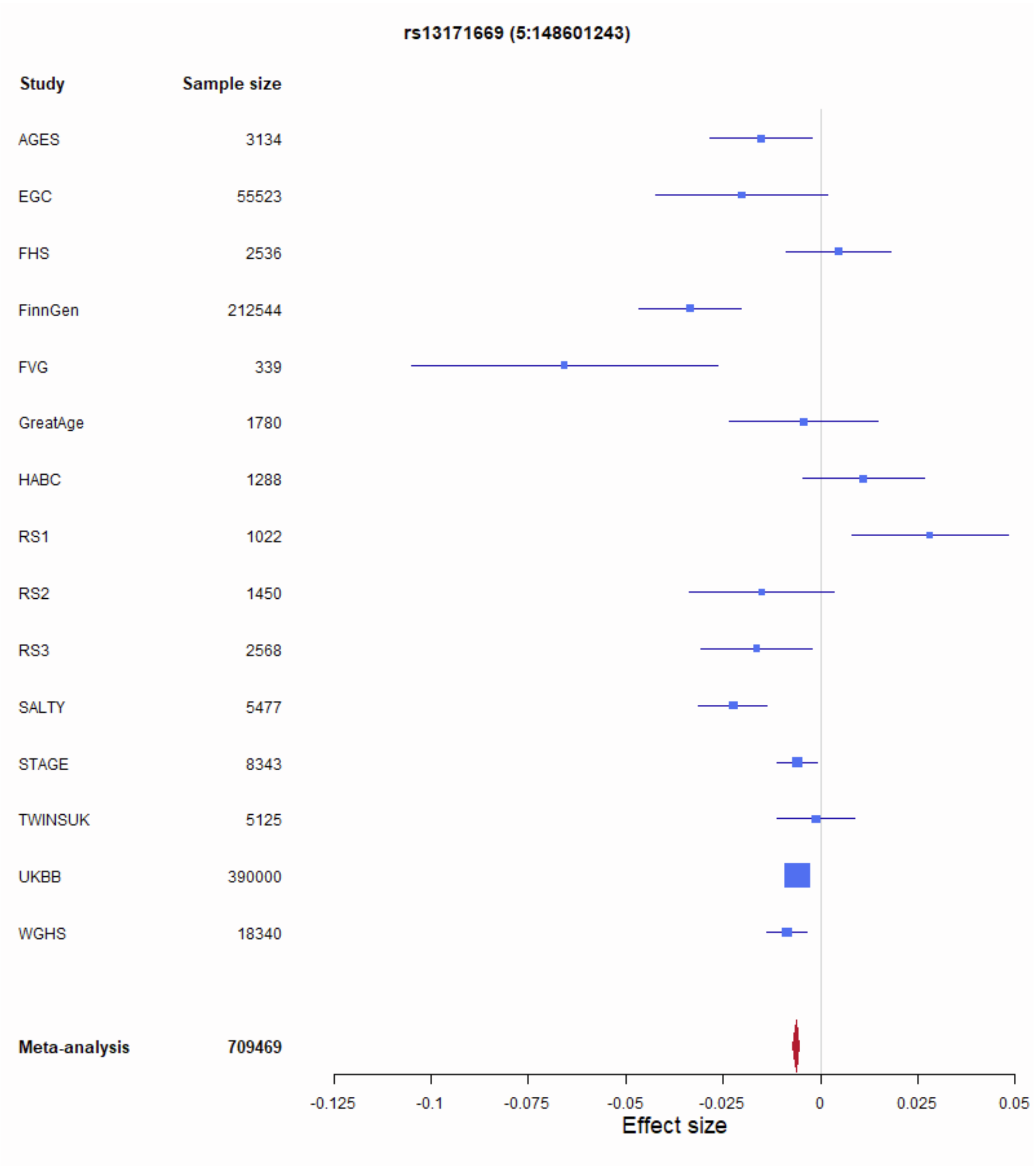

Locus annotation: [ABLIM3] intronic

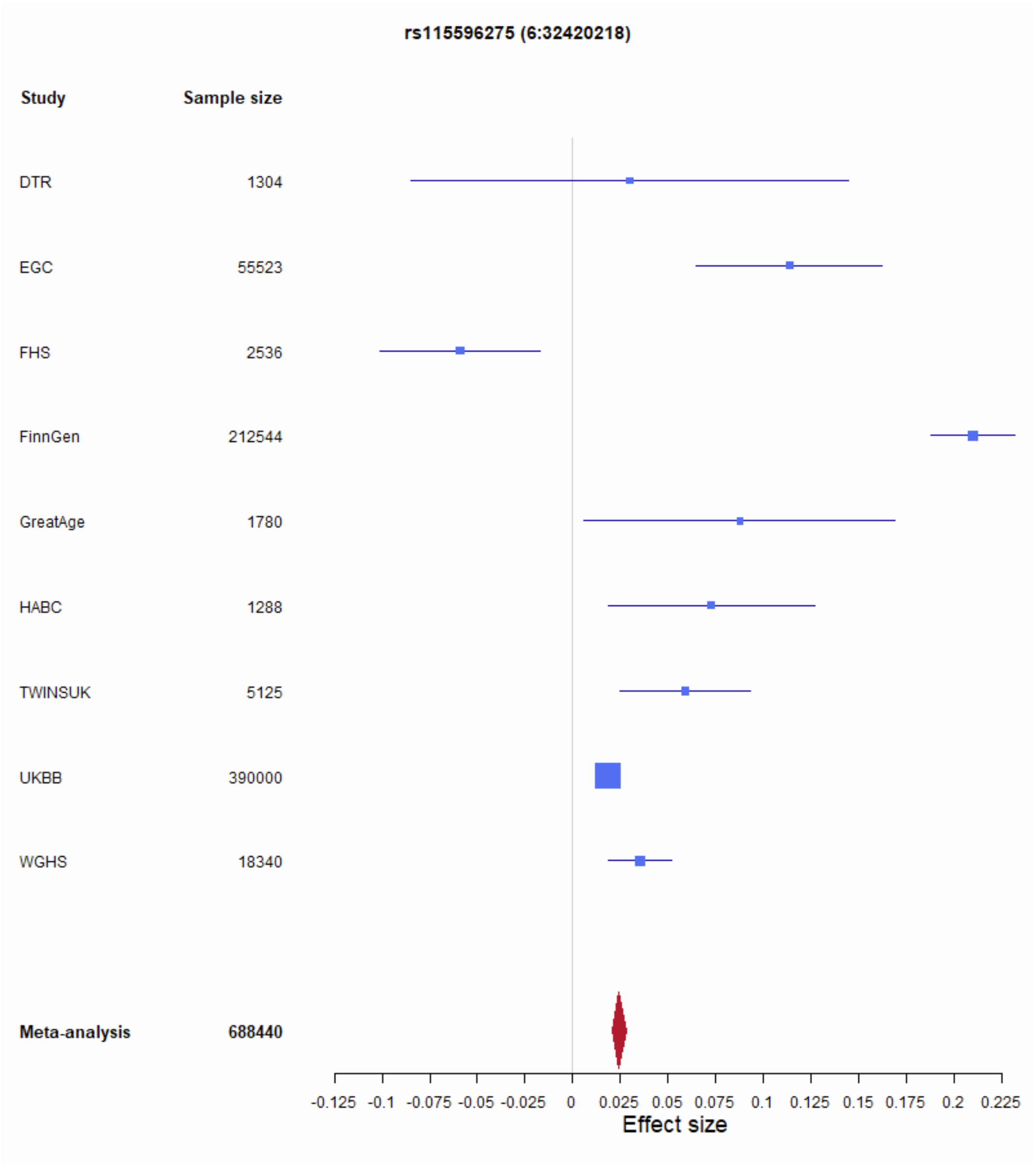

Locus annotation: HLA-DRA-[x]-HLA-DRB5

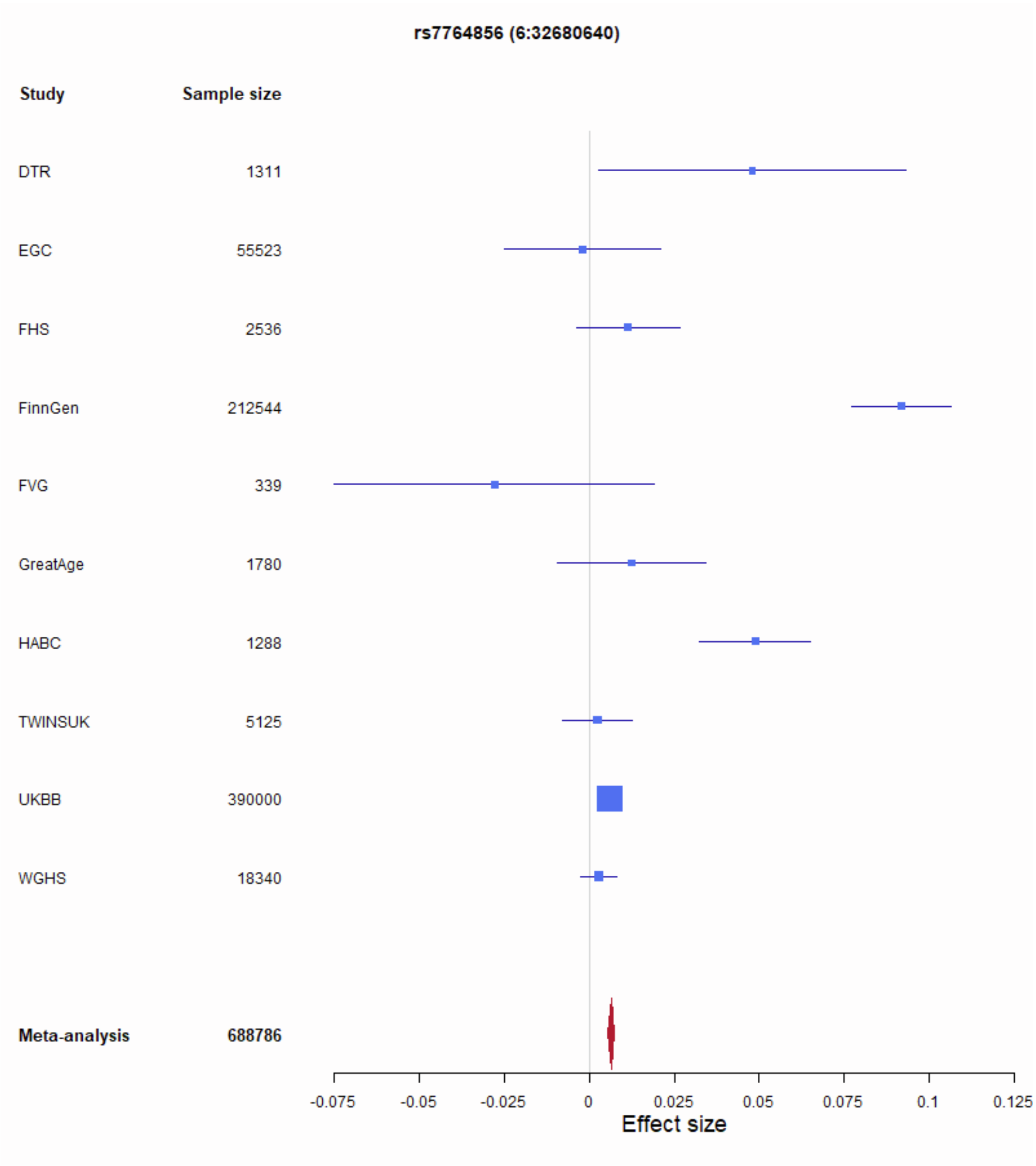

Locus annotation: HLA-DQB1-[x]-HLA-DQA2

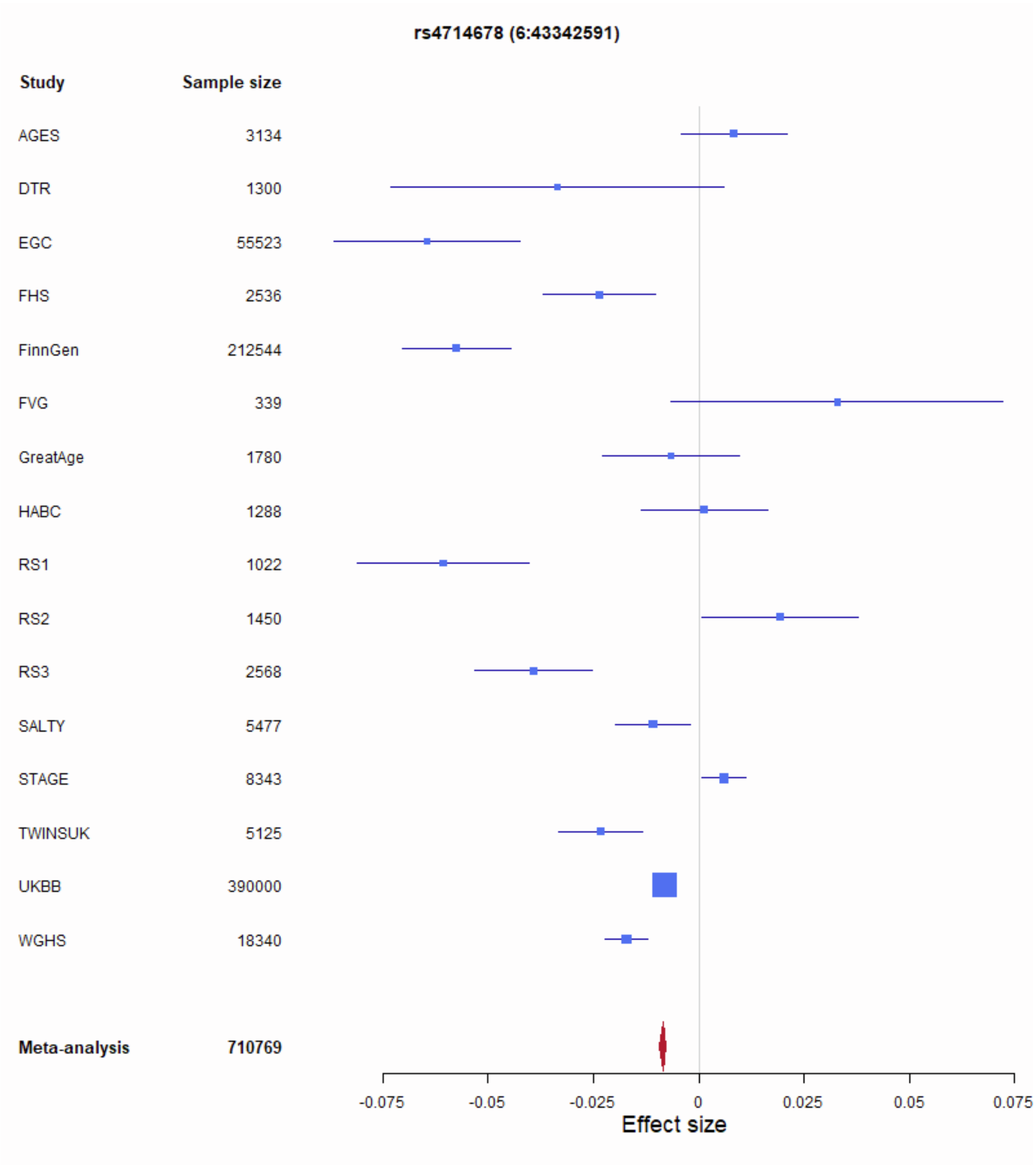

Locus annotation: ZNF318-[x]-ABCC10

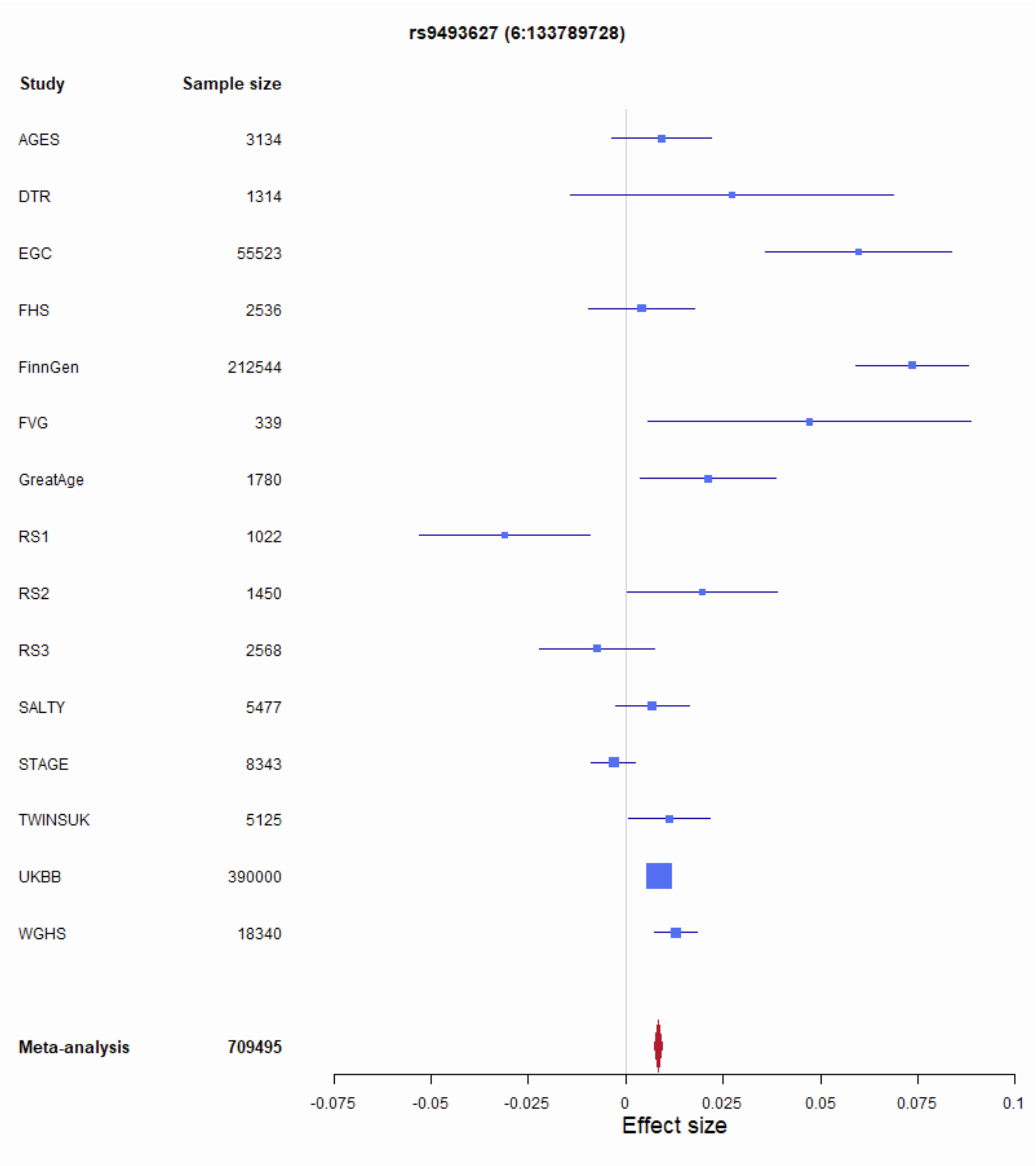

Locus annotation: [EYA4] G>S

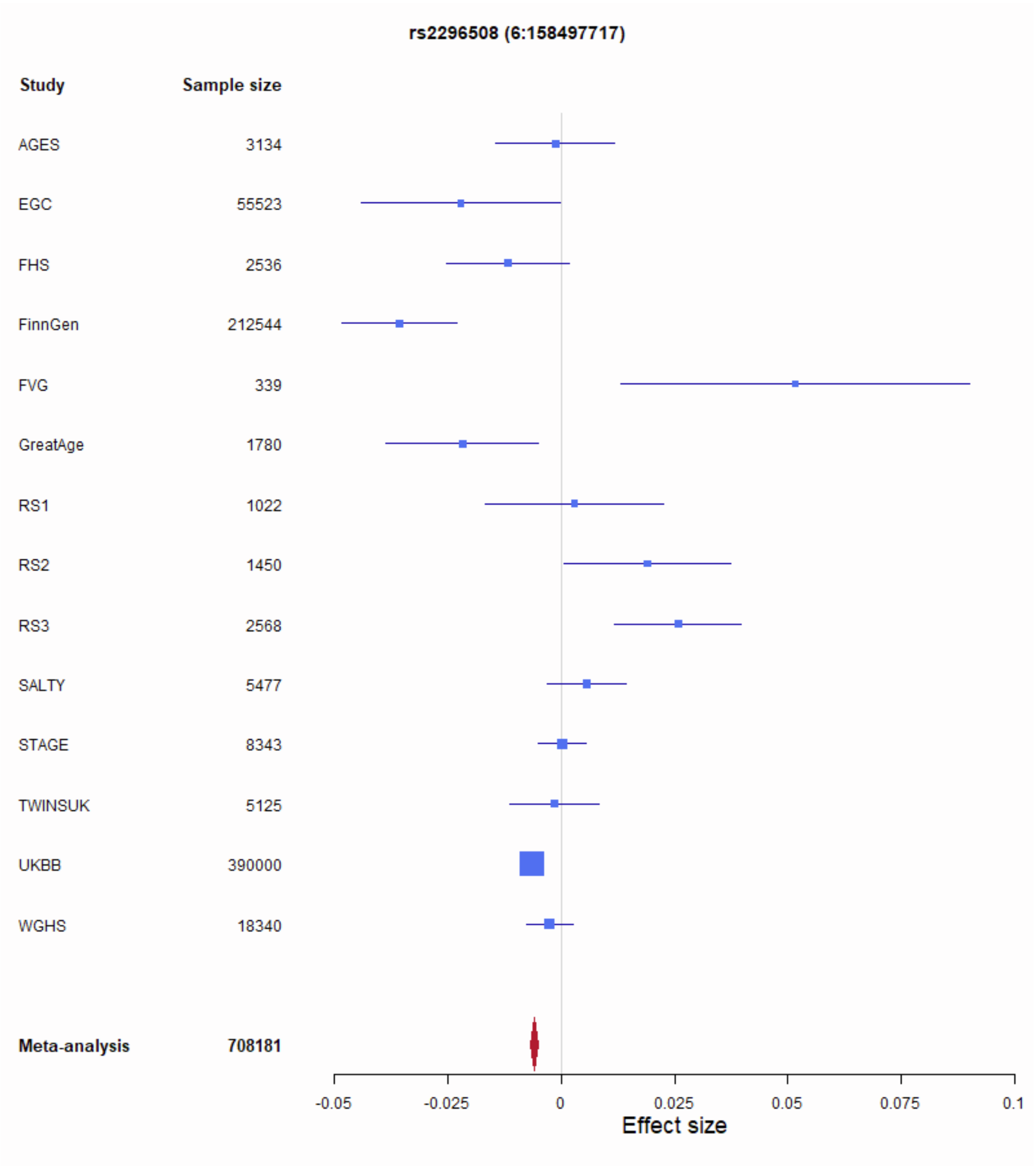

Locus annotation: [SYNJ2] V>V

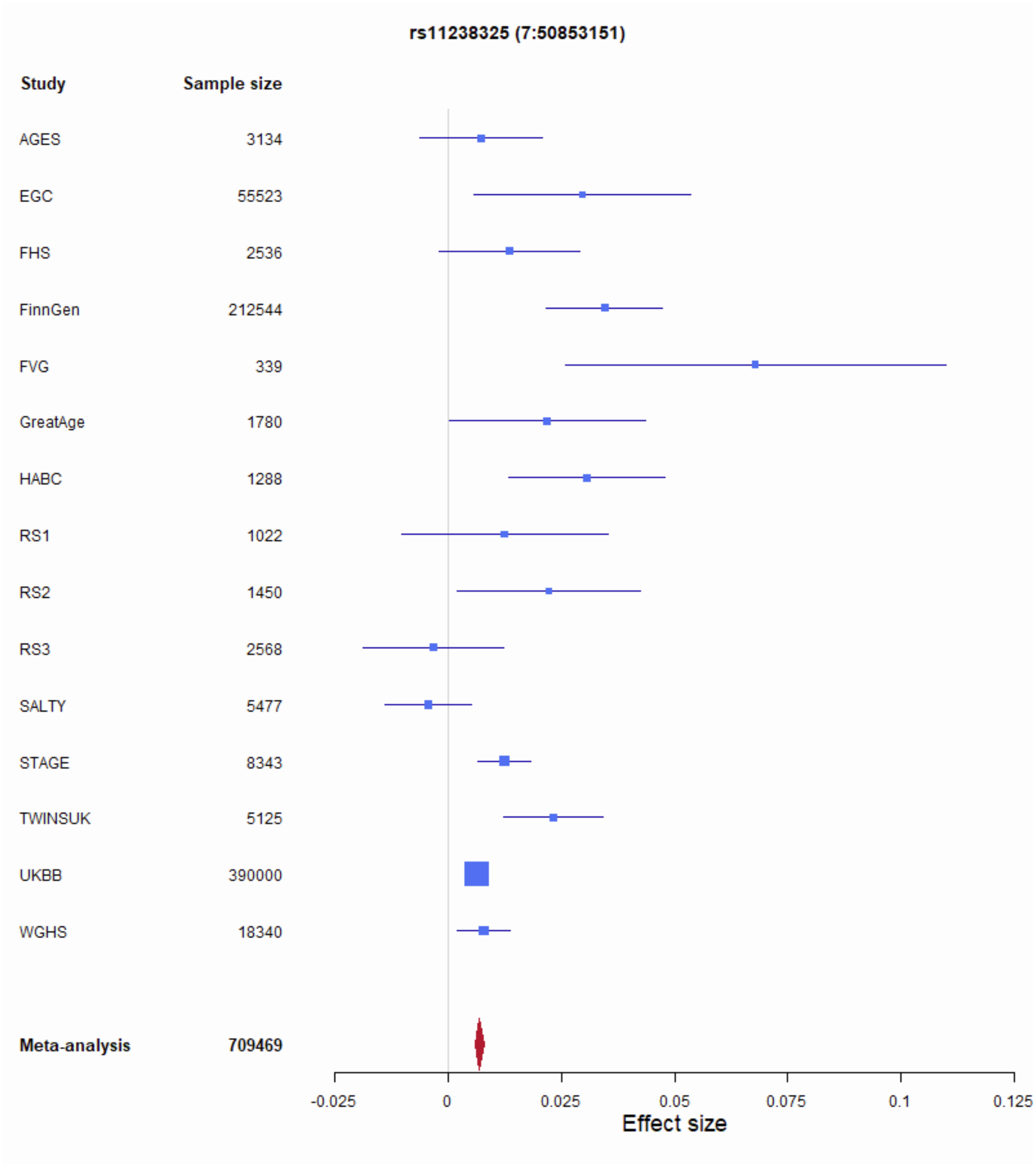

Locus annotation: [GRB10] intronic

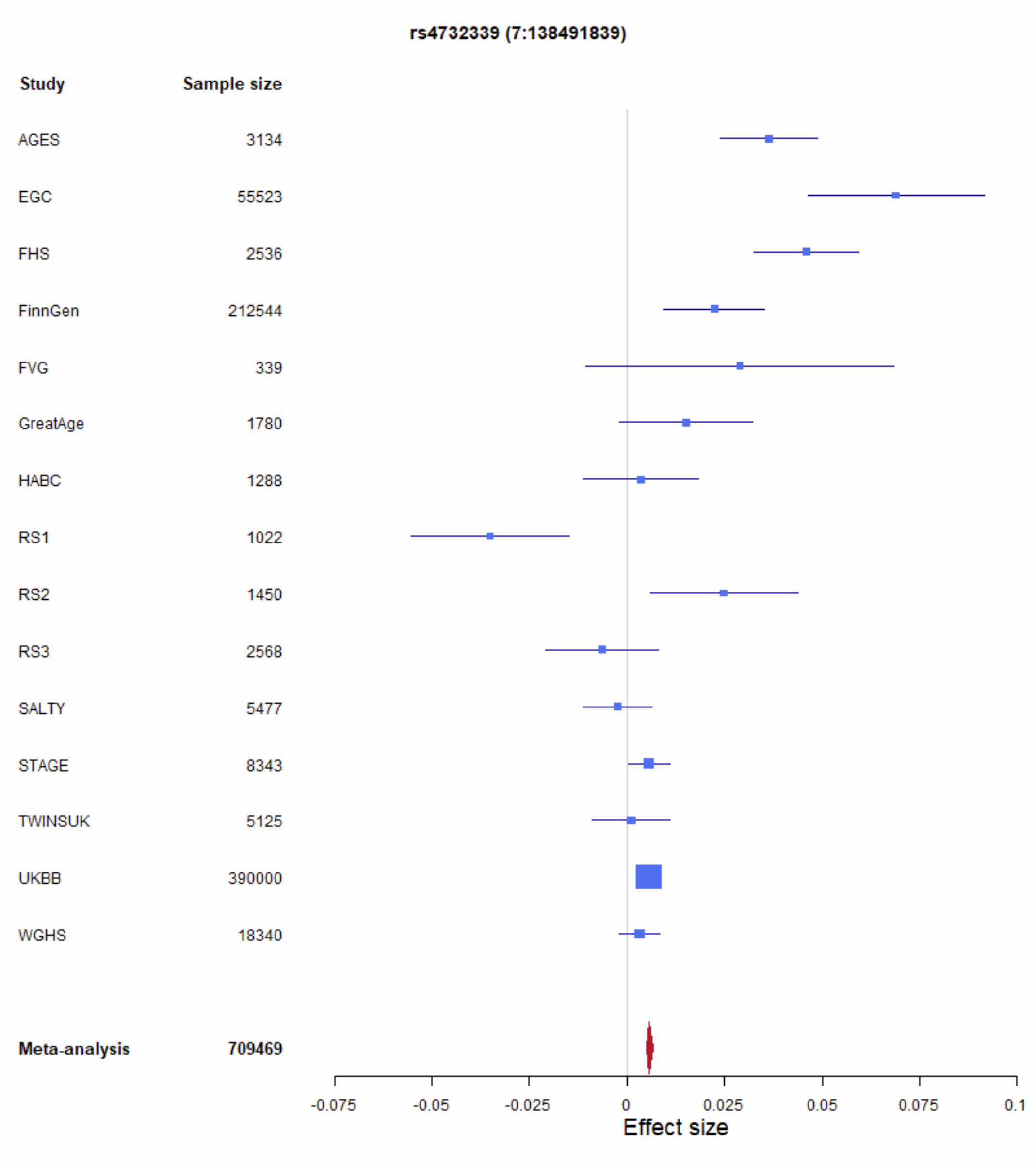

Locus annotation: TMEM213-[x]-KIAA1549

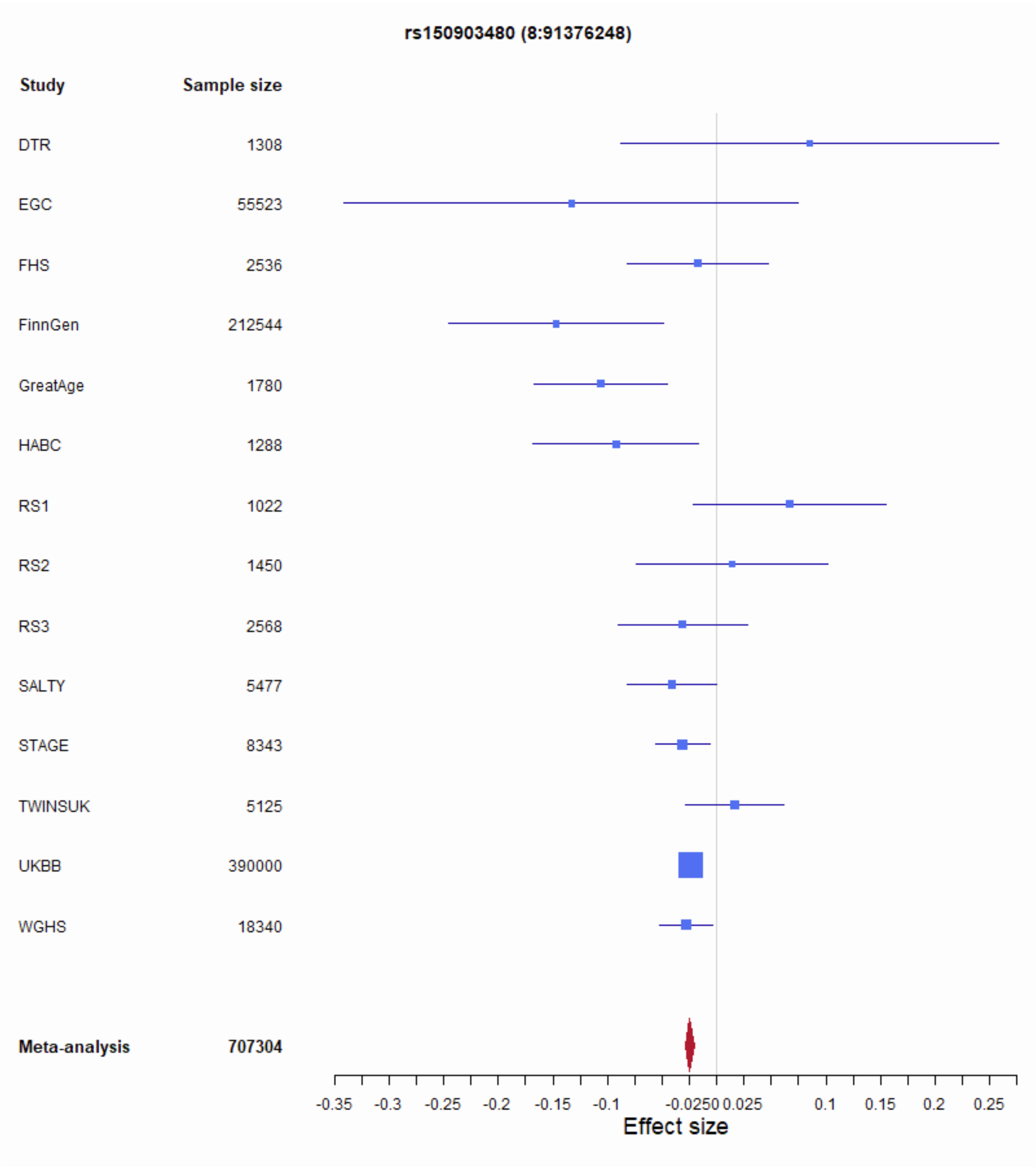

Locus annotation: [LINC00534]

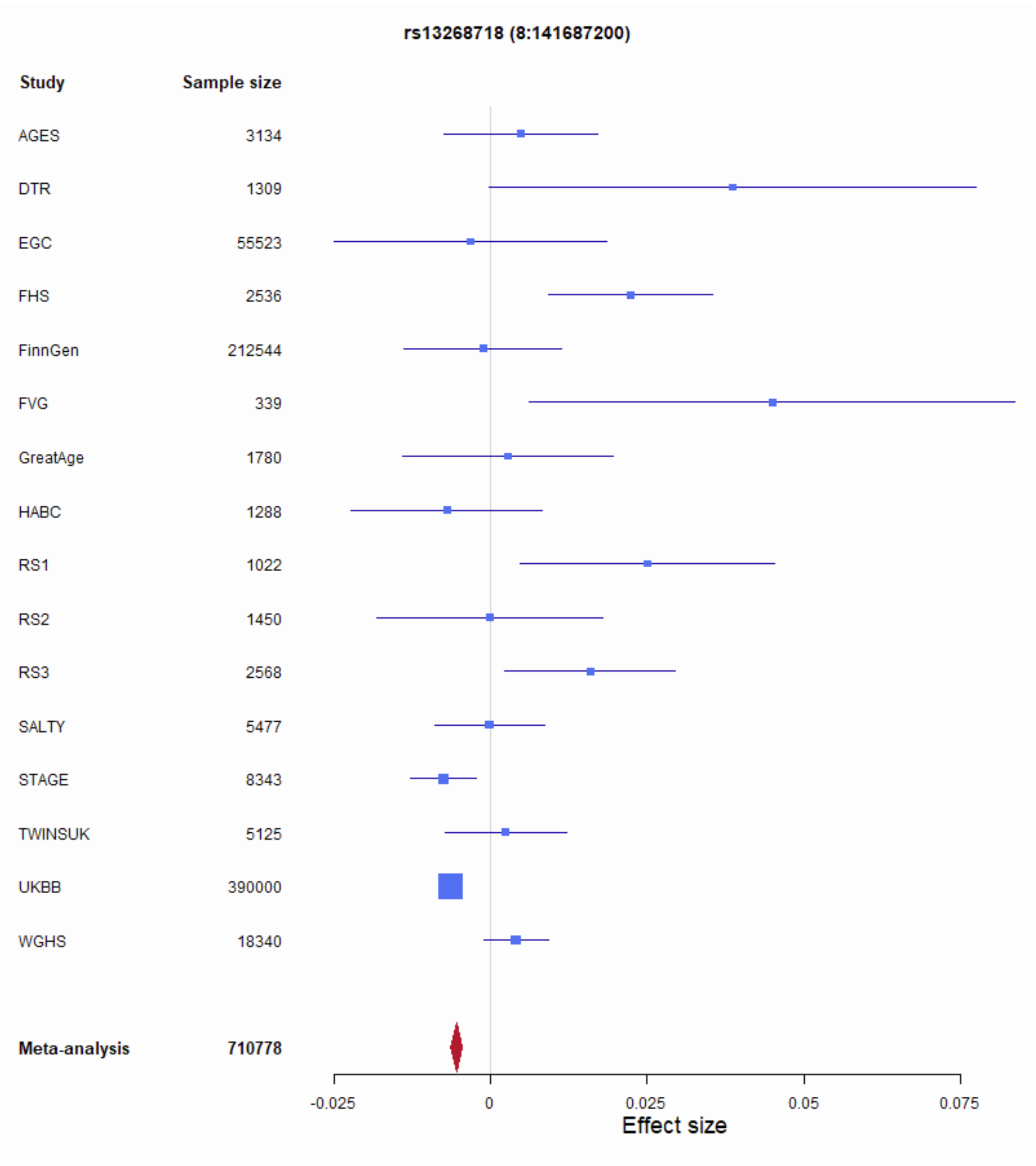

Locus annotation: [PTK2] intronic

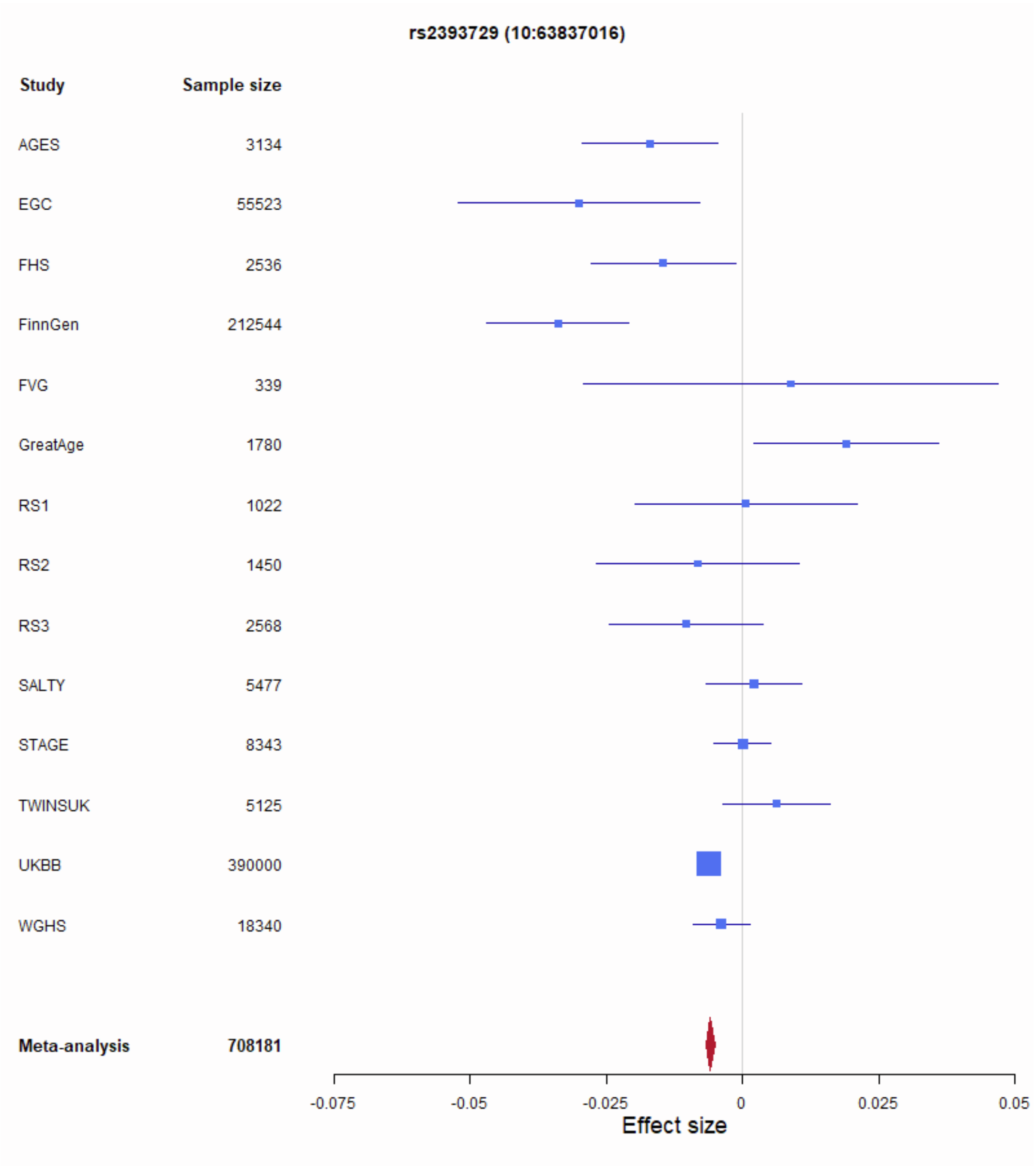

Locus annotation: [ARID5B] intronic

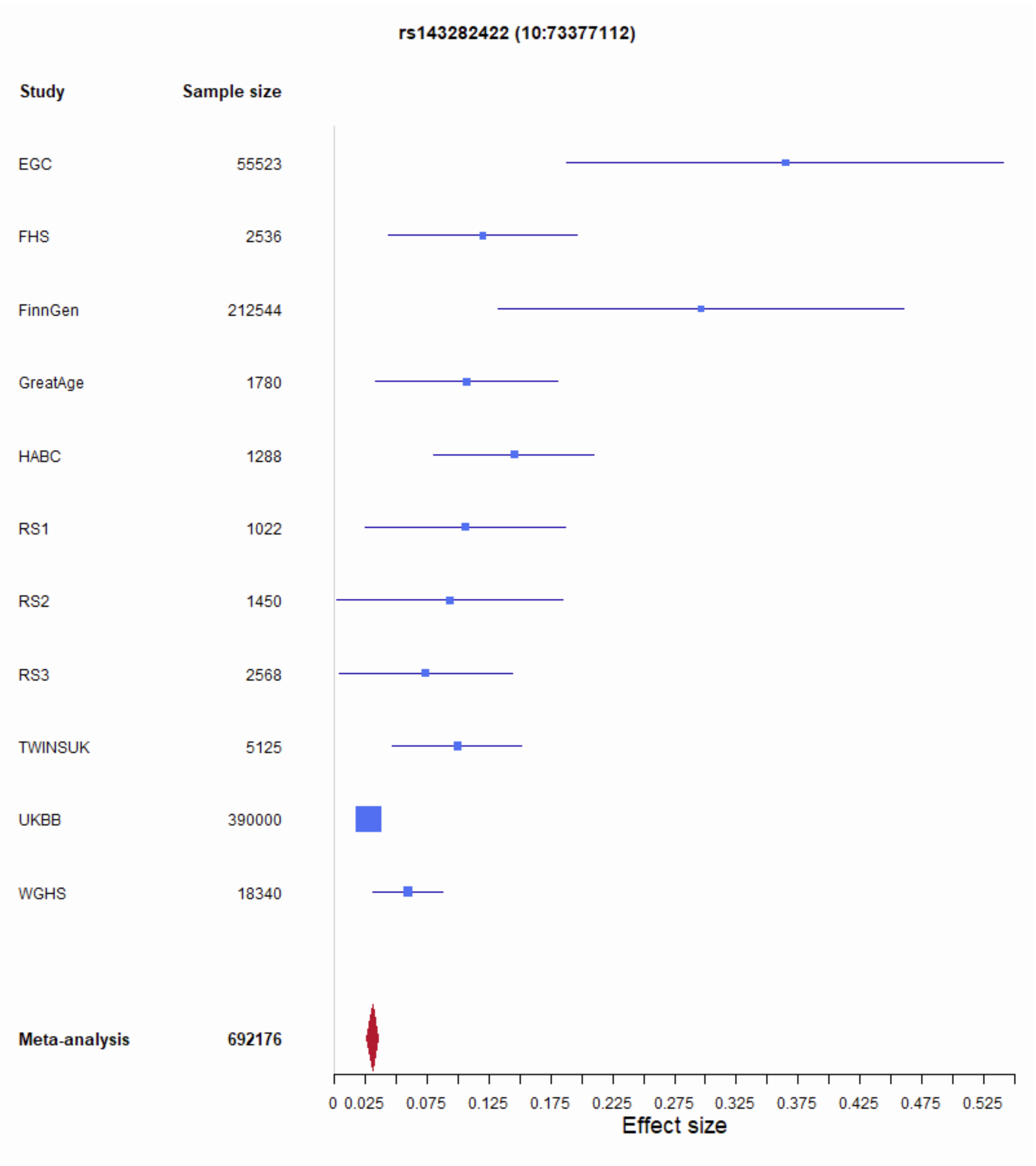

Locus annotation: [CDH23] A>T

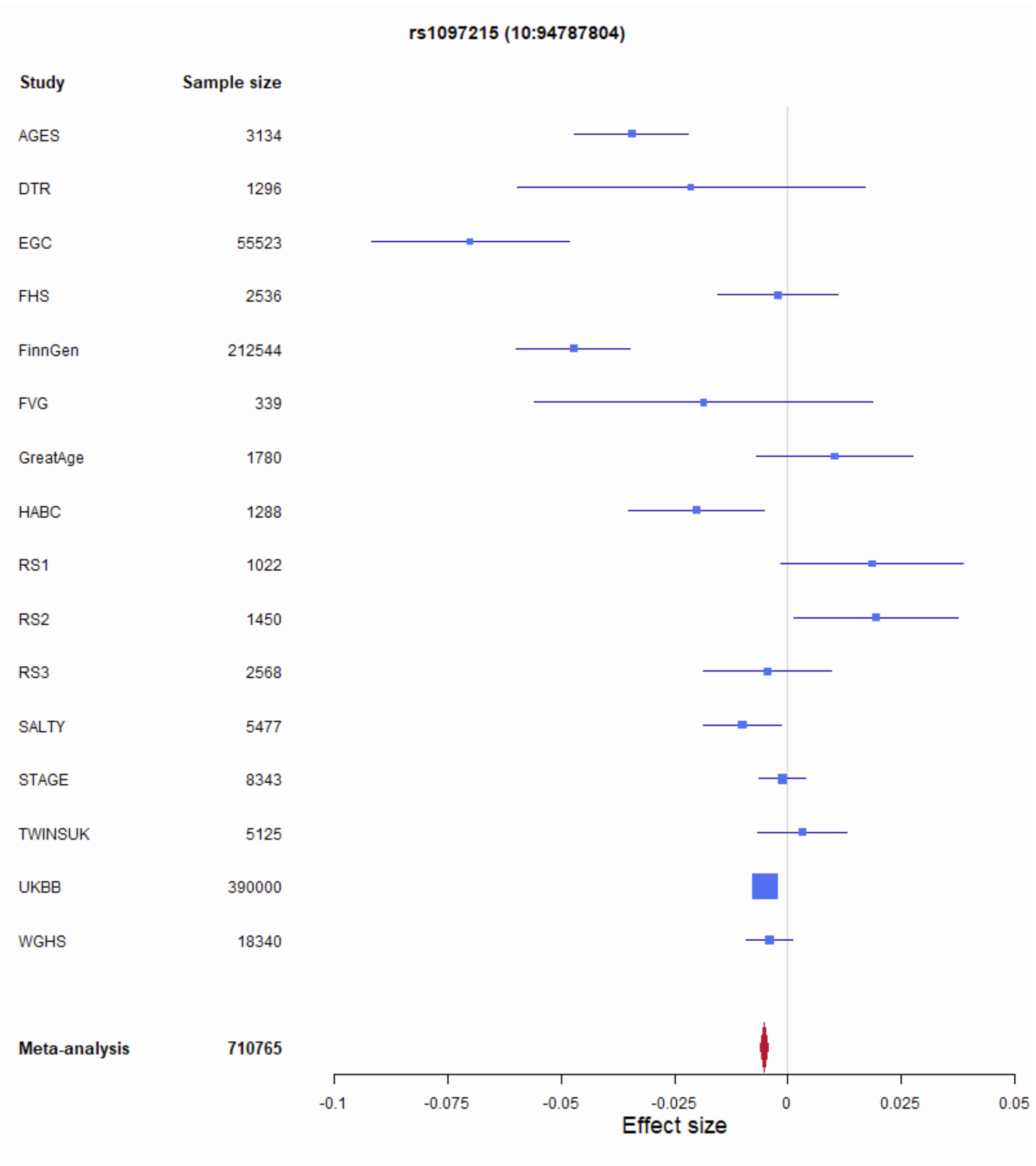

Locus annotation: [EXOC6] intronic

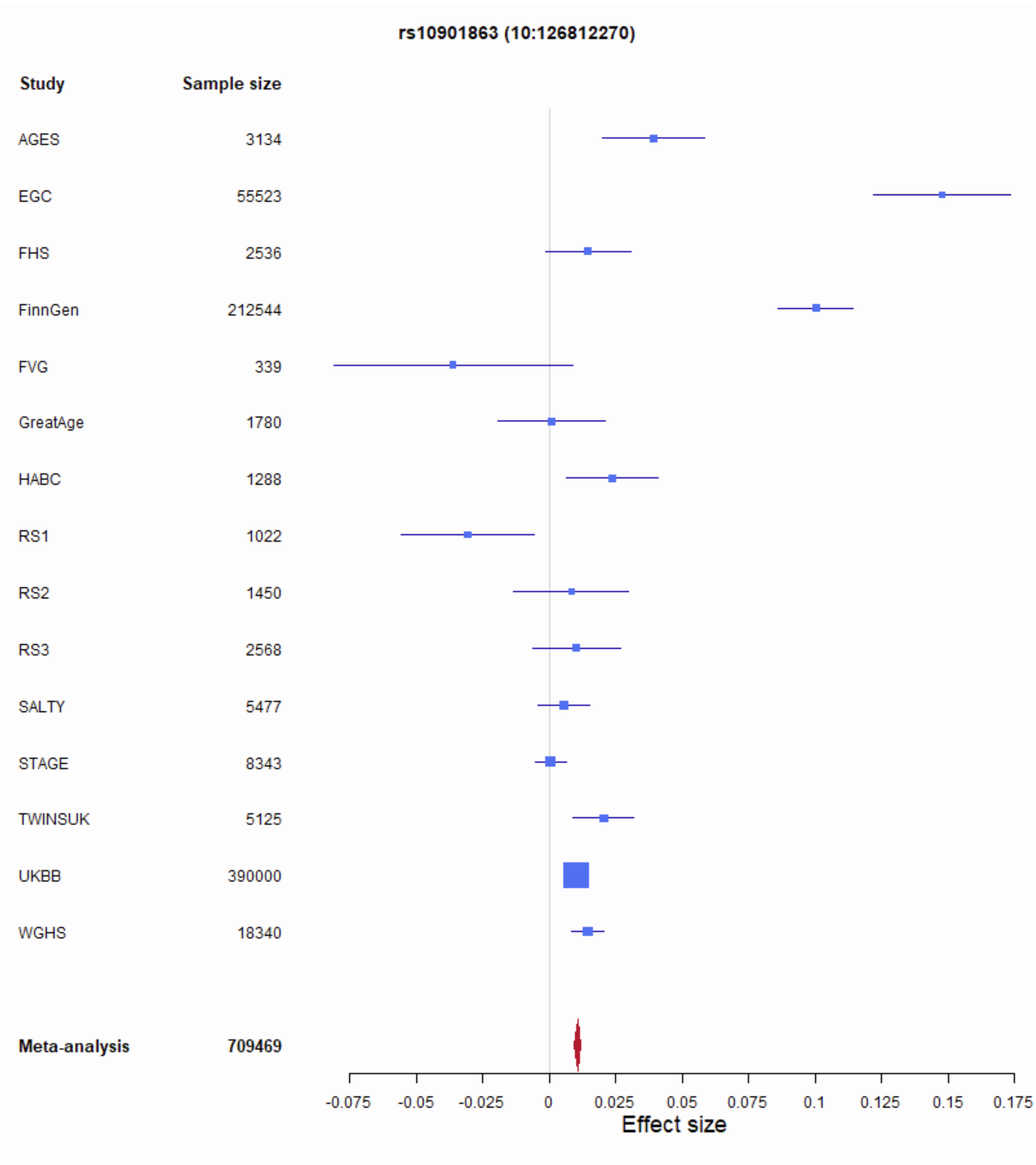

Locus annotation: [CTBP2] 5' UTR

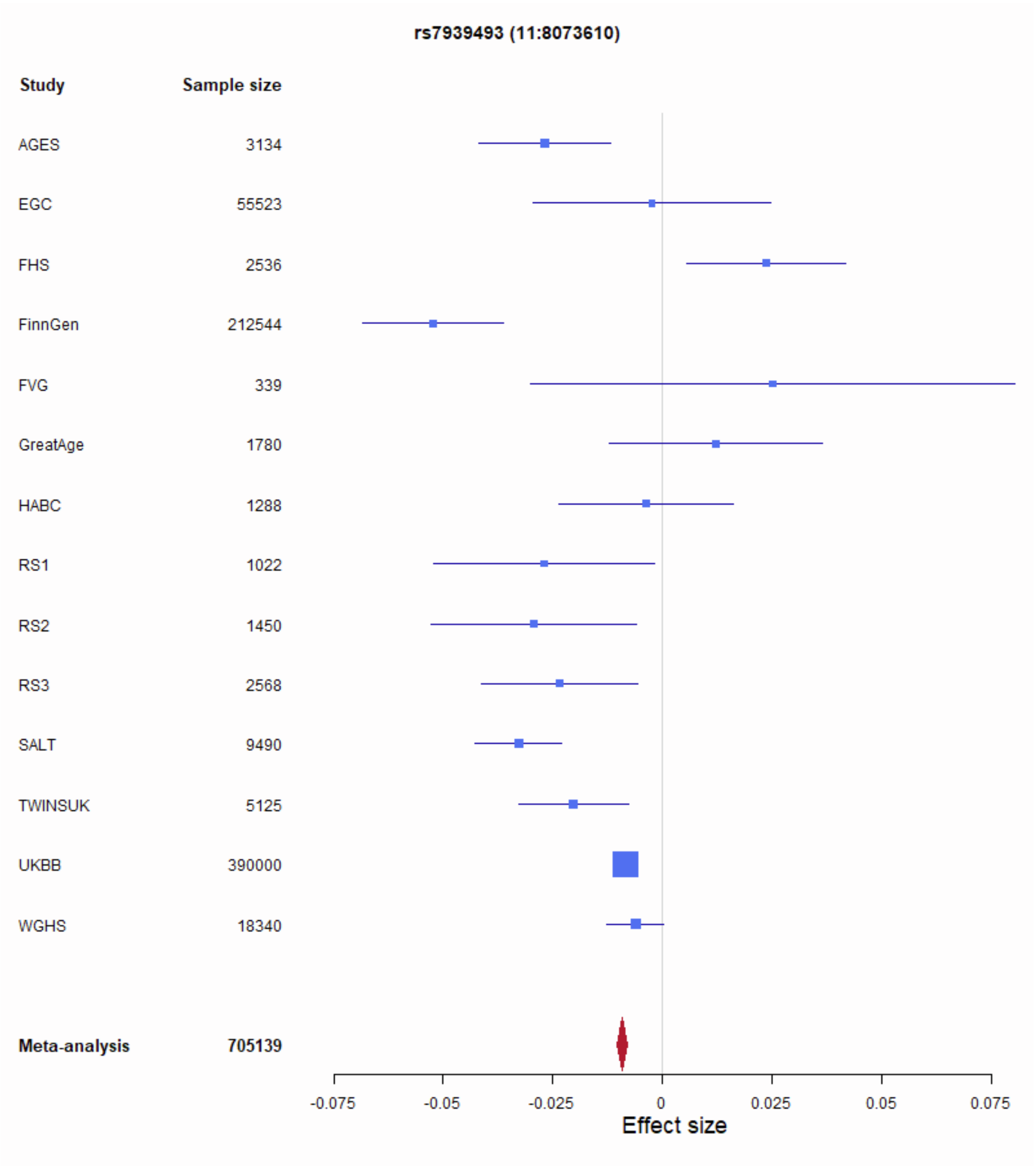

Locus annotation: [TUB] intronic

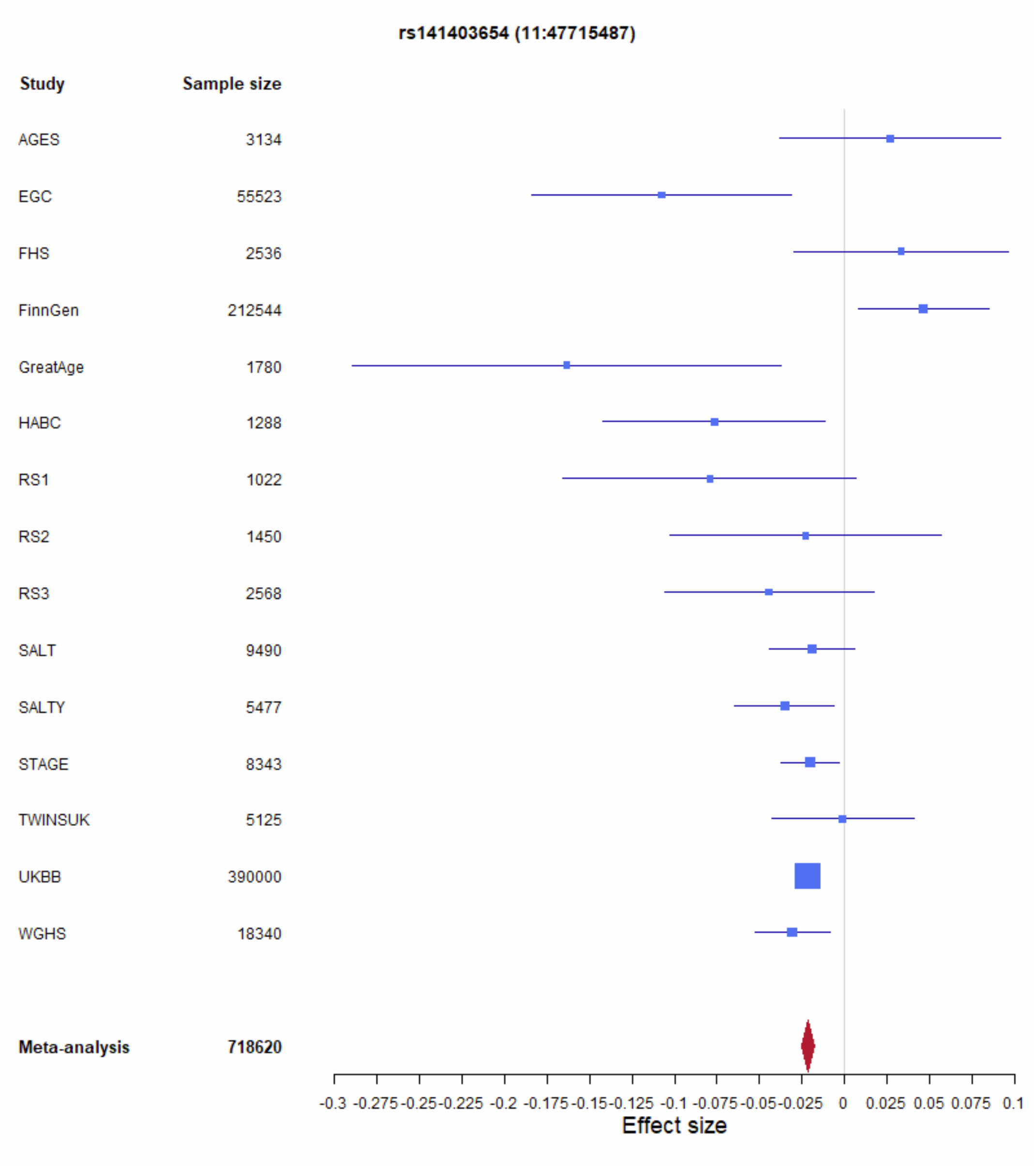

Locus annotation: [AGBL2] intronic

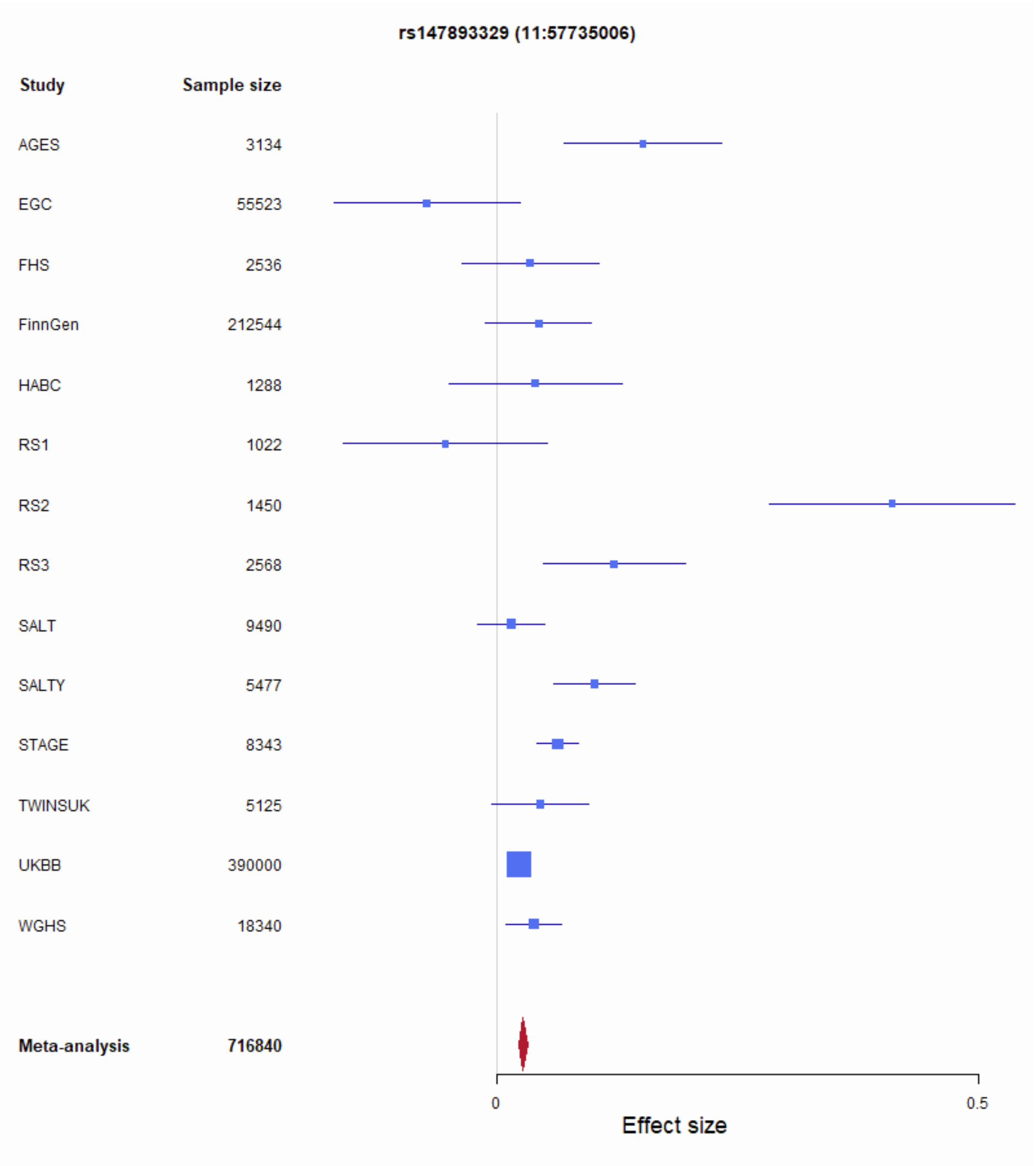

Locus annotation: CTNND1--[x]-OR9Q1

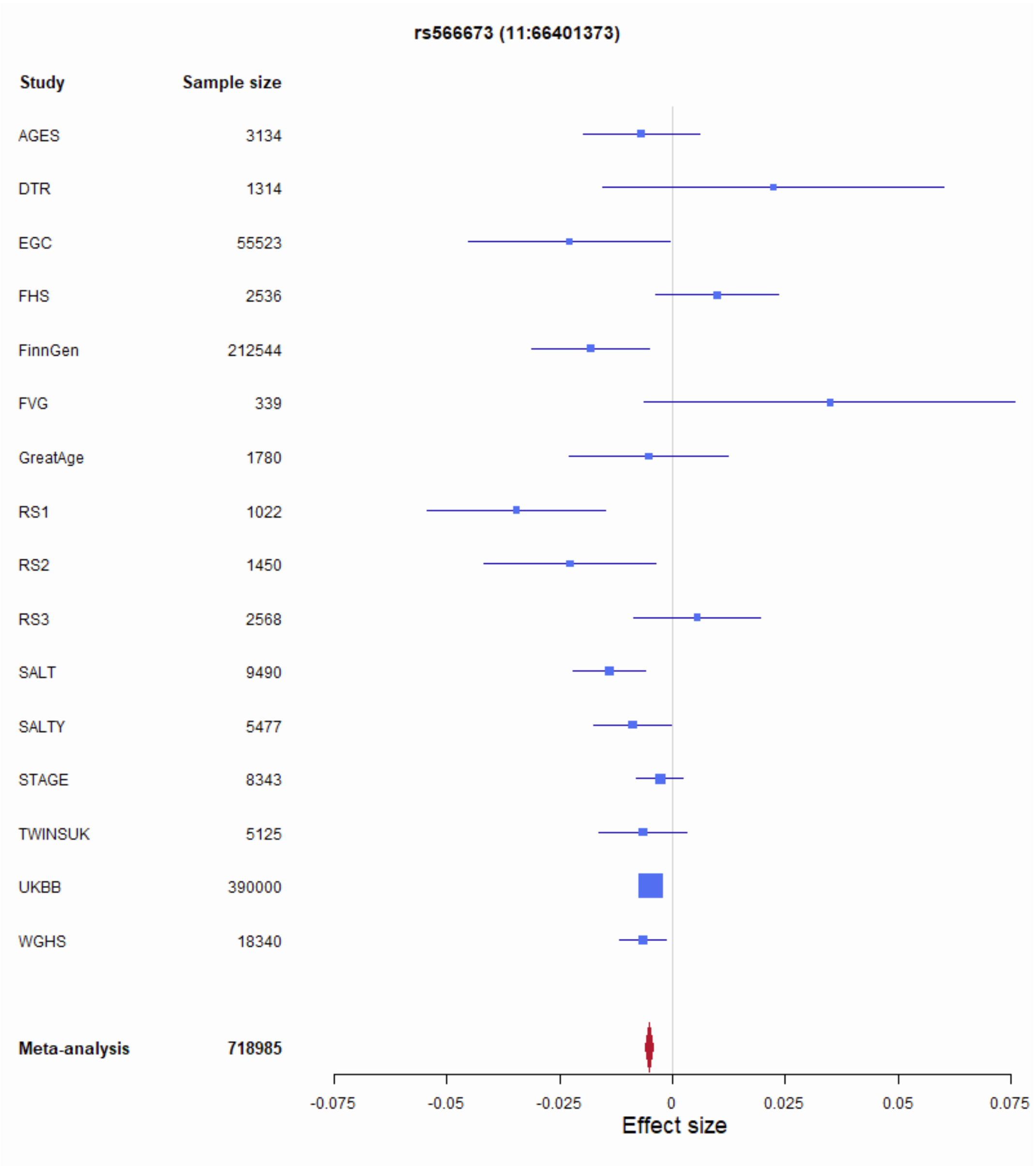

Locus annotation: RBM14-[x]-RBM4

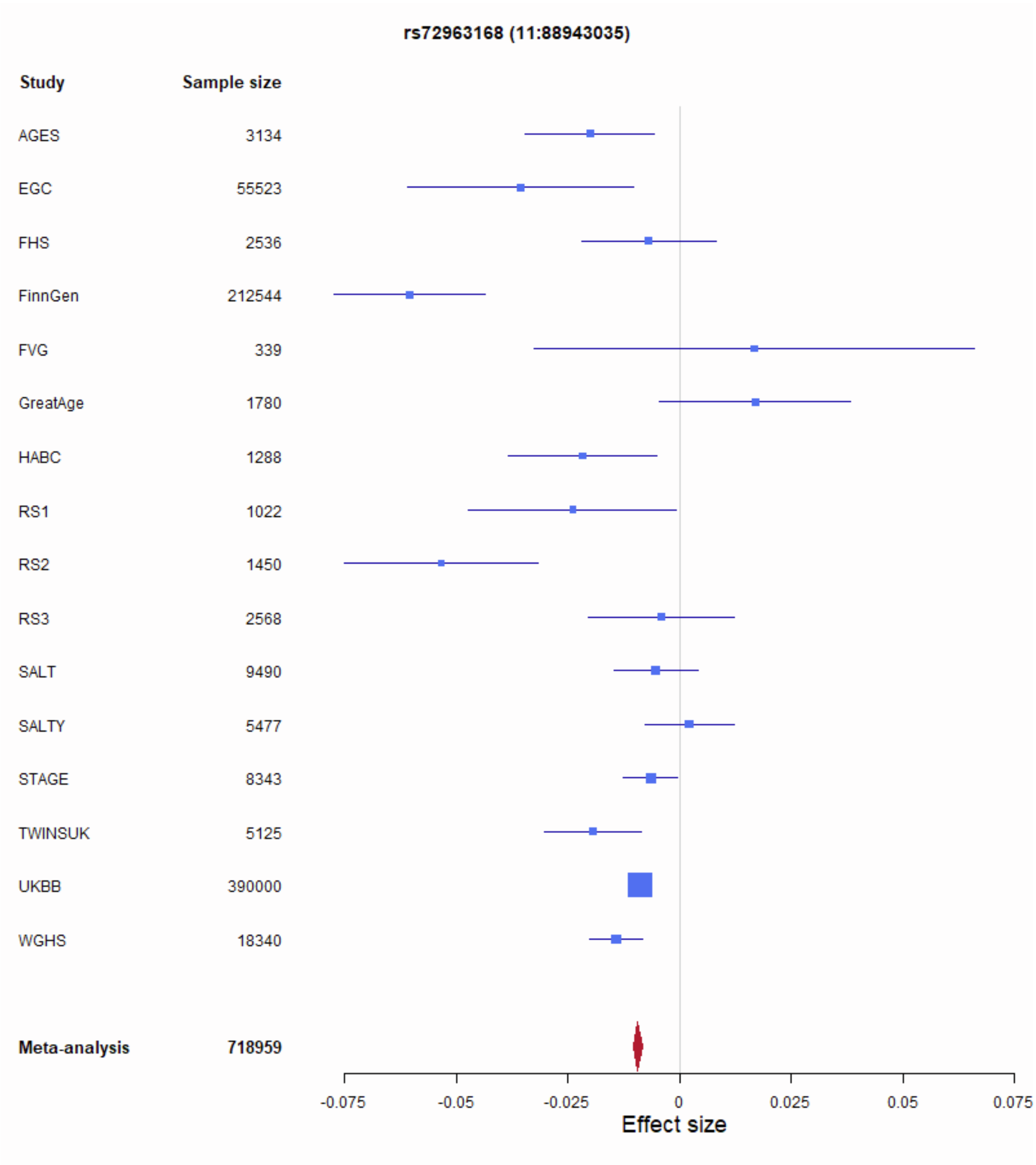

Locus annotation: [TYR] intronic

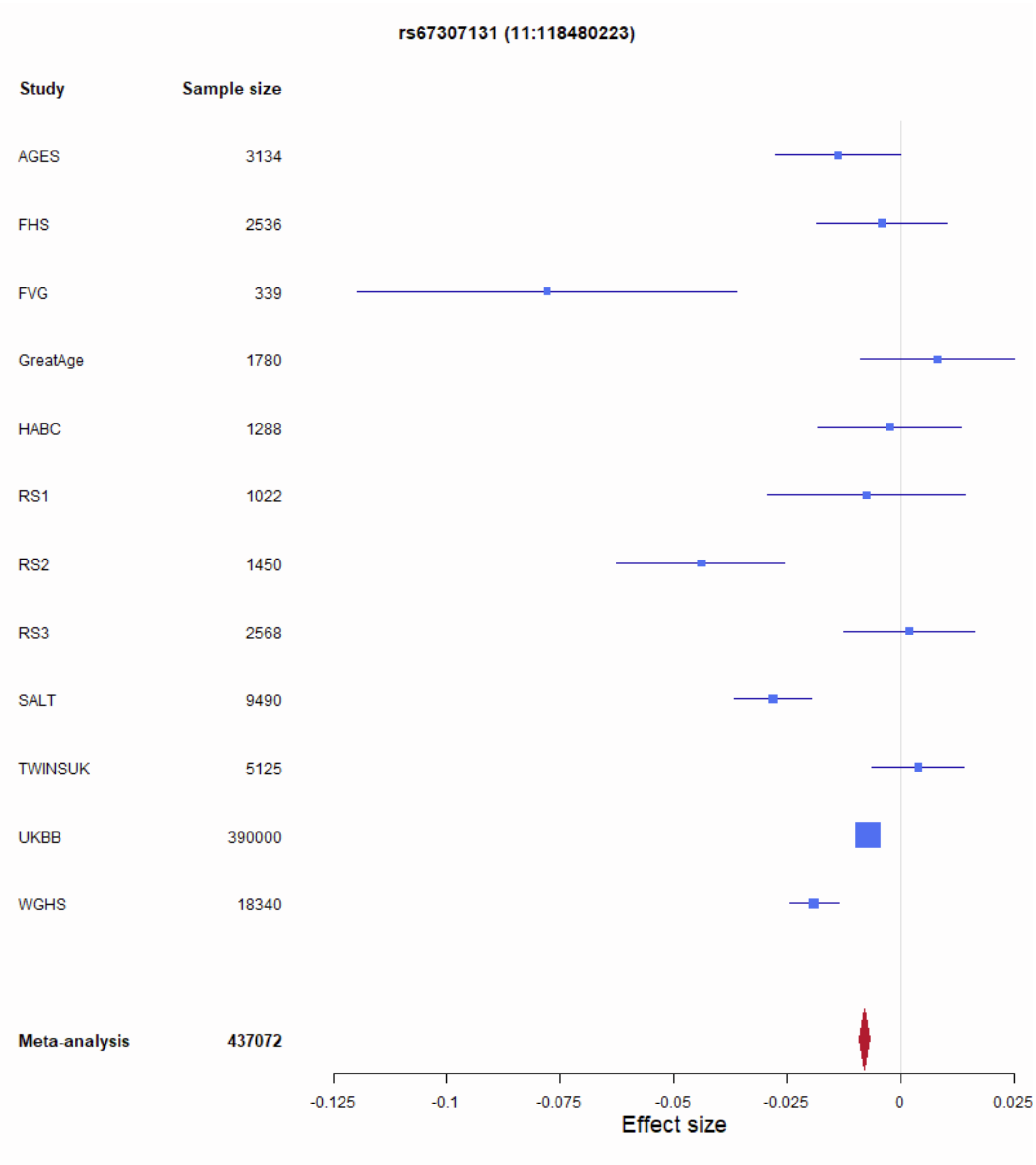

Locus annotation: [PHLDB1] intronic

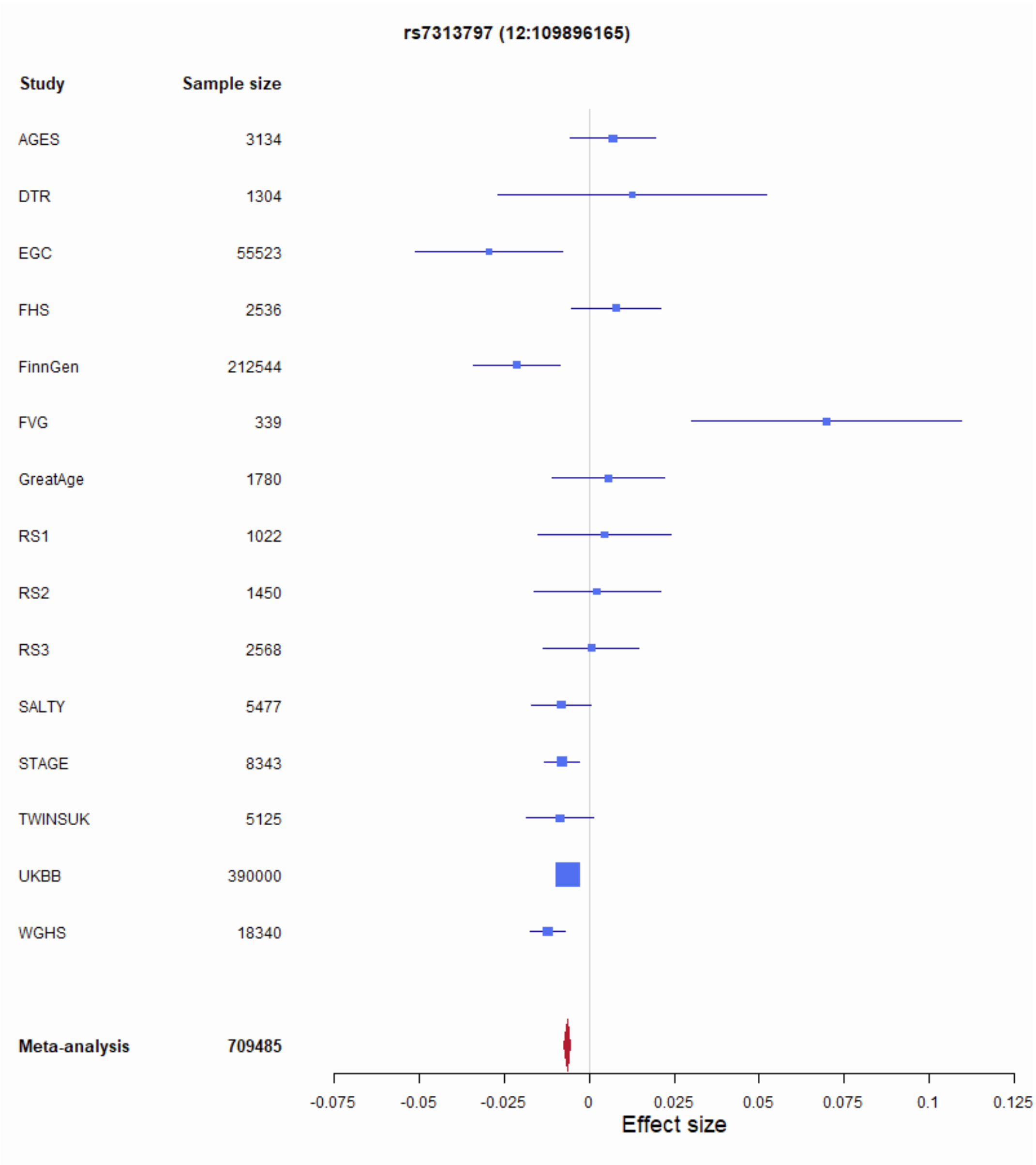

Locus annotation: [KCTD10] intronic

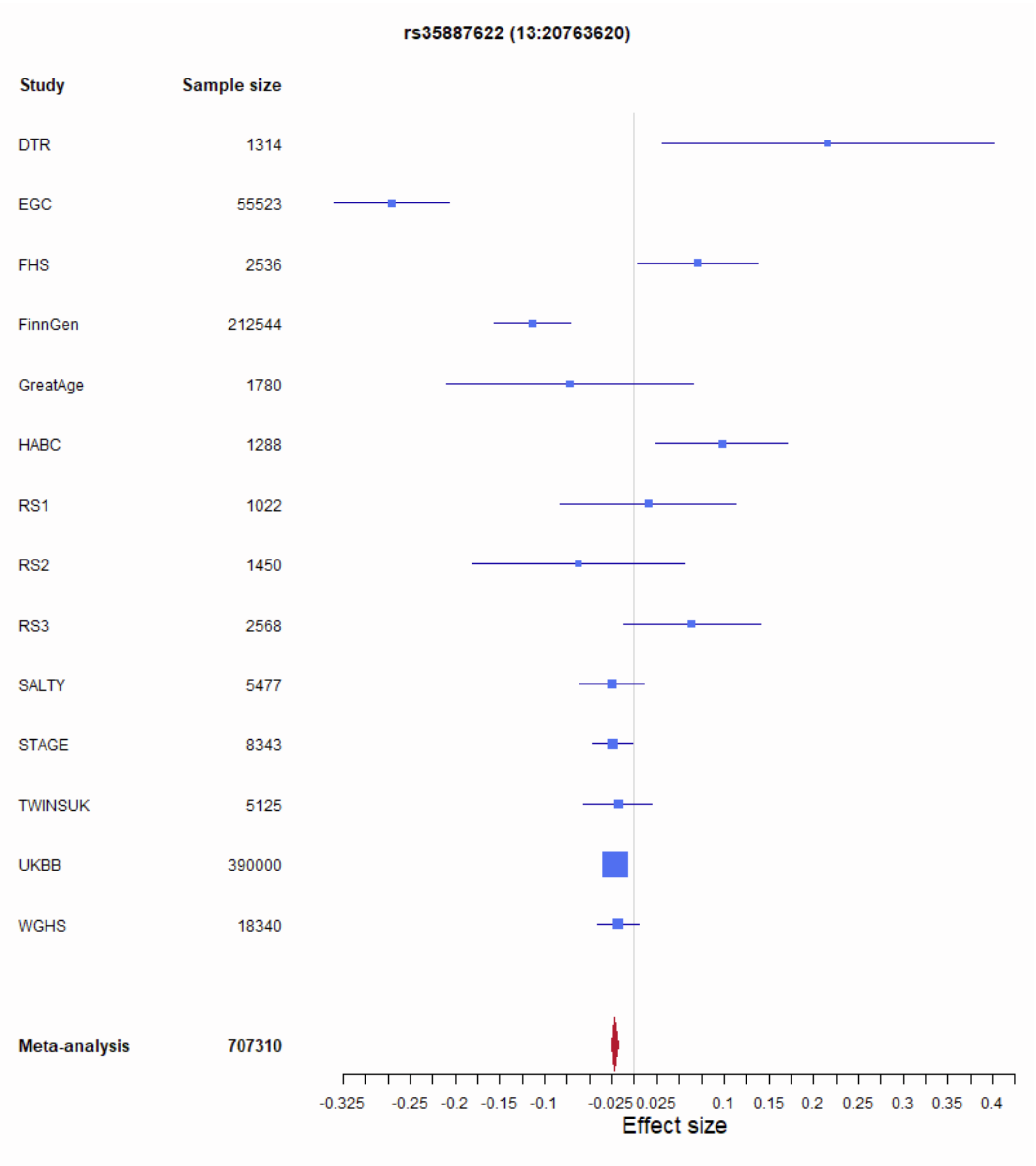

Locus annotation: [GJB2] M>T

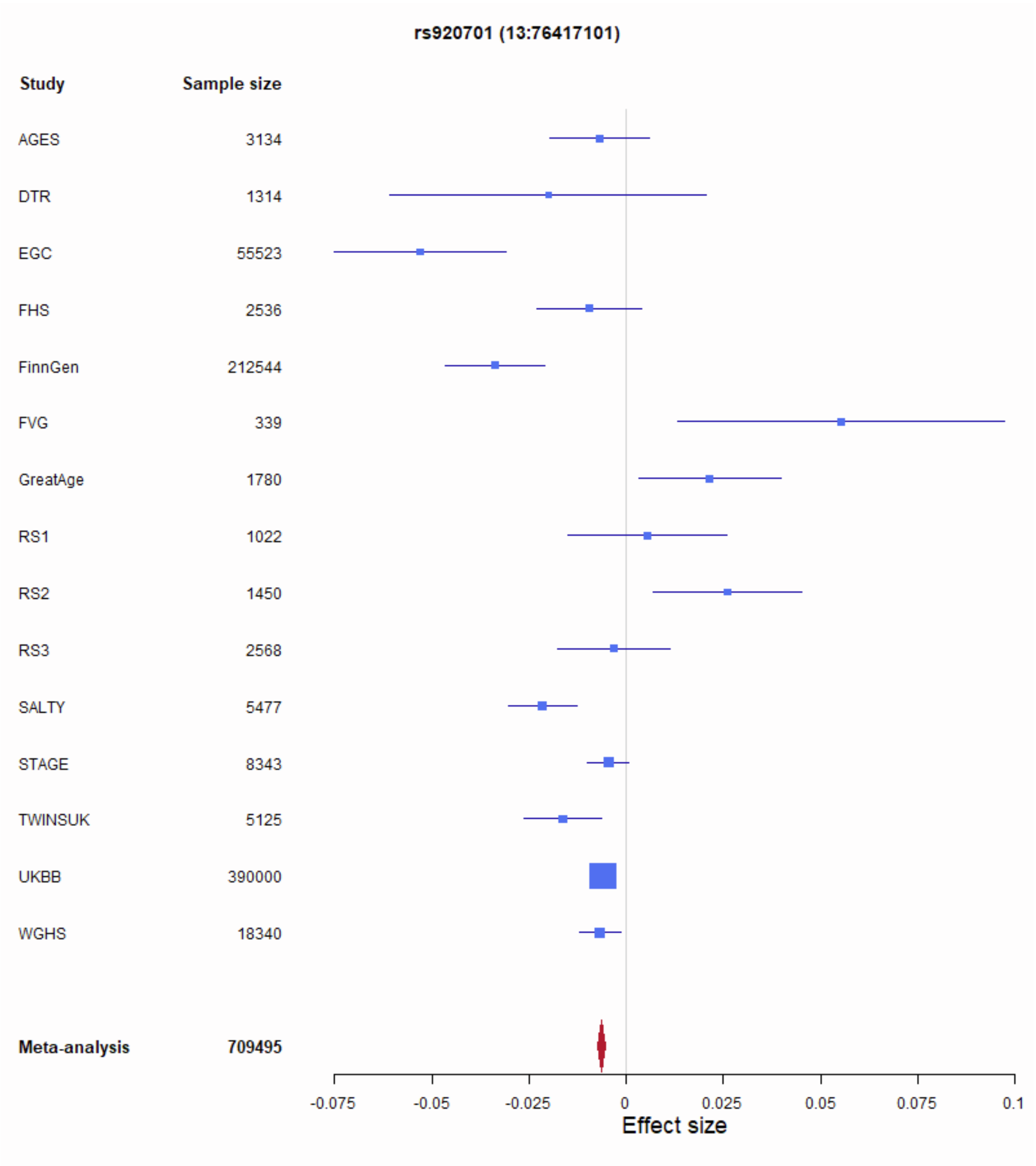

Locus annotation: [LMO7] intronic

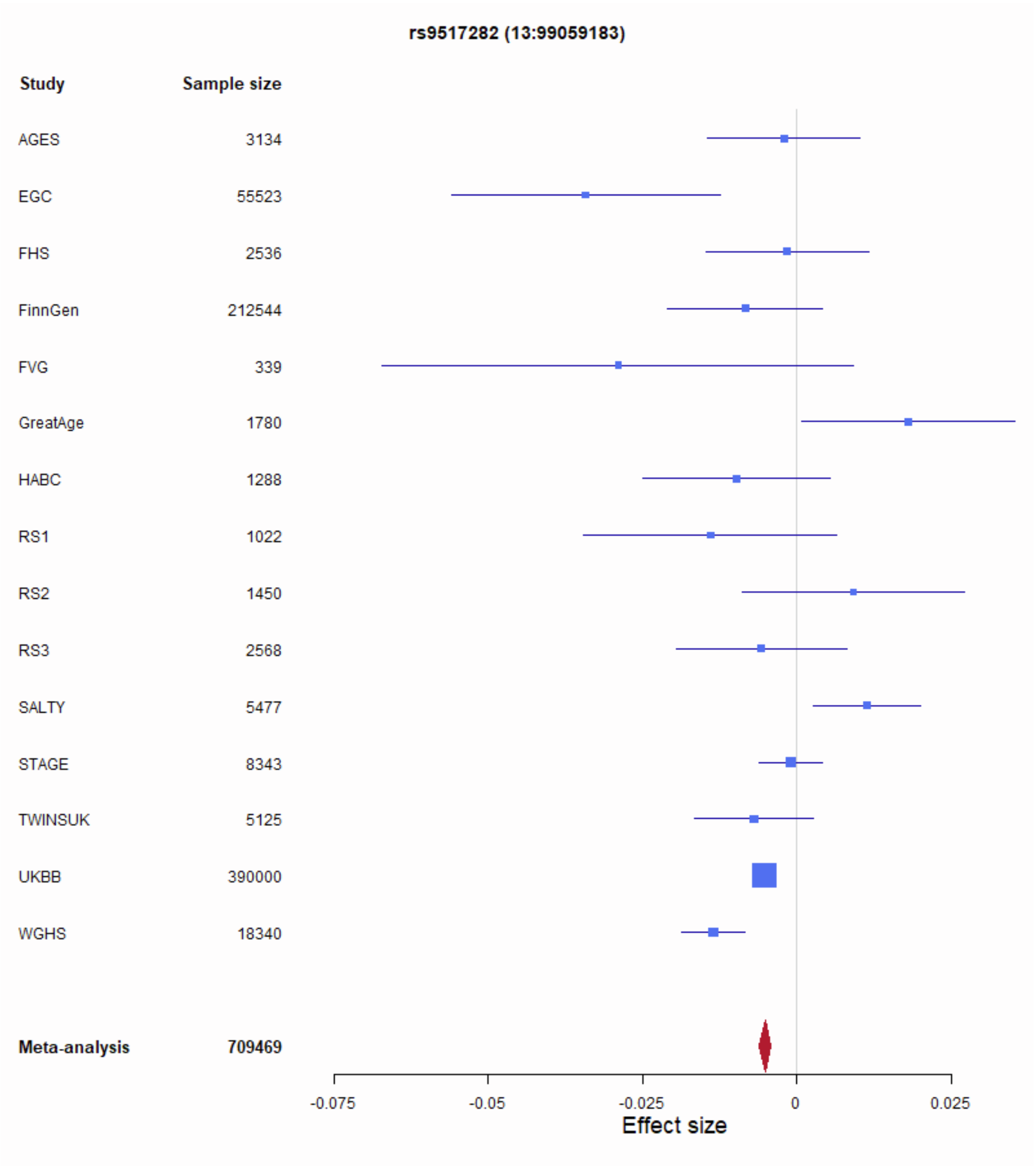

Locus annotation: [FARP1] intronic

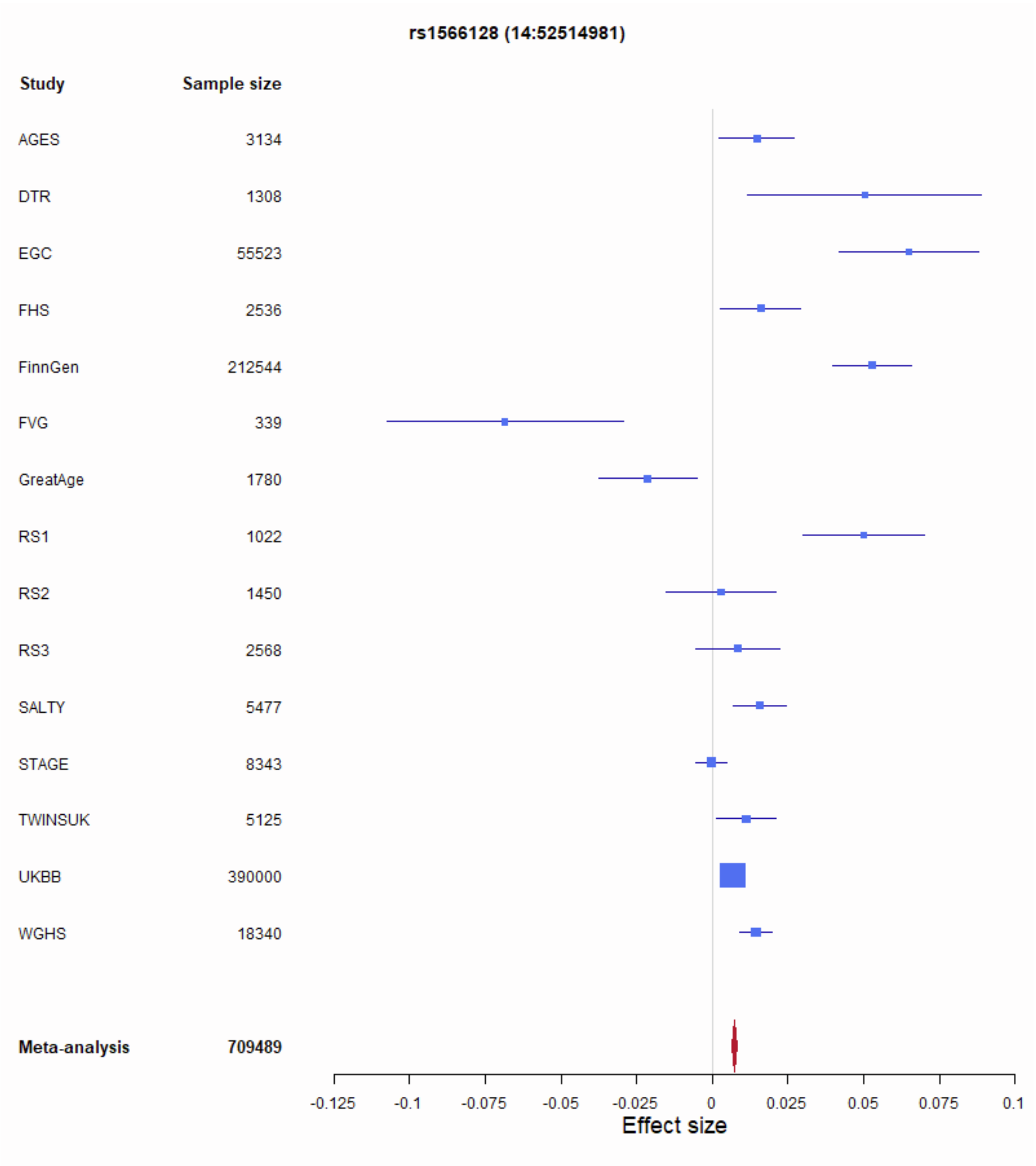

Locus annotation: [NID2] intronic

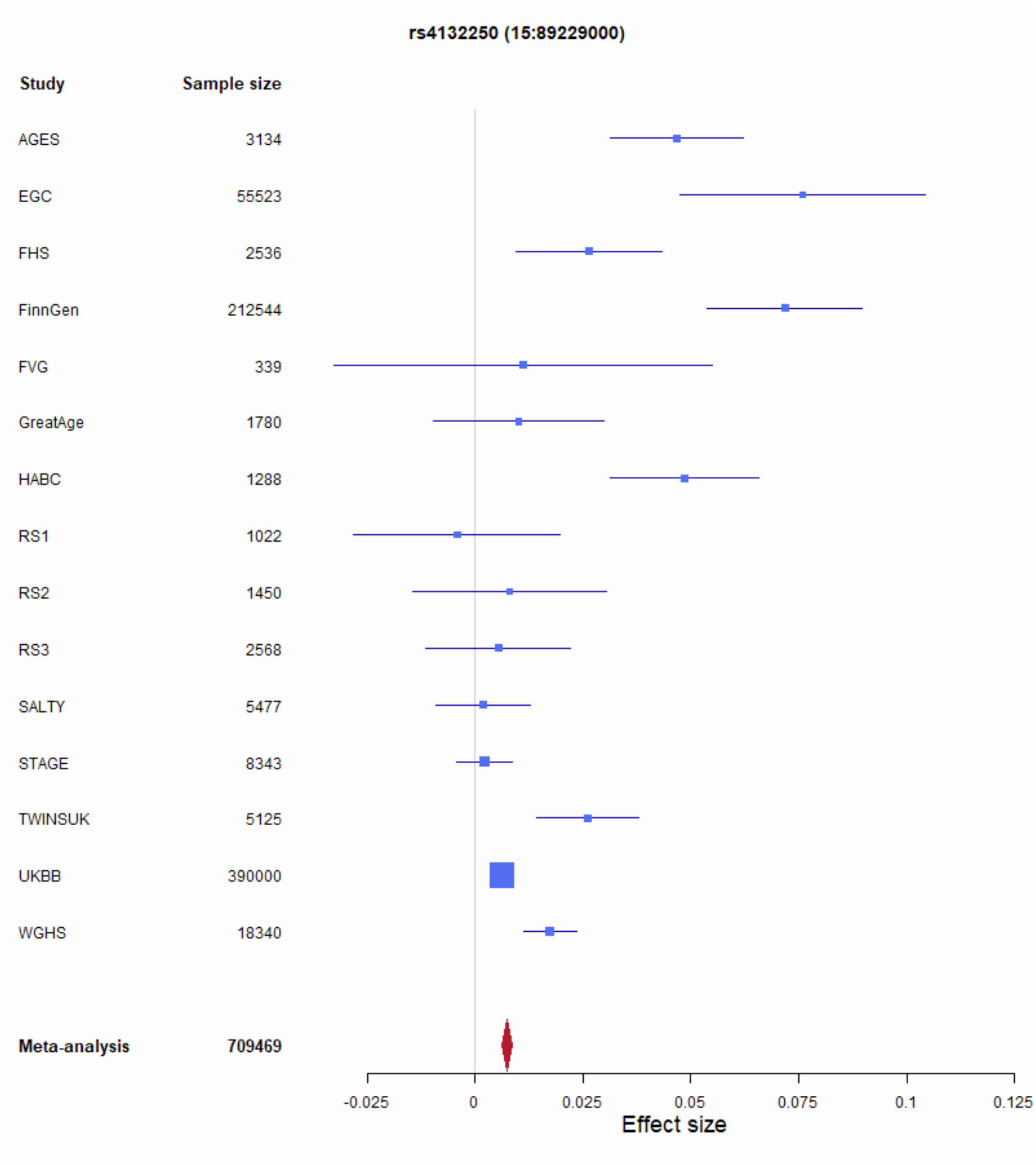

Locus annotation: ISG20-[x]--ACAN

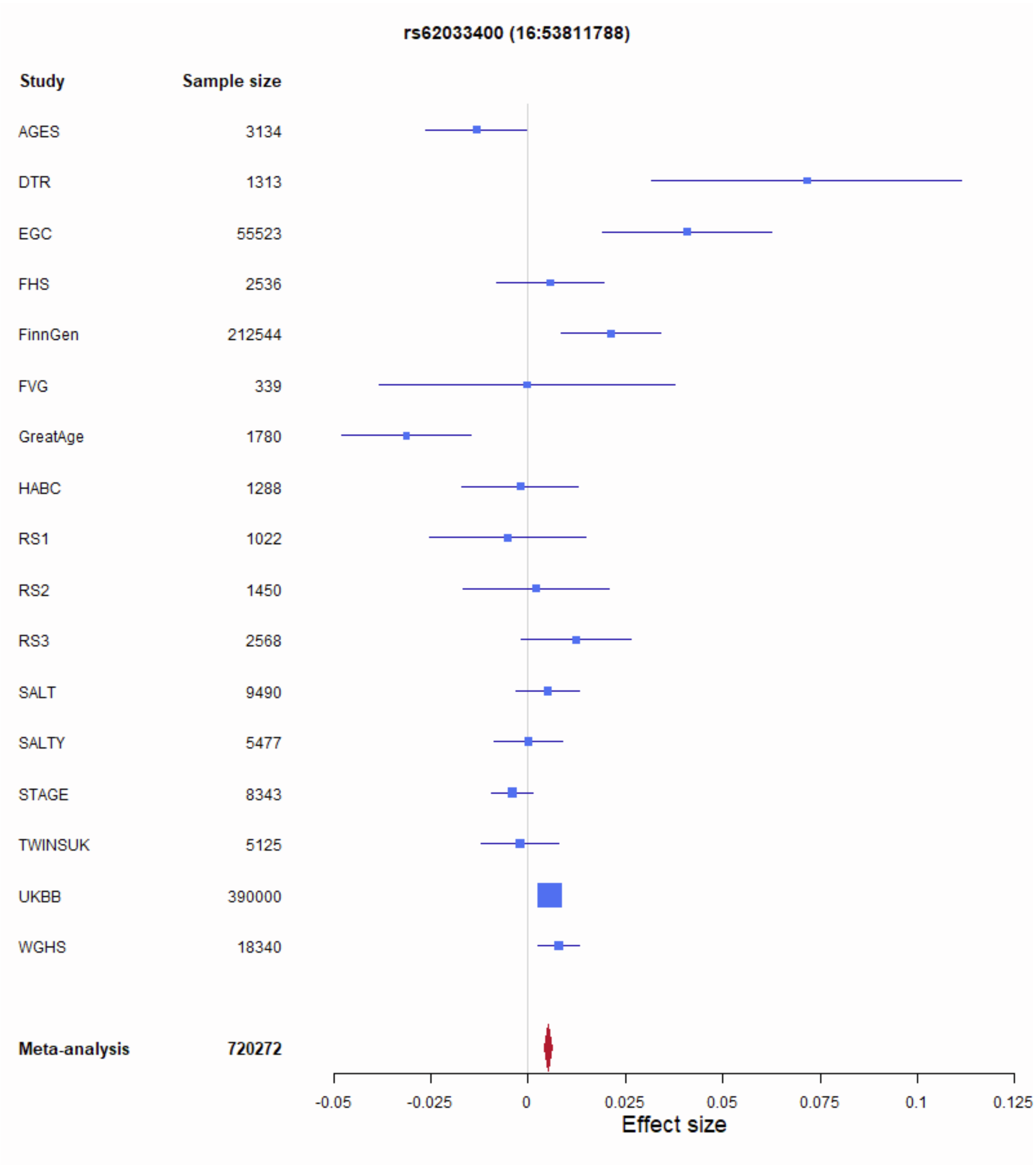

Locus annotation: [FTO] intronic

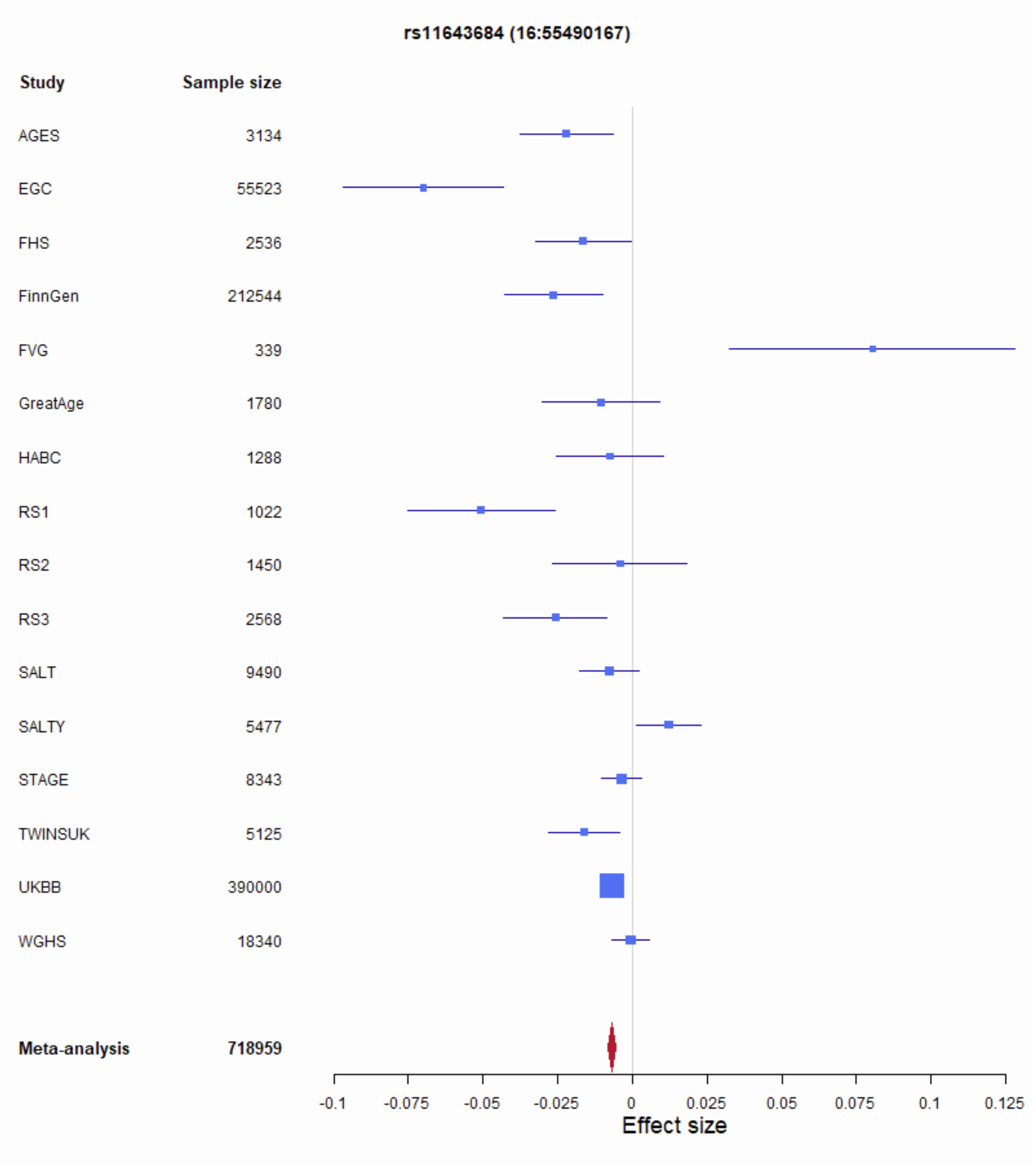

Locus annotation: IRX6--[x]-MMP2

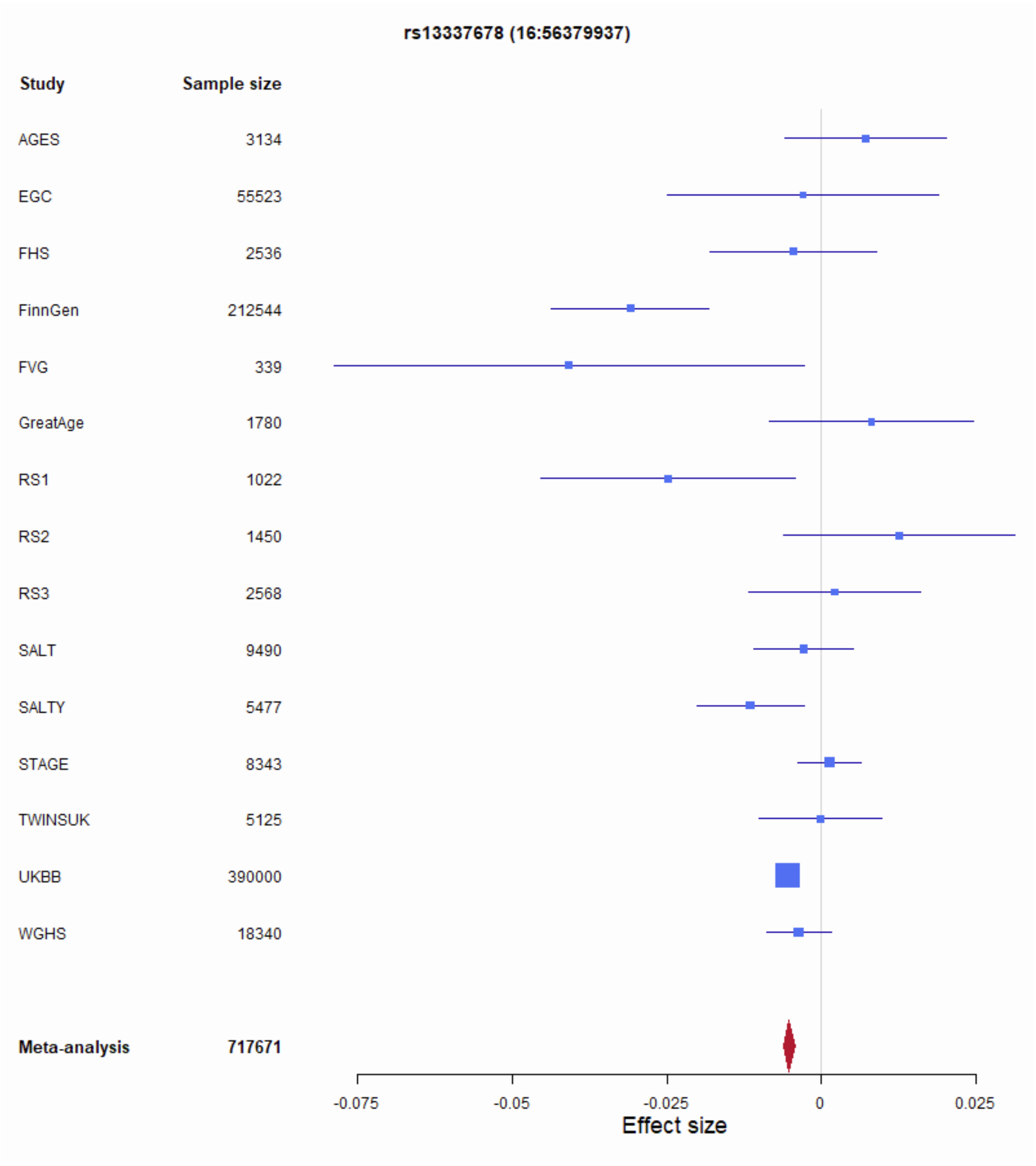

Locus annotation: [GNAO1] 3' UTR

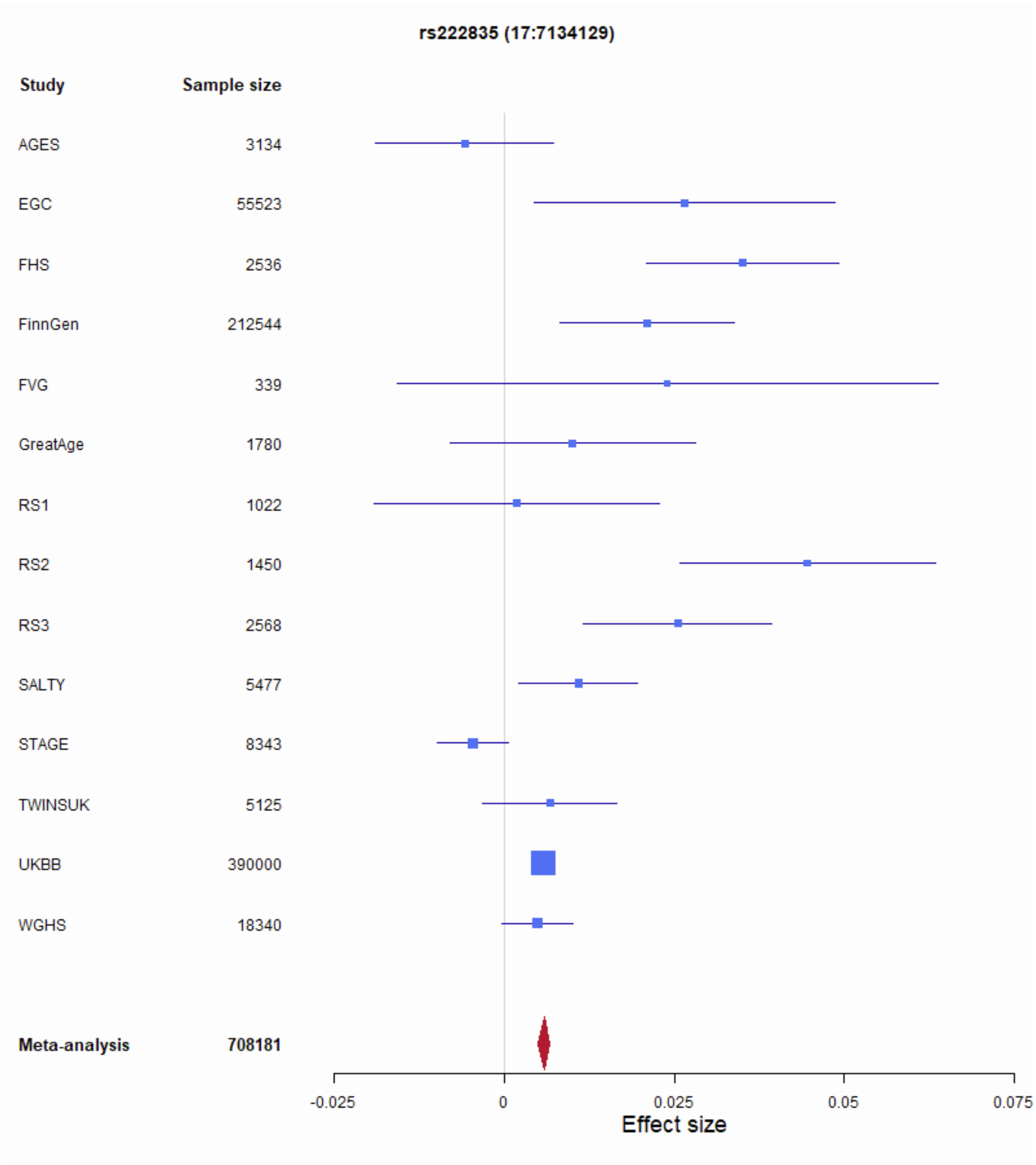

Locus annotation: [DVL2] intronic

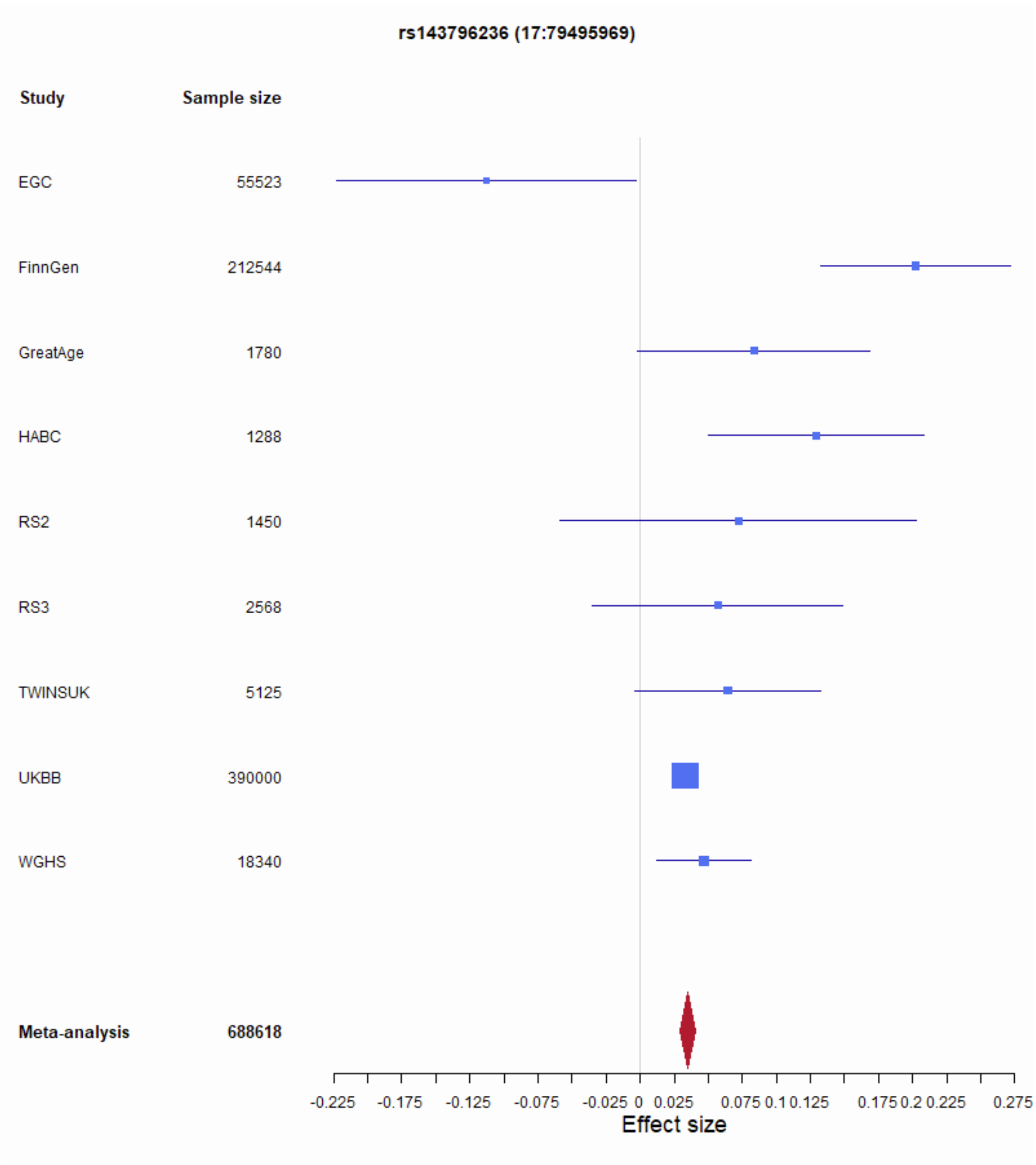

Locus annotation: [FSCN2] H>Y

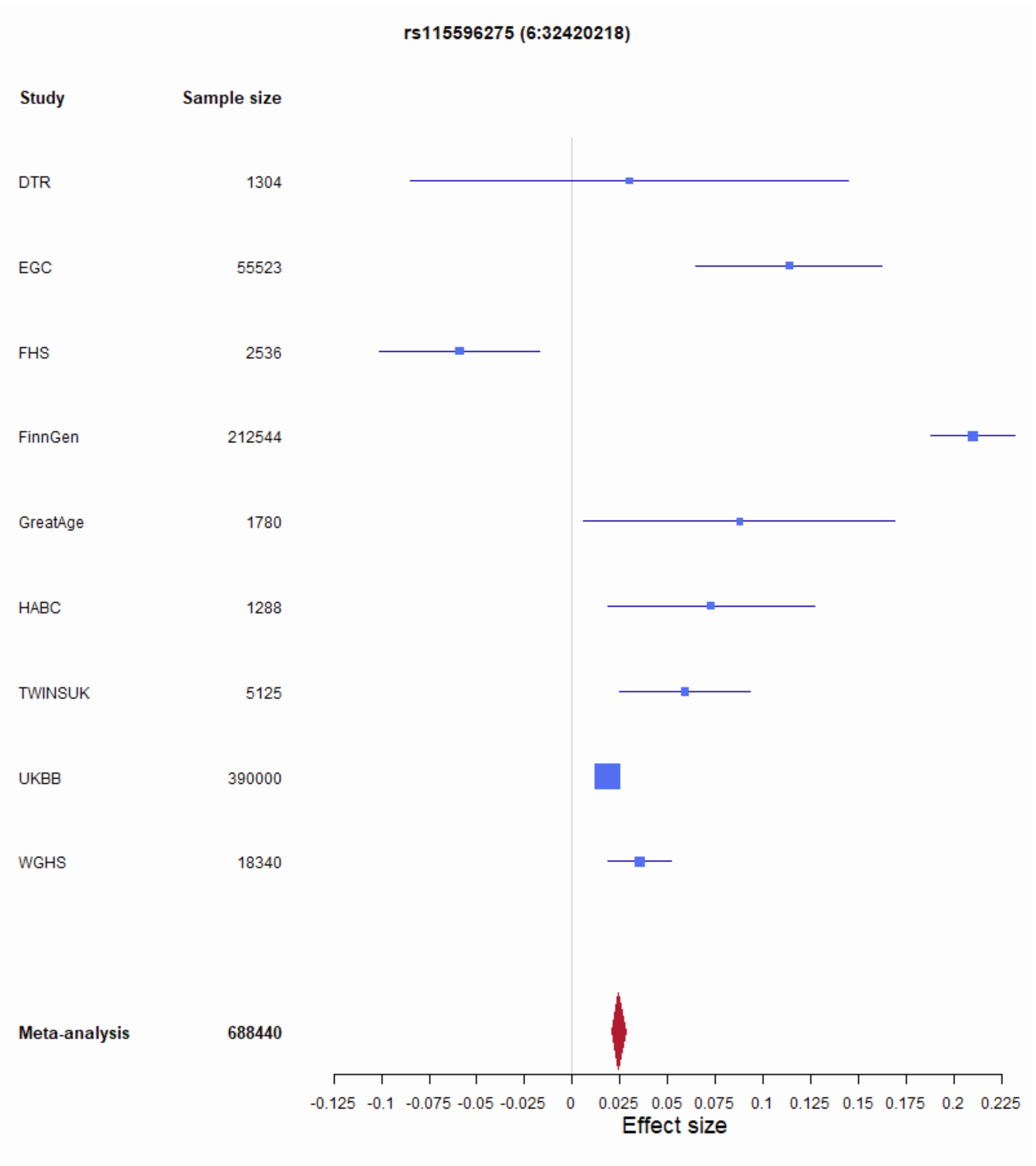

Locus annotation: [CCDC68] 5' UTR

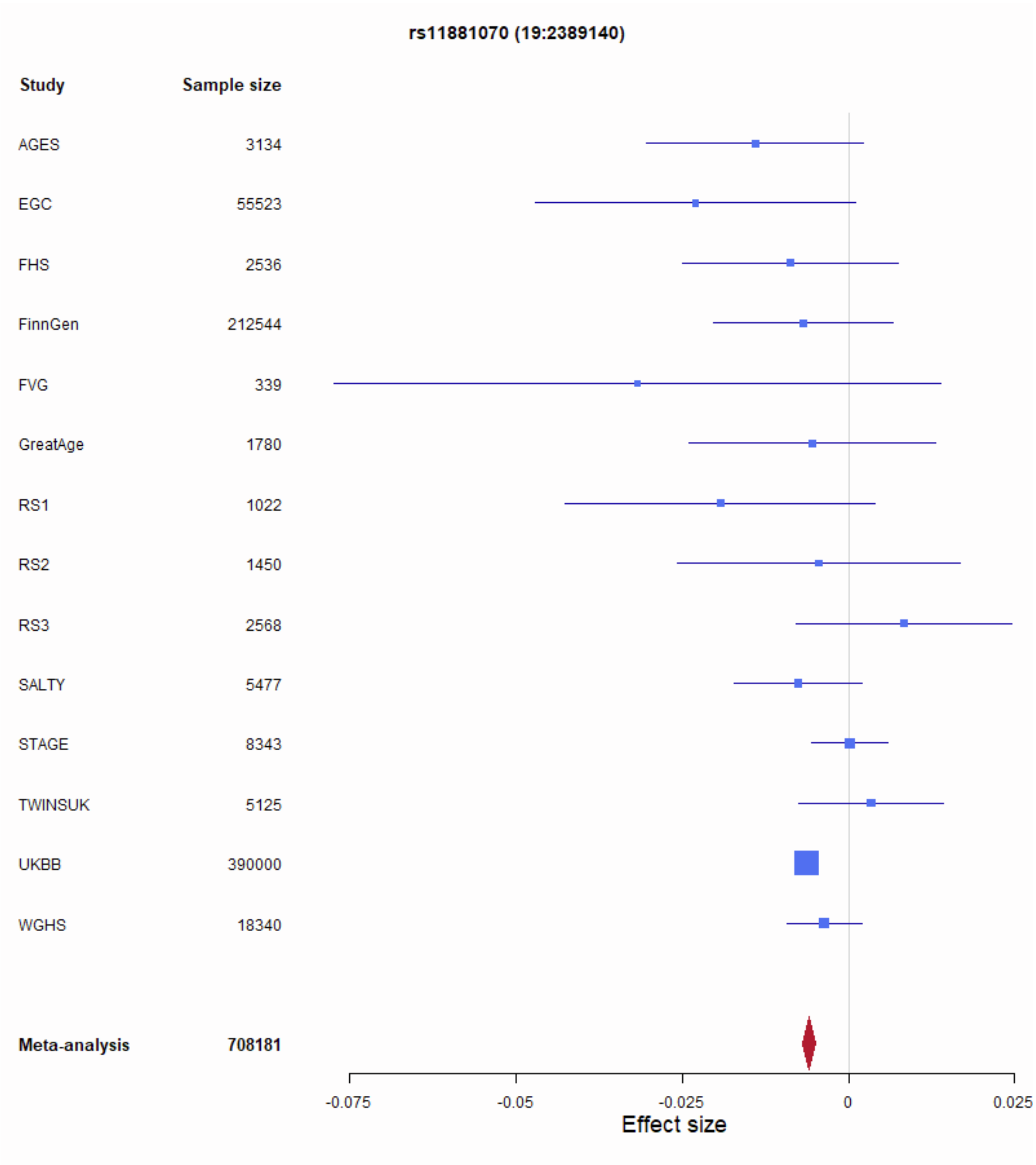

Locus annotation: SPPL2B-[x]-TMRPS9

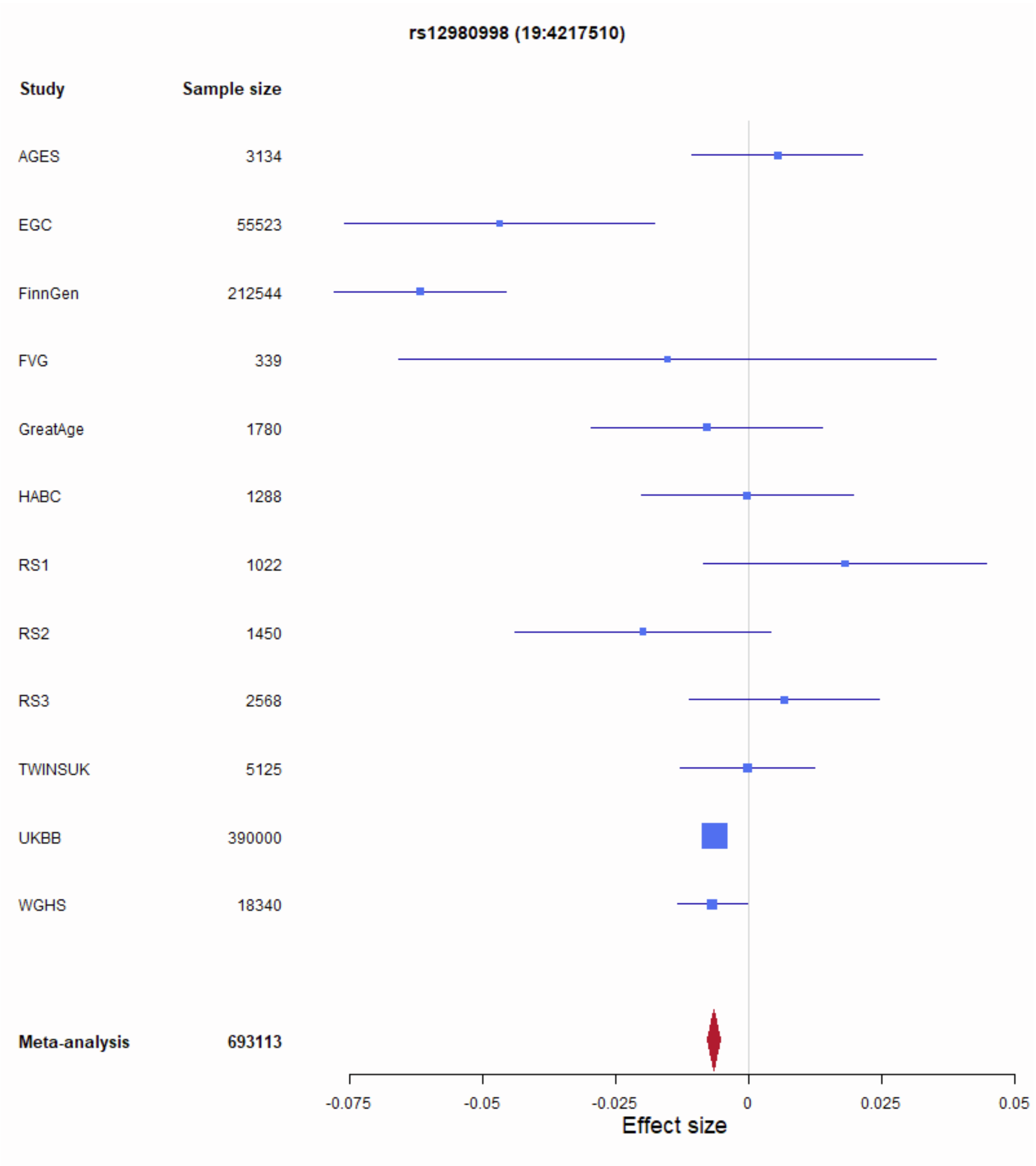

Locus annotation: [ANKRD24] T>S

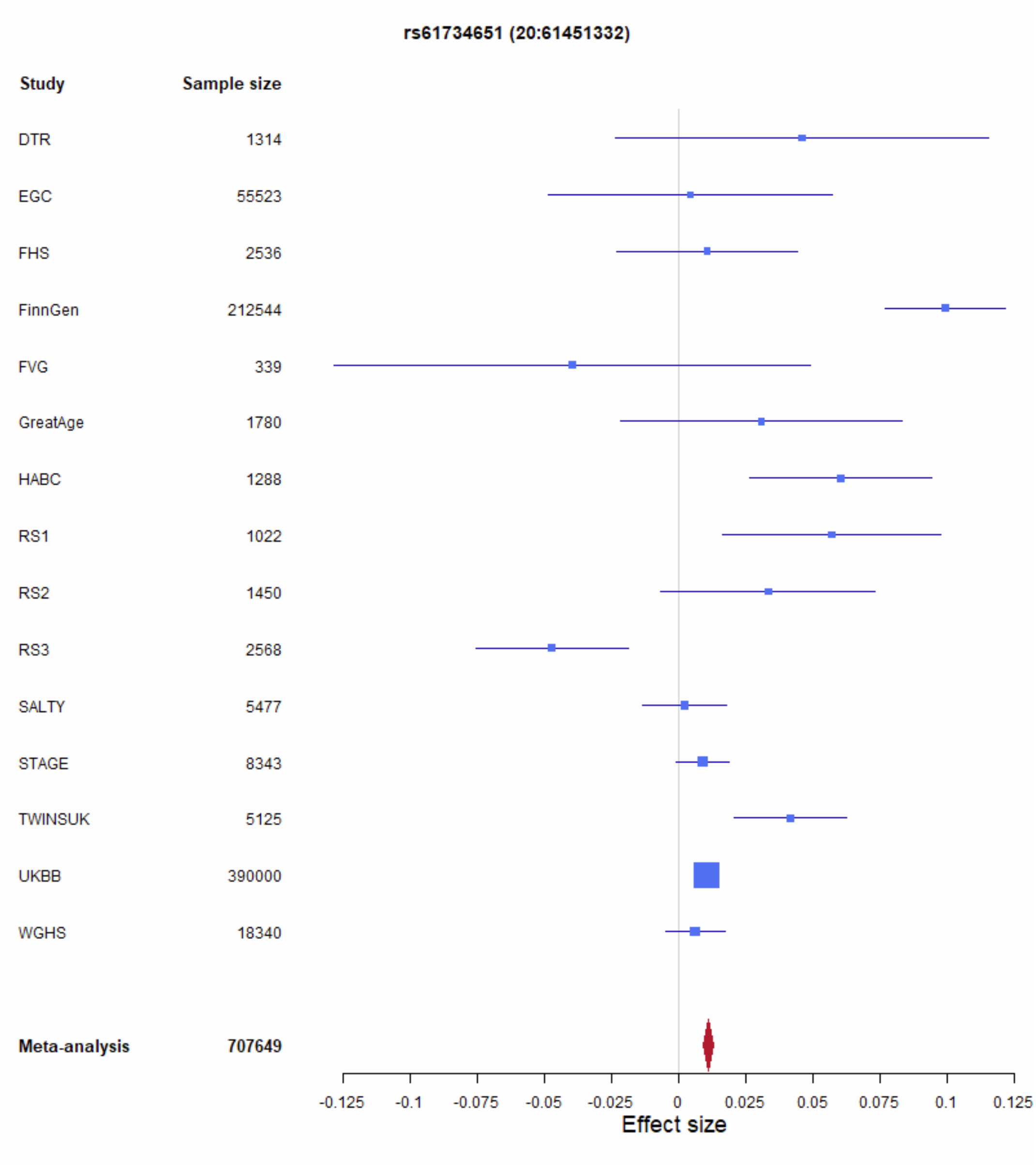

Locus annotation: [COL9A3] R>W

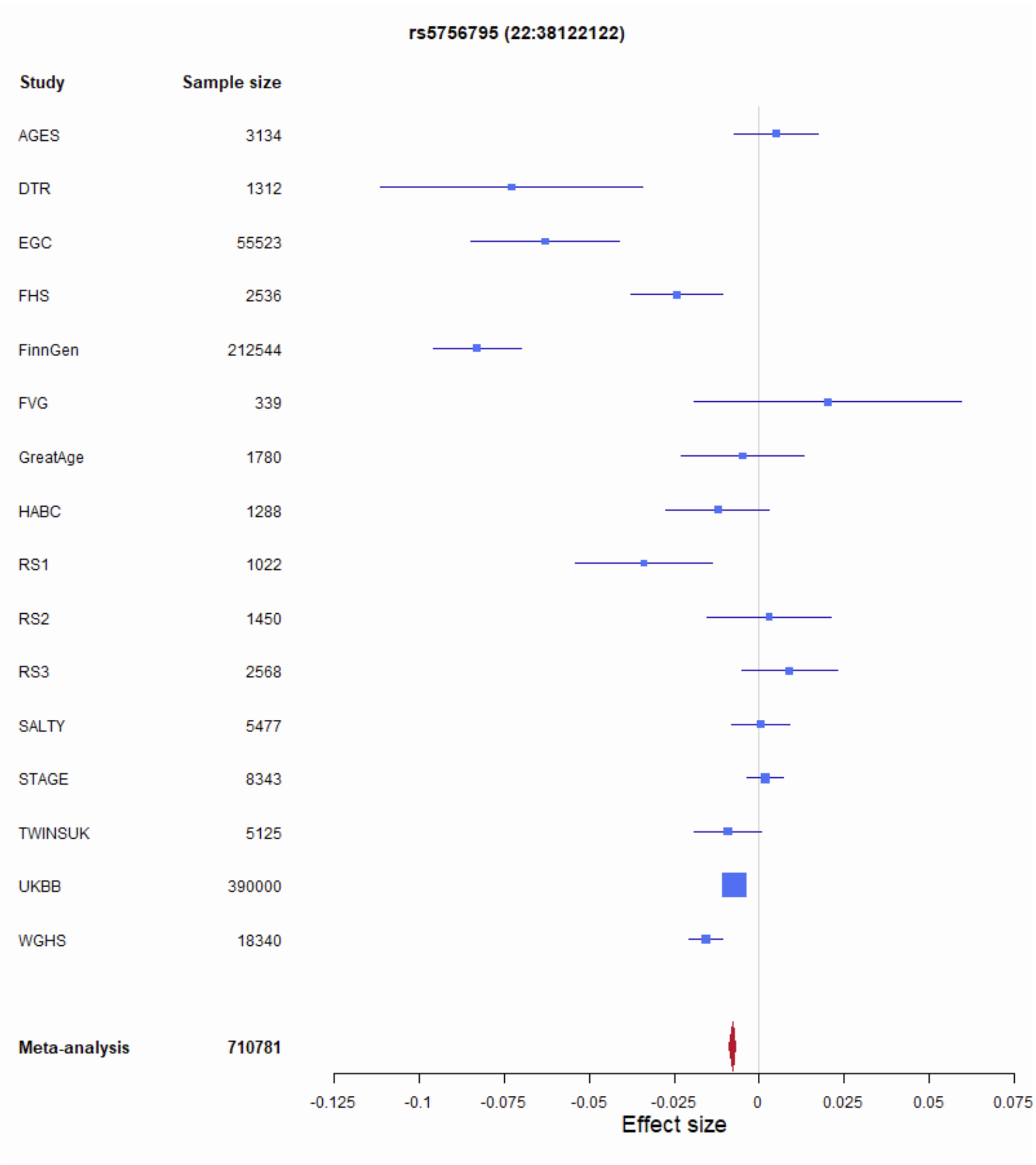

Locus annotation: [TRIOBP] F>I

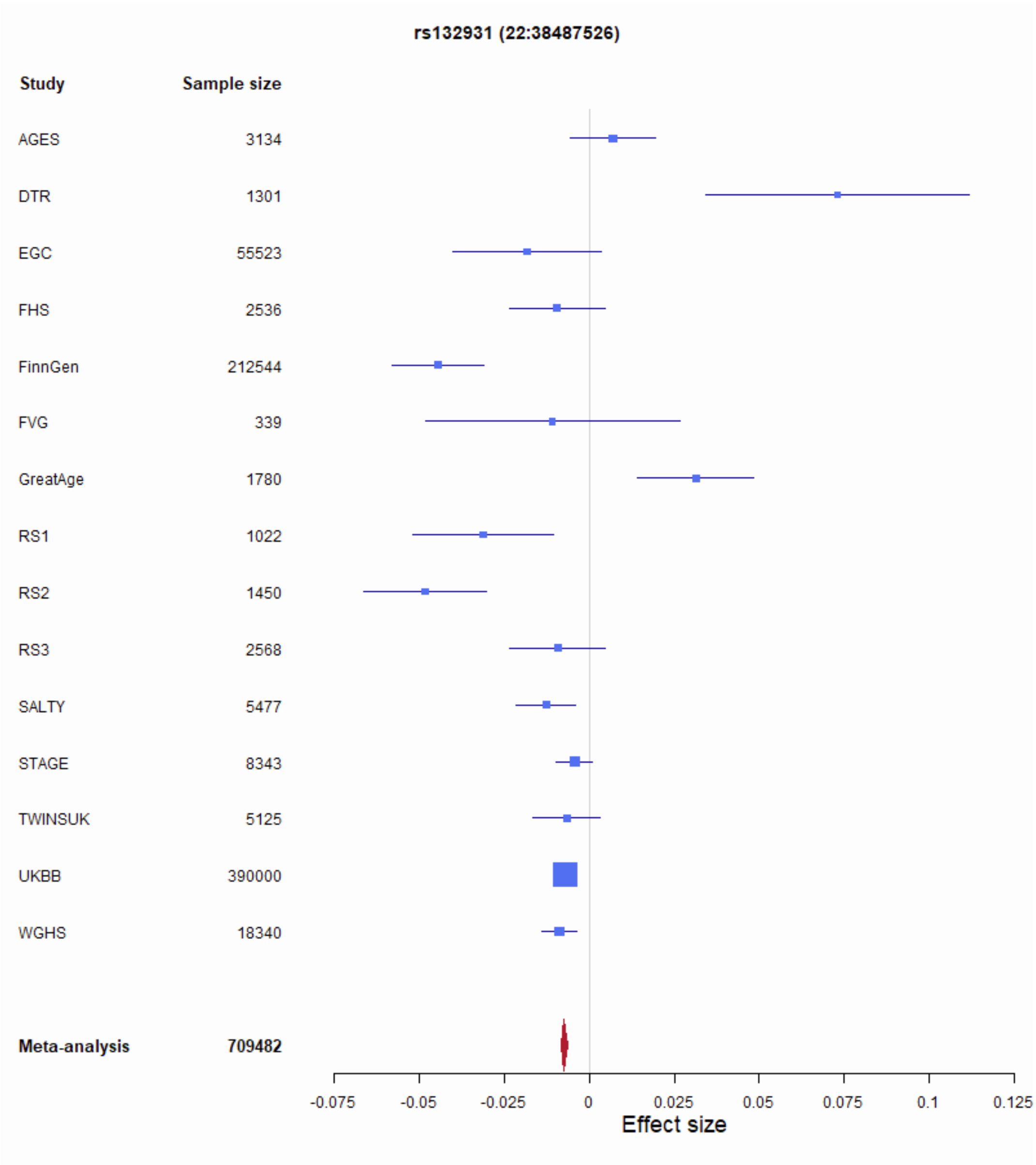

Locus annotation: [BAIAP2L2] intronic

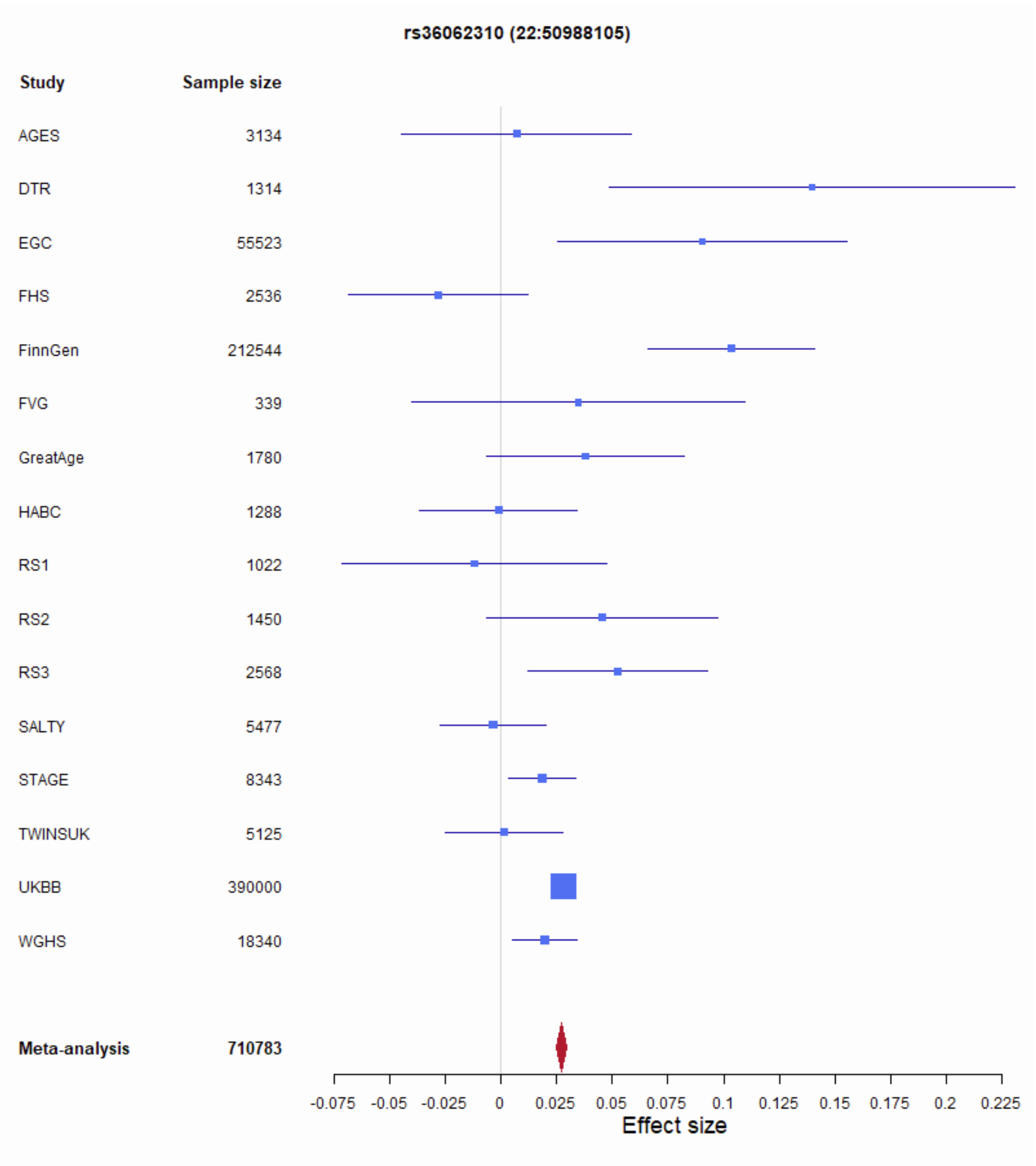

Locus annotation: [KLHDC7B] V>M

Figure S4. Manhattan plot for gene-based analysis. All significant genes (p<2.66e-6) identified by MAGMA (red) and genes identified by MAGMA and VEGAS simultaneously (blue).

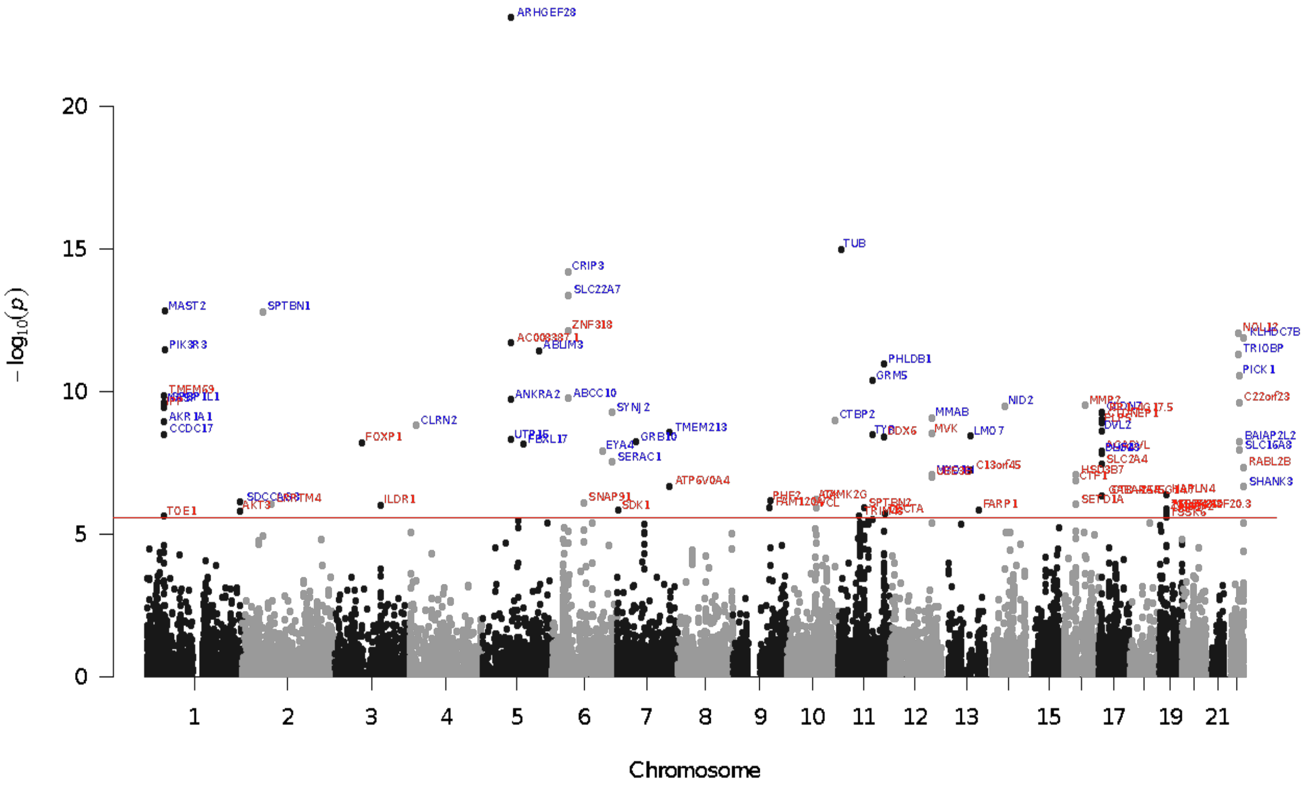

Figure S5. a) Protein structure and modeling of the missense variant effect on GJB2 transporter b) Hydrophilicity/hydrophobicity map

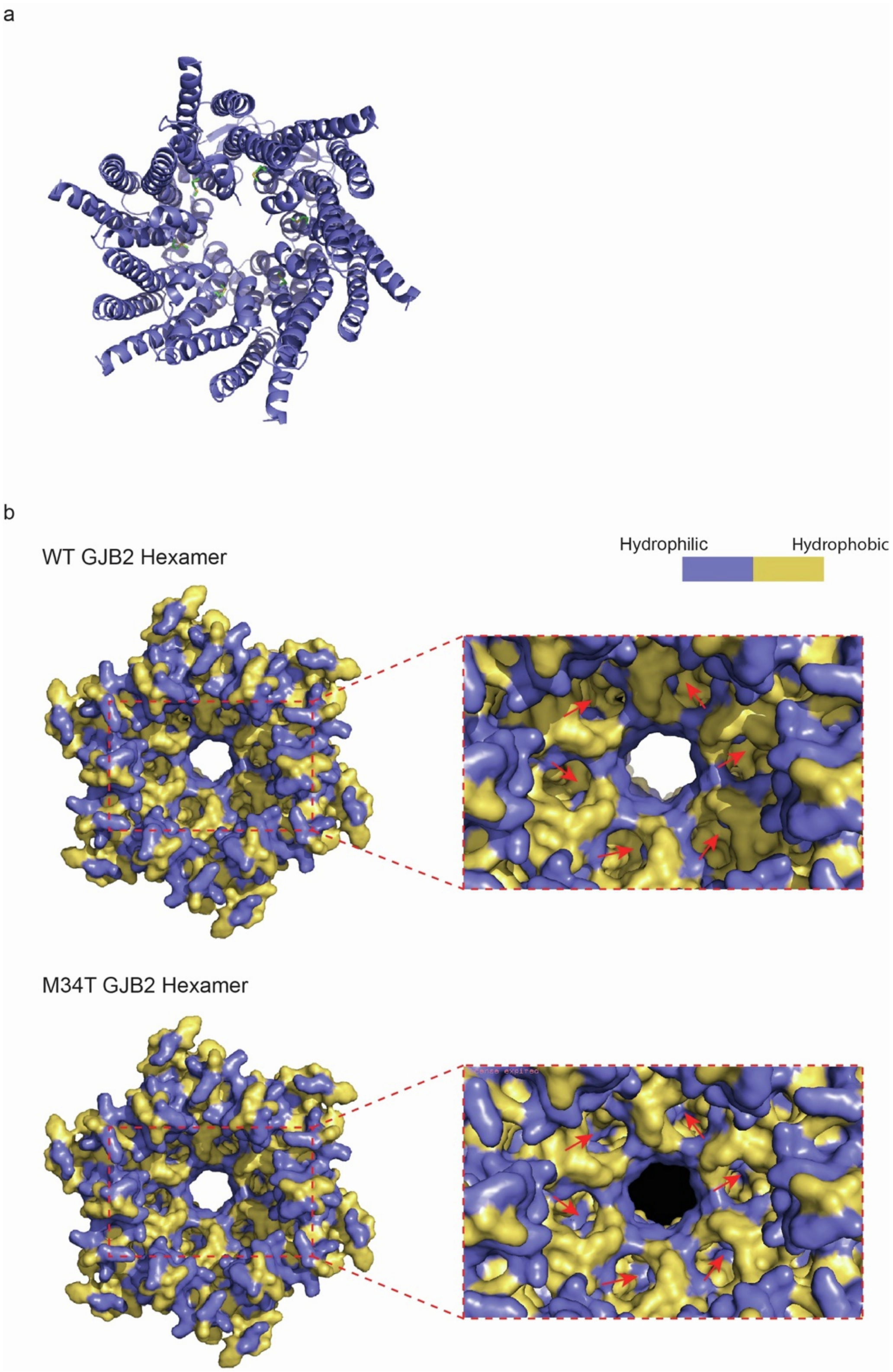

Figure S6. Genetic correlation between age-related hearing impairment and selected number of traits and diseases based on relevance in LDHub. Black filled circles pass the Bonferroni corrected significance threshold (p < 2e-04); bars represent standard error.

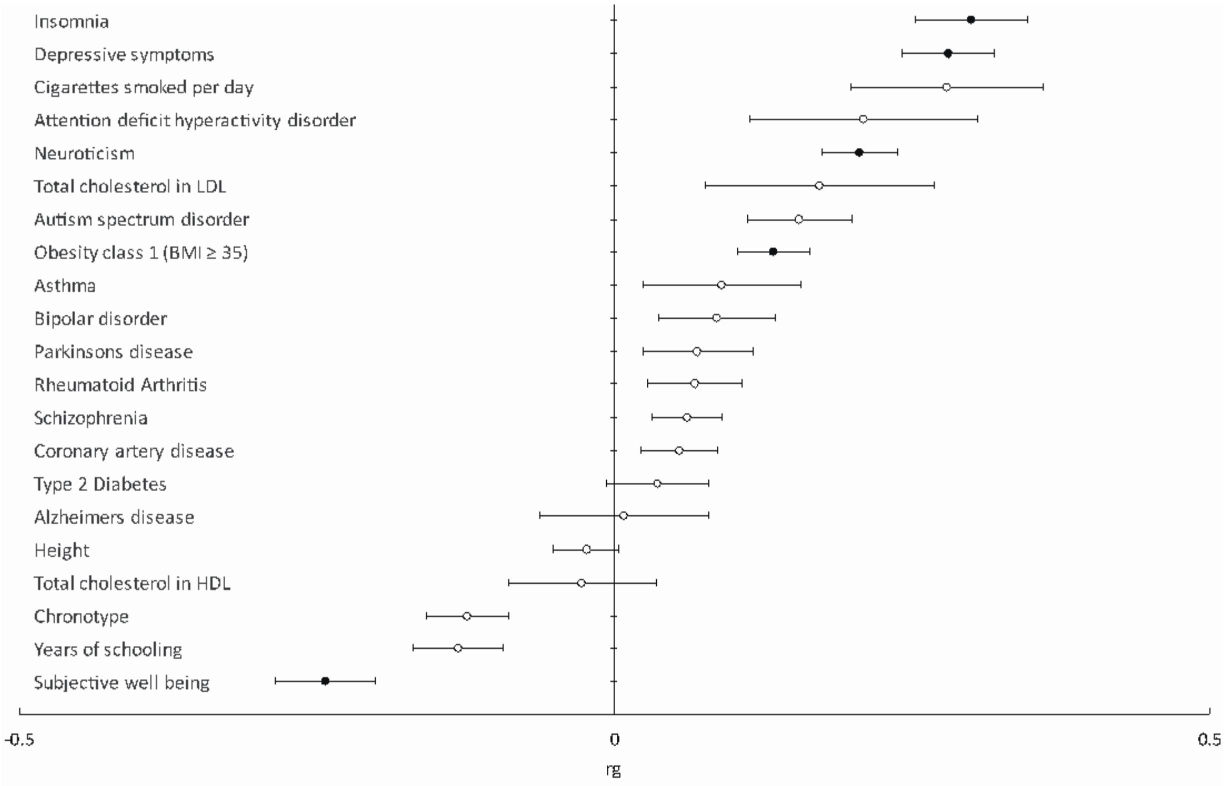

Figure S7. Genetic correlation between age-related hearing impairment and selected GWAS traits based on clinical relevance, collected by the Psychiatric Genomics Consortium. Black filled circles pass the Bonferroni corrected significance threshold ($p < 0.001$); bars represent standard error.

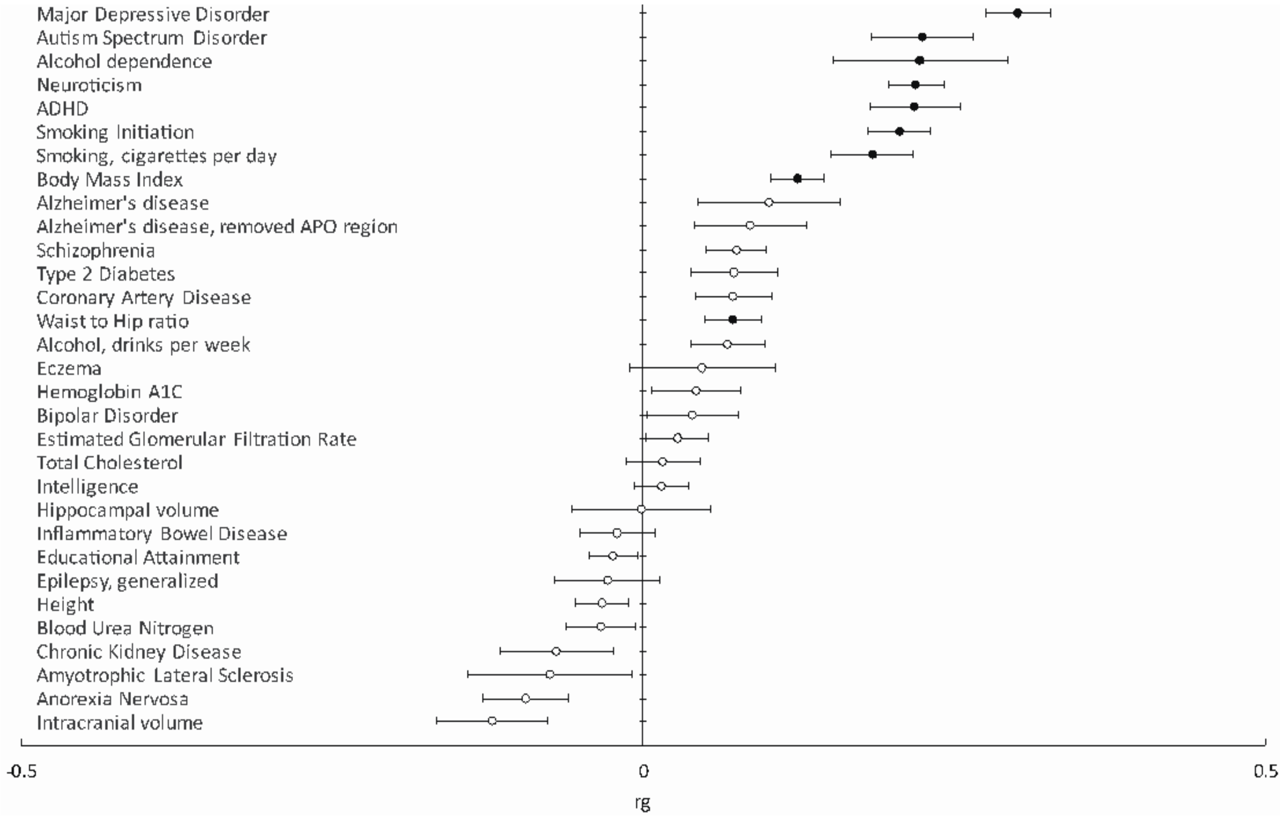

Figure S8. Tissue-set enrichment analysis of GTEx V8 tissues using MAGMA and partitioned LDSC. Bonferroni corrected significance threshold of -log10P-mean > 2.86. Bonferroni corrected significance threshold for MAGMA and LDSC P < 0.001

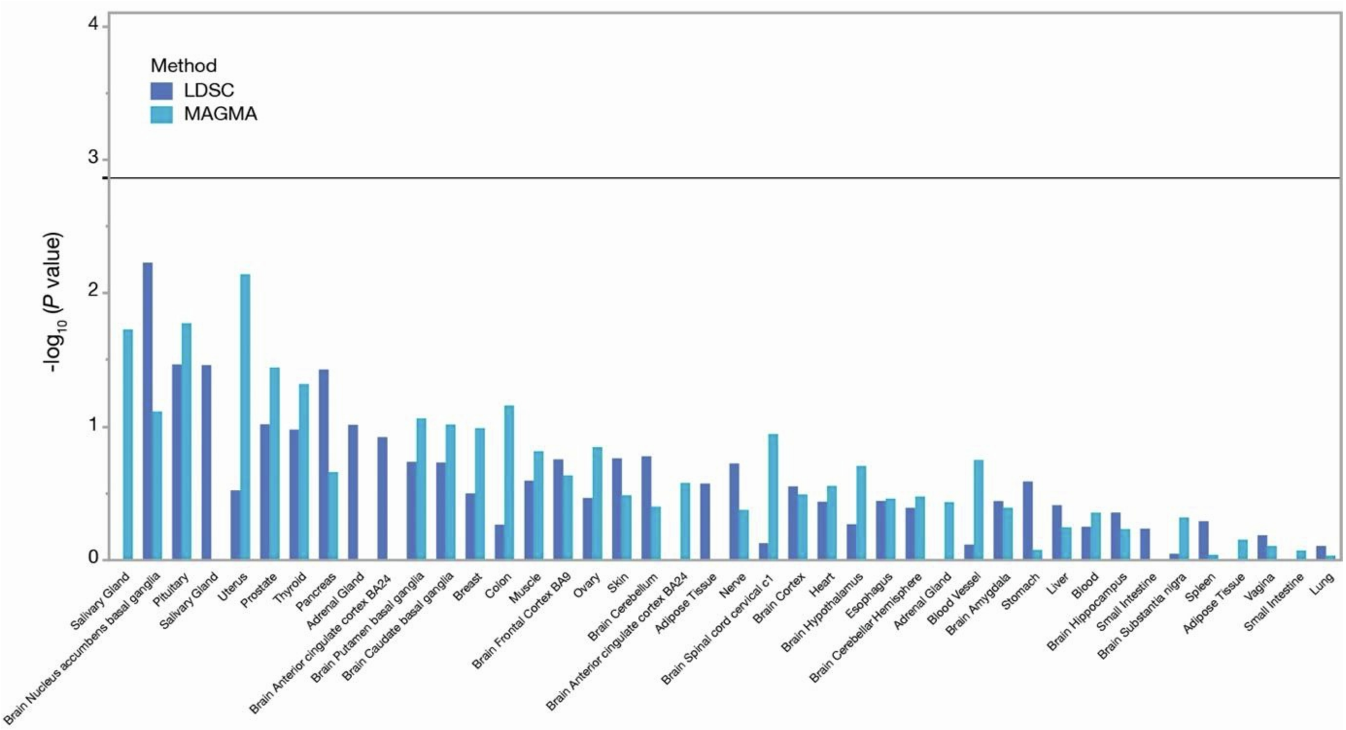

Figure S9. Density plot and b) bar plot demonstrating the proportion of missense variants in GWAS studies with more than 10 significant loci obtained from GWAS catalog. Fisher exact test p-values = 0.005

a)

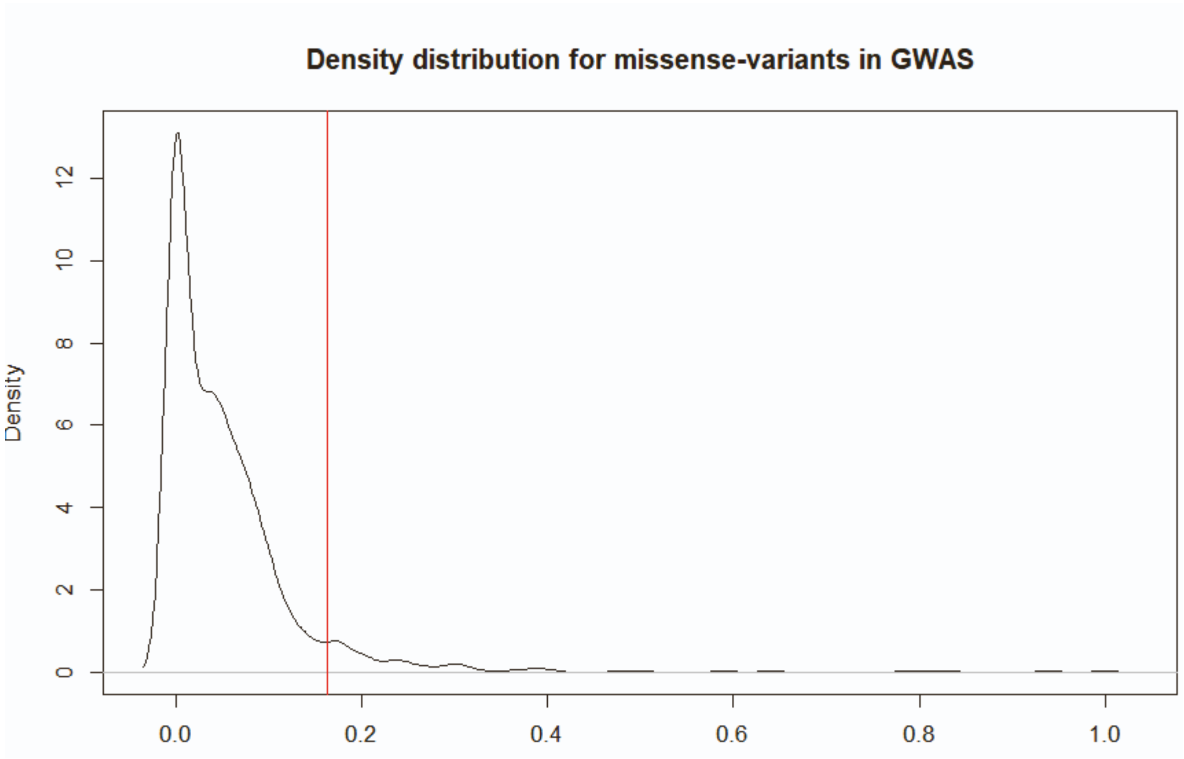

b)

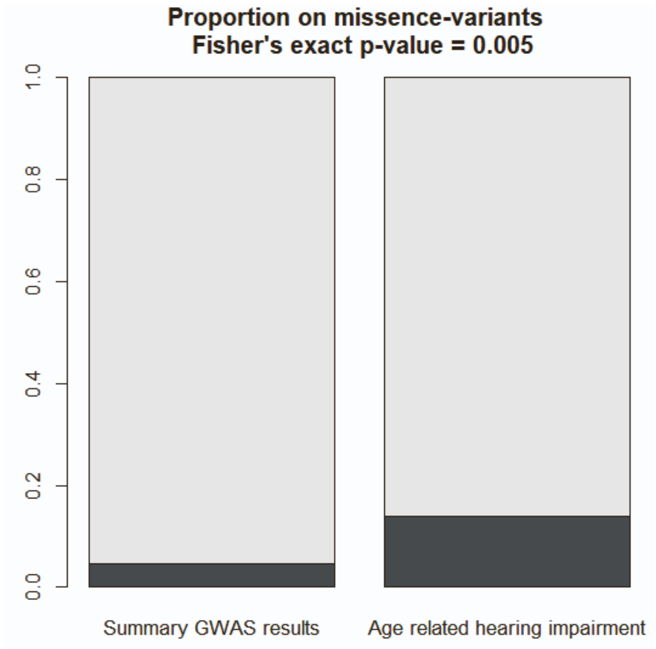

Supplementary Methods

Study Descriptions

Age, Gene/Environment Susceptibility Reykjavik Study: AGES-Reykjavik Study
The Reykjavik Study cohort originally comprised a random sample of 30,795 men and women born in 1907-1935 and living in Reykjavik in 1967[17]. A total of 19,381 people attended, resulting in 71% recruitment rate. The study sample was divided into six groups by birth year and birth date within month. One group was designated for longitudinal follow up and was examined in all stages. One group was designated a control group and was not included in examinations until 1991. Other groups were invited to participate in specific stages of the study. Between 2002 and 2006, the AGES-Reykjavik study re-examined 5764 survivors of the original cohort who had participated before in the Reykjavik Study.

The Danish Twin Registry
The Danish Twin Registry (DTR) sample included 1,314 individuals collected as part of the study of Middle Age Danish Twins (MADT, N=1,055) and the Longitudinal Study of Aging Danish Twins (LSADT, N=259)[18]. MADT was initiated in 1998 and includes 4,314 twins randomly chosen from the birth years 1931-1952. Surviving participants were revisited from 2008 to 2011, where the blood samples used in the present study were collected. The survey data used in the present study was obtained from the Omnibus 2 survey undertaken in 2002[18]. LSADT was initiated in 1995 and includes twins aged 70 years and older. Follow-up assessments were conducted every second year through 2005. The individuals included here all participated in the 1997 assessment, where blood samples were collected from same sex twin pairs, and in the 2001 assessment where the survey data used in the present study was collected[18].
Written informed consents were obtained from all participants. Collection and use of biological material, and survey and registry information were approved by the Regional Scientific Ethical Committees for Southern Denmark (MADT: S-VF-19980072, LSADT: S-VF-20040241). The study *is registered in SDU's internal list (notification no. 10.903) and complies with the rules in the General Data Protection Regulation.*

EGCUT
EstBB is a population-based cohort of 200,000 participants with a rich variety of phenotypic and health-related information collected for each individual[19]. At recruitment, participants have signed a consent to allow follow-up linkage of their electronic health records (EHR), thereby providing a longitudinal collection of phenotypic information. EstBB allows access to the records of the national Health Insurance Fund Treatment Bills (from 2004), Tartu University Hospital (from 2008) and North Estonia Medical Center (from 2005). For every participant there is information on diagnoses in ICD-10 coding and drug dispensing data, including drug ATC codes, prescription status and purchase date (if available).
At the time of this study 155,772 genotyped samples from the Estonian Biobank were available and genotyping was done at the Genotyping Core Facility of the Institute of Genomics, University of Tartu, using the Global Screening Array (GSAv1.0, GSAv2.0, and GSAv2.0_EST) from Illumina. Altogether 155,772 samples were genotyped and PLINK format files were exported using GenomeStudio v2.0.4. During the quality control all individuals with call-rate < 95% or mismatching sex that was defined based on the heterozygosity of X chromosome and sex in the phenotype data, were excluded from the analysis. Variants were filtered by call-rate < 95% and HWE p-value < 1e-4 (autosomal variants only). Variant positions were updated to Genome Reference Consortium Human Build 37 and all variants were changed to be from TOP strand using reference information

provided by Dr. Will Rayner from the University of Oxford (https://www.well.ox.ac.uk/~wrayner/strand/). Before imputation variants with MAF<1% and Indels were removed. Prephasing was done using the Eagle v2.3 software[20] (number of conditioning haplotypes Eagle2 uses when phasing each sample was set to: --Kpbwt=20000) and imputation was carried out using Beagle v.28Sep18.793[21,22] with an effective population size ne=20,000. As a reference, Estonian population specific imputation reference of 2,297 WGS samples was used[23]. For the current study, we determined 5,717 cases of hearing loss based on the participants EHRs as individuals with the ICD10 codes H90.3 or H91.1 and included only subjects of 45 years of age and above. We excluded among cases individuals with additional records of any of the following ICD10 diagnosis: H80, H81.0, H83, H90.4, H91.0, H91.2, H91.3, H93.0, H93.3, H94*, H95. Controls (n=49,806) were individuals 45 years of age and above and defined as undiagnosed participants. Individuals with records of H65, H66, H67, H68, H69, H70, H71, H72, H73, H74,H75,H80, H81.0, H83, H90, H91, H92, H93, H95, Q16, Z45.3,Z46.1,Z96.2,Z97.4 were removed among controls. We conducted the GWASes using the SAIGE software[24] adjusting for the first ten principal components of the genotype matrix, as well as for age and sex.

FinnGen

The FinnGen research project (www.finngen.fi) was launched in 2017 with an aim to improve human health through genetic research. The project combines genome information with digital health care data from national registries: the genotype data are linked to national hospital discharge, death, cancer, and medication reimbursement registries using the national personal identification numbers. The FinnGen study will combine approximately 200,000 existing samples from Finnish biobanks with approximately 300,000 samples from ongoing collections. Once final, the data resource will cover roughly 10% of the Finnish population. The present study comprised data of 212,544 Finnish adults (15,952 cases; 196,592 controls) from FinnGen Preparatory Phase Data Freeze 5.

Patients and control subjects in FinnGen provided informed consent for biobank research, based on the Finnish Biobank Act. Alternatively, separate research cohorts, collected prior the Finnish Biobank Act came into effect (in September 2013) and start of FinnGen (August 2017), were collected based on study-specific consents and later transferred to the Finnish biobanks after approval by Fimea, the National Supervisory Authority for Welfare and Health. Recruitment protocols followed the biobank protocols approved by Fimea. The Coordinating Ethics Committee of the Hospital District of Helsinki and Uusimaa (HUS) approved the FinnGen study protocol Nr HUS/990/2017.

The FinnGen study is approved by Finnish Institute for Health and Welfare (THL), approval number THL/2031/6.02.00/2017, amendments THL/1101/5.05.00/2017, THL/341/6.02.00/2018, THL/2222/6.02.00/2018, THL/283/6.02.00/2019, THL/1721/5.05.00/2019, Digital and population data service agency VRK43431/2017-3, VRK/6909/2018-3, VRK/4415/2019-3 the Social Insurance Institution (KELA) KELA 58/522/2017, KELA 131/522/2018, KELA 70/522/2019, KELA 98/522/2019, and Statistics Finland TK-53-1041-17.

The Biobank Access Decisions for FinnGen samples and data utilized in FinnGen Data Freeze 5 include: THL Biobank BB2017_55, BB2017_111, BB2018_19, BB_2018_34, BB_2018_67, BB2018_71, BB2019_7, BB2019_8, BB2019_26, Finnish Red Cross Blood Service Biobank 7.12.2017, Helsinki Biobank HUS/359/2017, Auria Biobank AB17-5154, Biobank Borealis of Northern Finland_2017_1013, Biobank of Eastern Finland 1186/2018, Finnish Clinical Biobank Tampere MH0004, Central Finland Biobank 1-2017, and Terveystalo Biobank STB 2018001.

Framingham Heart Study

The Framingham Heart Study is a prospective longitudinal investigation of the development of atherosclerosis and its clinical sequelae. Study participants were recruited at three time periods. The study was initiated in 1948-50 with the recruitment of 5209 individuals ages 28-62 (including some spouse pairs, parent-offspring pairs and siblings) for the purpose of investigating the multiple factors involved in the development of cardiovascular disease[25]. This group, known as the Original Cohort, has been examined every two years with a total of thirty-two examinations to date. In 1971-1975, offspring of the Original Cohort and the offspring spouses were recruited to examine among other goals the familial components of cardiovascular disease and its risk factors[26]. In 2002-2005, the third generation (children of the Offspring and grandchildren of the Original Cohort) was recruited[27]. The Offspring Cohort totalled 5124 and the Third Generation totalled 4095 at recruitment and have been examined every 4 to 8 years. The Offspring Cohort now has 9 examinations completed and the Third Generation has 2 examinations completed.
Between 1973 and 1975, hearing examinations were conducted on 2293 members of the original cohort, and between 1995 and 1999, identical examinations were conducted on 2262 members of the offspring cohort. Standard pure-tone audiograms were obtained on all participants using environments and meeting American National Standards Institute standards.

G-EAR

Within the International consortium called G-EAR, we used, in this study, individuals coming from two isolated cohorts: **Friuli Venezia Giulia (FVG)** Genetic Park and the **Salus in Apulia Study** (formerly known as Great Age Study).
The FVG cohort is a collection of samples coming from six small villages (Clauzetto, Erto, Illegio, Resia, San Martino del Carso and Sauris) located in north-eastern Italy, in the Friuli Venezia Giulia region[28]. The FVG Genetic Park is part of the INGI project, a collaboration between research institutions in Italy aimed at reconstructing the molecular bases of complex traits by investigating genetically isolated Italian populations. Studies were conducted referring to a common operational protocol. In each population, genotype samples were collected, alongside a detailed anamnesis, more than 120 biochemical parameters and 400 phenotypes, including anthropometric measures, lifestyle habits, diseases and pure-tone audiometry[29].
The “Salus in Apulia Study” is an ongoing population-based prospective cohort comprising 2,472 individuals aged >= 65 years and residents in Castellana Grotte, a town located near Bari, Puglia, in the Southeast of Italy. It focused on the sequence of lifestyle including diet, frailty, and other age-related impairments and age-related disease outcomes. In detail, Salus is a public health initiative funded by the Italian Ministry of Health and Apulia Regional Government and carried on at IRCCS “S. De Bellis” that combines data from two previous populations: the baseline data (MICOL3, M3) were recorded from 2003 to 2005 and the follow-up data from 2013 to 2015 (GreatAGE Study - MICOL4, M4). The GreatAGE-M4 study has been described elsewhere[30]. The invitation included also subjects of the MICOL studies that were in the respective age range above 64 years. In the GreatAge-M4 examination, in addition to the assessment of clinical and lifestyle aspects, sensory-related outcomes have been also evaluated together with neuropsychological features and genetic components. The study adhered to the “Standards for Reporting Diagnostic Accuracy Studies” (STARD) guidelines (http://www.stard-statement.org/), the “Strengthening the Reporting of Observational Studies in Epidemiology” (STROBE) guidelines (https://www.strobe-statement.org/).

Health, Aging, and Body Composition (HABC) Study

The HABC Study is a NIA-sponsored cohort study of the factors that contribute to incident disability and the decline in function of healthier older persons, with a particular emphasis on changes in body composition in old age. Between March 1997 and July 1998, 3075 70-79 year-old community-dwelling adults (41% African-American) were recruited to participate in the Health ABC Study.

Medicare beneficiary listings were used to recruit in metropolitan areas surrounding Pittsburgh, Pennsylvania, and Memphis, Tennessee. Eligibility criteria included having no difficulty walking one-quarter of a mile, climbing 10 steps, or performing activities of daily living (transferring, bathing, dressing, and eating); no history of active treatment for cancer in the prior 3 years; and no plans to move from the area within 3 years.

The Rotterdam Study

The Rotterdam study is a prospective, population-based cohort study among inhabitants of Ommoord, a district of Rotterdam, The Netherlands[31]. As of 2008, 14,926 subjects aged 45 years or over comprise the cohort. Since 2016, it is being expanded by persons aged 40 years and over. The Rotterdam study targets cardiovascular, endocrine, hepatic, neurological, ophthalmic, psychiatric, dermatological, otolaryngological, locomotor, and respiratory diseases. The participants were all examined in some detail at baseline. They were interviewed at home (2 h) and then had an extensive set of examinations (a total of 5 h) in a specially built research facility in the center of the district. Written informed consent was obtained from all participants and the Medical Ethics Committee of the Erasmus Medical Center, Rotterdam, approved the study.

The Swedish Twin Registry

The Swedish Twin Registry (STR) sample included in this study was recruited among Swedish born twins participating in two main national ascertainments; 1) the Screening Across the Lifespan Twin Study (SALT), which was a telephone interview study conducted in 1998-2002, covering twins born before 1959 and 2) Study of Twin Adults: Genes and Environment (STAGE), an online questionnaire study conducted 2005-2006, covering twins born 1959-1985[32]. Phenotypic information about hearing was collected though self-reports in these studies. Additional phenotypic information was available from a paper questionnaire (called SALTY) administered during 2009 to SALT participants born 1944-1985. DNA was collected from blood or saliva, and extracted by standard procedures. All genotyping was performed by SNP&SEQ genotyping facility in Uppsala, in three waves using three different Illumina chip arrays, OmniExpress for the blood DNA, Psychchip and Global screening array for the saliva DNA samples. Written informed consents were obtained from all participants.

TwinsUK

TwinsUK is the only adult twin registry in the UK, comprising of over 12,000 healthy twin volunteers aged 16-98[33]. Collection of data and biologic materials commenced in 1992 and is ongoing. Twins have completed detailed health and lifestyle questionnaires, and attended clinical evaluations. The pure tone audiometry data was collected on a subset of the cohort (N=1242) between April 2010 and November 2012. Participants were recruited with an aim to study aging in females. An air-conduction pure-tone audiogram was conducted by trained personnel using a Madsen XETA audiometer including TDH39 headphones. All research was conducted according to the ethical standards as defined by the Helsinki declaration. Ethical approval for this study was obtained from the National Research Ethics service London-Westminster (REC reference number: 07/H0802/84). Written informed consent was obtained from all participants prior to study conduction. Participants were excluded from analysis based on missing data, male, age <45. There were 819 female participants aged >45 remaining for analysis.

Women's Genome Health Study (WGHS)

WGHS is a prospective cohort of female North American health care professionals representing participants in the Women's Health Study (WHS) trial who provided a blood sample at baseline and consent for blood-based analyses[34]. Participants in the WHS were 45 years or older at enrollment and free of cardiovascular disease, cancer or other major chronic illness. The current data are derived from 23,294 WGHS participants for whom whole genome genotype information was available at the time of analysis and for whom self-reported European ancestry could be confirmed

by multidimensional scaling analysis of 1,443 ancestry informative markers in PLINK v. 1.06. At baseline, BP and lifestyle habits related to smoking, consumption of alcohol, and physical activity as well as other general clinical information were ascertained by a self-reported questionnaire, an approach which has been validated in the WGHS demographic, namely female health care professionals.

Information on hearing loss in the WHS (the WGHS parent cohort) was collected by self-report on the fourth observational questionnaire in 2008. Participants were asked: "As you age, do you have more trouble hearing in a crowded room?" with choices of "yes" or "no" as a response, encoded as 1 or 0, respectively.

Additional acknowledgements

The **AGES-Reykjavik** study has been funded by NIH contract N01-AG012100, the NIA Intramural Research Program, an Intramural Research Program Award (ZIAEY000401) from the National Eye Institute, an award from the National Institute on Deafness and Other Communication Disorders (NIDCD) Division of Scientific Programs (IAA Y2-DC_1004-02), Hjartavernd (the Icelandic Heart Association), and the Althingi (the Icelandic Parliament). The study is approved by the Icelandic National Bioethics Committee, VSN: 00-063. The researchers are indebted to the participants for their willingness to participate in the study.

DTR is supported by grants from The National Program for Research Infrastructure 2007 from the Danish Agency for Science, Technology and Innovation (09-063256) and the US National Institutes of Health (P01 AG08761). Genotyping was supported by NIH R01 AG037985 (Pedersen).

EstBB thanks all participants and staff of the Estonian biobank for their contribution to this research and the analytical work of EstBB was carried out in part in the High Performance Computing Center of the University of Tartu. We acknowledge the work of the Estonian Biobank Research Team: Andres Metspalu, Mari Nelis, Reedik Mägi and Tõnu Esko. The work by KK and LM was supported by the European Union through the European Regional Development Fund (Project No. 2014-2020.4.01.15-0012).

The **FinnGen** project is funded by two grants from Business Finland (HUS 4685/31/2016 and UH 4386/31/2016) and the following industry partners: AbbVie Inc., AstraZeneca UK Ltd, Biogen MA Inc., Bristol Myers Squibb (and Celgene Corporation & Celgene International II Sàrl), Genentech Inc., Merck Sharp & Dohme Corp, Pfizer Inc., GlaxoSmithKline Intellectual Property Development Ltd., Sanofi US Services Inc., Maze Therapeutics Inc., Janssen Biotech Inc, Novartis AG, and Boehringer Ingelheim. Following biobanks are acknowledged for delivering biobank samples to FinnGen: Auria Biobank (www.auria.fi/biopankki), THL Biobank (www.thl.fi/biobank), Helsinki Biobank (www.helsinginbiopankki.fi), Biobank Borealis of Northern Finland (https://www.ppshp.fi/Tutkimus-ja-opetus/Biopankki/Pages/Biobank-Borealis-briefly-in-English.aspx), Finnish Clinical Biobank Tampere (www.tays.fi/en-US/Research_and_development/Finnish_Clinical_Biobank_Tampere), Biobank of Eastern Finland (www.ita-suomenbiopankki.fi/en), Central Finland Biobank (www.ksshp.fi/fi-FI/Potilaalle/Biopankki), Finnish Red Cross Blood Service Biobank (www.veripalvelu.fi/verenluovutus/biopankkitoiminta) and Terveystalo Biobank (www.terveystalo.com/fi/Yritystietoa/Terveystalo-Biopankki/Biopankki/). All Finnish Biobanks are members of BBMRI.fi infrastructure (www.bbmri.fi). Finnish Biobank Cooperative -FINBB (https://finbb.fi/) is the coordinator of BBMRI-ERIC operations in Finland. The Finnish biobank data can be accessed through the Fingenious® services (https://site.fingenious.fi/en/) managed by FINBB.

The researchers would like to acknowledge all participants and investigators of the FinnGen study.

The **Framingham Heart Study** is conducted and supported by the National Heart, Lung, and Blood Institute (NHLBI) in collaboration with Boston University (Contract No. N01-HC-25195 and HHSN268201500001). This manuscript was not prepared in collaboration with investigators of the Framingham Heart Study and does not necessarily reflect the opinions or views of the Framingham Heart Study, Boston University, or NHLBI. Funding for SHARe Affymetrix genotyping was provided by NHLBI Contract N02-HL- 64278. Nancy L. Heard-Costa is supported by NIH/NHLBI HHSN268201500001 (Ramachandran). Further support was provided by P30AG066546, funded by the National Institute on Aging (HHS - NIH), awarded to University Of Texas Health Science Center Of San Antonio.

The Great Age study, Salus in Apulia study, was funded by Apulia Government and Italian Ministry of Health, under the Studies on Aging Network, at Italian Research Hospitals (IRCCS). The authors thank the MICOL Study group, the Salus in Apulia Research Team, and the General Practitioners of Castellana Grotte for the fundamental role in recruiting participants to this study.
The **Health, Aging and Body Composition Study** was supported by National Institute on Aging (NIA) Contracts N01-AG-6-2101; N01-AG-6-2103; N01-AG-6-2106; NIA grant R01-AG028050, and NINR grant R01-NR012459. This research was supported in part by the Intramural Research Program of the NIH, National Institute on Aging (NIA), National Institutes of Health, Department of Health and Human Services; project number ZO1 AG000535, as well as the National Institute of Neurological Disorders and Stroke.
The **Rotterdam Study** is funded by Erasmus Medical Center and Erasmus University, Rotterdam, Netherlands Organisation for the Health Research and Development (ZonMw), the Research Institute for Diseases in the Elderly (RIDE), the Ministry of Education, Culture and Science, the Ministry for Health, Welfare and Sports, the European Commission (DG XII), and the Municipality of Rotterdam. The authors are grateful to the study participants, the staff from the Rotterdam Study and the participating general practitioners and pharmacists. The generation and management of GWAS genotype data for the Rotterdam Study (RS I, RS II, RS III) was executed by the Human Genotyping Facility of the Genetic Laboratory of the Department of Internal Medicine, Erasmus MC, Rotterdam, The Netherlands. The GWAS datasets are supported by the Netherlands Organisation of Scientific Research NWO Investments (nr. 175.010.2005.011, 911-03-012), the Genetic Laboratory of the Department of Internal Medicine, Erasmus MC, the Research Institute for Diseases in the Elderly (014-93-015; RIDE2), the Netherlands Genomics Initiative (NGI)/Netherlands Organisation for Scientific Research (NWO) Netherlands Consortium for Healthy Aging (NCHA), project nr. 050-060-810. We thank Pascal Arp, Mila Jhamai, Marijn Verkerk, Lizbeth Herrera and Marjolein Peters, MSc, and Carolina Medina-Gomez, MSc, for their help in creating the GWAS database, and Karol Estrada, PhD, Yurii Aulchenko, PhD, and Carolina Medina-Gomez, MSc, for the creation and analysis of imputed data.
TwinsUK: The authors of this paper wish to express our appreciation to all study participants of the TwinsUK cohort. TwinsUK is funded by the Wellcome Trust, Medical Research Council, European Union, the National Institute for Health Research (NIHR)-funded BioResource, Clinical Research Facility and Biomedical Research Centre based at Guy's and St Thomas' NHS Foundation Trust in partnership with King's College London. HRRW is funded by Action on Hearing Loss, CJS is funded under a grant from the Chronic Disease Research Foundation (CDRF). FMKW is supported by Arthritis Research UK grant number 20682.
The **Women's Genome Health Study** is supported by the National Heart, Lung, and Blood Institute (HL043851 and HL080467) and the National Cancer Institute (CA047988 and UM1CA182913), with funding for genotyping provided by Amgen.

We are also thankful to all patients and participants in the various cohorts, and Fanny Stulz for graphic support. UK Biobank data has been used under the project # 11516. The computations and data handling were enabled by resources provided by the Swedish National Infrastructure for Computing (SNIC) at SNIC/UPPMAX partially funded by the Swedish Research Council through grant agreement no. 2018-05973. We acknowledge The Swedish Twin Registry for access to data. The Swedish Twin Registry is managed by Karolinska Institutet and receives funding through the Swedish Research Council under the grant no 2017-00641.

Supplementary references

1. Wesdorp, M., *et al.* Heterozygous missense variants of LMX1A lead to nonsyndromic hearing impairment and vestibular dysfunction. *Hum Genet* **137**, 389-400 (2018).
2. Liu, Y., *et al.* Critical role of spectrin in hearing development and deafness. *Sci Adv* **5**, eaav7803 (2019).
3. Xu, F., Shan, S., Sommerlad, S., Seddon, J.M. & Brenig, B. A Missense Mutation in the KLF7 Gene Is a Potential Candidate Variant for Congenital Deafness in Australian Stumpy Tail Cattle Dogs. *Genes (Basel)* **12**(2021).
4. Vona, B., *et al.* A biallelic variant in CLRN2 causes non-syndromic hearing loss in humans. *Hum Genet* **140**, 915-931 (2021).
5. Manji, S.S., *et al.* A mutation in synaptojanin 2 causes progressive hearing loss in the ENU-mutagenised mouse strain Mozart. *PLoS One* **6**, e17607 (2011).
6. Stover, E.H., *et al.* Novel ATP6V1B1 and ATP6V0A4 mutations in autosomal recessive distal renal tubular acidosis with new evidence for hearing loss. *J Med Genet* **39**, 796-803 (2002).
7. Han, S.Y., Kim, S., Shin, D.H., Cho, J.H. & Nam, S.I. The Expression of AGO2 and DGCR8 in Idiopathic Sudden Sensorineural Hearing Loss. *Clin Exp Otorhinolaryngol* **7**, 269-274 (2014).
8. Uthaiah, R.C. & Hudspeth, A.J. Molecular anatomy of the hair cell's ribbon synapse. *J Neurosci* **30**, 12387-12399 (2010).
9. Ohlemiller, K.K., *et al.* Progression of cochlear and retinal degeneration in the tubby (rd5) mouse. *Audiol Neurootol* **2**, 175-185 (1997).
10. Du, T.T., *et al.* LMO7 deficiency reveals the significance of the cuticular plate for hearing function. *Nat Commun* **10**, 1117 (2019).
11. Fredrich, M. & Illing, R.B. MMP-2 is involved in synaptic remodeling after cochlear lesion. *Neuroreport* **21**, 324-327 (2010).
12. Wu, J., *et al.* Matrix metalloproteinase-2 and -9 contribute to functional integrity and noiseinduced damage to the blood-labyrinth-barrier. *Mol Med Rep* **16**, 1731-1738 (2017).
13. Shin, J.B., *et al.* The R109H variant of fascin-2, a developmentally regulated actin crosslinker in hair-cell stereocilia, underlies early-onset hearing loss of DBA/2J mice. *J Neurosci* **30**, 9683-9694 (2010).
14. Wang, Y., *et al.* Blast-induced hearing impairment in rats is associated with structural and molecular changes of the inner ear. *Sci Rep* **10**, 10652 (2020).
15. Asamura, K., Abe, S., Fukuoka, H., Nakamura, Y. & Usami, S. Mutation analysis of COL9A3, a gene highly expressed in the cochlea, in hearing loss patients. *Auris Nasus Larynx* **32**, 113-117 (2005).
16. Carlton, A.J., *et al.* Loss of Baiap2l2 destabilizes the transducing stereocilia of cochlear hair cells and leads to deafness. *J Physiol* **599**, 1173-1198 (2021).
17. Harris, T.B., Launer, L.J., Eiriksdottir, G., Kjartansson, O., Jonsson, P.V., Sigurdsson, G., Thorgeirsson, G., Aspelund, T., Garcia, M.E., Cotch, M.F., et al. (2007). Age, Gene/Environment Susceptibility-Reykjavik Study: multidisciplinary applied phenomics. Am J Epidemiol 165, 1076-1087.
18. Pedersen, D.A., Larsen, L.A., Nygaard, M., Mengel-From, J., McGue, M., Dalgard, C., Hvidberg, L., Hjelmborg, J., Skytthe, A., Holm, N.V., et al. (2019). The Danish Twin Registry: An Updated Overview. Twin Res Hum Genet 22, 499-507.

19. Leitsalu, L., Alavere, H., Tammesoo, M.L., Leego, E., and Metspalu, A. (2015). Linking a population biobank with national health registries-the estonian experience. J Pers Med 5, 96-106.
20. Loh, P.R., Danecek, P., Palamara, P.F., Fuchsberger, C., Y, A.R., H, K.F., Schoenherr, S., Forer, L., McCarthy, S., Abecasis, G.R., et al. (2016). Reference-based phasing using the Haplotype Reference Consortium panel. Nature genetics 48, 1443-1448.
21. Browning, B.L., Zhou, Y., and Browning, S.R. (2018). A One-Penny Imputed Genome from Next-Generation Reference Panels. Am J Hum Genet 103, 338-348.
22. Browning, S.R., and Browning, B.L. (2007). Rapid and accurate haplotype phasing and missing-data inference for whole-genome association studies by use of localized haplotype clustering. Am J Hum Genet 81, 1084-1097.
23. Mitt, M., Kals, M., Parn, K., Gabriel, S.B., Lander, E.S., Palotie, A., Ripatti, S., Morris, A.P., Metspalu, A., Esko, T., et al. (2017). Improved imputation accuracy of rare and low-frequency variants using population-specific high-coverage WGS-based imputation reference panel. Eur J Hum Genet 25, 869-876.
24. Zhou, W., Nielsen, J.B., Fritsche, L.G., Dey, R., Gabrielsen, M.E., Wolford, B.N., LeFaive, J., VandeHaar, P., Gagliano, S.A., Gifford, A., et al. (2018). Efficiently controlling for case-control imbalance and sample relatedness in large-scale genetic association studies. Nature genetics 50, 1335-1341.
25. Dawber, T.R., Kannel, W.B., and Lyell, L.P. (1963). An approach to longitudinal studies in a community: the Framingham Study. Ann N Y Acad Sci 107, 539-556.
26. Feinleib, M., Kannel, W.B., Garrison, R.J., McNamara, P.M., and Castelli, W.P. (1975). The Framingham Offspring Study. Design and preliminary data. Prev Med 4, 518-525.
27. Splansky, G.L., Corey, D., Yang, Q., Atwood, L.D., Cupples, L.A., Benjamin, E.J., D'Agostino, R.B., Sr., Fox, C.S., Larson, M.G., Murabito, J.M., et al. (2007). The Third Generation Cohort of the National Heart, Lung, and Blood Institute's Framingham Heart Study: design, recruitment, and initial examination. Am J Epidemiol 165, 1328-1335.
28. Esko, T., Mezzavilla, M., Nelis, M., Borel, C., Debniak, T., Jakkula, E., Julia, A., Karachanak, S., Khrunin, A., Kisfali, P., et al. (2013). Genetic characterization of northeastern Italian population isolates in the context of broader European genetic diversity. Eur J Hum Genet 21, 659-665.
29. Girotto, G., Pirastu, N., Sorice, R., Biino, G., Campbell, H., d'Adamo, A.P., Hastie, N.D., Nutile, T., Polasek, O., Portas, L., et al. (2011). Hearing function and thresholds: a genome-wide association study in European isolated populations identifies new loci and pathways. J Med Genet 48, 369-374.
30. Sardone, R., Battista, P., Donghia, R., Lozupone, M., Tortelli, R., Guerra, V., Grasso, A., Griseta, C., Castellana, F., Zupo, R., et al. (2020). Age-Related Central Auditory Processing Disorder, MCI, and Dementia in an Older Population of Southern Italy. Otolaryngol Head Neck Surg 163, 348-355.
31. Ikram, M.A., Brusselle, G., Ghanbari, M., Goedegebure, A., Ikram, M.K., Kavousi, M., Kieboom, B.C.T., Klaver, C.C.W., de Knegt, R.J., Luik, A.I., et al. (2020). Objectives, design and main findings until 2020 from the Rotterdam Study. Eur J Epidemiol 35, 483-517.
32. Zagai, U., Lichtenstein, P., Pedersen, N.L., and Magnusson, P.K.E. (2019). The Swedish Twin Registry: Content and Management as a Research Infrastructure. Twin Res Hum Genet 22, 672-680.
33. Moayyeri, A., Hammond, C.J., Valdes, A.M., and Spector, T.D. (2013). Cohort Profile: TwinsUK and healthy ageing twin study. Int J Epidemiol 42, 76-85.

34. Ridker, P.M., Chasman, D.I., Zee, R.Y., Parker, A., Rose, L., Cook, N.R., Buring, J.E., and Women's Genome Health Study Working, G. (2008). Rationale, design, and methodology of the Women's Genome Health Study: a genome-wide association study of more than 25,000 initially healthy american women. Clin Chem 54, 249-255.
